# A “Qualitative–Pharmacological–Correlation–Molecular” Integrated Workflow Reveals HIF-1*α*–Relevant Anti-Hypoxia Metabolites in *Rhodiola* Species

**DOI:** 10.3390/ijms27052203

**Published:** 2026-02-26

**Authors:** Yixuan Li, Changming Zhong, Nan Zhang, Namin Wei, Siyu Li, Wanjun Yang, Huanfei Yang, Fanlin Yang, Feiyu Li, Jing Shang, Mengrui Guo, Shuo Liu, Jiaqi Tan, Wanting Tang, Zhaojuan Guo, Huaqiang Zhai

**Affiliations:** 1 Standardization Research Center of Traditional Chinese Medicine Dispensing, School of Chinese Materia Medica, Beijing University of Chinese Medicine, Beijing 102488, China; yy978709589@163.com (Y.L.); zhongchangming0411@163.com (C.Z.); zhangnanjessie@163.com (N.Z.); weinamin1992@163.com (N.W.); 202453050@bucm.edu.cn (S.L.); 20220935257@bucm.edu.cn (W.Y.); 20230935271@bucm.edu.cn (H.Y.); 15940596950@163.com (F.Y.); flyfishi0329@163.com (F.L.); sj010907@163.com (J.S.); gguomengrui@163.com (M.G.); a13948967231@163.com (S.L.); 20240935232@bucm.edu.cn (J.T.); 18810595256@163.com (W.T.); 2 Beijing Research Institute of Chinese Medicine, Beijing University of Chinese Medicine, Beijing 102488, China

**Keywords:** *Rhodiola crenulata*, *Rhodiola kirilowii*, *Rhodiola rosea*, anti-hypoxia effect, identifying functional constituents

## Abstract

*Rhodiola* species are traditionally used to mitigate hypoxia-related symptoms, but comparative evidence on their chemical bases and active constituents is limited. We implemented an integrated “qualitative analysis–pharmacological exploration–correlation analysis–molecular validation” workflow to compare *Rhodiola crenulata*, *R. kirilowii*, and *R. rosea*. Ultra-high-performance liquid chromatography–Q Exactive mass spectrometry (UPLC-QE-MS) profiling identified 175 metabolites across the three species, of which 161 were shared; multivariate analyses (principal component analysis, PCA; partial least squares–discriminant analysis, PLS-DA) revealed 30 differential compounds. In a normobaric hypoxia mouse model using herbal powder solutions, all three species significantly increased survival time versus control (*p* < 0.05), with mean survival times of 48.16 min (RR), 47.07 min (RC), and 44.82 min (RK) compared with 44.34 min for the positive control. Chemometric correlation (partial least squares regression, PLSR) combined with grey relational analysis (GRA) prioritized 14 compounds consistently associated with anti-hypoxia efficacy; six representative metabolites—epicatechin, 3-O-galloylquinic acid, salidroside, p-coumaric acid-4-O-glucoside, citric acid, and geraniol—were selected for in silico assessment. Molecular docking against hypoxia-inducible factor-1*α* (HIF-1*α*) yielded favorable binding poses (docking scores < −4.0), providing preliminary molecular-level plausibility without claiming mechanistic proof. This multi-level approach clarifies chemical–pharmacological relationships among *Rhodiola* species and provides prioritized candidate compounds for targeted isolation and mechanistic validation.

## 1. Introduction

Hypoxia, defined as insufficient oxygen availability, represents a fundamental physiological stress that disrupts cellular energy metabolism, redox balance, and tissue homeostasis [1]. It is closely associated with cardiopulmonary diseases, ischemic injury, neurological dysfunction, and high-altitude intolerance, making hypoxia-related disorders a major biomedical concern [2]. The hypoxia-inducible factor-1 (HIF-1) pathway is central to cellular adaptation to oxygen deprivation. Within this pathway, HIF-1*α* functions as the principal oxygen-responsive regulatory subunit, and its level and activity determine how cells adjust metabolism, maintain viability, and preserve oxygen homeostasis under hypoxic conditions [3]. Owing to this central role, HIF-1*α* is widely recognized as a critical molecular target in the search for effective anti-hypoxia interventions. However, current clinical options for managing hypoxia remain limited [4]. Agents commonly employed—such as vasodilators, erythropoiesis-stimulating drugs, and diuretics—primarily modulate individual physiological pathways and therefore provide only partial relief from the complex stress responses triggered by hypoxia [5]. Their restricted target spectrum, modest protective effects, and potential adverse reactions highlight the urgent need for new pharmacological candidates capable of exerting broader, multi-dimensional regulation of hypoxia-related biological processes [6]. In this context, natural products with rich chemical diversity and inherent multitarget potential offer an important reservoir for discovering compounds that may influence pathways associated with oxygen adaptation, including those regulated by HIF-1*α*.

Among natural medicinal resources, plants of the genus *Rhodiola* (Crassulaceae) occupy a notable position [7]. Traditionally regarded as adaptogens, *Rhodiola* species have long been used to alleviate fatigue, enhance physical endurance, and counteract symptoms related to high-altitude exposure [8]. Contemporary pharmacological studies have corroborated their anti-hypoxia, antioxidant, and anti-inflammatory effects, which are attributed to diverse phytochemicals such as phenylpropanoids, flavonoids, and phenolic acids [9].

Among them, *Rhodiola crenulata* (*Rhodiola crenulata* (HK. f. et Thoms.) H. Ohba) is the species included in the Chinese Pharmacopoeia and serves as the principal medicinal source [10]. Yet, its restricted natural distribution and rising market demand have resulted in increasing pressure on wild populations [11]. Other species, including *Rhodiola kirilowii* (*Rhodiola kirilowii* (Regel) Maxim) and *Rhodiola* rosea (*Rhodiola rosea* L.), share similar traditional applications and contain comparable classes of metabolites, suggesting that they may possess related pharmacological potential [12]. Nevertheless, systematic evidence comparing the chemical characteristics and anti-hypoxia efficacy of these species remains insufficient, and the specific constituents most closely associated with their biological activity have not been clearly defined [13]. Advancing the understanding of *Rhodiola* requires an analytical framework capable of linking chemical diversity to pharmacological outcomes and molecular relevance. Prior studies often examined chemical profiles or biological activity in isolation, limiting the ability to identify compounds that contribute most strongly to anti-hypoxia effects.

To address these gaps, the present study establishes an integrative four-step strategy—“qualitative analysis—pharmacological exploration—correlation analysis—molecular validation”—to systematically investigate three representative *Rhodiola* species. By applying UPLC-QE-MS-based profiling, a normobaric hypoxia mouse model, multivariate chemometric analysis, and in silico examination of compound–target interactions, we aim to identify key metabolites associated with anti-hypoxia activity and to clarify interspecies differences among *R. crenulata*, *R. kirilowii*, and *R. rosea*. This multi-level approach provides a coherent path for linking phytochemical diversity with pharmacological relevance and offers scientific evidence to support the rational evaluation and utilization of *Rhodiola* resources.

## 2. Results

### 2.1. Chemical Composition Analysis of Three Rhodiola Species

#### 2.1.1. Qualitative Analysis of Components

**Figure 1:**
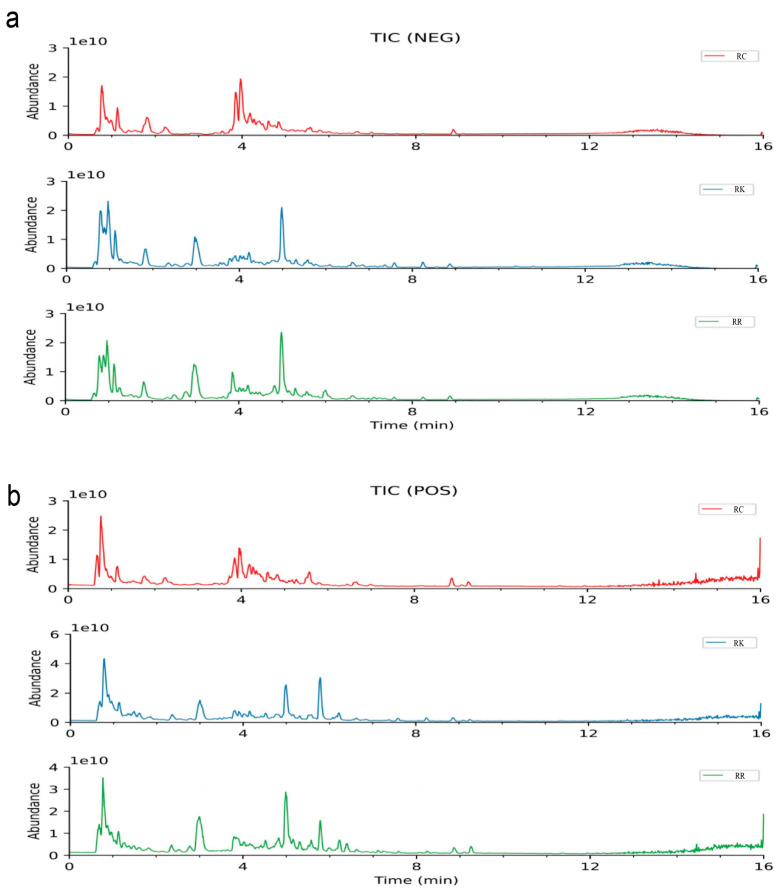
Total ion chromatograms of three *Rhodiola* species in positive and negative ion modes. (**a**) TIC of *R. crenulata*, *R. kirilowii*, and *R. rosea* in positive ion mode; (**b**) TIC of *R. crenulata*, *R. kirilowii*, and *R. rosea* in negative ion mode.

**Figure 2:**
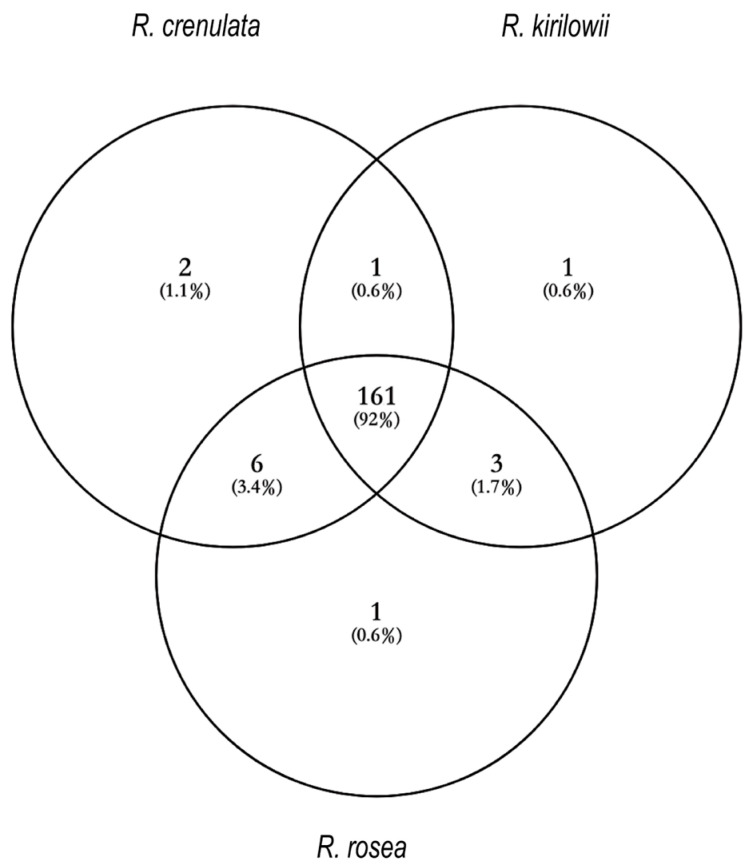
Venn diagram showing the number of metabolites detected in *R. crenulata, R. kirilowii*, and *R. rosea* based on LC–MS/MS qualitative analysis. Note: The Venn diagram reflects qualitative detection (presence/absence) rather than relative abundance.

Under the specified analytical conditions, the total ion chromatograms (TIC) of *R. crenulata*, *R. kirilowii*, and *R. rosea* were obtained in both positive and negative ion modes, as illustrated in Figure 1. Comprehensive qualitative analysis of the chemical constituents was conducted based on accurate mass-to-charge ratios (*m*/*z*) and secondary fragment ions. To provide an overview of the qualitative metabolite coverage among the three species, a Venn diagram was constructed to summarize the number of metabolites detected in each species and their overlap (Figure 2). The detailed results are summarized in the Table S1.

#### 2.1.2. Mass Spectrometric Fragmentation Characterization of Flavonoids

Flavonoids are a class of dibenzopyran derivatives widely found in plants, exhibiting various biological activities, including antioxidant, anti-inflammatory, anti-tumor, and cardiovascular protective effects [14]. A total of 24 flavonoid compounds were identified, including flavonols, procyanidins, flavones, flavanols, flavanones, and flavanonols.

Flavanonols identified include Taxifolin 7-O-*β*-d-glucoside (*m*/*z* 465.1035, [M−H]^−^) and Taxifolin (*m*/*z* 303.0508, [M−H]^−^). Flavones identified include Kaempferol-7-O-rhamnoside (*m*/*z* 433.1126, [M+H]^+^), Kaempferol 3-neohesperidoside (*m*/*z* 595.1655, [M+H]^+^), Kaempferol 3-sophoroside-7-glucoside (*m*/*z* 771.1988, [M−H]^−^), Luteolin (*m*/*z* 285.0403, [M−H]^−^), 6-Hydroxyluteolin (*m*/*z* 301.0360, [M−H]^−^), Luteolin 5-O-glucoside (*m*/*z* 449.1075, [M+H]^+^), and 7-[(*β*-d-Glucopyranosyl)oxy]-3′,4′,5,8-tetrahydroxyflavone (*m*/*z* 465.1025, [M+H]^+^).

Flavonols identified include Spiraeoside (*m*/*z* 465.1027, [M+H]^+^), Hibifolin (*m*/*z* 495.0766, [M+H]^+^), Ternatumoside II (*m*/*z* 595.1656, [M+H]^+^), Rhodiosin (*m*/*z* 611.1604, [M+H]^+^), Rhodionin (*m*/*z* 449.1073, [M+H]^+^), Hyperoside (*m*/*z* 465.1025, [M+H]^+^), Quercetin 3-sambubioside (*m*/*z* 595.1300, [M−H]^−^), and 6-Hydroxykaempferol 3,6-diglucoside (*m*/*z* 627.1553, [M+H]^+^).

Flavanols identified include Epicatechin (*m*/*z* 289.0715, [M−H]^−^), Gallocatechin (*m*/*z* 287.0559, [M−H−H_2_O]^−^), Catechin (*m*/*z* 291.0860, [M+H]^+^), Epigallocatechin (*m*/*z* 307.0809, [M+H]^+^), and Epicatechin gallate (*m*/*z* 443.0969, [M+H]^+^).

Flavanones identified include Taxifolin (*m*/*z* 303.0508, [M−H]^−^).

Procyanidins identified include Procyanidin B4 (*m*/*z* 579.1493, [M+H]^+^) and Procyanidin C1 (*m*/*z* 867.2125, [M+H]^+^). These compounds share a fundamental C_6_-C_3_-C_6_ skeleton. Flavonoid glycosides are difficult to lose sugar moieties directly and often undergo sugar ring-opening cleavage. Free flavonoid aglycones tend to lose fragment ions such as H_2_O, CH_3_, and CO_2_, or undergo RDA fragmentation via C-ring cleavage, generating characteristic fragment ions.

For instance, in Compound 23, the protonated molecular ion peak in the positive ion mode is observed at *m*/*z* 579.1493 [M+H]^+^. The fragment ion at *m*/*z* 427.1020 results from RDA cleavage of the F-ring, while *m*/*z* 411.1072 and *m*/*z* 123.0440 result from HRF cleavage of the C-ring. The fragment ion at *m*/*z* 291.0860 originates from cleavage of the C_4_-C_6_ bond of the parent ion. Based on reference standards, fragmentation rules, and the literature, this compound is identified as Procyanidin B4.

#### 2.1.3. Mass Spectrometric Fragmentation Characterization of Phenolic Compounds

A total of 11 compounds were identified, including monophenols and polyphenols. These compounds include 4-Hydroxyphenyl ethanol (*m*/*z* 139.0753, [M+H]^+^), 3-Hydroxybenzaldehyde (*m*/*z* 123.0442, [M+H]^+^), Isovanillin (*m*/*z* 153.0546, [M+H]^+^), Syringaldehyde (*m*/*z* 183.0651, [M+H]^+^), Purpurogallin (*m*/*z* 219.0294, [M−H]^−^), Gallic acid (*m*/*z* 171.0287, [M+H]^+^), 3,4-Dihydroxybenzaldehyde (*m*/*z* 139.0389, [M+H]^+^), 3,4,8,9,10-Pentahydroxy Urolithin (*m*/*z* 275.0196, [M−H]^−^), Octyl gallate (*m*/*z* 281.1393, [M−H]^−^), Demethylwedelolactone (*m*/*z* 299.0196, [M−H]^−^), and 3-Galloylquinic acid (*m*/*z* 345.0813, [M+H]^+^). These compounds are characterized by phenolic hydroxyl-substituted aromatic rings, acrylic acid derivatives, and amino acid compositions derived from lipids and terpenoids. They tend to exhibit higher responsiveness in negative ion mode and are prone to losing H_2_O and CO. For example, compound 28 shows a protonated molecular ion peak at *m*/*z* 183.0651 [M+H]^+^ in positive ion mode, with fragment ions at *m*/*z* 165.0546 [M+H−H_2_O]^+^ and *m*/*z* 137.0597 [M+H−H_2_O−CO], generated by the loss of a water molecule and CO, respectively. Based on reference standards, mass spectrometric fragmentation patterns, and the literature, it was identified as Syringaldehyde.

#### 2.1.4. Mass Spectrometric Fragmentation Characterization of Phenylpropanoids

The basic skeleton of phenylpropanoids consists of a benzene ring and a three-carbon side chain attached to the benzene ring, with the terminal end of the side chain usually being a carbonyl group. This basic structure varies among different phenylpropanoids due to differences in the side chain, oxidation level of the carbonyl group, and substituents on the benzene ring, leading to a diverse range of phenylpropanoid compounds [15]. A total of 17 compounds were identified, including simple phenylpropanoids such as Rosavin (*m*/*z* 429.1744, [M+H]^+^), phenylpropionic acids such as L-3-Phenyllactic acid (*m*/*z* 165.0548926, [M−H]^−^) and Hydroxyphenyllactic acid (*m*/*z* 181.0498, [M−H]^−^), stilbenes such as Isorhapontin (*m*/*z* 419.1347, [M−H]^−^) and Polydatin (*m*/*z* 435.1293, [M+HCOO]^−^, simple phenylpropanoids such as Cinnamyl Alcohol (*m*/*z* 117.0701, [M+H−H_2_O]^+^), Methyl p-coumarate (*m*/*z* 177.0549, [M−H]^−^), Coniferaldehyde (*m*/*z* 179.0702, [M+H]^+^), and Ferulic acid methyl ester (*m*/*z* 209.0807, [M+H]^+^), lignans such as (+)-Balanophonin (*m*/*z* 357.1329, [M+H]^+^) and Pinoresinol 4-O-*β*-d-glucopyranoside (*m*/*z* 519.1869, [M−H]^−^), and coumarins such as Isodemethylwedelolactone (*m*/*z* 301.0340, [M+H]^+^), 6-Hydroxy-4-methylcoumarin (*m*/*z* 194.0811, [M+NH_4_]^+^), and Dihydrocoumarin (*m*/*z* 149.0597, [M+H]^+^). Additionally, lignans such as Pinoresinol (*m*/*z* 341.1380636, [M+H−H_2_O]^+^), Cycloolivil (*m*/*z* 421.1503, [M+HCOO]^−^, and Matairesinoside (*m*/*z* 519.1869, [M−H]^−^) were identified. For example, compound 41 shows an MS1 ion at *m*/*z* 117.0701 [M+H−H_2_O]^+^ in positive ion mode, and a fragment ion at *m*/*z* 91.0546 [M+H−H_2_O−C_2_H_2_]^+^ due to the loss of an acetylene molecule, suggesting its identification as Cinnamyl Alcohol. Compound 44 exhibits a protonated molecular ion peak at *m*/*z* 209.0807 [M+H]^+^, with fragment ions at *m*/*z* 177.0546 [M+H−OCH_3_]^+^ and *m*/*z* 145.0283 [M+H−2OCH_3_]^+^ due to the consecutive loss of methoxy groups, suggesting its identification as Ferulic acid methyl ester.

#### 2.1.5. Mass Spectrometric Fragmentation Characterization of Amino Acids and Peptides

Amino acids are the fundamental units of proteins, consisting of a central carbon atom (C) connected to an amino group (-NH_2_), a carboxyl group (-COOH), a hydrogen atom (-H), and a side chain (R). Differences in the side chain structures confer different chemical properties and biological functions to standard amino acids. A total of 16 amino acid compounds were identified, including Aceglutamide (*m*/*z* 189.0869, [M+H]^+^), gamma-Aminobutyric acid (*m*/*z* 104.0709, [M+H]^+^), L-Proline (*m*/*z* 116.0708, [M+H]^+^), Pyroglutamic acid (*m*/*z* 130.0499042, [M+H]^+^), L-Pipecolic acid (*m*/*z* 130.0863, [M+H]^+^), L-Leucine (*m*/*z* 132.1019, [M+H]^+^), L-Glutamine (*m*/*z* 147.0763, [M+H]^+^), L-Glutamic acid (*m*/*z* 148.0603, [M+H]^+^), N-Acetylvaline (*m*/*z* 160.0967, [M+H]^+^), L-Phenylalanine (*m*/*z* 166.0862, [M+H]^+^), L-Arginine (*m*/*z* 175.1189, [M+H]^+^), L-Tyrosine (*m*/*z* 180.0658, [M−H]^−^), 3-Methoxytyrosine (*m*/*z* 212.0917, [M+H]^+^), N-(1-Deoxy-1-fructosyl)leucine (*m*/*z* 292.1400, [M−H]^−^), N-(1-Deoxy-1-fructosyl)phenylalanine (*m*/*z* 328.1388, [M+H]^+^), and N-(1-Deoxy-1-fructosyl)tyrosine (*m*/*z* 344.1341, [M+H]^+^).

Peptides, formed by the linkage of two or more amino acids through peptide bonds, were also identified, with 14 compounds including gamma-Glutamylleucine (*m*/*z* 261.1443, [M+H]^+^), N-Acetylleucine (*m*/*z* 172.0971, [M−H]^−^), Glycyl-Isoleucine (*m*/*z* 189.1233, [M+H]^+^), N-Acetyl-l-glutamic acid (*m*/*z* 190.0709, [M+H]^+^), Serylvaline (*m*/*z* 205.1182, [M+H]^+^), N-Acetyl-l-phenylalanine (*m*/*z* 206.0816, [M−H]^−^), N-Acetylarginine (*m*/*z* 217.1295, [M+H]^+^), Pantothenic acid (*m*/*z* 218.1028, [M−H]^−^), Asparaginyl-Leucine (*m*/*z* 246.1447, [M+H]^+^), Aspartyl-Leucine (*m*/*z* 247.1287, [M+H]^+^), Glutamylleucine (*m*/*z* 261.1443, [M+H]^+^), Isoleucyl-Glutamate (*m*/*z* 261.1443, [M+H]^+^), Ophthalmic acid (*m*/*z* 290.1343, [M+H]^+^), and Oxidized glutathione (*m*/*z* 611.1442, [M−H]^−^).

#### 2.1.6. Mass Spectrometric Fragmentation Characterization of Nucleotides and Their Derivatives

Nucleotides are the fundamental units of nucleic acids (DNA and RNA), consisting of three main components: a pentose sugar, at least one phosphate group, and a nitrogenous base (purine or pyrimidine). A total of six such compounds were identified: Inosine (*m*/*z* 267.0732, [M−H]^−^), Adenosine (*m*/*z* 268.1038, [M+H]^+^), Guanosine (*m*/*z* 284.0987, [M+H]^+^), Thymidine (*m*/*z* 287.0881, [M+HCOO]^−^, Guanosine monophosphate (*m*/*z* 364.0649, [M+H]^+^), and Succinyladenosine (*m*/*z* 384.1146, [M+H]^+^).

For the identification of compound 84, the protonated molecular ion peak was detected at *m*/*z* 268.1038 [M+H]^+^ in the positive ion mode. A secondary fragment ion at *m*/*z* 136.0617 [M+H−C_5_H_8_O_4_]^+^ was formed by the loss of a ribose moiety from the parent ion. Based on reference standards, mass spectrometric fragmentation patterns, and literature data, this compound was identified as adenosine.

#### 2.1.7. Mass Spectrometric Fragmentation Characterization of Sugars and Glycosides

The sugar and glycoside components in *Rhodiola* primarily include simple sugars (such as glucose and fructose) and glycosides, which are formed by the combination of sugars with other compounds (such as phenylpropanoids and phenolic compounds). The fundamental framework of these sugar components consists of a cyclic carbohydrate structure, while glycosides contain a sugar moiety linked to a non-sugar moiety (aglycone) via a glycosidic bond.

A total of 20 sugar compounds were identified, including 1,4-d-Gulonolactone (*m*/*z* 177.0397, [M−H]^−^), Manosamine (*m*/*z* 180.0865, [M+H]^+^), Galactitol (*m*/*z* 181.0709, [M−H]^−^), D-altrofurano-heptulose-3 (*m*/*z* 191.0552, [M−H−H_2_O]^−^), Galacturonic acid (*m*/*z* 193.0346, [M−H]^−^), Ribonolactone (*m*/*z* 193.0347, [M+HCOO]^−^), Gluconic acid (*m*/*z* 195.0503, [M−H]^−^), N-Acetyl-d-glucosamine (*m*/*z* 204.0866, [M+H]^+^), Glucose (*m*/*z* 225.0610, [M+HCOO]^−^), 1-*β*-d-Arabinofuranosyluracil (*m*/*z* 243.0619, [M−H]^−^), Cytarabine (*m*/*z* 244.0927, [M+H]^+^), Galactose 1-phosphate (*m*/*z* 259.0221, [M−H]^−^), Trehalose (*m*/*z* 325.1127, [M+H−H_2_O]^+^), 2-O-*β*-d-Glucopyranosyl-l-ascorbic acid (*m*/*z* 337.0774, [M−H]^−^), 6-(*α*-d-Glucosaminyl)-1D-myo-inositol (*m*/*z* 342.1391, [M+H]^+^), Sucrose (*m*/*z* 365.1051, [M+H]^+^), *α*-Lactose (*m*/*z* 387.1141, [M−H]^−^), A-d-Glucopyranoside (*m*/*z* 493.2288, [M+HCOO]^−^), Maltotriose (*m*/*z* 549.1670, [M−H]^−^), and Stachyose (*m*/*z* 711.2201, [M+HCOO]^−^).

Ten glycoside compounds were identified, namely Regaloside B (*m*/*z* 487.1453, [M+HCOO]^−^), Glucovanillin (*m*/*z* 313.0929, [M−H]^−^), Arbutin (*m*/*z* 317.0875, [M−H]^−^), Phaseoloidin (*m*/*z* 329.0876, [M−H]^−^), Vanillic acid 4-*β*-d-glucopyranoside (*m*/*z* 329.0876, [M−H]^−^), 4-O-*β*-Glucopyranosyl-cis-coumaric acid (*m*/*z* 371.0979, [M−H]^−^), Coniferin (*m*/*z* 387.1292, [M+HCOO]^−^, Trans-ferulic acid-4-*β*-glucoside (*m*/*z* 401.1085, [M+HCOO]^−^), Neolinustatin (*m*/*z* 468.1719, [M−H]^−^), and Salidroside (*m*/*z* 345.1186, [M+HCOO]^−^).

The fragmentation of these compounds primarily involves the loss of sugar moieties. The cleavage of glycosidic bonds is the most crucial fragmentation pathway in the analysis of these compounds, as it facilitates the separation of the sugar and aglycone moieties, thereby aiding in structural identification and characterization. For instance, in the case of compound 91, the MS1 ion in the negative ion mode was detected at *m*/*z* 317.0875 [M+FA-H]^−^, with *m*/*z* 271.0825 [M−H]^−^ as the quasi-molecular ion peak. A secondary fragment ion at *m*/*z* 108.0206 [M−H−C_6_H_11_O_5_]^−^ was generated due to the loss of a glucose moiety from the parent ion. Based on reference standards, mass spectrometric fragmentation patterns, and literature data, this compound was identified as arbutin.

#### 2.1.8. Mass Spectrometric Fragmentation Characterization of Terpenoid Compounds

Terpenoids in *Rhodiola* represent an important class of natural organic compounds that are widely involved in plant physiological processes and significantly contribute to the medicinal value of *Rhodiola*. The fundamental skeleton of terpenoid compounds is constructed from isoprene units connected in a head-to-tail manner, forming diverse structures [16]. A total of 11 such compounds were identified, including Patchouli alcohol (*m*/*z* 205.1950, [M+H−H_2_O]^+^), *β*-Eudesmol (*m*/*z* 205.195, [M+H−H_2_O]^+^), Micheliolide (*m*/*z* 231.1379, [M+H−H_2_O]^+^), 1*β*-Hydroxyalantolactone (*m*/*z* 231.1379, [M+H−H_2_O]^+^), Atractylenolide III (*m*/*z* 249.1483, [M+H]^+^), Diacetoxyscirpenol (*m*/*z* 367.1753, [M+H]^+^), Geraniol (*m*/*z* 137.1324, [M+H−H_2_O]^+^), Rosiridin (*m*/*z* 350.2170, [M+NH_4_]^+^), Secologanic acid (*m*/*z* 355.1032, [M−H−H_2_O]^−^), Crenulatin (*m*/*z* 247.1183, [M−H]^−^), and Dihydroactinidiolide (*m*/*z* 181.1222, [M+H]^+^).

For example, Compound 126 exhibited a protonated molecular ion peak at *m*/*z* 350.2170 [M+NH_4_]^+^ in positive ion mode. The secondary fragment ion at *m*/*z* 153.1273 [M+H−glc]^+^ resulted from the loss of one glucose molecule (C_6_H_12_O_6_) from the parent ion, followed by the loss of one water molecule, generating a fragment ion at *m*/*z* 135.1169 [M+H−glc−H_2_O]^+^. Subsequently, consecutive methyl group losses led to fragment ions at *m*/*z* 107.0858 [M+H−glc−H_2_O−2CH_3_]^+^ and *m*/*z* 93.0703 [M+H−glc−H_2_O−3CH3]^+^. Based on reference standards, mass spectrometric fragmentation patterns, and literature data, this compound was identified as Rosiridin. Similarly, Compound 128, in positive ion mode, exhibited a protonated molecular ion peak at *m*/*z* 271.1149 [M+Na]^+^. The secondary fragment ion at *m*/*z* 201.0368 [M+Na−C_5_H_10_]^+^ was formed through the cleavage of a glucose unit with sodium attachment. Combining reference standards, fragmentation patterns, and literature data, this compound was identified as Rhodioloside.

#### 2.1.9. Mass Spectrometric Fragmentation Characterization of Organic Acids and Their Derivatives

The basic framework of organic acids consists of a carbon chain and at least one carboxyl group. The properties of organic acids are influenced by the length and saturation of the carbon chain as well as the position of the carboxyl group (either terminal or within the chain). Derivatives of organic acids are formed through reactions between the carboxyl group and other chemical groups, such as esters (formed with alcohols) and amides (formed with amines). In *Rhodiola*, the mass spectrometric analysis of organic acids and their derivatives primarily involves cleavage of the carboxyl group, side-chain fragmentation, dehydration reactions, and the elimination of specific functional groups. A total of 11 compounds were identified, including Caffeic acid (*m*/*z* 181.0494, [M+H]^+^), Ferulic acid (*m*/*z* 193.0499, [M−H]^−^), p-Coumaric acid (*m*/*z* 165.0545, [M+H]^+^), Quinic acid (*m*/*z* 175.0600, [M+H−H_2_O]^+^), Ascorbic acid (*m*/*z* 177.0393, [M+H]^+^), Citric acid (*m*/*z* 191.0189, [M−H]^−^), 2-Methylcitric acid (*m*/*z* 205.0347, [M−H]^−^), Xanthurenic acid (*m*/*z* 206.0447, [M+H]^+^), Isocitric acid (*m*/*z* 210.06078, [M+NH_4_]^+^), 3-Hydroxysebacic acid (*m*/*z* 217.1076, [M−H]^−^), and Abscisic acid (*m*/*z* 247.1327, [M+H−H_2_O]^+^).

Carboxyl group cleavage often results in the loss of the carboxyl moiety, generating protonated or deprotonated fragment ions. For example, Compound 134 exhibited a protonated molecular ion peak at *m*/*z* 177.0393 [M+H]^+^ in positive ion mode. The secondary fragment ion at *m*/*z* 149.0230 [M+H−CO]^+^ resulted from the neutral loss of one CO molecule (28 Da) from the parent ion. Further fragmentation led to the loss of two water molecules, generating fragment ions at *m*/*z* 141.0182 [M+H−2H_2_O]^+^, *m*/*z* 113.0235 [M+H−2H_2_O−CO]^+^, and *m*/*z* 95.0131 [M+H−3H_2_O−CO]^+^. Based on reference standards, fragmentation rules, and literature data, this compound was identified as Ascorbic acid.

#### 2.1.10. Mass Spectrometric Fragmentation Characterization of Fatty Acyl Compounds

Fatty acyl compounds in *Rhodiola* mainly refer to various fatty acids and their derivatives, including saturated and unsaturated fatty acids. These compounds have a long-chain carbon backbone terminated by a carboxyl (-COOH) group. Fatty acid derivatives can be formed through esterification of the carboxyl group with molecules like glycerol or by introducing other functional groups such as hydroxyl or double bonds into the carbon chain. A total of 13 such compounds were identified, including Decenoic acid (*m*/*z* 153.1273, [M+H−H_2_O]^+^), Suberic acid (*m*/*z* 173.0811, [M−H]^−^), Sebacic acid (*m*/*z* 185.1174, [M+H−H_2_O]^+^), Azelaic acid (*m*/*z* 187.0968, [M−H]^−^), Undecanedioic acid (*m*/*z* 217.1434, [M+H−H_2_O]^+^), Dodecanedioic acid (*m*/*z* 229.1441, [M−H]^−^), Octadecatrienoic acid (*m*/*z* 279.2316, [M+H]^+^), Oleamide (*m*/*z* 282.2789, [M+H]^+^), Hexadecanedioic acid (*m*/*z* 285.2069, [M−H]^−^), Octadecanedioic acid (*m*/*z* 313.2382, [M−H]^−^), 12,13-DHOME (*m*/*z* 313.2383, [M−H]^−^), Linoleoyl ethanolamide (*m*/*z* 324.2895, [M+H]^+^), and Docosenamide (*m*/*z* 338.3413, [M+H]^+^).

For example, Compound 148 exhibited a protonated molecular ion peak at *m*/*z* 282.2789 [M+H]^+^ in positive ion mode. The secondary fragment ion at *m*/*z* 265.2520 [M+H−NH_3_]^+^ resulted from the loss of one NH3 molecule (17 Da) from the parent ion, followed by the loss of an oxygen atom, generating *m*/*z* 247.2418. Based on reference standards, mass spectrometric fragmentation rules, and literature data, this compound was identified as Oleamide. Similarly, Compound 142, in negative ion mode, exhibited a deprotonated molecular ion peak at *m*/*z* 173.0811 [M−H]^−^. The secondary fragment ion at *m*/*z* 129.0910 [M−H−CO_2_]^−^ resulted from the loss of one CO_2_ molecule (44 Da), followed by the loss of one water molecule (18 Da), generating a fragment ion at *m*/*z* 111.0805. Based on reference standards, fragmentation patterns, and literature data, this compound was identified as Suberic acid.

#### 2.1.11. Mass Spectrometric Fragmentation Characterization of Alkaloids

Alkaloids, as a class of nitrogen-containing natural basic compounds, typically have nitrogen atoms in their molecular structures that carry a positive charge, making them prone to forming protonated molecular ion peaks under positive ion mode, thereby exhibiting high responsiveness. One characteristic structural feature of this compound group is the presence of a pyridine ring in the C-ring, which undergoes the Retro-Diels–Alder (RDA) fragmentation mechanism. Accordingly, their mass spectrometric characteristics are mainly reflected in fragmentation reactions of substituents, particularly the formation of fragment ions such as demethylation and CO loss. One compound was identified as Hygric acid (*m*/*z* 130.0863, [M+H]^+^).

#### 2.1.12. Carboxylic Acids and Derivatives

Carboxylic acids and their derivatives in *Rhodiola* species form a crucial part of the plant’s chemical composition, exhibiting diverse biological activities. The fundamental skeleton of these compounds consists of one or more carboxyl (-COOH) groups, and their derivatives exist in various forms, such as esters and amides. This structural diversity endows them with extensive biological functions. In this study, one compound of this category was identified: Camphoric acid (*m*/*z* 199.0969, [M−H]^−^).

#### 2.1.13. Other Compounds

Other identified compounds include Steroids (1 compound), Organoheterocyclic Compounds (3 compounds), Indoles and Derivatives (4 compounds), Sphingolipids (2 compounds), and Quinones (1 compound), totaling 11 compounds. Although these compounds are present in *Rhodiola* species at low levels and have been less studied, they may contribute to the plant’s pharmacological activities, warranting further investigation.

#### 2.1.14. Principal Component Analysis (PCA)

Each data point in the multivariate analysis represents a pooled composite sample rather than an individual biological replicate. Accordingly, multivariate analyses were applied to visualize overall species-level metabolic patterns rather than for statistical inference.

**Figure 3:**
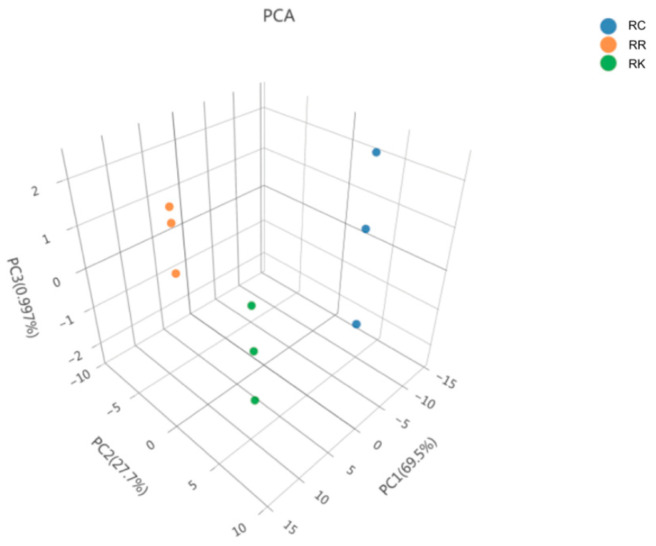
PCA-3D Plot of Three *Rhodiola* Species. Note: Each point represents a pooled composite sample generated from multiple batches, with different color and shape combinations indicating different sample groups.

PCA was applied as an exploratory multivariate approach to visualize overall metabolic trends among the three *Rhodiola* species based on pooled composite samples. Accordingly, this analysis was used to compare species-level metabolic patterns rather than to support formal statistical inference, as shown in Figure 3. The PCA score scatter plot shows that samples of *R. crenulata*, *R. kirilowii*, and *R. rosea* tend to cluster in distinct regions. The high R^2^X value (0.985) indicates that the principal components capture a large proportion of the variance in the dataset.

#### 2.1.15. Partial Least Squares Discriminant Analysis (PLS-DA)

PLS-DA was subsequently performed using the same pooled samples to further examine potential discriminatory trends among species. Given the limited number of composite samples, the PLS-DA results were interpreted in an exploratory manner. PLS-DA, a supervised multivariate analysis method, was subsequently applied using the same pooled composite samples to further explore potential discriminatory trends among the three *Rhodiola* species. Unlike PCA, which is an unsupervised approach, PLS-DA incorporates sample class information to emphasize variation related to predefined groups. Therefore, PLS-DA was used here as a complementary exploratory tool to assist in visualizing potential separation trends observed in the PCA results.

**Figure 4:**
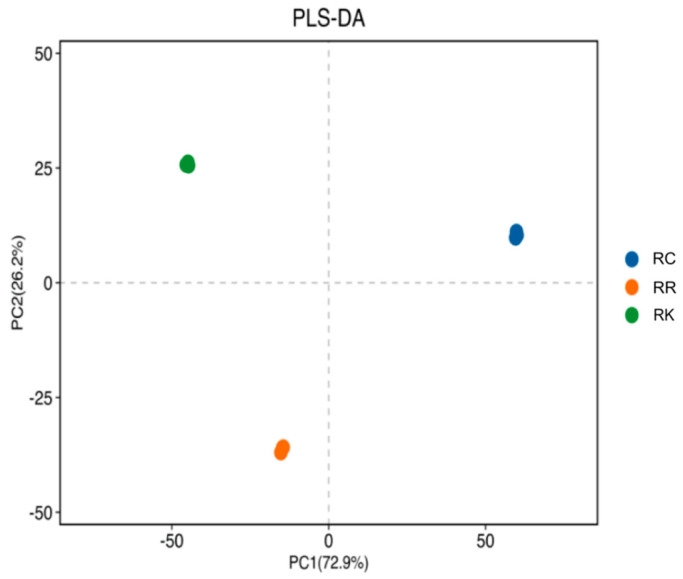
PLS-DA Plot of Three *Rhodiola* Species. Note: Each point represents a pooled composite sample generated from multiple batches.

The performance of the PLS-DA model was evaluated using R^2^X (cum), R^2^Y (cum), and Q^2^ (cum) values, which describe the explained variance in independent variables, dependent variables, and the model’s predictive capability, respectively. The model yielded R^2^X (cum) = 0.998, R^2^Y (cum) = 1, and Q^2^ (cum) = 1; given the pooled nature and limited number of composite samples, these parameters were used for descriptive evaluation rather than to assess predictive accuracy or statistical significance. The PLS-DA score plot (Figure 4) shows a separation trend among the three species that is generally consistent with the PCA results.

#### 2.1.16. Differential Component Analysis

**Figure 5:**
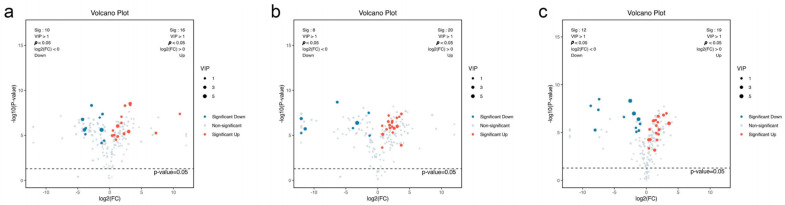
Volcano Plots of Differential Components Among Three *Rhodiola* Species. Note: Each point represents a metabolite. The *x*-axis represents the log_2_(FC) value of the two-group comparison, while the *y*-axis represents the −log10(*p*-value). (**a**): *R. rosea* vs. *R. crenulata*; (**b**): *R. kirilowii* vs. *R. crenulata*; (**c**): *R. kirilowii* vs. *R. rosea*.

To visualize the results of the differential component analysis, volcano plots were generated using data from *p*-values, VIP values, and fold change (FC) values. Components meeting the criteria of *p* < 0.05, VIP > 1, and FC > 1 were classified as upregulated differential components, while those with *p* < 0.05, VIP > 1, and FC < 1 were classified as downregulated differential components. In the volcano plot, upregulated components are clustered in red, while downregulated components are clustered in blue. The volcano plots for pairwise comparisons among the three *Rhodiola* species are shown in Figure 5.

By conducting both overall and pairwise comparisons among the three *Rhodiola* species, a total of 30 differential components were identified. The comparison results are summarized in Table 1.

In the differential component comparison between *R. rosea* and *R. crenulata*, the upregulated components were predominantly amino acids and peptides, while the downregulated components were mostly flavonoids. This indicates that *R. rosea* contains relatively higher levels of amino acids and peptides but lower levels of flavonoids. Similarly, the comparison between *R. kirilowii* and *R. crenulata* revealed that *R. kirilowii* has relatively higher amino acid and peptide content but lower flavonoid content. The comparison between *R. kirilowii* and *R. rosea* also showed that *R. kirilowii* has relatively higher levels of amino acids and peptides but lower flavonoid content.

#### 2.1.17. Analysis of the Top 30 Differential Metabolites

**Figure 6:**
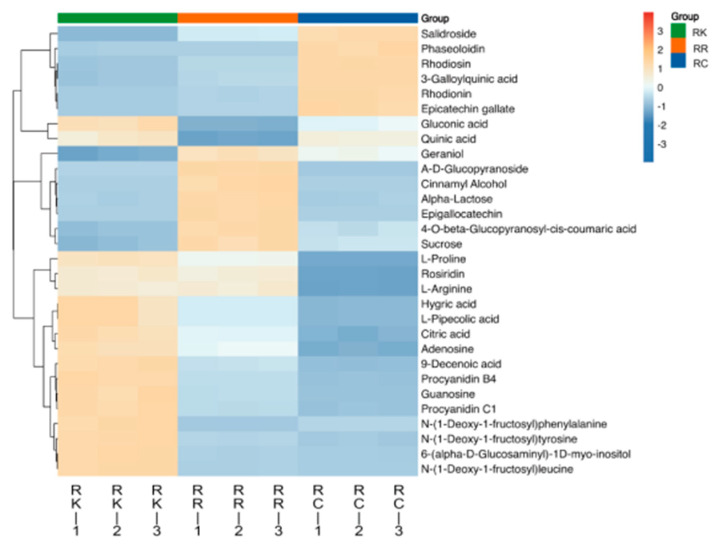
Clustering Heatmap. Note: Rows represent individual metabolites, and columns represent pooled composite samples from each species. Colors indicate relative metabolite abundance after normalization, with hierarchical clustering applied to highlight similarity patterns among species and metabolites.

To further characterize the abundance patterns of the 30 selected differential metabolites, a heatmap analysis was performed (Figure 6). The heatmap illustrates their relative abundance across the three *Rhodiola* species, revealing distinct species-specific distribution patterns. Among these metabolites, salidroside, borneol 7-O-[*β*-d-apiofuranosyl-(1→6)]-*β*-d-glucopyranoside, and *α*-lactose exhibited pronounced differences in relative abundance among species.

Specifically, *R. crenulata* showed higher relative abundance of several phenolic and flavonoid-related metabolites, including salidroside and rhodiosin. In contrast, *R. rosea* was characterized by higher levels of carbohydrate- and volatile-related metabolites, such as *α*-lactose and geraniol. *R. kirilowii* exhibited increased abundance of amino acid- and nucleoside-associated metabolites, including L-proline and adenosine. Overall, this heatmap provides a clear visualization of the species-specific distribution patterns of the identified differential metabolites.

### 2.2. Investigation of Anti-Hypoxia Active Components in Three Rhodiola Species

#### 2.2.1. Study on the Anti-Hypoxia Intervention Effects of Three Rhodiola Species

During the experiment, as oxygen levels gradually decreased in the sealed environment over time, mice exhibited typical physiological responses to hypoxia. These included an increased respiratory rate and limb convulsions in the early stage, progressing to auricular cyanosis, a change in eye color from red to purple, urinary incontinence, and ultimately respiratory failure leading to death. In this study, the duration from sealing the bottle to the cessation of respiration was defined as the hypoxia survival time of the mice, which was used to evaluate their hypoxia tolerance.

The average survival time for the blank control group (BC) was 39.16 min, while the positive control group (PC) survived for an average of 44.34 min. The *R. crenulata* group (RC) had an average survival time of 47.07 min, the *R. kirilowii* group (RK) averaged 44.82 min, and the *R. rosea* group (RR) survived for 48.16 min. Compared to the blank control group, the survival time increased by 13.23%, 20.20%, 14.45%, and 22.98%, respectively. The detailed results of the hypoxia survival time for each group are presented in Table 2.

#### 2.2.2. PLSR Analysis Results of the Anti-Hypoxia Effects of Three Rhodiola Species

The relative content percentages of chemical constituents in the three *Rhodiola* species were calculated. Constituents with a content greater than 0.5% were selected, and a total of 45 compounds were identified after taking the union (Appendix A). These target compounds were then subjected to correlation analysis with pharmacodynamic results.

**Figure 7:**
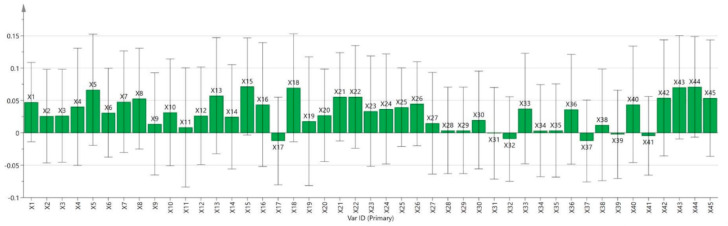
Regression coefficient plot of the PLS model for anti-hypoxia effects. Note: Data represent pooled composite samples analyzed in triplicate.

In the PLSR analysis, rat survival time was used as the dependent variable, and the regression coefficient plot is shown in Figure 7. In this analysis, the absolute value of the regression coefficient of an independent variable directly reflects the strength of its influence on the dependent variable. Specifically, if the regression coefficient of a chemical constituent is greater than zero, it indicates that the constituent contributes positively to the anti-hypoxia activity of *Rhodiola*, and the larger the coefficient, the more significant its role. Among the target compounds, 39 showed a positive correlation with anti-hypoxia effects.

**Figure 8:**
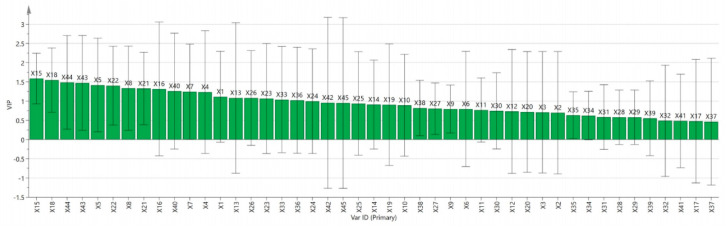
VIP values in the PLS model for anti-hypoxia effects. Data represent pooled composite samples analyzed in triplicate.

The VIP value reflects the explanatory power of an independent variable on the dependent variable. Generally, variables with VIP > 1 are considered to be specific and important in this study (Figure 8). The analysis revealed that in the three *Rhodiola* species, the following compounds had VIP values greater than 1: X15, X18, X44, X43, X5, X22, X8, X21, X16, X40, X7, X4, X1, X13, X26, X23, X33, and X36.

The relatively large standard deviations likely reflect biological heterogeneity among pooled batches rather than analytical variability.

#### 2.2.3. Grey Relational Analysis

**Figure 9:**
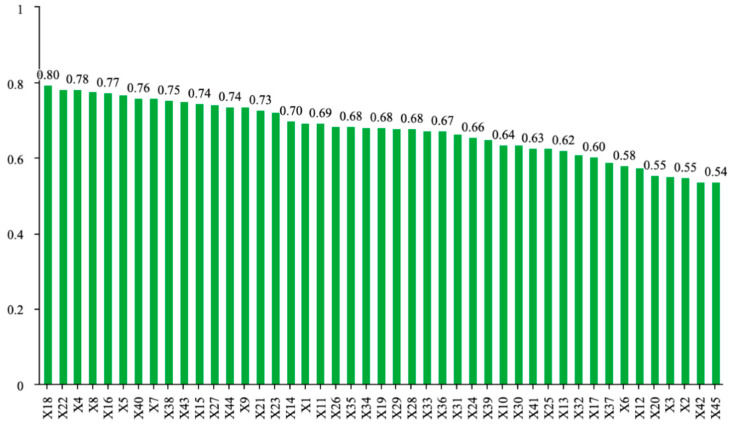
Grey relational analysis results.

The correlation between chromatographic peaks and pharmacodynamic effects was evaluated based on the grey relational grade, where a higher coefficient value indicates a stronger correlation. Generally, when the correlation grade exceeds 0.6, the compound is considered to be associated with the pharmacodynamic indicator. As shown in Figure 9, a total of 36 compounds were identified as relevant to the anti-hypoxia effect, highlighting the synergistic action of multiple components in traditional Chinese medicine to exert therapeutic effects.

The GRA method mainly focuses on the similarity of data trend changes rather than the specific nature of directional changes. Therefore, it cannot directly determine whether a particular compound has a positive or negative effect on pharmacodynamics, and its results must be interpreted in conjunction with the PLSR model analysis.

### 2.3. Verification of Anti-Hypoxia Active Compounds via Molecular Docking

Molecular docking technology was employed to simulate the interaction between six functionally targeted compounds—Geraniol, 4-O-*β*-Glucopyranosyl-cis-coumaric acid, (−)-Epicatechin, 3-Galloylquinic acid, Citric acid, and Salidroside—and HIF-1*α*, to validate the findings of the spectrum-effect analysis. Under the current docking parameters, the binding free energy of the best-docked conformations of the ligand-receptor complexes is shown in Table 3, where a lower binding free energy indicates a higher affinity between the ligand and receptor.

**Figure 10:**
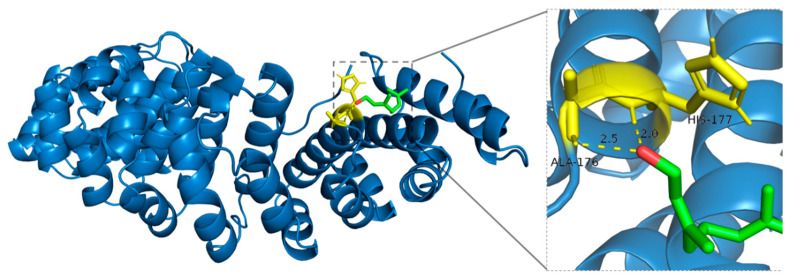
Binding mode of Geraniol with HIF-1*α*.

Geraniol forms stable hydrogen bonds with specific residues of HIF-1*α*, attributed to its dual functionality as both a hydrogen bond donor and acceptor. Although Alanine (ALA-176), due to its simple structure, does not directly participate in hydrogen bond formation via its methyl side chain, its backbone amino and carboxyl groups can still interact with the hydroxyl group of Geraniol. Additionally, the nitrogen atom in the imidazole side chain of Histidine (HIS-177) is directly involved in hydrogen bonding, further stabilizing the Geraniol-HIF-1*α* complex, which may influence protein conformation and biological function (Figure 10).

**Figure 11:**
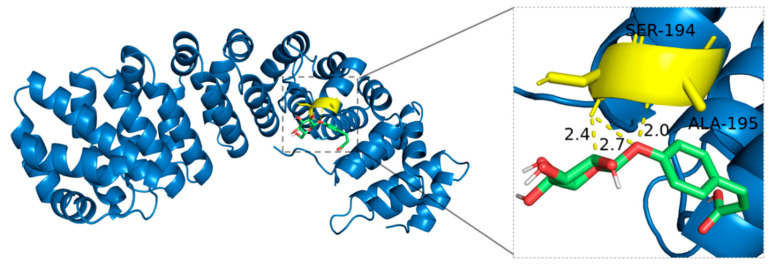
Binding mode of 4-O-*β*-Glucopyranosyl-cis-coumaric acid with HIF-1*α*.

4-O-*β*-Glucopyranosyl-cis-coumaric acid interacts with the side chains of SER-194 and ALA-195 in HIF-1*α*, forming hydrogen bonds. The structural characteristics of 4-O-*β*-Glucopyranosyl-cis-coumaric acid, particularly its carboxyl and hydroxyl groups in the coumaric acid moiety and multiple hydroxyl groups in the glucose unit, provide multiple potential sites for hydrogen bonding. Notably, the hydroxyl group at the polar terminus of SER-194 enables it to act as both a hydrogen bond donor and acceptor, directly forming hydrogen bonds with 4-O-*β*-Glucopyranosyl-cis-coumaric acid. Although ALA-195 is nonpolar and its methyl side chain does not engage in hydrogen bonding, indirect interactions involving the protein backbone atoms may promote hydrogen bond formation, demonstrating the intricate spatial complementarity and dynamic intermolecular interactions (Figure 11).

**Figure 12:**
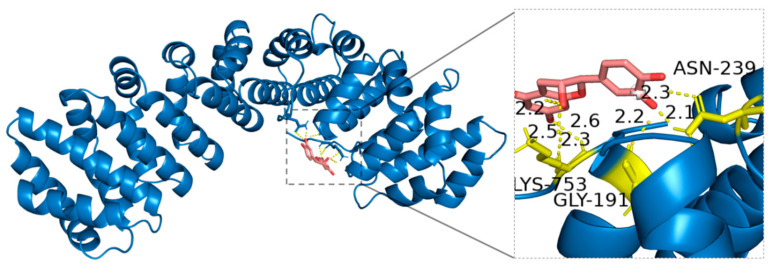
Binding mode of (−)-Epicatechin with HIF-1*α*.

Molecular docking analysis revealed that (−)-Epicatechin forms hydrogen bonds with specific amino acid residues of HIF-1*α*, including Asparagine (ASN-239), Lysine (LYS-753), and Glycine (GLY-191). The amide side chain of ASN-239 has a polar group that can act as both a hydrogen bond donor and acceptor, participating in precise intermolecular interactions. The positively charged amine terminal of LYS-753 enables it to function as a strong hydrogen bond donor and enhances interactions with negatively charged ligands, such as the hydroxyl groups in (−)-Epicatechin. Although GLY-191 lacks a side chain, its backbone amino and carboxyl groups can still participate in hydrogen bonding. These interactions highlight a high degree of spatial complementarity and molecular recognition, stabilizing the (−)-Epicatechin-HIF-1*α* complex (Figure 12).

**Figure 13:**
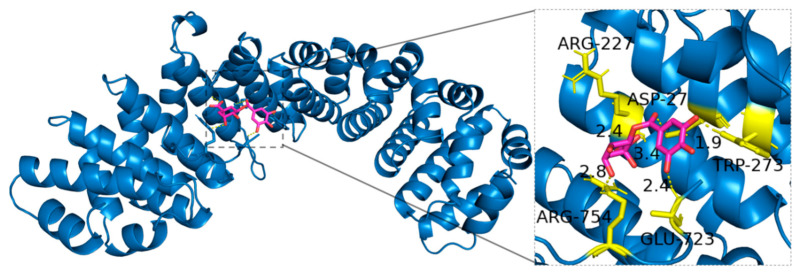
Binding mode of 3-Galloylquinic acid with HIF-1*α*.

This study found that 3-Galloylquinic acid forms hydrogen bonds with Arginine (ARG-227, ARG-754), Aspartic acid (ASP-27), Glutamic acid (GLU-723), and Tryptophan (TRP-273) in HIF-1*α*. Specifically, the guanidine group at the terminus of ARG-227 and ARG-754 serves as a hydrogen bond donor, facilitating interaction with 3-Galloylquinic acid. The negatively charged carboxyl groups of ASP-27 and GLU-723 provide acceptor sites for hydrogen bonding, while the indole nitrogen in the TRP-273 side chain further enhances the molecular complementarity and stability of interactions (Figure 13).

**Figure 14:**
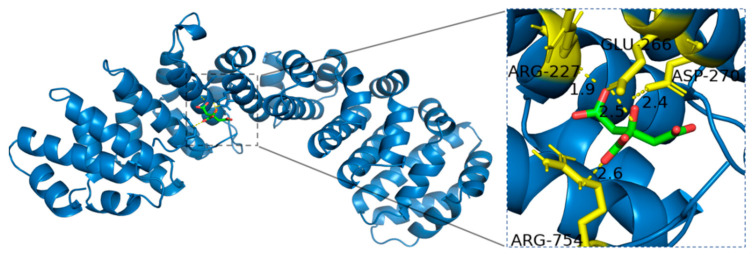
Binding mode of Citric acid with HIF-1*α*.

Citric acid, possessing both carboxyl and hydroxyl functional groups, was found to form hydrogen bonds with specific residues of HIF-1*α*, including Arginine (ARG-227, ARG-754), Aspartic acid (ASP-270), and Glutamic acid (GLU-266). The positively charged guanidine groups of ARG-227 and ARG-754 act as strong hydrogen bond donors, facilitating interactions with the carboxyl and hydroxyl groups of Citric acid. Additionally, the negatively charged carboxyl groups of ASP-270 and GLU-266 serve as hydrogen bond acceptors, further stabilizing the Citric acid-HIF-1*α* complex (Figure 14).

**Figure 15:**
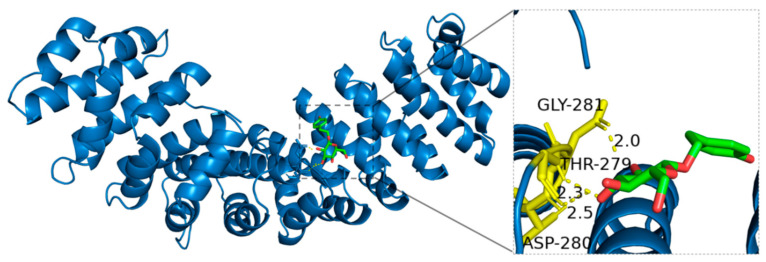
Binding mode of Salidroside with HIF-1*α*.

Detailed molecular docking analysis revealed that Salidroside interacts with HIF-1*α* through hydrogen bonds formed between its hydroxyl functional groups and specific amino acid residues. Glycine (GLY-281), due to its simple structure and lack of a side chain, relies on its backbone amino and carboxyl groups to form key hydrogen bonds with Salidroside. The hydroxyl-containing side chain of Threonine (THR-279) allows it to act flexibly as a hydrogen bond donor or acceptor, enhancing the molecular interaction. Moreover, the negatively charged carboxyl group of Aspartic acid (ASP-280) forms strong hydrogen bonds with the hydroxyl groups of Salidroside, significantly increasing its binding affinity with HIF-1*α* (Figure 15).

## 3. Discussion

This study systematically compared the chemical profiles and anti-hypoxia activities of *R. crenulata*, *R. kirilowii*, and *R. rosea* using an integrative approach integrating UPLC-QE-MS-based metabolite identification, in vivo pharmacological evaluation with herbal powder solutions, chemometric correlation analyses, and molecular docking. A total of 161 shared metabolites were identified across the three species, demonstrating a highly similar phytochemical foundation, while 30 differential metabolites revealed species-specific features. These compositional characteristics provide important chemical context for interpreting the broadly comparable anti-hypoxia effects observed among the three species.

In the animal experiments, a normobaric hypoxia model was used to evaluate the anti-hypoxia activity of the three *Rhodiola* species, with salidroside as the positive control. This study aimed to explore their potential in enhancing hypoxia tolerance in mice. The experiment used a rapid normobaric hypoxia induction protocol that is straightforward to implement under laboratory conditions [17]. Salidroside, a key active compound in *Rhodiola*, was chosen as the positive drug due to its well-documented efficacy in mitigating hypoxia. This is mainly attributed to its excellent antioxidant and free radical scavenging capabilities, which help neutralize the excessive free radicals produced under hypoxic conditions and alleviate oxidative stress, thereby providing cellular protection [18]. Salidroside has been extensively studied and applied in the development of anti-altitude sickness drugs [19]. Regarding dosage selection, based on the clinical dosage of *Rhodiola* documented in the Chinese Pharmacopoeia (3–6 g), a dose of 6 g was chosen to ensure maximum efficacy within a safe range, with the equivalent dosage for mice calculated based on the body surface area conversion ratio.

All three *Rhodiola* species significantly prolonged survival time in a normobaric hypoxia mouse model, indicating that the anti-hypoxia effects are likely mediated through the coordinated actions of multiple metabolites rather than a single compound. This multi-component pharmacological feature is characteristic of botanical medicines and highlights the value of chemometric approaches for identifying metabolites most strongly associated with in vivo efficacy. Among the three *Rhodiola* species, the RR group exhibited the longest survival time, surpassing both RC group and RK group. This finding not only confirms the anti-hypoxia effects of *Rhodiola* but also suggests possible differences in bioactivity among different species in enhancing hypoxia tolerance. It underscores the necessity of further exploring the bioactive compounds responsible for *Rhodiola*’s anti-hypoxia effects and the importance of understanding the differences in bioactive components among species.

From a functional classification perspective, the metabolites identified in the three *Rhodiola* species mainly belonged to flavonoids, phenylpropanoids, amino acid–related metabolites, carbohydrates and glycosides, terpenes, and organic acids. Flavonoids and phenylpropanoids are widely recognized for their roles in modulating oxidative stress and inflammatory responses, which are central pathological features of hypoxia-induced injury. Amino acids and small peptides contribute to cellular stress adaptation by supporting redox balance, nitrogen metabolism, and protein turnover under adverse conditions. Carbohydrates and glycosides primarily reflect energy storage and mobilization processes, which are essential for maintaining metabolic homeostasis when oxygen availability is limited. Terpenes and organic acids, although structurally diverse, have been implicated in membrane stabilization, mitochondrial function, and metabolic regulation. Collectively, the coexistence of these metabolite classes suggests that the anti-hypoxia effects of *Rhodiola* species arise from coordinated, multi-pathway metabolic support rather than from a single dominant compound.

The identification of bioactive constituents is a central component of TCM modernization, as it links the chemical variability in herbal materials with their pharmacological effects. In this context, chemometric tools are particularly valuable for dissecting the complex chemical–activity relationships inherent in multi-component systems such as *Rhodiola* [20] Because different analytical models possess complementary strengths, combining grey relational analysis (GRA) with partial least squares regression (PLSR) improves the robustness of compound prioritization. In this study, high-abundance metabolites were selected for correlation analysis, and the integrated GRA–PLSR workflow consistently highlighted 14 components positively associated with anti-hypoxia activity. Although primary metabolites such as sugars and amino acids may play roles in energy provision and redox buffering under hypoxic stress, they are central metabolic intermediates involved in basal physiological processes and are highly conserved across species, reflecting generalized metabolic adjustments rather than specific regulatory mechanisms [21]. Central carbon and amino acid metabolism are modulated by low oxygen conditions via HIF-dependent pathways, but these pathways are shared broadly across taxa and the corresponding metabolites are ubiquitous, limiting their utility in explaining species-specific pharmacological differences [22]. In contrast, plant secondary metabolites are specialized organic compounds that are not directly required for growth or reproduction but often mediate interactions with the environment and stress responses. Secondary metabolites such as flavonoids, phenylpropanoids, terpenoids, and related classes accumulate in response to biotic and abiotic stress and exhibit substantial interspecific variability due to differences in biosynthetic gene regulation [23]. Because their biosynthesis is tightly regulated and species-dependent, these metabolites are more likely to contribute mechanistically to differential bioactivity and to show stronger statistical correlation with biological outcomes in chemometric analyses [24].

Given the central regulatory role of HIF-1*α* in hypoxia adaptation, molecular docking was employed as a hypothesis-generating assessment to explore whether prioritized metabolites could form plausible interactions with this key protein [25]. It should be noted that molecular docking scores do not represent absolute binding affinities and cannot be interpreted using universal cut-off values. Instead, docking outcomes are inherently dependent on the scoring function and docking protocol employed. In the present study, molecular docking was therefore applied under identical computational settings for all selected metabolites, and the resulting scores were interpreted in a comparative manner. Compounds exhibiting relatively lower binding energies together with stable and well-defined interaction patterns were considered to possess higher binding propensity toward HIF-1*α*. Importantly, in this work molecular docking was not used as a standalone predictive tool, but rather as a structure-based validation step to support the mechanistic plausibility of metabolite–target interactions suggested by the integrated chemometric and biological analyses.

Epicatechin, a representative flavanol, possesses established antioxidant and anti-inflammatory activities and showed stable binding interactions with HIF-1*α*, suggesting a potential role in alleviating oxidative and inflammatory injury under hypoxic stress [26]. 3-O-Galloylquinic acid, characterized by its galloyl and quinic acid moieties, has documented redox-regulatory potential and formed multiple hydrogen-bond interactions with HIF-1*α*, indicating its possible involvement in cellular stress adaptation [27]. Salidroside, a hallmark glycoside of *Rhodiola* species, is widely recognized for its cytoprotective and metabolic regulatory effects; its positive correlation with survival time and stable docking interactions further support its potential contribution to anti-hypoxia activity.

Additionally, p-coumaric acid-4-O-glucoside, citric acid, and geraniol also demonstrated docking stability and possess reported antioxidative or cytoprotective functions. p-Coumaric acid-4-O-glucoside, as a phenylpropanoid glycoside, may mitigate hypoxia-induced oxidative injury through modulation of cellular redox balance. Citric acid, a central intermediate in the tricarboxylic acid cycle, may enhance metabolic resilience under hypoxic conditions by supporting cellular energy homeostasis. Geraniol, a monoterpenoid with known antioxidant and anti-inflammatory properties, may contribute to hypoxia tolerance by reducing inflammatory stress and stabilizing redox equilibrium. These complementary features suggest that multiple structurally diverse metabolites may jointly underlie the integrated anti-hypoxia effects of *Rhodiola* species.

Notably, citric acid, as a central intermediate of the tricarboxylic acid (TCA) cycle, is not a direct regulator of HIF-1*α* activity. However, alterations in TCA cycle flux and mitochondrial metabolism under hypoxic conditions have been shown to indirectly influence HIF-1*α* stability by modulating cellular redox status and the activity of prolyl hydroxylase enzymes. Therefore, the association of citric acid with anti-hypoxia effects in this study is interpreted as reflecting metabolic adaptation linked to hypoxia signaling, rather than a direct molecular interaction with HIF-1*α*.

Taken together, the integration of metabolite profiling, pharmacological evaluation, chemometric correlation, and molecular docking forms a coherent evidence framework supporting the involvement of multiple bioactive metabolites in the anti-hypoxia effects of *Rhodiola* species. The overall similarity in chemical composition and pharmacological activity among *R. crenulata*, *R. kirilowii*, and *R. rosea* further suggests that the latter two species hold promise as complementary or alternative resources for *R. crenulata*, particularly in the context of increasing pressure on wild populations.

Although this study utilized herbal powder solutions rather than purified single compounds, which limits definitive attribution of effects to individual metabolites, and molecular docking represents an inferential approach requiring further biochemical validation, the integrative strategy effectively identified biologically plausible candidates and established testable hypotheses for future mechanistic studies. Future research should focus on the targeted isolation and structural confirmation of prioritized metabolites, followed by in vitro and in vivo mechanistic studies to determine their direct effects on HIF-1*α* stability and downstream signaling. In addition, dose–response relationships, pharmacokinetics, and potential synergistic interactions among co-occurring constituents should be evaluated to advance the translational potential of selected *Rhodiola* compounds.

## 4. Materials and Methods

### 4.1. Analysis of Chemical Components of Three Rhodiola Species Based on UPLC-QE-MS

#### 4.1.1. Instruments and Materials

TGL-16MS tabletop high-speed freezing centrifuge (Shanghai Luxiang Instrument Co., Ltd., Shanghai, China); TYXH-I vortex shaker (Shanghai Hanno Instrument Co., Ltd., Shanghai, China); ACQUITY UPLC I-Class HF high-performance liquid chromatograph (Waters Corporation, Milford, MA, USA); F-060SD ultrasonic cleaner (Shenzhen Fuyang Technology Group Co., Ltd., Shenzhen, China); ACQUITY UPLC PDA detector (Waters Corporation, Milford, MA, USA); Q Exactive™ Orbitrap high-resolution mass spectrometer (Thermo Fisher Scientific, Bremen, Germany); ACQUITY UPLC HSS T3 chromatographic column (100 mm × 2.1 mm, 1.8 μm; Waters Corporation, Milford, MA, USA). A total of 30 samples of *Rhodiola* were collected, including 10 samples each of *R. crenulata*, *R. kirilowii*, and *R. rosea*. All plant samples were authenticated by Professor Zhai Huaqiang (Beijing University of Chinese Medicine). Voucher specimens (Nos. RC-2022-01–RC-2022-10 for *R. crenulata*, RK-2023-01–RK-2023-10 for *R. kirilowii*, and RR-2023-01–RR-2023-10 for *R. rosea*) were deposited at the Herbarium of Beijing University of Chinese Medicine. Acetonitrile (Batch No. A998-4, Thermo Fisher Scientific); Formic acid (Batch No. A117-50, Thermo Fisher Scientific); Methanol (Batch No. A452-4, Thermo Fisher Scientific).

#### 4.1.2. Preparation of Sample Solutions

A total of ten independent batches were collected for each *Rhodiola* species. First, the *Rhodiola* samples were placed in liquid nitrogen, frozen, and then ground into powder. To reduce individual batch variability and considering the high cost of UPLC-QE-MS analysis, samples from the same species were equally pooled and homogenized to generate three representative composite samples for metabolomic profiling.

About 100 mg of the powder was placed in a 1.5 mL centrifuge tube. Then, 1 mL of water containing a mixed internal standard (concentration of 4 μg/mL) was added. The sample was vortexed for 1 min to mix, and steel beads were added. The sample was pre-cooled at −40 °C for 2 min to reduce the initial temperature, followed by grinding for 2 min in a 60 Hz grinder to increase surface area and improve extraction efficiency. The sample was then subjected to ultrasonic-assisted extraction in an ice-water bath for 60 min at 4 °C. Afterward, the sample was left standing at −40 °C for 30 min to further enhance the stability of the extract. The sample was then centrifuged at 12,000× *g* rpm for 10 min at 4 °C to separate the supernatant. The supernatant was diluted 3 times with water containing 4 μg/mL of the internal standard. Finally, 200 μL of the diluted supernatant was placed in a sample vial with an insert for analysis.

#### 4.1.3. Chromatographic Conditions

The chromatographic column used was ACQUITY UPLC HSS T3 (100 mm × 2.1 mm, 1.8 μm); column temperature was set to 45 °C. A Waters C18 column (2.1 × 50 mm, 1.7 μm) was also used. The mobile phase consisted of acetonitrile (A) and 0.1% formic acid water (B), with the following gradient: 95–70% A (0–4 min), 70–50% A (4–8 min), 50–20% A (8–10 min), 20–0% A (10–15 min), 0–95% A (15–16 min). The flow rate was 0.35 mL/min and the injection volume was 5 μL.

#### 4.1.4. Mass Spectrometry Conditions

An electrospray ionization (HESI) source was used to acquire ion fragmentation information in both positive and negative scan modes. Data-dependent acquisition (DDA) mode was used, with Full MS/dd-MS2 (TOP 8) scanning mode. The scan range was 100–1200 *m*/*z*. The capillary temperature was set to 320 °C, and the auxiliary gas heater temperature was set to 350 °C, sheath gas flow rate to 5.0 arbitrary units (a.u.), and auxiliary gas flow rate to 8 a.u.

#### 4.1.5. Chemical Component Database Construction for Three Rhodiola Species

The chemical components of the three *Rhodiola* species were compiled by searching the TCM integrated database (TCMID), TCM pharmacological database (TCMSP), Chinese Academy of Sciences Shanghai Institute of Organic Chemistry’s TCM and chemical component library, TCMIP platform, and NPASS database. Relevant literature on chemical component identification was also retrieved from CNKI, Wanfang, VIP, and Web of Science (WOS) databases. The database includes the compound number, name, molecular formula, and accurate molecular weight.

#### 4.1.6. Data Analysis and Metabolite Identification

Raw UPLC–QE–MS data were processed using Progenesis QI v3.0 software (Nonlinear Dynamics, Newcastle, UK) for baseline filtering, peak detection, integration, retention time correction, peak alignment, and normalization. Peak detection and alignment were conducted using the built-in optimized workflows of Progenesis QI, including automated baseline correction and retention time alignment applied consistently across all samples. Peaks with missing values exceeding 50% were removed prior to further analysis. Metabolite identification was performed based on accurate mass measurements, MS/MS fragmentation patterns, isotopic distribution, and retention time information by searching against a Traditional Chinese Medicine (TCM) compound database. The database contains more than 5000 reference compounds covering major classes of plant secondary metabolites, including alkaloids, phenolic acids, flavonoids, coumarins, phenylpropanoids, lignans, and terpenoids.

For compound annotation, candidate metabolites were retained when the overall matching score exceeded 50, or when the overall score exceeded 40 with an MS/MS spectral matching score greater than 50. Retention time information was used as supportive evidence rather than as an absolute identification criterion.

To ensure transparency and reproducibility, the experimental MS/MS spectra of all annotated metabolites, together with their corresponding reference fragmentation information from the database, are provided in the Supplementary Materials (MS/MS spectra for metabolite identification). Accordingly, all metabolites reported in this study are considered putatively identified.

#### 4.1.7. Differential Component Analysis Methodology

OECloud tools (https://cloud.oebiotech.com, accessed on 12 June 2025) were used for statistical analysis, including PCA and PLS-DA, to build reliable models for analyzing the differences, grouping trends, and correlations of chemical components in *R. crenulata*, *R. kirilowii*, and *R. rosea*, and to identify differential marker compounds based on variable importance (VIP).

### 4.2. Methodology for Screening Anti-Hypoxia Active Components

#### 4.2.1. Experimental Animals

In total, 40 male BALB/c mice (8 weeks old, 20–25 g) were obtained from Spbef (Beijing, China) Biotechnology Co., Ltd., and housed at Beijing University of Chinese Medicine with a 6-day acclimation period prior to experiments. The animal study protocol was approved by the Institutional Animal Care and Use Committee of Beijing University of Chinese Medicine (Approval No. BUCM-2024012207-1047, approved on 8 March 2024) and was conducted in accordance with the Guidelines for the Care and Use of Laboratory Animals.

#### 4.2.2. Drugs

The *R. crenulata* (Batch numbers: 202201, 202202, 202203, 202204, 202205, 202206, 202207, 202208, 202209, 202210); *R. kirilowii* (Batch numbers: 202301, 202302, 202303, 202304, 202305, 202306, 202307, 202308, 202309, 202310); *R. rosea* (Batch numbers: 20230205, 20220605, 20211213, 20230524, 20221125, 20220817, 20220123, 20231208, 20220105, 20230902); Salidroside (purity ≥ 98%, Batch No. RH562262, Shanghai Yien Chemical Technology Co., Ltd., Shanghai, China).

#### 4.2.3. Animal Grouping and Dosage

The 40 mice were randomly divided into 5 groups (8 mice per group): Blank group, Positive drug group (Salidroside), *R. crenulata* group, *R. kirilowii* group, *R. rosea* group. After a 6-day adaptation period, different drugs were administered via gavage once a day for 7 consecutive days.

#### 4.2.4. Experimental Process

For hypoxia tolerance testing, a 250 mL wide-mouth bottle was used, filled with 10 g of soda lime, and the bottle was sealed with petroleum jelly to ensure airtightness. A mouse was placed inside each bottle, and the bottle was sealed immediately. The survival status and hypoxia survival time of the mice were monitored throughout the experiment.

#### 4.2.5. Data Processing

The data were analyzed using SPSS 23.0. Continuous variables were expressed as means ± standard deviation (x ± s). Independent t-tests or non-parametric tests were used to compare two groups. *p* < 0.05 was considered statistically significant, and *p* < 0.01 was considered highly significant.

#### 4.2.6. PLS Regression Analysis

SIMCA-P 14.1 software (Umetrics, Umeå, Sweden) was used to establish a PLSR model for the correlation between chemical components and anti-hypoxia effects of *R. crenulata*, *R. kirilowii*, and *R. rosea*, and to screen for bioactive components.

#### 4.2.7. Grey Relational Analysis Methodology

SPSSAU online software (https://www.spssau.com, accessed on 12 June 2025) was used for grey relational analysis. Data were standardized by dividing each value by the mean of its respective sequence. Correlation coefficients were then calculated to establish the relationship between data sets.

### 4.3. Verification of Anti-Hypoxia Active Components Using Molecular Docking

Molecular docking was performed to analyze the interaction between the bioactive components of the three *Rhodiola* species and the HIF-1*α* target. The 3D structures of the active components were obtained from the PubChem database and converted to mol2 format. The HIF-1*α* structure was retrieved from the RCSB protein database. Pre-docking preparation was carried out using Autodock Vina 1.1.2 software and PyMOL (v2.5.0, Schrödinger, LLC, New York, NY, USA) for protein preparation. The docking results were analyzed to select the most stable conformations, and protein-ligand interactions were visualized and analyzed using PyMOL software.

## 5. Conclusions

In this study, an integrated strategy combining chemical profiling, in vivo pharmacological evaluation, chemometric correlation, and molecular docking was applied to compare the chemical composition and anti-hypoxia activity of *R. crenulata*, *R. kirilowii*, and *R. rosea*. The three species exhibited highly similar metabolite profiles and comparable protective effects in a normobaric hypoxia model. Through an integrated GRA–PLSR screening framework, six metabolites—epicatechin, 3-O-galloylquinic acid, salidroside, p-coumaric acid-4-O-glucoside, citric acid, and geraniol—were identified as biologically plausible contributors to anti-hypoxia activity. These findings elucidate the chemical–pharmacological relationships underlying the effects of *Rhodiola* species and provide a scientific foundation for their rational evaluation and sustainable utilization. The integrated workflow established in this study also offers a generalizable methodological reference for identifying functional constituents in complex botanical medicines.

## Figures and Tables

**Table 1:** Comparison of differential components.

Comparison	Up-Regulation	Down-Regulation	Total Number
*R. crenulata* vs. *R. rosea* vs. *R. kirilowii*	30		30
*R. rosea* vs. *R. crenulata*	20	8	28
*R. kirilowii* vs. *R. crenulata*	16	10	26
*R. kirilowii* vs. *R. rosea*	19	12	31

**Table 2:** Survival time of mice in each group under hypoxia (min, x ± s).

Group	*n*	Survival Time
BC	8	39.16 ± 4.42
PC	8	44.34 ± 3.02 *
RC	8	47.07 ± 5.42 **
RK	8	44.82 ± 6.89 *
RR	8	48.16 ± 4.80 **

Note: BC: blank control group; PC: positive control group; RC: *R. crenulata* group; RK: *R. kirilowii* group; RR: *R. rosea* group. * *p* < 0.05 and ** *p* < 0.01 indicate statistically significant differences compared with the blank control group.

**Table 3:** Molecular docking results of targeted functional compounds with HIF-1*α*.

Component Name	Docking Protein	Docking Score
Geraniol	HIF-1*α*	−4.5
4-O-*β*-Glucopyranosyl-cis-coumaric acid	HIF-1*α*	−5.9
Epicatechin	HIF-1*α*	−6.4
3-Galloylquinic acid	HIF-1*α*	−6.1
Citric acid	HIF-1*α*	−5.3
Salidroside	HIF-1*α*	−6.1

## Data Availability

The data supporting the findings of this study are available from the corresponding author upon reasonable request. All relevant data will also be made publicly available in an appropriate data repository after publication.

## Supplementary Materials

The following supporting information can be downloaded at: https://www.mdpi.com/article/10.3390/ijms27052203/s1

## Abbreviations

The following abbreviations are used in this manuscript:

PCA	Principal component analysis
PLSR	Partial least square
PLS-DA	Least squares discriminant analysis
UPLC-QE-MS	Ultra-high-performance liquid chromatography quadrupole electrostatic field orbital trap mass spectrometry
VIP	Variable importance in the projection

## Appendix A

### Target Components for Correlation Analysis

**No.**	**Molecular Formula**	**Component Name**	**Component Type**
1	C_14_H_20_O_7_	Salidroside	Sugars and Glycosides
2	C_21_H_20_O_11_	Rhodionin	Flavonoids
3	C_22_H_18_O_10_	Epicatechin gallate	Flavonoids
4	C_6_H_8_O_7_	Citric acid	Organic Acids and Derivatives
5	C_12_H_22_O_11_	*α*-Lactose	Sugars and Glycosides
6	C_27_H_30_O_16_	Rhodiosin	Flavonoids
7	C_14_H_16_O_10_	3-Galloylquinic acid	Phenols
8	C_15_H_14_O_6_	Epicatechin	Flavonoids
9	C_30_H_26_O_12_	Procyanidin B4	Flavonoids
10	C_16_H_28_O_7_	Rosiridin	Terpenoids
11	C_6_H_12_O_7_	Gluconic acid	Sugars and Glycosides
12	C_14_H_18_O_9_	Phaseoloidin	Sugars and Glycosides
13	C_21_H_36_O_10_	A-d-Glucopyranoside	Sugars and Glycosides
14	C_10_H_13_N_5_O_4_	Adenosine	Nucleotides and Derivatives
15	C_10_H_18_O	Geraniol	Terpenoids
16	C_18_H_32_O_16_	Maltotriose	Sugars and Glycosides
17	C_15_H_21_NO_7_	N-(1-Deoxy-1-fructosyl)phenylalanine	Amino Acids and Peptides
18	C_15_H_18_O_8_	4-O-*β*-Glucopyranosyl-cis-coumaric acid	Sugars and Glycosides
19	C_7_H_12_O_6_	Quinic acid	Organic Acids and Derivatives
20	C_21_H_20_O_10_	Kaempferol-7-O-rhamnoside	Flavonoids
21	C_5_H_9_NO_4_	L-Glutamic acid	Amino Acids and Peptides
22	C_12_H_22_O_11_	Trehalose	Sugars and Glycosides
23	C_9_H_11_NO_2_	L-Phenylalanine	Amino Acids and Peptides
24	C_6_H_14_N_4_O_2_	L-Arginine	Amino Acids and Peptides
25	C_27_H_30_O_15_	Ternatumoside II	Flavonoids
26	C_16_H_20_O_9_	Trans-ferulic acid-4-*β*-glucoside	Sugars and Glycosides
27	C_10_H_18_O_2_	Decenoic acid	Fatty Acyls
28	C_12_H_23_NO_10_	6-(*α*-d-Glucosaminyl)-1D-myo-inositol	Sugars and Glycosides
29	C_10_H_13_N_5_O_5_	Guanosine	Nucleotides and Derivatives
30	C_45_H_38_O_18_	Procyanidin C1	Flavonoids
31	C_12_H_23_NO_7_	N-(1-Deoxy-1-fructosyl)leucine	Amino Acids and Peptides
32	C_6_H_12_O_6_	Glucose	Sugars and Glycosides
33	C_9_H_13_N_3_O_5_	Cytarabine	Sugars and Glycosides
34	C_17_H_29_NO_11_	Neolinustatin	Sugars and Glycosides
35	C_5_H_9_NO_2_	L-Proline	Amino Acids and Peptides
36	C_9_H_10_O	Cinnamyl Alcohol	Phenylpropanoids
37	C_6_H_11_NO_2_	L-Pipecolic acid	Amino Acids and Peptides
38	C_6_H_11_NO_2_	Hygric acid	Alkaloids
39	C_6_H_8_O_7_	Isocitric acid	Organic Acids and Derivatives
40	C_6_H_13_NO_2_	L-Leucine	Amino Acids and Peptides
41	C_12_H_22_O_11_	Sucrose	Sugars and Glycosides
42	C_5_H_10_N_2_O_3_	L-Glutamine	Amino Acids and Peptides
43	C_5_H_7_NO_3_	Pyroglutamic acid	Amino Acids and Peptides
44	C_15_H_21_NO_8_	N-(1-Deoxy-1-fructosyl)tyrosine	Amino Acids and Peptides
45	C_15_H_14_O_7_	Epigallocatechin	Flavonoids
