# Supplementary material for: A “Qualitative–Pharmacological–Correlation–Molecular” Integrated Workflow Reveals HIF-1α–Relevant Anti-Hypoxia Metabolites in Rhodiola Species
Source: Int J Mol Sci. 2026 Feb 26;27(5):2203. doi: 10.3390/ijms27052203 (PMC12984455; doi:10.3390/ijms27052203)
Supplement: Supplementary file 1 [file ijms-27-02203-s001.zip › ijms-4070482 Supplementary/ijms-4070482 Table S1.docx]

**Table S1. LC–MS/MS–based identification and distribution of metabolites in three *Rhodiola* species (*R. crenulata, R. kirilowii*, and *R. rosea*)**

|  | Adducts | Molecular formula | component name | theoretical m/z | *m/z* | Mass Error (ppm) | *t*R (min) | CAS | classification | RC | RK | RR |
| --- | --- | --- | --- | --- | --- | --- | --- | --- | --- | --- | --- | --- |
| 1 | [M－H]^−^ | C_21_H_22_O_12_ | Taxifolin 7-*O*-*β*-D-glucoside | 465.1038 | 465.1035 | -0.0003 | 4.0246 |  | Flavonoids | + | + | + |
| 2 | [M－H]^−^ | C_15_H_12_O_7_ | Taxifolin | 303.0510 | 303.0508 | -0.0002 | 5.0658 | 480-18-2 | Flavonoids | + | + | + |
| 3 | [M+H]^+^ | C_21_H_20_O_10_ | Kaempferol-7-*O*-rhamnoside | 433.1129 | 433.1126 | -0.0003 | 5.8985 |  | Flavonoids | + | − | + |
| 4 | [M+H]^+^ | C_27_H_30_O_15_ | Kaempferol 3-neohesperidoside | 595.1657 | 595.1655 | -0.0002 | 4.5353 | 32602-81-6 | Flavonoids | + | − | + |
| 5 | [M－H]^−^ | C_33_H_40_O_21_ | Kaempferol 3-sophoroside-7-glucoside | 771.1989 | 771.1988 | -0.0001 | 4.0246 |  | Flavonoids | + | − | + |
| 6 | [M－H]^−^ | C_15_H_10_O_6_ | Luteolin | 285.0405 | 285.0403 | -0.0002 | 6.0917 | 491-70-3 | Flavonoids | + | + | + |
| 7 | [M－H]^−^ | C_15_H_10_O_7_ | 6-Hydroxyluteolin | 301.0354 | 301.0360 | 0.0006 | 5.2822 | 18003-33-3 | Flavonoids | + | + | + |
| 8 | [M+H]^+^ | C_21_H_20_O_11_ | Luteolin 5-*O*-glucoside | 449.1078 | 449.1075 | -0.0003 | 4.4400 |  | Flavonoids | + | + | + |
| 9 | [M+H]^+^ | C_21_H_20_O_12_ | 7-[(*β*-D-Glucopyranosyl)oxy]-3',4',5,8-tetrahydroxyflavone | 465.1028 | 465.1025 | -0.0003 | 4.5353 |  | Flavonoids | + | + | + |
| 10 | [M+H]^+^ | C_21_H_20_O_12_ | Spiraeoside | 465.1028 | 465.1027 | -0.0001 | 5.3910 | 20229-56-5 | Flavonoids | + | − | + |
| 11 | [M+H]^+^ | C_21_H_18_O_14_ | Hibifolin | 495.0769 | 495.0766 | -0.0003 | 5.0528 |  | Flavonoids | + | − | − |
| 12 | [M+H]^+^ | C_27_H_30_O_15_ | Ternatumoside II | 595.1657 | 595.1656 | -0.0001 | 5.1062 | 1473419-87-2 | Flavonoids | + | − | + |
| 13 | [M+H]^+^ | C_27_H_30_O_16_ | Rhodiosin | 611.1604 | 611.1604 | 0.0000 | 4.3700 |  | Flavonoids | + | + | + |
| 14 | [M+H]^+^ | C_21_H_20_O_11_ | Rhodionin | 449.1078 | 449.1073 | -0.0005 | 5.5024 |  | Flavonoids | + | + | + |
| 15 | [M+H]^+^ | C_21_H_20_O_12_ | Hyperoside | 465.1028 | 465.1025 | -0.0003 | 4.8035 | 482-36-0 | Flavonoids | + | + | + |
| 16 | [M－H]^−^ | C_26_H_28_O_16_ | Quercetin 3-sambubioside | 595.1305 | 595.1301 | -0.0004 | 4.5635 |  | Flavonoids | + | + | + |
| 17 | [M+H]^+^ | C_27_H_30_O_17_ | 6-Hydroxykaempferol 3,6-diglucoside | 627.1556 | 627.1553 | -0.0003 | 4.2971 |  | Flavonoids | + | + | + |
| 18 | [M－H]^−^ | C_15_H_14_O_6_ | Epicatechin | 289.0718 | 289.0715 | -0.0003 | 4.4136 | 490-46-0 | Flavonoids | + | − | − |
| 19 | [M－H－H_2_O]^−^ | C_15_H_14_O_7_ | Gallocatechin | 287.0561 | 287.0559 | -0.0002 | 5.5267 | 970-73-0 | Flavonoids | + | + | + |
| 20 | [M+H]^+^ | C_15_H_14_O_6_ | Catechin | 291.0863 | 291.0860 | -0.0003 | 4.1455 | 154-23-4 | Flavonoids | + | + | + |
| 21 | [M+H]^+^ | C_15_H_14_O_7_ | Epigallocatechin | 307.0812 | 307.0809 | -0.0003 | 3.9654 | 970-74-1 | Flavonoids | + | + | + |
| 22 | [M+H]^+^ | C_22_H_18_O_10_ | Epicatechin gallate | 443.0973 | 443.0969 | -0.0004 | 4.8511 | 1257-08-5 | Flavonoids | + | + | + |
| 23 | [M+H]^+^ | C_30_H_26_O_12_ | Procyanidin B4 | 579.1497 | 579.1493 | -0.0004 | 4.2369 | 4852-22-6 | Flavonoids | + | + | + |
| 24 | [M+H]^+^ | C_45_H_38_O_18_ | Procyanidin C1 | 867.2131 | 867.2125 | -0.0006 | 4.3914 | 37064-30-5 | Flavonoids | + | + | + |
| 25 | [M+H]^+^ | C_8_H_10_O_2_ | 4-Hydroxyphenyl ethanol | 139.0753 | 139.0753 | 0.0000 | 4.0700 | 501-94-0 | Phenols | + | + | + |
| 26 | [M+H]^+^ | C_7_H_6_O_2_ | 3-Hydroxybenzaldehyde | 123.0441 | 123.0442 | 0.0001 | 4.6930 | 100-83-4 | Phenols | + | + | + |
| 27 | [M+H]^+^ | C_8_H_8_O_3_ | Isovanillin | 153.0546 | 153.0546 | 0.0000 | 4.9644 |  | Phenols | + | + | + |
| 28 | [M+H]^+^ | C_9_H_10_O_4_ | Syringaldehyde | 183.0652 | 183.0651 | -0.0001 | 4.9397 |  | Phenols | + | + | + |
| 29 | [M－H]^−^ | C_11_H_8_O_5_ | Purpurogallin | 219.0299 | 219.0294 | -0.0005 | 5.9155 | 569-77-7 | Phenols | + | + | + |
| 30 | [M+H]^+^ | C_7_H_6_O_5_ | Gallic acid | 171.0287 | 171.0287 | 0.0000 | 1.8100 | 149-91-7 | Benzene and substituted derivatives | + | + | + |
| 31 | [M+H]^+^ | C_7_H_6_O_3_ | 3,4-Dihydroxybenzaldehyde | 139.0390 | 139.0389 | -0.0001 | 4.0962 | 139-85-5 | Phenols | + | + | + |
| 32 | [M－H]^−^ | C_13_H_8_O_7_ | 3,4,8,9,10-Pentahydroxy Urolithin | 275.0197 | 275.0196 | -0.0001 | 4.5908 | 91485-02-8 | Phenols | + | + | + |
| 33 | [M－H]^−^ | C_15_H_22_O_5_ | Octyl gallate | 281.1394 | 281.1393 | -0.0001 | 9.9084 | 1034-01-1 | Phenols | + | + | + |
| 34 | [M－H]^−^ | C_15_H_8_O_7_ | Demethylwedelolactone | 299.0197 | 299.0196 | -0.0001 | 4.9967 |  | Phenols | + | + | + |
| 35 | [M+H]^+^ | C_14_H_16_O_10_ | 3-Galloylquinic acid | 345.0816 | 345.0813 | -0.0003 | 1.7646 | 17365-11-6 | Phenols | + | + | + |
| 36 | [M+H]^+^ | C_20_H_28_O_10_ | rosavin | 429.1744 | 429.1744 | 0.0000 | 4.9200 |  | Phenylpropanoids | + | + | + |
| 37 | [M－H]^−^ | C_9_H_10_O_3_ | L-3-Phenyllactic acid | 165.0557 | 165.0549 | -0.0008 | 4.9830 | 20312-36-1 | Phenylpropanoids | + | + | + |
| 38 | [M－H]^−^ | C_9_H_10_O_4_ | Hydroxyphenyllactic acid | 181.0506 | 181.0498 | -0.0008 | 3.8506 | 306-23-0 | Phenylpropanoids | + | + | + |
| 39 | [M－H]^−^ | C_21_H_24_O_9_ | Isorhapontin | 419.1348 | 419.1347 | -0.0001 | 5.0511 |  | Phenylpropanoids | + | + | + |
| 40 | [M+HCOO]^−^ | C_20_H_22_O_8_ | Polydatin | 435.1291 | 435.1293 | 0.0002 | 4.7912 | 27208-80-6 | Phenylpropanoids | + | + | + |
| 41 | [M+H－H_2_O]^+^ | C_9_H_10_O_2_ | Cinnamyl Alcohol | 117.0699 | 117.0701 | 0.0002 | 6.4052 | 104-54-1 | Phenylpropanoids | + | + | + |
| 42 | [M－H]^−^ | C_10_H_10_O_3_ | Methyl p-coumarate | 177.0557 | 177.0549 | -0.0008 | 6.5926 | 3943-97-3 | Phenylpropanoids | + | + | + |
| 43 | [M+H]^+^ | C_10_H_10_O_3_ | Coniferaldehyde | 179.0703 | 179.0702 | -0.0001 | 5.5569 | 20649-42-7 | Phenylpropanoids | + | + | + |
| 44 | [M+H]^+^ | C_11_H_12_O_4_ | Ferulic acid methyl ester | 209.0808 | 209.0807 | -0.0001 | 6.8330 |  | Phenylpropanoids | + | + | + |
| 45 | [M+H]^+^ | C_20_H_20_O_6_ | (+)-Balanophonin | 357.1333 | 357.1329 | -0.0004 | 6.4611 | 215319-47-4 | Phenylpropanoids | + | + | + |
| 46 | [M－H]^−^ | C_26_H_32_O_11_ | Pinoresinol 4-*O*-*β*-D-glucopyranoside | 519.1872 | 519.1869 | -0.0003 | 5.0241 | 69251-96-3 | Phenylpropanoids | + | + | + |
| 47 | [M+H]^+^ | C_15_H_8_O_7_ | Isodemethylwedelolactone | 301.0343 | 301.0340 | -0.0003 | 5.2805 | 350681-33-3 | Phenylpropanoids | + | − | + |
| 48 | [M+NH_4_]^+^ | C_10_H_8_O_3_ | 6-hydroxy-4-methylcoumarin | 194.0812 | 194.0811 | -0.0001 | 5.0676 |  | Phenylpropanoids | − | + | + |
| 49 | [M+H]^+^ | C_9_H_8_O_2_ | Dihydrocoumarin | 149.0597 | 149.0597 | 0.0000 | 5.3489 | 119-84-6 | Phenylpropanoids | + | + | + |
| 50 | [M+H－H_2_O]^+^ | C_20_H_22_O_6_ | Pinoresinol | 341.1383 | 341.1381 | -0.0002 | 6.4749 | 487-36-5 | Phenylpropanoids | + | + | + |
| 51 | [M+HCOO]^−^ | C_20_H_24_O_7_ | Cycloolivil | 421.1499 | 421.1503 | 0.0004 | 4.9278 |  | Phenylpropanoids | + | + | + |
| 52 | [M－H]^−^ | C_26_H_32_O_11_ | Matairesinoside | 519.1872 | 519.1869 | -0.0003 | 5.3409 |  | Phenylpropanoids | + | + | + |
| 53 | [M+H]^+^ | C_7_H_12_N_2_O_4_ | Aceglutamide | 189.0870 | 189.0869 | -0.0001 | 1.1141 | 2490-97-3 | Amino Acids, Peptides and derivatives | − | + | − |
| 54 | [M+H]^+^ | C_4_H_9_NO_2_ | gamma-Aminobutyric acid | 104.0706 | 104.0709 | 0.0003 | 0.7825 | 56-12-2 | Amino Acids, Peptides and derivatives | + | + | + |
| 55 | [M+H]^+^ | C_5_H_9_NO_2_ | L-Proline | 116.0706 | 116.0708 | 0.0002 | 0.8046 | 147-85-3 | Amino Acids, Peptides and derivatives | + | + | + |
| 56 | [M+H]^+^ | C_5_H_7_NO_3_ | Pyroglutamic acid | 130.0499 | 130.0499 | 0.0000 | 1.1541 | 98-79-3 | Amino Acids, Peptides and derivatives | + | + | + |
| 57 | [M+H]^+^ | C_6_H_11_NO_2_ | L-Pipecolic acid | 130.0863 | 130.0863 | 0.0000 | 1.0519 | 3105-95-1 | Amino Acids, Peptides and derivatives | + | + | + |
| 58 | [M+H]^+^ | C_6_H_13_NO_2_ | L-Leucine | 132.1019 | 132.1019 | 0.0000 | 1.4905 | 61-90-5 | Amino Acids, Peptides and derivatives | + | + | + |
| 59 | [M+H]^+^ | C_5_H_10_N_2_O_3_ | L-Glutamine | 147.0764 | 147.0763 | -0.0001 | 0.7914 | 56-85-9 | Amino Acids, Peptides and derivatives | + | + | + |
| 60 | [M+H]^+^ | C_5_H_9_NO_4_ | L-Glutamic acid | 148.0604 | 148.0603 | -0.0001 | 0.7761 | 56-86-0 | Amino Acids, Peptides and derivatives | + | + | + |
| 61 | [M+H]^+^ | C_7_H_13_NO_3_ | N-Acetylvaline | 160.0968 | 160.0967 | -0.0001 | 3.9325 | 96-81-1 | Amino Acids, Peptides and derivatives | + | + | + |
| 62 | [M+H]^+^ | C_9_H_11_NO_2_ | L-Phenylalanine | 166.0863 | 166.0862 | -0.0001 | 2.3593 | 63-91-2 | Amino Acids, Peptides and derivatives | + | + | + |
| 63 | [M+H]^+^ | C_6_H_14_N_4_O_2_ | L-Arginine | 175.1190 | 175.1189 | -0.0001 | 0.7444 | 74-79-3 | Amino Acids, Peptides and derivatives | + | + | + |
| 64 | [M－H]^−^ | C_9_H_11_NO_3_ | L-Tyrosine | 180.0666 | 180.0658 | -0.0008 | 1.2578 | 60-18-4 | Amino Acids, Peptides and derivatives | + | + | + |
| 65 | [M+H]^+^ | C_10_H_13_NO_4_ | 3-Methoxytyrosine | 212.0917 | 212.0917 | 0.0000 | 1.6820 | 7636-26-2 | Amino Acids, Peptides and derivatives | + | + | + |
| 66 | [M－H]^−^ | C_12_H_23_NO_7_ | N-(1-Deoxy-1-fructosyl)leucine | 292.1402 | 292.1400 | -0.0002 | 1.4683 | 34393-18-5 | Amino Acids, Peptides and derivatives | + | + | + |
| 67 | [M+H]^+^ | C_15_H_21_NO_7_ | N-(1-Deoxy-1-fructosyl)phenylalanine | 328.1391 | 328.1388 | -0.0003 | 2.3593 | 87251-83-0 | Amino Acids, Peptides and derivatives | + | + | + |
| 68 | [M+H]^+^ | C_15_H_21_NO_8_ | N-(1-Deoxy-1-fructosyl)tyrosine | 344.134 | 344.1341 | 0.0001 | 1.0913 | 34393-22-1 | Amino Acids, Peptides and derivatives | + | + | + |
| 69 | [M+H]^+^ | C_11_H_20_N_2_O_5_ | gamma-Glutamylleucine | 261.1445 | 261.1443 | -0.0002 | 3.8814 | 2566-39-4 | Amino Acids, Peptides and derivatives | − | + | + |
| 70 | [M－H]^−^ | C_8_H_15_NO_3_ | N-Acetylleucine | 172.0979 | 172.0971 | -0.0008 | 4.5772 | 1188-21-2 | Amino Acids, Peptides and derivatives | + | + | + |
| 71 | [M+H]^+^ | C_8_H_16_N_2_O_3_ | Glycyl-Isoleucine | 189.1234 | 189.1233 | -0.0001 | 2.7139 |  | Amino Acids, Peptides and derivatives | + | + | + |
| 72 | [M+H]^+^ | C_7_H_11_NO_5_ | N-Acetyl-L-glutamic acid | 190.071 | 190.0709 | -0.0001 | 1.2178 | 1188-37-0 | Amino Acids, Peptides and derivatives | + | + | + |
| 73 | [M+H]^+^ | C_8_H_16_N_2_O_4_ | Serylvaline | 205.1183 | 205.1182 | -0.0001 | 1.2362 | 51782-06-0 | Amino Acids, Peptides and derivatives | + | + | + |
| 74 | [M－H]^−^ | C_11_H_13_NO_3_ | N-Acetyl-L-phenylalanine | 206.0823 | 206.0816 | -0.0007 | 4.8042 | 2018-61-3 | Amino Acids, Peptides and derivatives | + | + | + |
| 75 | [M+H]^+^ | C_8_H_16_N_4_O_3_ | N-Acetylarginine | 217.1295 | 217.1295 | 0.0000 | 1.0519 | 155-84-0 | Amino Acids, Peptides and derivatives | + | + | + |
| 76 | [M－H]^−^ | C_9_H_17_NO_5_ | Pantothenic acid | 218.1034 | 218.1028 | -0.0006 | 3.0035 | 79-83-4 | Amino Acids, Peptides and derivatives | + | + | + |
| 77 | [M+H]^+^ | C_10_H_19_N_3_O_4_ | Asparaginyl-Leucine | 246.1448 | 246.1447 | -0.0001 | 2.8025 |  | Amino Acids, Peptides and derivatives | + | + | + |
| 78 | [M+H]^+^ | C_10_H_18_N_2_O_5_ | Aspartyl-Leucine | 247.1288 | 247.1287 | -0.0001 | 3.3091 |  | Amino Acids, Peptides and derivatives | + | + | + |
| 79 | [M+H]^+^ | C_11_H_20_N_2_O_5_ | Glutamylleucine | 261.1445 | 261.1443 | -0.0002 | 3.3865 | 5969-52-8 | Amino Acids, Peptides and derivatives | + | + | + |
| 80 | [M+H]^+^ | C_11_H_20_N_2_O_5_ | Isoleucyl-Glutamate | 261.1445 | 261.1443 | -0.0002 | 1.4954 |  | Amino Acids, Peptides and derivatives | + | + | + |
| 81 | [M+H]^+^ | C_11_H_19_N_3_O_6_ | Ophthalmic acid | 290.1347 | 290.1343 | -0.0004 | 1.1333 | 495-27-2 | Amino Acids, Peptides and derivatives | + | + | + |
| 82 | [M－H]^−^ | C_20_H_32_N_6_O_12_S_2_ | Oxidized glutathione | 611.1447 | 611.1442 | -0.0005 | 1.1228 | 27025-41-8 | Amino Acids, Peptides and derivatives | + | + | + |
| 83 | [M－H]^−^ | C_10_H_12_N_4_O_5_ | Inosine | 267.0735 | 267.0732 | -0.0003 | 1.4254 | 58-63-9 | Nucleotides and derivatives | + | + | + |
| 84 | [M+H]^+^ | C_10_H_13_N_5_O_4_ | Adenosine | 268.104 | 268.1038 | -0.0002 | 1.3075 | 58-61-7 | Nucleotides and derivatives | + | + | + |
| 85 | [M+H]^+^ | C_10_H_13_N_5_O_5_ | Guanosine | 284.0989 | 284.0987 | -0.0002 | 1.4233 | 118-00-3 | Nucleotides and derivatives | + | + | + |
| 86 | [M+HCOO]^−^ | C_10_H_14_N_2_O_5_ | Thymidine | 287.0885 | 287.0881 | -0.0004 | 2.3297 | 50-89-5 | Nucleotides and derivatives | + | + | + |
| 87 | [M+H]^+^ | C_10_H_14_N_5_O_8_P | Guanosine monophosphate | 364.0653 | 364.0649 | -0.0004 | 1.0519 | 85-32-5 | Nucleotides and derivatives | + | + | + |
| 88 | [M+H]^+^ | C_14_H_17_N_5_O_8_ | Succinyladenosine | 384.1150 | 384.1146 | -0.0004 | 3.3864 | 4542-23-8 | Nucleotides and derivatives | + | + | + |
| 89 | [M+HCOO]^−^ | C_20_H_26_O_11_ | Regaloside B | 487.1452 | 487.1453 | 0.0001 | 4.8403 | 114420-67-6 | Carbohydrates and Glycosides | − | − | + |
| 90 | [M－H]^−^ | C_14_H_18_O_8_ | Glucovanillin | 313.0929 | 313.0929 | 0.0000 | 4.0140 | 494-08-6 | Carbohydrates and Glycosides | + | + | + |
| 91 | [M－H]^−^ | C_12_H_16_O_7_ | Arbutin | 317.0879 | 317.0875 | -0.0004 | 1.4423 | 497-76-7 | Carbohydrates and Glycosides | + | + | + |
| 92 | [M－H]^−^ | C_14_H_18_O_9_ | Phaseoloidin | 329.0878 | 329.0876 | -0.0002 | 1.6523 | 118555-82-1 | Carbohydrates and Glycosides | + | + | + |
| 93 | [M－H]^−^ | C_14_H_18_O_9_ | Vanillic acid 4-*β*-*D*-glucopyranoside | 329.0878 | 329.0876 | -0.0002 | 3.2929 |  | Carbohydrates and Glycosides | + | + | + |
| 94 | [M－H]^−^ | C_15_H_18_O_8_ | 4-*O*-*β*-Glucopyranosyl-cis-coumaric acid | 371.0984 | 371.0979 | -0.0005 | 3.7844 |  | Carbohydrates and Glycosides | + | + | + |
| 95 | [M+HCOO]^−^ | C_16_H_22_O_8_ | Coniferin | 387.1291 | 387.1292 | 0.0001 | 3.9686 | 124151-33-3 | Carbohydrates and Glycosides | + | + | + |
| 96 | [M+HCOO]^−^ | C_16_H_20_O_9_ | Trans-ferulic acid-4-*β*-glucoside | 401.1084 | 401.1085 | 0.0001 | 3.9686 | 537-98-4 | Carbohydrates and Glycosides | + | + | + |
| 97 | [M－H]^−^ | C_17_H_29_NO_11_ | Neolinustatin | 468.1723 | 468.1719 | -0.0004 | 3.5671 | 72229-42-6 | Carbohydrates and Glycosides | + | + | + |
| 98 | [M+HCOO]^−^ | C_14_H_20_O_7_ | Salidroside | 345.1186 | 345.1186 | 0.0000 | 3.8506 | 10338-51-9 | Carbohydrates and Glycosides | + | + | + |
| 99 | [M－H]^−^ | C_6_H_10_O_6_ | 1,4-*D*-Gulonolactone | 177.0405 | 177.0397 | -0.0008 | 1.2038 | 3327-64-8 | Carbohydrates and Glycosides | + | + | + |
| 100 | [M+H]^+^ | C_6_H_13_NO_5_ | Manosamine | 180.0866 | 180.0865 | -0.0001 | 0.7566 |  | Carbohydrates and Glycosides | + | + | + |
| 101 | [M－H]^−^ | C_6_H_14_O_6_ | Galactitol | 181.0718 | 181.0709 | -0.0009 | 0.7544 | 608-66-2 | Carbohydrates and Glycosides | + | + | + |
| 102 | [M－H－H_2_O]^−^ | C_7_H_14_O_7_ | D-altrofurano-heptulose-3 | 191.0560 | 191.0552 | -0.0008 | 1.2038 | 25545-06-6 | Carbohydrates and Glycosides | + | + | + |
| 103 | [M－H]^−^ | C_6_H_10_O_7_ | Galacturonic acid | 193.0354 | 193.0346 | -0.0008 | 0.7911 |  | Carbohydrates and Glycosides | + | + | + |
| 104 | [M+HCOO]^−^ | C_5_H_8_O_5_ | Ribonolactone | 193.0348 | 193.0347 | -0.0001 | 0.5070 | 5336-08-3 | Carbohydrates and Glycosides | + | + | + |
| 105 | [M－H]^−^ | C_6_H_12_O_7_ | Gluconic acid | 195.0510 | 195.0503 | -0.0007 | 0.7698 | 526-95-4 | Carbohydrates and Glycosides | + | + | + |
| 106 | [M+H]^+^ | C_8_H_15_NO_6_ | N-Acetyl-D-glucosamine | 204.0866 | 204.0866 | 0.0000 | 0.8046 | 7512-17-6 | Carbohydrates and Glycosides | + | + | + |
| 107 | [M+HCOO]^−^ | C_6_H_12_O_6_ | Glucose | 225.0610 | 225.0610 | 0.0000 | 0.7911 | 921-60-8 | Carbohydrates and Glycosides | + | + | + |
| 108 | [M－H]^−^ | C_9_H_12_N_2_O_6_ | 1-*β*-*D*-Arabinofuranosyluracil | 243.0623 | 243.0619 | -0.0004 | 1.1772 |  | Carbohydrates and Glycosides | + | + | + |
| 109 | [M+H]^+^ | C_9_H_13_N_3_O_5_ | Cytarabine | 244.0928 | 244.0927 | -0.0001 | 0.9684 | 147-94-4 | Carbohydrates and Glycosides | + | + | + |
| 110 | [M－H]^−^ | C_6_H_13_O_9_P | Galactose 1-phosphate | 259.0224 | 259.0221 | -0.0003 | 0.7295 | 2255-14-3 | Carbohydrates and Glycosides | + | + | + |
| 111 | [M+H－H_2_O]^+^ | C_12_H_22_O_11_ | Trehalose | 325.1129 | 325.1127 | -0.0002 | 0.7868 | 99-20-7 | Carbohydrates and Glycosides | + | + | + |
| 112 | [M－H]^−^ | C_12_H_18_O_11_ | 2-*O*-*β*-*D*-Glucopyranosyl-L-ascorbic acid | 337.0776 | 337.0774 | -0.0002 | 1.0827 |  | Carbohydrates and Glycosides | + | + | + |
| 113 | [M+H]^+^ | C_12_H_23_NO_10_ | 6-(*α*-D-Glucosaminyl)-1D-myo-inositol | 342.1395 | 342.1391 | -0.0004 | 0.7566 |  | Carbohydrates and Glycosides | + | + | + |
| 114 | [M+H]^+^ | C_12_H_22_O_11_ | Sucrose | 365.1055 | 365.1051 | -0.0004 | 0.8224 | 57-50-1 | Carbohydrates and Glycosides | + | + | + |
| 115 | [M－H]^−^ | C_12_H_22_O_11_ | *α*-Lactose | 387.1145 | 387.1141 | -0.0004 | 0.7818 | 63-42-3 | Carbohydrates and Glycosides | + | + | + |
| 116 | [M+HCOO]^−^ | C_21_H_36_O_10_ | A-*D*-Glucopyranoside | 493.2291 | 493.2288 | -0.0003 | 5.9892 | 88700-35-0 | Carbohydrates and Glycosides | + | + | + |
| 117 | [M－H]^−^ | C_18_H_32_O_16_ | Maltotriose | 549.1673 | 549.1670 | -0.0003 | 0.8005 |  | Carbohydrates and Glycosides | + | + | + |
| 118 | [M+HCOO]^−^ | C_24_H_42_O_21_ | Stachyose | 711.2195 | 711.2201 | 0.0006 | 0.7818 | 470-55-3 | Carbohydrates and Glycosides | + | + | + |
| 119 | [M+H－H_2_O]^+^ | C_15_H_26_O | Patchouli alcohol | 205.1951 | 205.1950 | -0.0001 | 11.7718 | 5986-55-0 | Terpenes | + | + | + |
| 120 | [M+H－H_2_O]^+^ | C_15_H_26_O | *β*-Eudesmol | 205.1951 | 205.1950 | -0.0001 | 11.2255 | 473-15-4 | Terpenes | + | + | + |
| 121 | [M+H－H_2_O]^+^ | C_15_H_20_O_3_ | Micheliolide | 231.138 | 231.1379 | -0.0001 | 8.5007 | 68370-47-8 | Terpenes | + | + | + |
| 122 | [M+H－H_2_O]^+^ | C_15_H_20_O_3_ | 1*β*-Hydroxyalantolactone | 231.138 | 231.1379 | -0.0001 | 7.4445 | 68776-47-6 | Terpenes | + | + | + |
| 123 | [M+H]^+^ | C_15_H_20_O_3_ | Atractylenolide III | 249.1485 | 249.1483 | -0.0002 | 9.4561 |  | Terpenes | + | + | + |
| 124 | [M+H]^+^ | C_19_H_26_O_7_ | Diacetoxyscirpenol | 367.1751 | 367.1753 | 0.0002 | 6.8796 | 2270-40-8 | Terpenes | + | + | + |
| 125 | [M+H－H_2_O]^+^ | C_10_H_18_O | Geraniol | 137.1325 | 137.1324 | -0.0001 | 9.2707 | 106-24-1 | Terpenes | + | + | + |
| 126 | [M+NH_4_]^+^ | C_16_H_28_O_7_ | Rosiridin | 350.2173 | 350.2170 | -0.0003 | 4.9888 |  | Terpenes | + | + | + |
| 127 | [M－H－H_2_O]^−^ | C_16_H_22_O_10_ | Secologanic acid | 355.1034 | 355.1032 | -0.0002 | 3.9686 | 60077-46-5 | Terpenes | + | + | + |
| 128 | [M－H]^−^ | C_11_H_20_O_6_ | Crenulatin | 247.1187 | 247.1183 | -0.0004 | 4.2455 | 63026-02-8 | Terpenes | + | + | + |
| 129 | [M+H]^+^ | C_11_H_16_O_2_ | Dihydroactinidiolide | 181.1223 | 181.1222 | -0.0001 | 8.1254 | 15356-74-8 | Terpenes | + | + | + |
| 130 | [M+H]^+^ | C_9_H_8_O_4_ | Caffeic acid | 181.0495 | 181.0494 | -0.0001 | 4.0486 | 501-16-6 | Organic acids and derivatives | + | + | + |
| 131 | [M－H]^−^ | C_10_H_10_O_4_ | Ferulic acid | 193.0506 | 193.0499 | -0.0007 | 5.0374 | 537-98-4 | Organic acids and derivatives | + | + | + |
| 132 | [M+H]^+^ | C_9_H_8_O_3_ | *p*-Coumaric acid | 165.0546 | 165.0545 | -0.0001 | 4.8630 | 4501-31-9 | Organic acids and derivatives | + | + | + |
| 133 | [M+H－H_2_O]^+^ | C_7_H_12_O_6_ | Quinic acid | 175.0601 | 175.0600 | -0.0001 | 0.7957 | 77-95-2 | Organic acids and derivatives | + | + | + |
| 134 | [M+H]^+^ | C_6_H_8_O_6_ | Ascorbic acid | 177.0394 | 177.0393 | -0.0001 | 1.0906 | 50-81-7 | Organic acids and derivatives | + | + | + |
| 135 | [M－H]^−^ | C_6_H_8_O_7_ | Citric acid | 191.0197 | 191.0189 | -0.0008 | 1.1350 | 77-92-9 | Organic acids and derivatives | + | + | + |
| 136 | [M－H]^−^ | C_7_H_10_O_7_ | 2-Methylcitric acid | 205.0354 | 205.0347 | -0.0007 | 1.6382 | 6061-96-7 | Organic acids and derivatives | + | + | + |
| 137 | [M+H]^+^ | C_10_H_7_NO_4_ | Xanthurenic acid | 206.0448 | 206.0447 | -0.0001 | 3.5051 | 59-00-7 | Organic acids and derivatives | + | + | + |
| 138 | [M+NH_4_]^+^ | C_6_H_8_O_7_ | Isocitric acid | 210.0608 | 210.0608 | 0.0000 | 0.9925 | 320-77-4 | Organic acids and derivatives | + | + | + |
| 139 | [M－H]^−^ | C_10_H_18_O_5_ | 3-Hydroxysebacic acid | 217.1081 | 217.1076 | -0.0005 | 4.9682 | 446881-43-2 | Organic acids and derivatives | + | + | + |
| 140 | [M+H－H_2_O]^+^ | C_15_H_20_O_4_ | Abscisic acid | 247.1329 | 247.1327 | -0.0002 | 6.0735 | 21293-29-8 | Organic acids and derivatives | + | + | + |
| 141 | [M+H－H_2_O]^+^ | C_10_H_18_O_2_ | Decenoic acid | 153.1274 | 153.1273 | -0.0001 | 6.1641 | 14436-32-9 | Fatty Acyls | + | + | + |
| 142 | [M－H]^−^ | C_8_H_14_O_4_ | Suberic acid | 173.0819 | 173.0811 | -0.0008 | 4.8448 | 505-48-6 | Fatty Acyls | + | + | + |
| 143 | [M+H－H_2_O]^+^ | C_10_H_18_O_4_ | Sebacic acid | 185.1172 | 185.1174 | 0.0002 | 6.0374 | 111-20-6 | Fatty Acyls | + | + | + |
| 144 | [M－H]^−^ | C_9_H_16_O_4_ | Azelaic acid | 187.0976 | 187.0968 | -0.0008 | 5.3677 | 123-99-9 | Fatty Acyls | + | + | + |
| 145 | [M+H－H_2_O]^+^ | C_11_H_20_O_4_ | Undecanedioic acid | 217.1434 | 217.1434 | 0.0000 | 6.8330 | 1852-04-6 | Fatty Acyls | + | + | + |
| 146 | [M－H]^−^ | C_12_H_22_O_4_ | Dodecanedioic acid | 229.1445 | 229.1441 | -0.0004 | 7.6844 | 693-23-2 | Fatty Acyls | + | + | + |
| 147 | [M+H]^+^ | C_18_H_30_O_2_ | Octadecatrienoic acid | 279.2319 | 279.2316 | -0.0003 | 10.8136 | 544-72-9 | Fatty Acyls | + | + | + |
| 148 | [M+H]^+^ | C_18_H_35_NO | Oleamide | 282.2791 | 282.2789 | -0.0002 | 12.8542 | 301-02-0 | Fatty Acyls | + | + | + |
| 149 | [M－H]^−^ | C_16_H_30_O_4_ | Hexadecanedioic acid | 285.2071 | 285.2069 | -0.0002 | 10.2988 | 505-54-4 | Fatty Acyls | + | + | + |
| 150 | [M－H]^−^ | C_18_H_34_O_4_ | Octadecanedioic acid | 313.2384 | 313.2382 | -0.0002 | 11.0602 | 871-70-5 | Fatty Acyls | + | + | + |
| 151 | [M－H]^−^ | C_18_H_34_O_4_ | 12,13-DHOME | 313.2384 | 313.2383 | -0.0001 | 9.9685 | 7293-40-5 | Fatty Acyls | + | + | + |
| 152 | [M+H]^+^ | C_20_H_37_NO_2_ | Linoleoyl ethanolamide | 324.2897 | 324.2895 | -0.0002 | 11.6624 | 68171-52-8 | Fatty Acyls | + | + | + |
| 153 | [M+H]^+^ | C_22_H_43_NO | Docosenamide | 338.3417 | 338.3413 | -0.0004 | 15.1987 |  | Fatty Acyls | + | + | + |
| 154 | [M+H]^+^ | C_6_H_11_NO_2_ | Hygric acid | 130.0863 | 130.0863 | 0.0000 | 0.8981 | 475-11-6 | Alkaloids | + | + | + |
| 155 | [M－H]^−^ | C_10_H_16_O_4_ | Camphoric acid | 199.0976 | 199.0969 | -0.0007 | 5.3499 | 560-09-8 | Carboxylic acid and derivatives | + | + | + |
| 156 | [M+H]^+^ | C_9_H_7_NO | 4-formyl Indole | 146.06 | 146.0600 | 0.0000 | 5.4485 |  | Indoles and derivatives | + | + | + |
| 157 | [M－H]^−^ | C_11_H_12_N_2_O_2_ | L-Tryptophan | 203.0826 | 203.0819 | -0.0007 | 3.7844 | 73-22-3 | Indoles and derivatives | + | + | + |
| 158 | [M+NH_4_]^+^ | C_10_H_9_NO_3_ | 5-Hydroxyindoleacetic acid | 209.0921 | 209.0920 | -0.0001 | 3.7809 | 54-16-0 | Indoles and derivatives | + | + | + |
| 159 | [M－H]^−^ | C_11_H_12_N_2_O_3_ | 5-Hydroxy-L-tryptophan | 219.0775 | 219.0769 | -0.0006 | 1.6600 | 4350-09-8 | Indoles and derivatives | + | + | + |
| 160 | [M+H]^+^ | C_4_H_5_N_3_O | Cytosine | 112.0505 | 112.0508 | 0.0003 | 0.9534 | 71-30-7 | Organoheterocyclic compounds | + | + | + |
| 161 | [M+H]^+^ | C_4_H_4_N_2_O_2_ | Uracil | 113.0346 | 113.0348 | 0.0002 | 1.1709 | 66-22-8 | Organoheterocyclic compounds | + | + | + |
| 162 | [M+H－H_2_O]^+^ | C_5_H_7_N_5_O | FAPy-adenine | 136.0618 | 136.0618 | 0.0000 | 1.0020 | 5122-36-1 | Organoheterocyclic compounds | + | + | + |
| 163 | [M+H]^+^ | C_29_H_48_O | Stigmasterol | 413.3778 | 413.3773 | -0.0005 | 14.7620 |  | Steroids | + | + | − |
| 164 | [M+H]^+^ | C_15_H_14_O_3_ | Lapachol | 243.1016 | 243.1015 | -0.0001 | 10.1593 | 84-79-7 | Quinones | − | + | + |
| 165 | [M+H－H_2_O]^+^ | C_18_H_37_NO_3_ | Dehydrophytosphingosine | 298.2740 | 298.2738 | -0.0002 | 9.3151 | 3687-54-5 | Sphingolipids | + | + | + |
| 166 | [M+H]^+^ | C_18_H_39_NO_3_ | Phytosphingosine | 318.3003 | 318.3000 | -0.0003 | 9.4364 | 554-62-1 | Sphingolipids | + | + | + |
| 167 | [M+H－H_2_O]^+^ | C_7_H_8_O_2_ | 3-Hydroxybenzyl alcohol | 107.0492 | 107.0494 | 0.0002 | 3.4797 | 620-24-6 | Others | + | + | + |
| 168 | [M]^+^ | C_5_H_15_NO_4_P+ | Phosphorylcholine | 184.0739 | 184.0732 | -0.0007 | 0.7666 | 3616-04-4 | Others | + | + | + |
| 169 | [M+H]^+^ | C_12_H_14_O_2_ | Butylphthalide | 191.1067 | 191.1067 | 0.0000 | 9.6613 | 3413-15-8 | Others | + | + | + |
| 170 | [M－H]^−^ | C_7_H_10_O_7_ | Methyl citrate | 205.0354 | 205.0347 | -0.0007 | 1.9868 | 26163-61-1 | Others | + | + | + |
| 171 | [M+H－H_2_O]^+^ | C_13_H_20_O_3_ | Vomifoliol | 207.1379 | 207.1379 | 0.0000 | 4.7550 |  | Others | + | + | + |
| 172 | [M+H]^+^ | C_11_H_12_O_4_ | Methyl kakuol | 209.0808 | 209.0808 | 0.0000 | 8.3905 |  | Others | + | + | + |
| 173 | [M－H]^−^ | C_13_H_24_O_4_ | Tridecanedioic acid | 243.1602 | 243.1597 | -0.0005 | 8.5350 | 505-52-2 | Others | + | + | + |
| 174 | [M－H]^−^ | C_10_H_14_N_2_O_6_ | 2′-O-Methyluridine | 257.0779 | 257.0780 | 0.0001 | 1.9897 | 2140-76-3 | Others | + | + | + |
| 175 | [2M－H]^−^ | C_11_H_8_N_2_O_3_S_2_ | Luciferin | 558.9880 | 558.9875 | -0.0005 | 5.5810 | 2591-17-5 | Others | + | + | + |

Abbreviations:

RC, *Rhodiola crenulata*; RK, *Rhodiola kirilowii*; RR, *Rhodiola rosea*.

“+” indicates detected; “−” indicates not detected.

tR, retention time.

Note: For unsaturated fatty acids and lipid-related metabolites, the exact positions of double bonds or oxidation sites could not be unambiguously determined due to the inherent limitations of LC–MS/MS analysis; therefore, these compounds were annotated at the level of molecular class or isomer without specification of double-bond or oxidation-site positions. In addition, the ion forms reported in this table correspond to the predominant ion species detected under the applied LC–MS conditions. In negative ion mode, some metabolites were detected as formate adducts ([M+HCOO]^-^) due to the presence of formic acid in the mobile phase, while for certain labile compounds, ions corresponding to neutral losses (e.g., −H_2_O) were observed as a result of ionization or in-source fragmentation behavior. The molecular formulas listed throughout the table refer to the intact neutral compounds and do not imply chemical modification of the metabolites.
