# Supplementary material for: A “Qualitative–Pharmacological–Correlation–Molecular” Integrated Workflow Reveals HIF-1α–Relevant Anti-Hypoxia Metabolites in Rhodiola Species
Source: Int J Mol Sci. 2026 Feb 26;27(5):2203. doi: 10.3390/ijms27052203 (PMC12984455; doi:10.3390/ijms27052203)
Supplement: Supplementary file 1 [file ijms-27-02203-s001.zip › ijms-4070482 Table S1.pdf]

Table S1. LC–MS/MS–based identification and distribution of metabolites in three *Rhodiola* species (*R. crenulata, R. kirilowii*, and *R. rosea*)

	Adducts	Molecular formula	component name	theoretical m/z	*m/z*	Mass Error (ppm)	*t*R (min)	CAS	classification	RC	RK	RR
1	$[M-H]^-$	$C_{21}H_{22}O_{12}$	Taxifolin 7-*O*-β-D-glucoside	465.1038	465.1035	-0.0003	4.0246		Flavonoids	+	+	+
2	$[M-H]^-$	$C_{15}H_{12}O_7$	Taxifolin	303.0510	303.0508	-0.0002	5.0658	480-18-2	Flavonoids	+	+	+
3	$[M+H]^+$	$C_{21}H_{20}O_{10}$	Kaempferol-7-*O*-rhamnoside	433.1129	433.1126	-0.0003	5.8985		Flavonoids	+	−	+
4	$[M+H]^+$	$C_{27}H_{30}O_{15}$	Kaempferol 3-neohesperidoside	595.1657	595.1655	-0.0002	4.5353	32602-81-6	Flavonoids	+	−	+
5	$[M-H]^-$	$C_{33}H_{40}O_{21}$	Kaempferol 3-sophoroside-7-glucoside	771.1989	771.1988	-0.0001	4.0246		Flavonoids	+	−	+
6	$[M-H]^-$	$C_{15}H_{10}O_6$	Luteolin	285.0405	285.0403	-0.0002	6.0917	491-70-3	Flavonoids	+	+	+
7	$[M-H]^-$	$C_{15}H_{10}O_7$	6-Hydroxyluteolin	301.0354	301.0360	0.0006	5.2822	18003-33-3	Flavonoids	+	+	+
8	$[M+H]^+$	$C_{21}H_{20}O_{11}$	Luteolin 5-*O*-glucoside	449.1078	449.1075	-0.0003	4.4400		Flavonoids	+	+	+
9	$[M+H]^+$	$C_{21}H_{20}O_{12}$	7-[(β-D-Glucopyranosyl)oxy]-3',4',5,8-tetrahydroxyflavone	465.1028	465.1025	-0.0003	4.5353		Flavonoids	+	+	+
10	$[M+H]^+$	$C_{21}H_{20}O_{12}$	Spiraeoside	465.1028	465.1027	-0.0001	5.3910	20229-56-5	Flavonoids	+	−	+
11	$[M+H]^+$	$C_{21}H_{18}O_{14}$	Hibifolin	495.0769	495.0766	-0.0003	5.0528		Flavonoids	+	−	−
12	$[M+H]^+$	$C_{27}H_{30}O_{15}$	Ternatumoside II	595.1657	595.1656	-0.0001	5.1062	1473419-87-2	Flavonoids	+	−	+
13	$[M+H]^+$	$C_{27}H_{30}O_{16}$	Rhodiosin	611.1604	611.1604	0.0000	4.3700		Flavonoids	+	+	+
14	$[M+H]^+$	$C_{21}H_{20}O_{11}$	Rhodionin	449.1078	449.1073	-0.0005	5.5024		Flavonoids	+	+	+
15	$[M+H]^+$	$C_{21}H_{20}O_{12}$	Hyperoside	465.1028	465.1025	-0.0003	4.8035	482-36-0	Flavonoids	+	+	+
16	$[M-H]^-$	$C_{26}H_{28}O_{16}$	Quercetin 3-sambubioside	595.1305	595.1301	-0.0004	4.5635		Flavonoids	+	+	+
17	$[M+H]^+$	$C_{27}H_{30}O_{17}$	6-Hydroxykaempferol 3,6-diglucoside	627.1556	627.1553	-0.0003	4.2971		Flavonoids	+	+	+
18	$[M-H]^-$	$C_{15}H_{14}O_6$	Epicatechin	289.0718	289.0715	-0.0003	4.4136	490-46-0	Flavonoids	+	−	−
19	$[M-H-H_2O]^-$	$C_{15}H_{14}O_7$	Gallocatechin	287.0561	287.0559	-0.0002	5.5267	970-73-0	Flavonoids	+	+	+
20	$[M+H]^+$	$C_{15}H_{14}O_6$	Catechin	291.0863	291.0860	-0.0003	4.1455	154-23-4	Flavonoids	+	+	+
21	$[M+H]^+$	$C_{15}H_{14}O_7$	Epigallocatechin	307.0812	307.0809	-0.0003	3.9654	970-74-1	Flavonoids	+	+	+
22	$[M+H]^+$	$C_{22}H_{18}O_{10}$	Epicatechin gallate	443.0973	443.0969	-0.0004	4.8511	1257-08-5	Flavonoids	+	+	+
23	$[M+H]^+$	$C_{30}H_{26}O_{12}$	Procyanidin B4	579.1497	579.1493	-0.0004	4.2369	4852-22-6	Flavonoids	+	+	+
24	$[M+H]^+$	$C_{45}H_{38}O_{18}$	Procyanidin C1	867.2131	867.2125	-0.0006	4.3914	37064-30-5	Flavonoids	+	+	+
25	$[M+H]^+$	$C_8H_{10}O_2$	4-Hydroxyphenyl ethanol	139.0753	139.0753	0.0000	4.0700	501-94-0	Phenols	+	+	+
26	$[M+H]^+$	$C_7H_6O_2$	3-Hydroxybenzaldehyde	123.0441	123.0442	0.0001	4.6930	100-83-4	Phenols	+	+	+
27	$[M+H]^+$	$C_8H_8O_3$	Isovanillin	153.0546	153.0546	0.0000	4.9644		Phenols	+	+	+
28	$[M+H]^+$	$C_9H_{10}O_4$	Syringaldehyde	183.0652	183.0651	-0.0001	4.9397		Phenols	+	+	+
29	$[M-H]^-$	$C_{11}H_8O_5$	Purpurogallin	219.0299	219.0294	-0.0005	5.9155	569-77-7	Phenols	+	+	+

	Adducts	Molecular formula	component name	theoretical m/z	*m/z*	Mass Error (ppm)	*t*R (min)	CAS	classification	RC	RK	RR
30	$[M+H]^+$	$C_7H_6O_5$	Gallic acid	171.0287	171.0287	0.0000	1.8100	149-91-7	Benzene and substituted derivatives	+	+	+
31	$[M+H]^+$	$C_7H_6O_3$	3,4-Dihydroxybenzaldehyde	139.0390	139.0389	-0.0001	4.0962	139-85-5	Phenols	+	+	+
32	$[M-H]^-$	$C_{13}H_8O_7$	3,4,8,9,10-Pentahydroxy Urolithin	275.0197	275.0196	-0.0001	4.5908	91485-02-8	Phenols	+	+	+
33	$[M-H]^-$	$C_{15}H_{22}O_5$	Octyl gallate	281.1394	281.1393	-0.0001	9.9084	1034-01-1	Phenols	+	+	+
34	$[M-H]^-$	$C_{15}H_8O_7$	Demethylwedelolactone	299.0197	299.0196	-0.0001	4.9967		Phenols	+	+	+
35	$[M+H]^+$	$C_{14}H_{16}O_{10}$	3-Galloylquinic acid	345.0816	345.0813	-0.0003	1.7646	17365-11-6	Phenols	+	+	+
36	$[M+H]^+$	$C_{20}H_{28}O_{10}$	rosavin	429.1744	429.1744	0.0000	4.9200		Phenylpropanoids	+	+	+
37	$[M-H]^-$	$C_9H_{10}O_3$	L-3-Phenyllactic acid	165.0557	165.0549	-0.0008	4.9830	20312-36-1	Phenylpropanoids	+	+	+
38	$[M-H]^-$	$C_9H_{10}O_4$	Hydroxyphenyllactic acid	181.0506	181.0498	-0.0008	3.8506	306-23-0	Phenylpropanoids	+	+	+
39	$[M-H]^-$	$C_{21}H_{24}O_9$	Isorhapontin	419.1348	419.1347	-0.0001	5.0511		Phenylpropanoids	+	+	+
40	$[M+HCOO]^-$	$C_{20}H_{22}O_8$	Polydatin	435.1291	435.1293	0.0002	4.7912	27208-80-6	Phenylpropanoids	+	+	+
41	$[M+H-H_2O]^+$	$C_9H_{10}O_2$	Cinnamyl Alcohol	117.0699	117.0701	0.0002	6.4052	104-54-1	Phenylpropanoids	+	+	+
42	$[M-H]^-$	$C_{10}H_{10}O_3$	Methyl p-coumarate	177.0557	177.0549	-0.0008	6.5926	3943-97-3	Phenylpropanoids	+	+	+
43	$[M+H]^+$	$C_{10}H_{10}O_3$	Coniferaldehyde	179.0703	179.0702	-0.0001	5.5569	20649-42-7	Phenylpropanoids	+	+	+
44	$[M+H]^+$	$C_{11}H_{12}O_4$	Ferulic acid methyl ester	209.0808	209.0807	-0.0001	6.8330		Phenylpropanoids	+	+	+
45	$[M+H]^+$	$C_{20}H_{20}O_6$	(+)-Balanophonin	357.1333	357.1329	-0.0004	6.4611	215319-47-4	Phenylpropanoids	+	+	+
46	$[M-H]^-$	$C_{26}H_{32}O_{11}$	Pinoresinol 4-*O*-β-D-glucopyranoside	519.1872	519.1869	-0.0003	5.0241	69251-96-3	Phenylpropanoids	+	+	+
47	$[M+H]^+$	$C_{15}H_8O_7$	Isodemethylwedelolactone	301.0343	301.0340	-0.0003	5.2805	350681-33-3	Phenylpropanoids	+	−	+
48	$[M+NH_4]^+$	$C_{10}H_8O_3$	6-hydroxy-4-methylcoumarin	194.0812	194.0811	-0.0001	5.0676		Phenylpropanoids	−	+	+
49	$[M+H]^+$	$C_9H_8O_2$	Dihydrocoumarin	149.0597	149.0597	0.0000	5.3489	119-84-6	Phenylpropanoids	+	+	+
50	$[M+H-H_2O]^+$	$C_{20}H_{22}O_6$	Pinoresinol	341.1383	341.1381	-0.0002	6.4749	487-36-5	Phenylpropanoids	+	+	+
51	$[M+HCOO]^-$	$C_{20}H_{24}O_7$	Cycloolivil	421.1499	421.1503	0.0004	4.9278		Phenylpropanoids	+	+	+
52	$[M-H]^-$	$C_{26}H_{32}O_{11}$	Matairesinoside	519.1872	519.1869	-0.0003	5.3409		Phenylpropanoids	+	+	+
53	$[M+H]^+$	$C_7H_{12}N_2O_4$	Aceglutamide	189.0870	189.0869	-0.0001	1.1141	2490-97-3	Amino Acids, Peptides and derivatives	−	+	−
54	$[M+H]^+$	$C_4H_9NO_2$	gamma-Aminobutyric acid	104.0706	104.0709	0.0003	0.7825	56-12-2	Amino Acids, Peptides and derivatives	+	+	+
55	$[M+H]^+$	$C_5H_9NO_2$	L-Proline	116.0706	116.0708	0.0002	0.8046	147-85-3	Amino Acids, Peptides and derivatives	+	+	+
56	$[M+H]^+$	$C_5H_7NO_3$	Pyroglutamic acid	130.0499	130.0499	0.0000	1.1541	98-79-3	Amino Acids, Peptides and derivatives	+	+	+

	Adducts	Molecular formula	component name	theoretical m/z	*m/z*	Mass Error (ppm)	*t*R (min)	CAS	classification	RC	RK	RR
57	$[M+H]^+$	$C_6H_{11}NO_2$	L-Pipecolic acid	130.0863	130.0863	0.0000	1.0519	3105-95-1	Amino Acids, Peptides and derivatives	+	+	+
58	$[M+H]^+$	$C_6H_{13}NO_2$	L-Leucine	132.1019	132.1019	0.0000	1.4905	61-90-5	Amino Acids, Peptides and derivatives	+	+	+
59	$[M+H]^+$	$C_5H_{10}N_2O_3$	L-Glutamine	147.0764	147.0763	-0.0001	0.7914	56-85-9	Amino Acids, Peptides and derivatives	+	+	+
60	$[M+H]^+$	$C_5H_9NO_4$	L-Glutamic acid	148.0604	148.0603	-0.0001	0.7761	56-86-0	Amino Acids, Peptides and derivatives	+	+	+
61	$[M+H]^+$	$C_7H_{13}NO_3$	N-Acetylvaline	160.0968	160.0967	-0.0001	3.9325	96-81-1	Amino Acids, Peptides and derivatives	+	+	+
62	$[M+H]^+$	$C_9H_{11}NO_2$	L-Phenylalanine	166.0863	166.0862	-0.0001	2.3593	63-91-2	Amino Acids, Peptides and derivatives	+	+	+
63	$[M+H]^+$	$C_6H_{14}N_4O_2$	L-Arginine	175.1190	175.1189	-0.0001	0.7444	74-79-3	Amino Acids, Peptides and derivatives	+	+	+
64	$[M-H]^-$	$C_9H_{11}NO_3$	L-Tyrosine	180.0666	180.0658	-0.0008	1.2578	60-18-4	Amino Acids, Peptides and derivatives	+	+	+
65	$[M+H]^+$	$C_{10}H_{13}NO_4$	3-Methoxytyrosine	212.0917	212.0917	0.0000	1.6820	7636-26-2	Amino Acids, Peptides and derivatives	+	+	+
66	$[M-H]^-$	$C_{12}H_{23}NO_7$	N-(1-Deoxy-1-fructosyl)leucine	292.1402	292.1400	-0.0002	1.4683	34393-18-5	Amino Acids, Peptides and derivatives	+	+	+
67	$[M+H]^+$	$C_{15}H_{21}NO_7$	N-(1-Deoxy-1-fructosyl)phenylala nine	328.1391	328.1388	-0.0003	2.3593	87251-83-0	Amino Acids, Peptides and derivatives	+	+	+
68	$[M+H]^+$	$C_{15}H_{21}NO_8$	N-(1-Deoxy-1-fructosyl)tyrosine	344.134	344.1341	0.0001	1.0913	34393-22-1	Amino Acids, Peptides and derivatives	+	+	+
69	$[M+H]^+$	$C_{11}H_{20}N_2O_5$	gamma-Glutamylleucine	261.1445	261.1443	-0.0002	3.8814	2566-39-4	Amino Acids, Peptides and derivatives	−	+	+
70	$[M-H]^-$	$C_8H_{15}NO_3$	N-Acetylleucine	172.0979	172.0971	-0.0008	4.5772	1188-21-2	Amino Acids, Peptides and derivatives	+	+	+
71	$[M+H]^+$	$C_8H_{16}N_2O_3$	Glycyl-Isoleucine	189.1234	189.1233	-0.0001	2.7139		Amino Acids, Peptides and derivatives	+	+	+
72	$[M+H]^+$	$C_7H_{11}NO_5$	N-Acetyl-L-glutamic acid	190.071	190.0709	-0.0001	1.2178	1188-37-0	Amino Acids, Peptides and derivatives	+	+	+
73	$[M+H]^+$	$C_8H_{16}N_2O_4$	Serylvaline	205.1183	205.1182	-0.0001	1.2362	51782-06-0	Amino Acids, Peptides and derivatives	+	+	+
74	$[M-H]^-$	$C_{11}H_{13}NO_3$	N-Acetyl-L-phenylalanine	206.0823	206.0816	-0.0007	4.8042	2018-61-3	Amino Acids, Peptides and derivatives	+	+	+
75	$[M+H]^+$	$C_8H_{16}N_4O_3$	N-Acetylarginine	217.1295	217.1295	0.0000	1.0519	155-84-0	Amino Acids, Peptides and derivatives	+	+	+
76	$[M-H]^-$	$C_9H_{17}NO_5$	Pantothenic acid	218.1034	218.1028	-0.0006	3.0035	79-83-4	Amino Acids, Peptides and derivatives	+	+	+
77	$[M+H]^+$	$C_{10}H_{19}N_3O_4$	Asparaginyl-Leucine	246.1448	246.1447	-0.0001	2.8025		Amino Acids, Peptides and	+	+	+

	Adducts	Molecular formula	component name	theoretical m/z	*m/z*	Mass Error (ppm)	*t*R (min)	CAS	classification	RC	RK	RR
									derivatives			
78	$[M+H]^+$	$C_{10}H_{18}N_2O_5$	Aspartyl-Leucine	247.1288	247.1287	-0.0001	3.3091		Amino Acids, Peptides and derivatives	+	+	+
79	$[M+H]^+$	$C_{11}H_{20}N_2O_5$	Glutamylleucine	261.1445	261.1443	-0.0002	3.3865	5969-52-8	Amino Acids, Peptides and derivatives	+	+	+
80	$[M+H]^+$	$C_{11}H_{20}N_2O_5$	Isoleucyl-Glutamate	261.1445	261.1443	-0.0002	1.4954		Amino Acids, Peptides and derivatives	+	+	+
81	$[M+H]^+$	$C_{11}H_{19}N_3O_6$	Ophthalmic acid	290.1347	290.1343	-0.0004	1.1333	495-27-2	Amino Acids, Peptides and derivatives	+	+	+
82	$[M-H]^-$	$C_{20}H_{32}N_6O_{12}S_2$	Oxidized glutathione	611.1447	611.1442	-0.0005	1.1228	27025-41-8	Amino Acids, Peptides and derivatives	+	+	+
83	$[M-H]^-$	$C_{10}H_{12}N_4O_5$	Inosine	267.0735	267.0732	-0.0003	1.4254	58-63-9	Nucleotides and derivatives	+	+	+
84	$[M+H]^+$	$C_{10}H_{13}N_5O_4$	Adenosine	268.104	268.1038	-0.0002	1.3075	58-61-7	Nucleotides and derivatives	+	+	+
85	$[M+H]^+$	$C_{10}H_{13}N_5O_5$	Guanosine	284.0989	284.0987	-0.0002	1.4233	118-00-3	Nucleotides and derivatives	+	+	+
86	$[M+HCOO]^-$	$C_{10}H_{14}N_2O_5$	Thymidine	287.0885	287.0881	-0.0004	2.3297	50-89-5	Nucleotides and derivatives	+	+	+
87	$[M+H]^+$	$C_{10}H_{14}N_5O_8P$	Guanosine monophosphate	364.0653	364.0649	-0.0004	1.0519	85-32-5	Nucleotides and derivatives	+	+	+
88	$[M+H]^+$	$C_{14}H_{17}N_5O_8$	Succinyladenosine	384.1150	384.1146	-0.0004	3.3864	4542-23-8	Nucleotides and derivatives	+	+	+
89	$[M+HCOO]^-$	$C_{20}H_{26}O_{11}$	Regaloside B	487.1452	487.1453	0.0001	4.8403	114420-67-6	Carbohydrates and Glycosides	−	−	+
90	$[M-H]^-$	$C_{14}H_{18}O_8$	Glucovanillin	313.0929	313.0929	0.0000	4.0140	494-08-6	Carbohydrates and Glycosides	+	+	+
91	$[M-H]^-$	$C_{12}H_{16}O_7$	Arbutin	317.0879	317.0875	-0.0004	1.4423	497-76-7	Carbohydrates and Glycosides	+	+	+
92	$[M-H]^-$	$C_{14}H_{18}O_9$	Phaseoloidin	329.0878	329.0876	-0.0002	1.6523	118555-82-1	Carbohydrates and Glycosides	+	+	+
93	$[M-H]^-$	$C_{14}H_{18}O_9$	Vanillic acid 4- β -*D*-glucopyranoside	329.0878	329.0876	-0.0002	3.2929		Carbohydrates and Glycosides	+	+	+
94	$[M-H]^-$	$C_{15}H_{18}O_8$	4-*O*- β -Glucopyranosyl-cis-coumaric acid	371.0984	371.0979	-0.0005	3.7844		Carbohydrates and Glycosides	+	+	+
95	$[M+HCOO]^-$	$C_{16}H_{22}O_8$	Coniferin	387.1291	387.1292	0.0001	3.9686	124151-33-3	Carbohydrates and Glycosides	+	+	+
96	$[M+HCOO]^-$	$C_{16}H_{20}O_9$	Trans-ferulic acid-4- β -glucoside	401.1084	401.1085	0.0001	3.9686	537-98-4	Carbohydrates and Glycosides	+	+	+
97	$[M-H]^-$	$C_{17}H_{29}NO_{11}$	Neolinustatin	468.1723	468.1719	-0.0004	3.5671	72229-42-6	Carbohydrates and Glycosides	+	+	+
98	$[M+HCOO]^-$	$C_{14}H_{20}O_7$	Salidroside	345.1186	345.1186	0.0000	3.8506	10338-51-9	Carbohydrates and Glycosides	+	+	+
99	$[M-H]^-$	$C_6H_{10}O_6$	1,4-*D*-Gulonolactone	177.0405	177.0397	-0.0008	1.2038	3327-64-8	Carbohydrates and Glycosides	+	+	+
100	$[M+H]^+$	$C_6H_{13}NO_5$	Manosamine	180.0866	180.0865	-0.0001	0.7566		Carbohydrates and Glycosides	+	+	+
101	$[M-H]^-$	$C_6H_{14}O_6$	Galactitol	181.0718	181.0709	-0.0009	0.7544	608-66-2	Carbohydrates and Glycosides	+	+	+
102	$[M-H-H_2O]^-$	$C_7H_{14}O_7$	D-altrofurano-heptulose-3	191.0560	191.0552	-0.0008	1.2038	25545-06-6	Carbohydrates and Glycosides	+	+	+

	Adducts	Molecular formula	component name	theoretical m/z	*m/z*	Mass Error (ppm)	*t*R (min)	CAS	classification	RC	RK	RR
103	[M−H]⁻	$C_6H_{10}O_7$	Galacturonic acid	193.0354	193.0346	-0.0008	0.7911		Carbohydrates and Glycosides	+	+	+
104	[M+HCOO]⁻	$C_5H_8O_5$	Ribonolactone	193.0348	193.0347	-0.0001	0.5070	5336-08-3	Carbohydrates and Glycosides	+	+	+
105	[M−H]⁻	$C_6H_{12}O_7$	Gluconic acid	195.0510	195.0503	-0.0007	0.7698	526-95-4	Carbohydrates and Glycosides	+	+	+
106	[M+H]⁺	$C_8H_{15}NO_6$	N-Acetyl-D-glucosamine	204.0866	204.0866	0.0000	0.8046	7512-17-6	Carbohydrates and Glycosides	+	+	+
107	[M+HCOO]⁻	$C_6H_{12}O_6$	Glucose	225.0610	225.0610	0.0000	0.7911	921-60-8	Carbohydrates and Glycosides	+	+	+
108	[M−H]⁻	$C_9H_{12}N_2O_6$	1-β-*D*-Arabinofuranosyluracil	243.0623	243.0619	-0.0004	1.1772		Carbohydrates and Glycosides	+	+	+
109	[M+H]⁺	$C_9H_{13}N_3O_5$	Cytarabine	244.0928	244.0927	-0.0001	0.9684	147-94-4	Carbohydrates and Glycosides	+	+	+
110	[M−H]⁻	$C_6H_{13}O_9P$	Galactose 1-phosphate	259.0224	259.0221	-0.0003	0.7295	2255-14-3	Carbohydrates and Glycosides	+	+	+
111	[M+H−H₂O]⁺	$C_{12}H_{22}O_{11}$	Trehalose	325.1129	325.1127	-0.0002	0.7868	99-20-7	Carbohydrates and Glycosides	+	+	+
112	[M−H]⁻	$C_{12}H_{18}O_{11}$	2-*O*-β-*D*-Glucopyranosyl-L-ascorbic acid	337.0776	337.0774	-0.0002	1.0827		Carbohydrates and Glycosides	+	+	+
113	[M+H]⁺	$C_{12}H_{23}NO_{10}$	6-(α-D-Glucosaminyl)-1D-myo-inositol	342.1395	342.1391	-0.0004	0.7566		Carbohydrates and Glycosides	+	+	+
114	[M+H]⁺	$C_{12}H_{22}O_{11}$	Sucrose	365.1055	365.1051	-0.0004	0.8224	57-50-1	Carbohydrates and Glycosides	+	+	+
115	[M−H]⁻	$C_{12}H_{22}O_{11}$	α-Lactose	387.1145	387.1141	-0.0004	0.7818	63-42-3	Carbohydrates and Glycosides	+	+	+
116	[M+HCOO]⁻	$C_{21}H_{36}O_{10}$	A-*D*-Glucopyranoside	493.2291	493.2288	-0.0003	5.9892	88700-35-0	Carbohydrates and Glycosides	+	+	+
117	[M−H]⁻	$C_{18}H_{32}O_{16}$	Maltotriose	549.1673	549.1670	-0.0003	0.8005		Carbohydrates and Glycosides	+	+	+
118	[M+HCOO]⁻	$C_{24}H_{42}O_{21}$	Stachyose	711.2195	711.2201	0.0006	0.7818	470-55-3	Carbohydrates and Glycosides	+	+	+
119	[M+H−H₂O]⁺	$C_{15}H_{26}O$	Patchouli alcohol	205.1951	205.1950	-0.0001	11.7718	5986-55-0	Terpenes	+	+	+
120	[M+H−H₂O]⁺	$C_{15}H_{26}O$	β-Eudesmol	205.1951	205.1950	-0.0001	11.2255	473-15-4	Terpenes	+	+	+
121	[M+H−H₂O]⁺	$C_{15}H_{20}O_3$	Micheliolide	231.138	231.1379	-0.0001	8.5007	68370-47-8	Terpenes	+	+	+
122	[M+H−H₂O]⁺	$C_{15}H_{20}O_3$	1β-Hydroxyalantolactone	231.138	231.1379	-0.0001	7.4445	68776-47-6	Terpenes	+	+	+
123	[M+H]⁺	$C_{15}H_{20}O_3$	Atractylenolide III	249.1485	249.1483	-0.0002	9.4561		Terpenes	+	+	+
124	[M+H]⁺	$C_{19}H_{26}O_7$	Diacetoxyscirpenol	367.1751	367.1753	0.0002	6.8796	2270-40-8	Terpenes	+	+	+
125	[M+H−H₂O]⁺	$C_{10}H_{18}O$	Geraniol	137.1325	137.1324	-0.0001	9.2707	106-24-1	Terpenes	+	+	+
126	[M+NH₄]⁺	$C_{16}H_{28}O_7$	Rosiridin	350.2173	350.2170	-0.0003	4.9888		Terpenes	+	+	+
127	[M−H−H₂O]⁻	$C_{16}H_{22}O_{10}$	Secologanic acid	355.1034	355.1032	-0.0002	3.9686	60077-46-5	Terpenes	+	+	+

	Adducts	Molecular formula	component name	theoretical m/z	*m/z*	Mass Error (ppm)	*t*R (min)	CAS	classification	RC	RK	RR
128	$[M-H]^-$	$C_{11}H_{20}O_6$	Crenulatin	247.1187	247.1183	-0.0004	4.2455	63026-02-8	Terpenes	+	+	+
129	$[M+H]^+$	$C_{11}H_{16}O_2$	Dihydroactinidiolide	181.1223	181.1222	-0.0001	8.1254	15356-74-8	Terpenes	+	+	+
130	$[M+H]^+$	$C_9H_8O_4$	Caffeic acid	181.0495	181.0494	-0.0001	4.0486	501-16-6	Organic acids and derivatives	+	+	+
131	$[M-H]^-$	$C_{10}H_{10}O_4$	Ferulic acid	193.0506	193.0499	-0.0007	5.0374	537-98-4	Organic acids and derivatives	+	+	+
132	$[M+H]^+$	$C_9H_8O_3$	*p*-Coumaric acid	165.0546	165.0545	-0.0001	4.8630	4501-31-9	Organic acids and derivatives	+	+	+
133	$[M+H-H_2O]^+$	$C_7H_{12}O_6$	Quinic acid	175.0601	175.0600	-0.0001	0.7957	77-95-2	Organic acids and derivatives	+	+	+
134	$[M+H]^+$	$C_6H_8O_6$	Ascorbic acid	177.0394	177.0393	-0.0001	1.0906	50-81-7	Organic acids and derivatives	+	+	+
135	$[M-H]^-$	$C_6H_8O_7$	Citric acid	191.0197	191.0189	-0.0008	1.1350	77-92-9	Organic acids and derivatives	+	+	+
136	$[M-H]^-$	$C_7H_{10}O_7$	2-Methylcitric acid	205.0354	205.0347	-0.0007	1.6382	6061-96-7	Organic acids and derivatives	+	+	+
137	$[M+H]^+$	$C_{10}H_7NO_4$	Xanthurenic acid	206.0448	206.0447	-0.0001	3.5051	59-00-7	Organic acids and derivatives	+	+	+
138	$[M+NH_4]^+$	$C_6H_8O_7$	Isocitric acid	210.0608	210.0608	0.0000	0.9925	320-77-4	Organic acids and derivatives	+	+	+
139	$[M-H]^-$	$C_{10}H_{18}O_5$	3-Hydroxysebacic acid	217.1081	217.1076	-0.0005	4.9682	446881-43-2	Organic acids and derivatives	+	+	+
140	$[M+H-H_2O]^+$	$C_{15}H_{20}O_4$	Abscisic acid	247.1329	247.1327	-0.0002	6.0735	21293-29-8	Organic acids and derivatives	+	+	+
141	$[M+H-H_2O]^+$	$C_{10}H_{18}O_2$	Decenoic acid	153.1274	153.1273	-0.0001	6.1641	14436-32-9	Fatty Acyls	+	+	+
142	$[M-H]^-$	$C_8H_{14}O_4$	Suberic acid	173.0819	173.0811	-0.0008	4.8448	505-48-6	Fatty Acyls	+	+	+
143	$[M+H-H_2O]^+$	$C_{10}H_{18}O_4$	Sebacic acid	185.1172	185.1174	0.0002	6.0374	111-20-6	Fatty Acyls	+	+	+
144	$[M-H]^-$	$C_9H_{16}O_4$	Azelaic acid	187.0976	187.0968	-0.0008	5.3677	123-99-9	Fatty Acyls	+	+	+
145	$[M+H-H_2O]^+$	$C_{11}H_{20}O_4$	Undecanedioic acid	217.1434	217.1434	0.0000	6.8330	1852-04-6	Fatty Acyls	+	+	+
146	$[M-H]^-$	$C_{12}H_{22}O_4$	Dodecanedioic acid	229.1445	229.1441	-0.0004	7.6844	693-23-2	Fatty Acyls	+	+	+
147	$[M+H]^+$	$C_{18}H_{30}O_2$	Octadecatrienoic acid	279.2319	279.2316	-0.0003	10.8136	544-72-9	Fatty Acyls	+	+	+
148	$[M+H]^+$	$C_{18}H_{35}NO$	Oleamide	282.2791	282.2789	-0.0002	12.8542	301-02-0	Fatty Acyls	+	+	+
149	$[M-H]^-$	$C_{16}H_{30}O_4$	Hexadecanedioic acid	285.2071	285.2069	-0.0002	10.2988	505-54-4	Fatty Acyls	+	+	+
150	$[M-H]^-$	$C_{18}H_{34}O_4$	Octadecanedioic acid	313.2384	313.2382	-0.0002	11.0602	871-70-5	Fatty Acyls	+	+	+
151	$[M-H]^-$	$C_{18}H_{34}O_4$	12,13-DHOME	313.2384	313.2383	-0.0001	9.9685	7293-40-5	Fatty Acyls	+	+	+
152	$[M+H]^+$	$C_{20}H_{37}NO_2$	Linoleoyl ethanolamide	324.2897	324.2895	-0.0002	11.6624	68171-52-8	Fatty Acyls	+	+	+
153	$[M+H]^+$	$C_{22}H_{43}NO$	Docosenamide	338.3417	338.3413	-0.0004	15.1987		Fatty Acyls	+	+	+
154	$[M+H]^+$	$C_6H_{11}NO_2$	Hygric acid	130.0863	130.0863	0.0000	0.8981	475-11-6	Alkaloids	+	+	+
155	$[M-H]^-$	$C_{10}H_{16}O_4$	Camphoric acid	199.0976	199.0969	-0.0007	5.3499	560-09-8	Carboxylic acid and derivatives	+	+	+
156	$[M+H]^+$	C_9H_7NO	4-formyl Indole	146.06	146.0600	0.0000	5.4485		Indoles and derivatives	+	+	+
157	$[M-H]^-$	$C_{11}H_{12}N_2O_2$	L-Tryptophan	203.0826	203.0819	-0.0007	3.7844	73-22-3	Indoles and derivatives	+	+	+
158	$[M+NH_4]^+$	$C_{10}H_9NO_3$	5-Hydroxyindoleacetic acid	209.0921	209.0920	-0.0001	3.7809	54-16-0	Indoles and derivatives	+	+	+
159	$[M-H]^-$	$C_{11}H_{12}N_2O_3$	5-Hydroxy-L-tryptophan	219.0775	219.0769	-0.0006	1.6600	4350-09-8	Indoles and derivatives	+	+	+

	Adducts	Molecular formula	component name	theoretical m/z	*m/z*	Mass Error (ppm)	*t*R (min)	CAS	classification	RC	RK	RR
160	$[M+H]^+$	$C_4H_5N_3O$	Cytosine	112.0505	112.0508	0.0003	0.9534	71-30-7	Organoheterocyclic compounds	+	+	+
161	$[M+H]^+$	$C_4H_4N_2O_2$	Uracil	113.0346	113.0348	0.0002	1.1709	66-22-8	Organoheterocyclic compounds	+	+	+
162	$[M+H-H_2O]^+$	$C_5H_7N_5O$	FAPy-adenine	136.0618	136.0618	0.0000	1.0020	5122-36-1	Organoheterocyclic compounds	+	+	+
163	$[M+H]^+$	$C_{29}H_{48}O$	Stigmasterol	413.3778	413.3773	-0.0005	14.7620		Steroids	+	+	−
164	$[M+H]^+$	$C_{15}H_{14}O_3$	Lapachol	243.1016	243.1015	-0.0001	10.1593	84-79-7	Quinones	−	+	+
165	$[M+H-H_2O]^+$	$C_{18}H_{37}NO_3$	Dehydrophytosphingosine	298.2740	298.2738	-0.0002	9.3151	3687-54-5	Sphingolipids	+	+	+
166	$[M+H]^+$	$C_{18}H_{39}NO_3$	Phytosphingosine	318.3003	318.3000	-0.0003	9.4364	554-62-1	Sphingolipids	+	+	+
167	$[M+H-H_2O]^+$	$C_7H_8O_2$	3-Hydroxybenzyl alcohol	107.0492	107.0494	0.0002	3.4797	620-24-6	Others	+	+	+
168	$[M]^+$	$C_5H_{15}NO_4P^+$	Phosphorylcholine	184.0739	184.0732	-0.0007	0.7666	3616-04-4	Others	+	+	+
169	$[M+H]^+$	$C_{12}H_{14}O_2$	Butylphthalide	191.1067	191.1067	0.0000	9.6613	3413-15-8	Others	+	+	+
170	$[M-H]^-$	$C_7H_{10}O_7$	Methyl citrate	205.0354	205.0347	-0.0007	1.9868	26163-61-1	Others	+	+	+
171	$[M+H-H_2O]^+$	$C_{13}H_{20}O_3$	Vomifoliol	207.1379	207.1379	0.0000	4.7550		Others	+	+	+
172	$[M+H]^+$	$C_{11}H_{12}O_4$	Methyl kakuol	209.0808	209.0808	0.0000	8.3905		Others	+	+	+
173	$[M-H]^-$	$C_{13}H_{24}O_4$	Tridecanedioic acid	243.1602	243.1597	-0.0005	8.5350	505-52-2	Others	+	+	+
174	$[M-H]^-$	$C_{10}H_{14}N_2O_6$	2′-O-Methyluridine	257.0779	257.0780	0.0001	1.9897	2140-76-3	Others	+	+	+
175	$[2M-H]^-$	$C_{11}H_8N_2O_3S_2$	Luciferin	558.9880	558.9875	-0.0005	5.5810	2591-17-5	Others	+	+	+

Abbreviations:

RC, *Rhodiola crenulata*; RK, *Rhodiola kirilowii*; RR, *Rhodiola rosea*.

"+" indicates detected; "−" indicates not detected.

tR, retention time.

Note: For unsaturated fatty acids and lipid-related metabolites, the exact positions of double bonds or oxidation sites could not be unambiguously determined due to the inherent limitations of LC–MS/MS analysis; therefore, these compounds were annotated at the level of molecular class or isomer without specification of double-bond or oxidation-site positions. In addition, the ion forms reported in this table correspond to the predominant ion species detected under the applied LC–MS conditions. In negative ion mode, some metabolites were detected as formate adducts ($[M+HCOO]^-$) due to the presence of formic acid in the mobile phase, while for certain labile compounds, ions corresponding to neutral losses (e.g., $-H_2O$) were observed as a result of ionization or in-source fragmentation behavior. The molecular formulas listed throughout the table refer to the intact neutral compounds and do not imply chemical modification of the metabolites.
