# Supplementary figures and images for: A “Qualitative–Pharmacological–Correlation–Molecular” Integrated Workflow Reveals HIF-1α–Relevant Anti-Hypoxia Metabolites in Rhodiola Species (part 2 of 2)
Source: Int J Mol Sci. 2026 Feb 26;27(5):2203. doi: 10.3390/ijms27052203 (PMC12984455; doi:10.3390/ijms27052203)

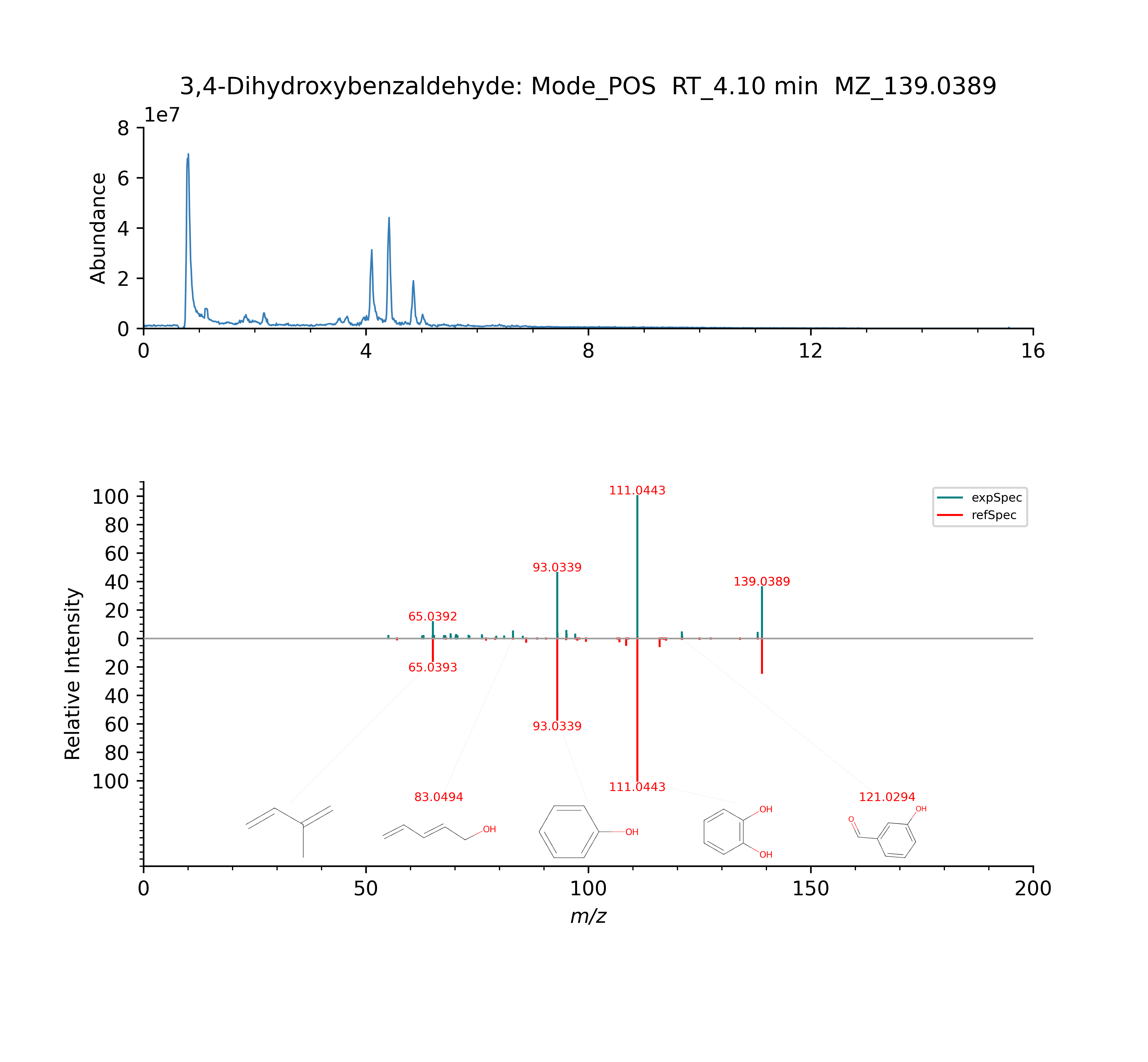

Supplement: Supplementary file 1 [file ijms-27-02203-s001.zip › ijms-4070482 Supplementary/Metabolite List Identified by LC-MS_MS from Rhodiola Species/31.png]

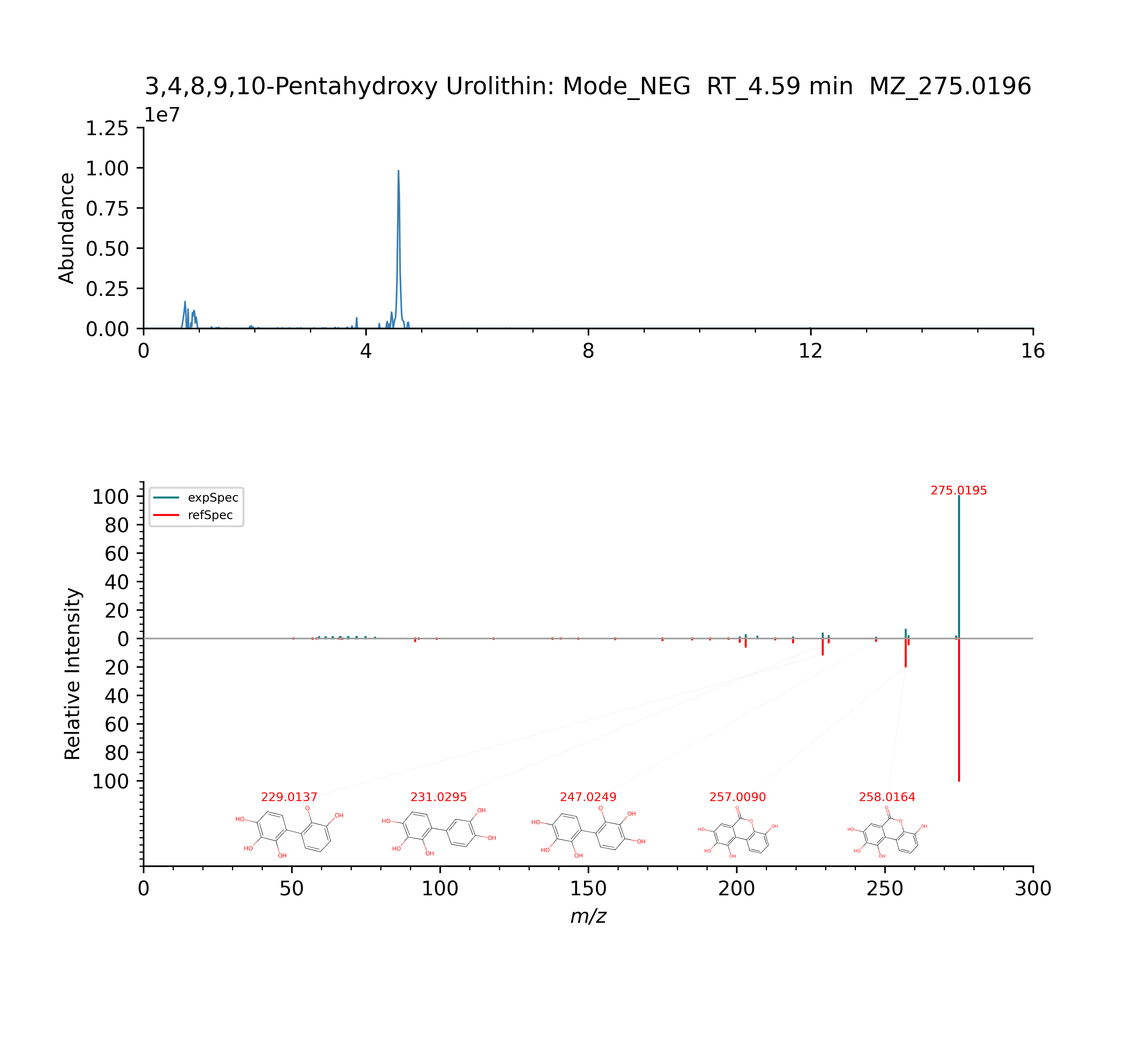

Supplement: Supplementary file 1 [file ijms-27-02203-s001.zip › ijms-4070482 Supplementary/Metabolite List Identified by LC-MS_MS from Rhodiola Species/32.png]

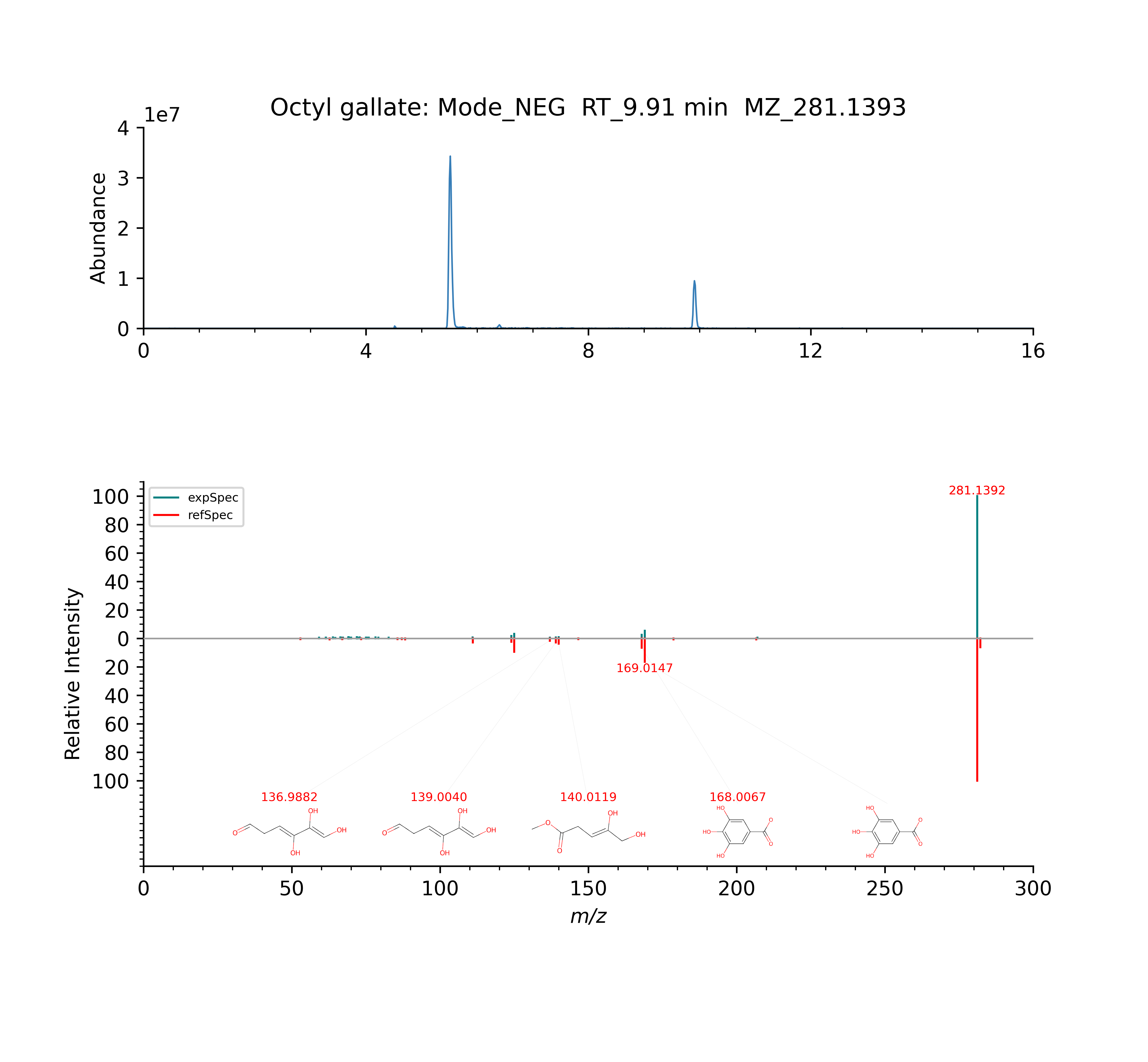

Supplement: Supplementary file 1 [file ijms-27-02203-s001.zip › ijms-4070482 Supplementary/Metabolite List Identified by LC-MS_MS from Rhodiola Species/33.png]

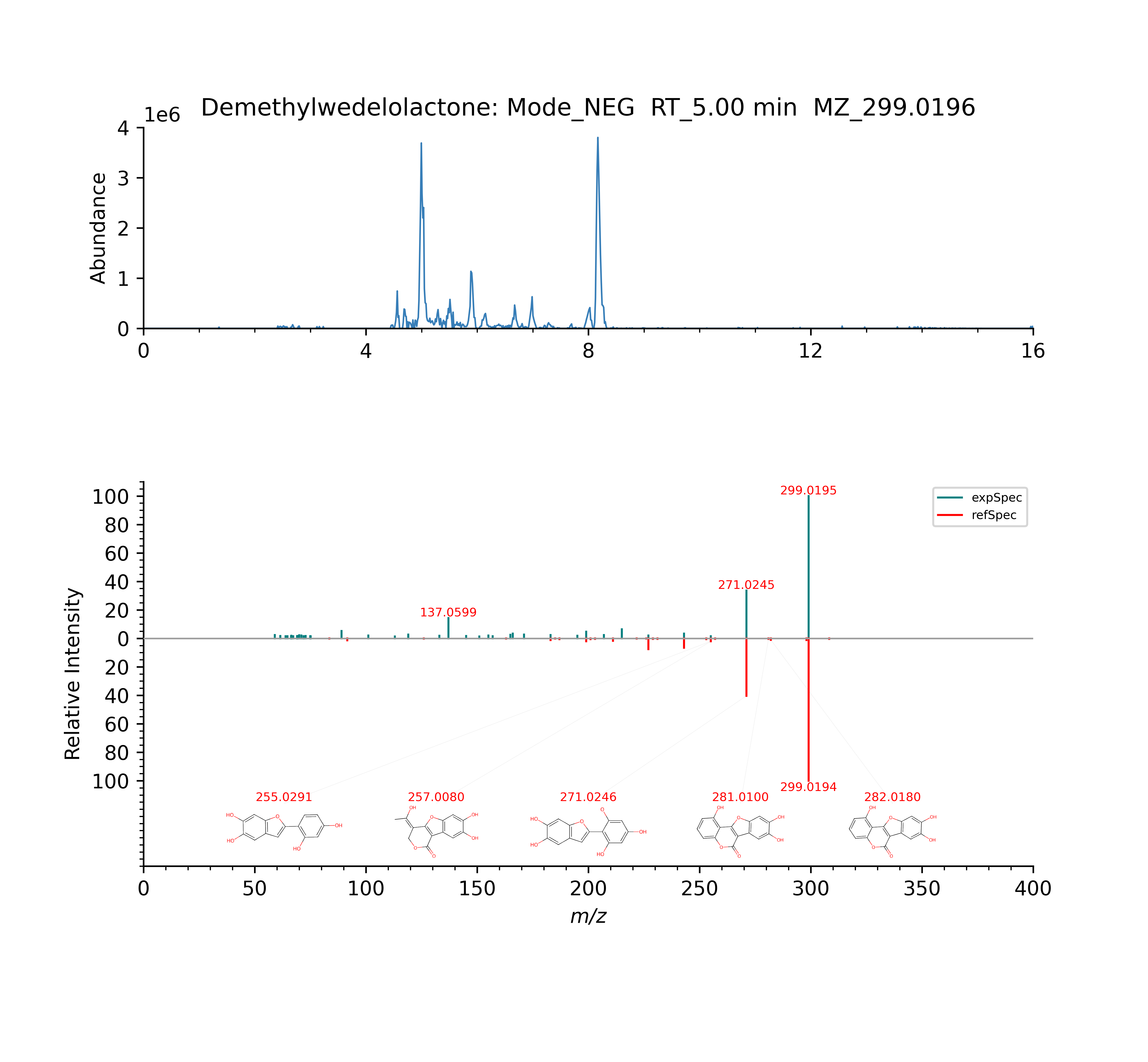

Supplement: Supplementary file 1 [file ijms-27-02203-s001.zip › ijms-4070482 Supplementary/Metabolite List Identified by LC-MS_MS from Rhodiola Species/34.png]

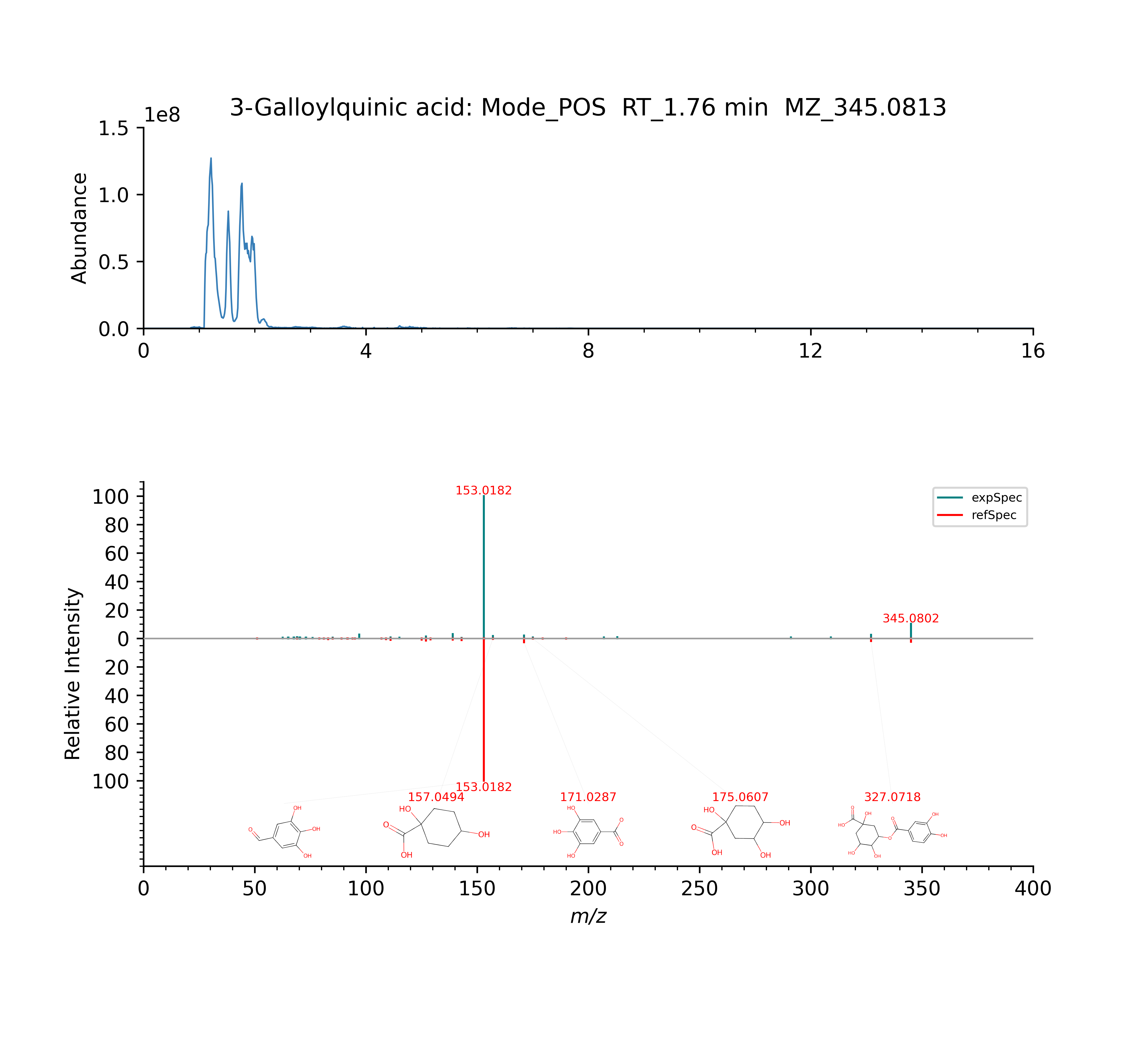

Supplement: Supplementary file 1 [file ijms-27-02203-s001.zip › ijms-4070482 Supplementary/Metabolite List Identified by LC-MS_MS from Rhodiola Species/35.png]

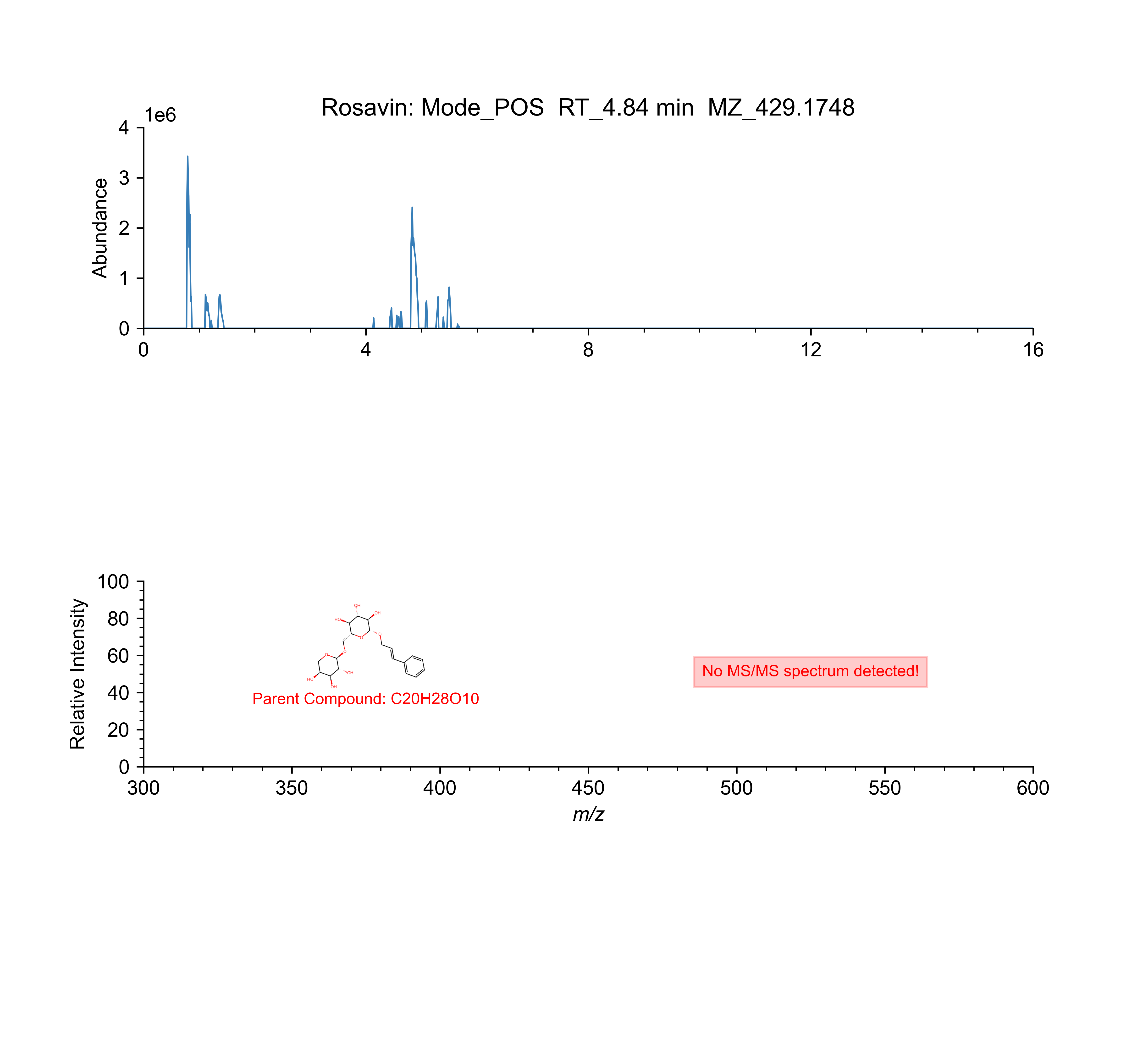

Supplement: Supplementary file 1 [file ijms-27-02203-s001.zip › ijms-4070482 Supplementary/Metabolite List Identified by LC-MS_MS from Rhodiola Species/36.png]

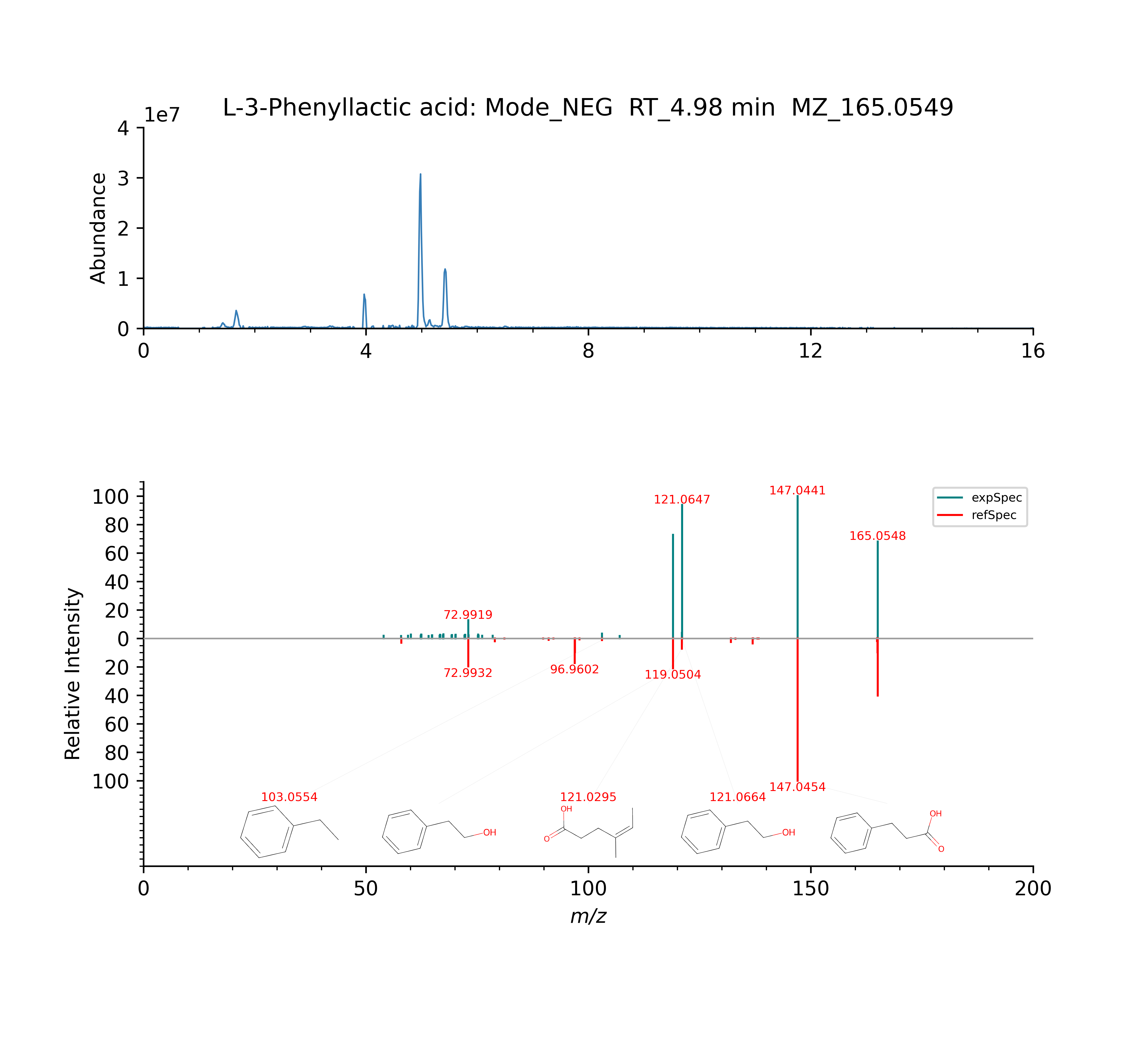

Supplement: Supplementary file 1 [file ijms-27-02203-s001.zip › ijms-4070482 Supplementary/Metabolite List Identified by LC-MS_MS from Rhodiola Species/37.png]

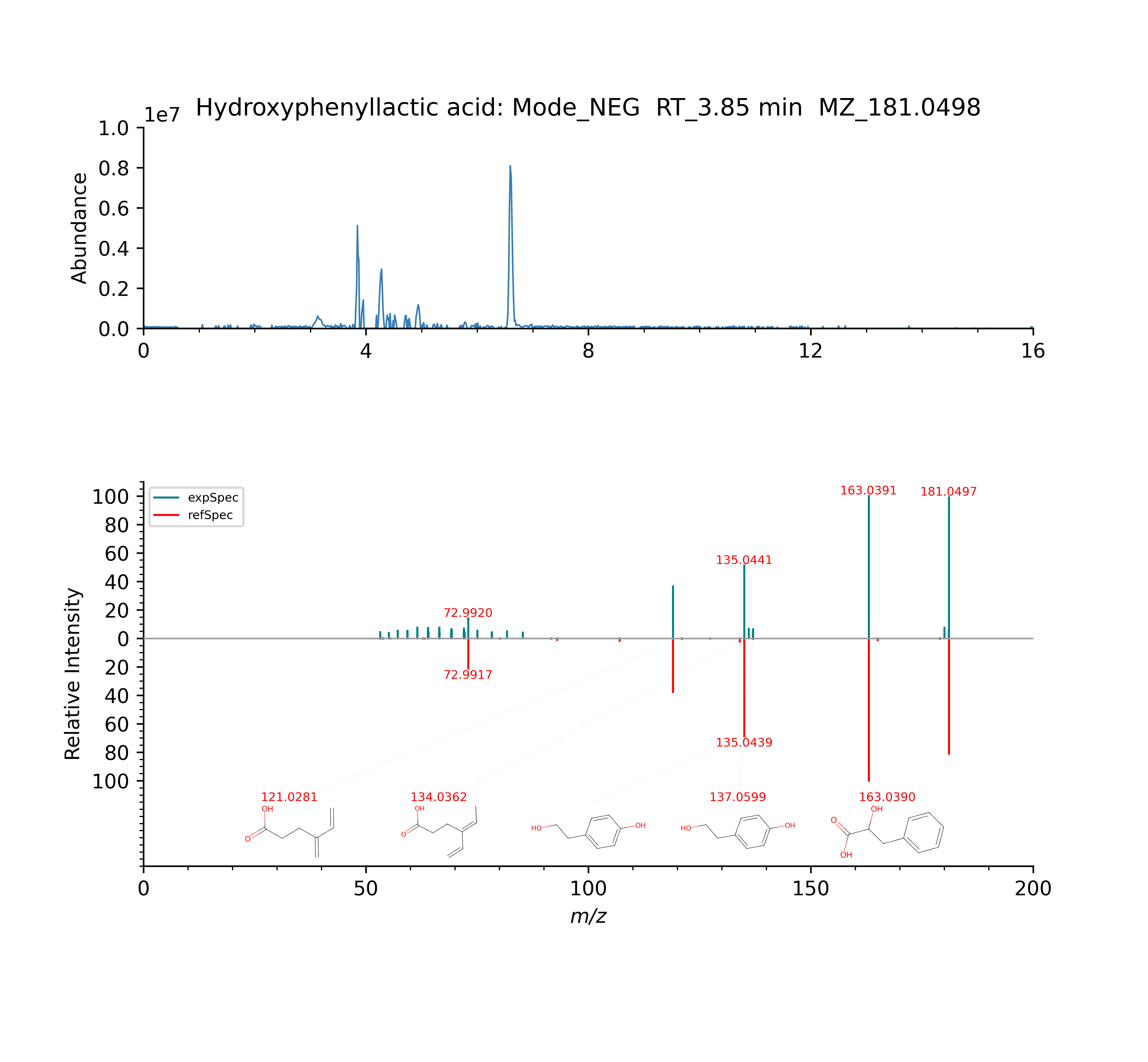

Supplement: Supplementary file 1 [file ijms-27-02203-s001.zip › ijms-4070482 Supplementary/Metabolite List Identified by LC-MS_MS from Rhodiola Species/38.png]

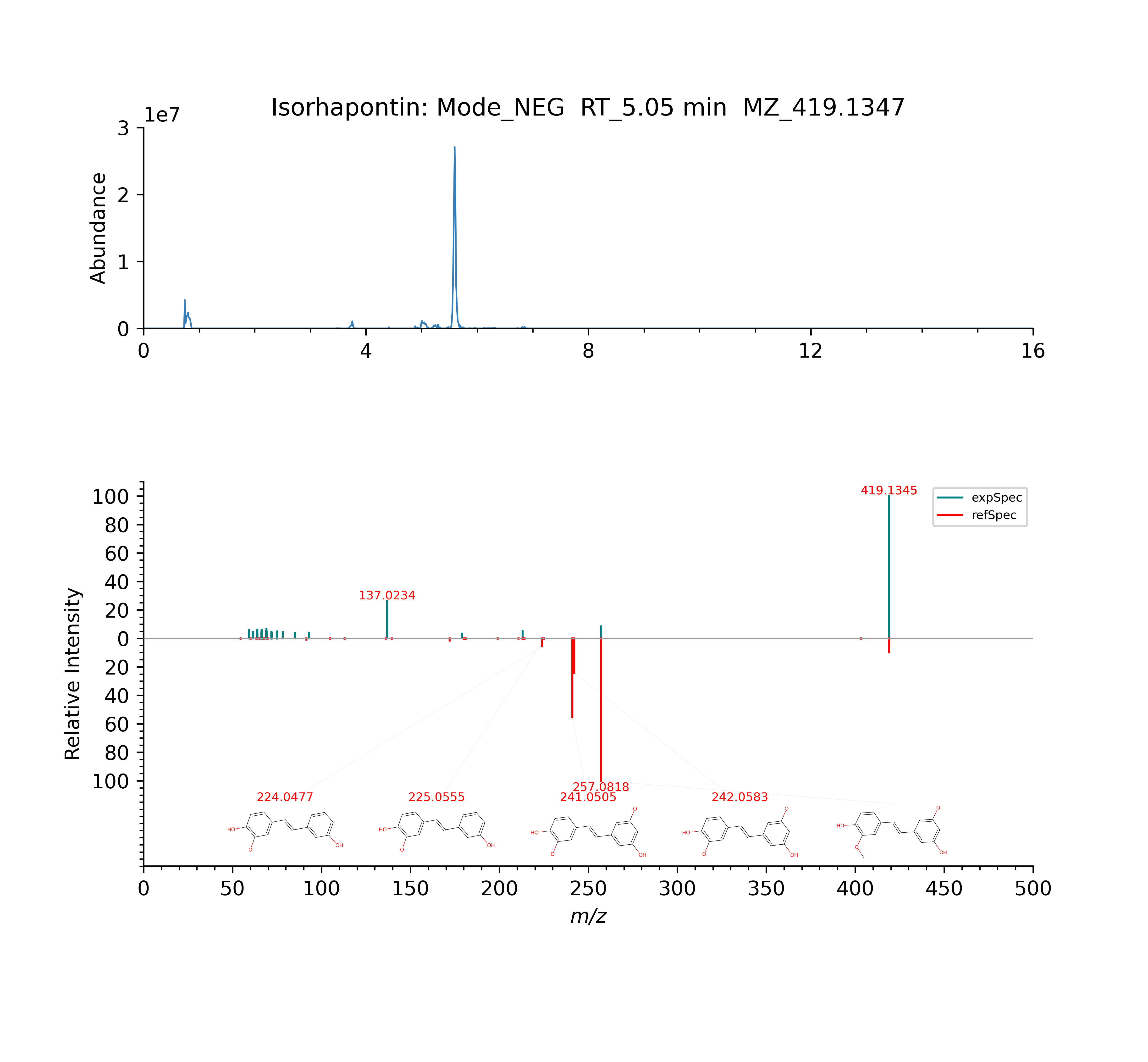

Supplement: Supplementary file 1 [file ijms-27-02203-s001.zip › ijms-4070482 Supplementary/Metabolite List Identified by LC-MS_MS from Rhodiola Species/39.png]

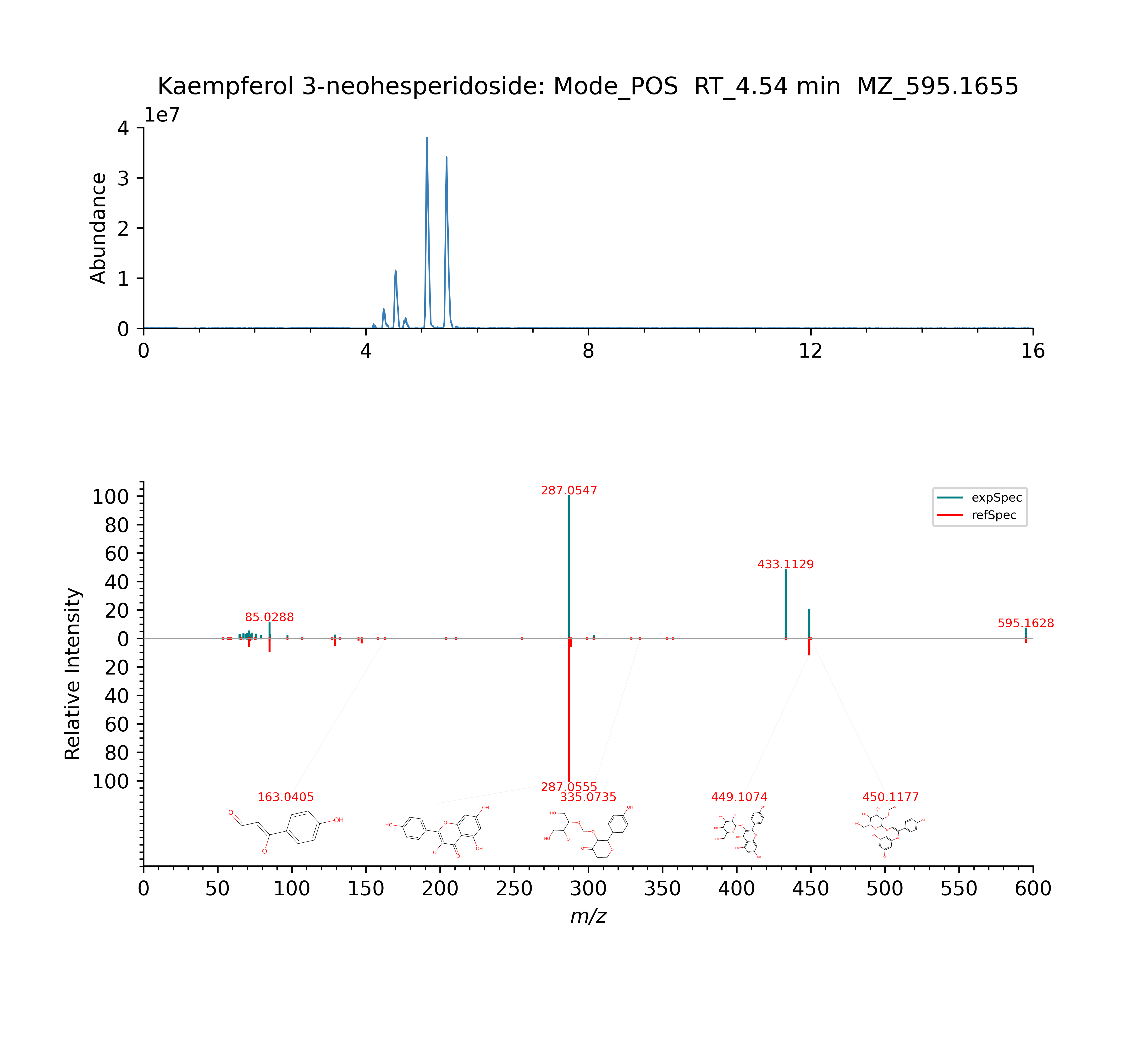

Supplement: Supplementary file 1 [file ijms-27-02203-s001.zip › ijms-4070482 Supplementary/Metabolite List Identified by LC-MS_MS from Rhodiola Species/4.png]

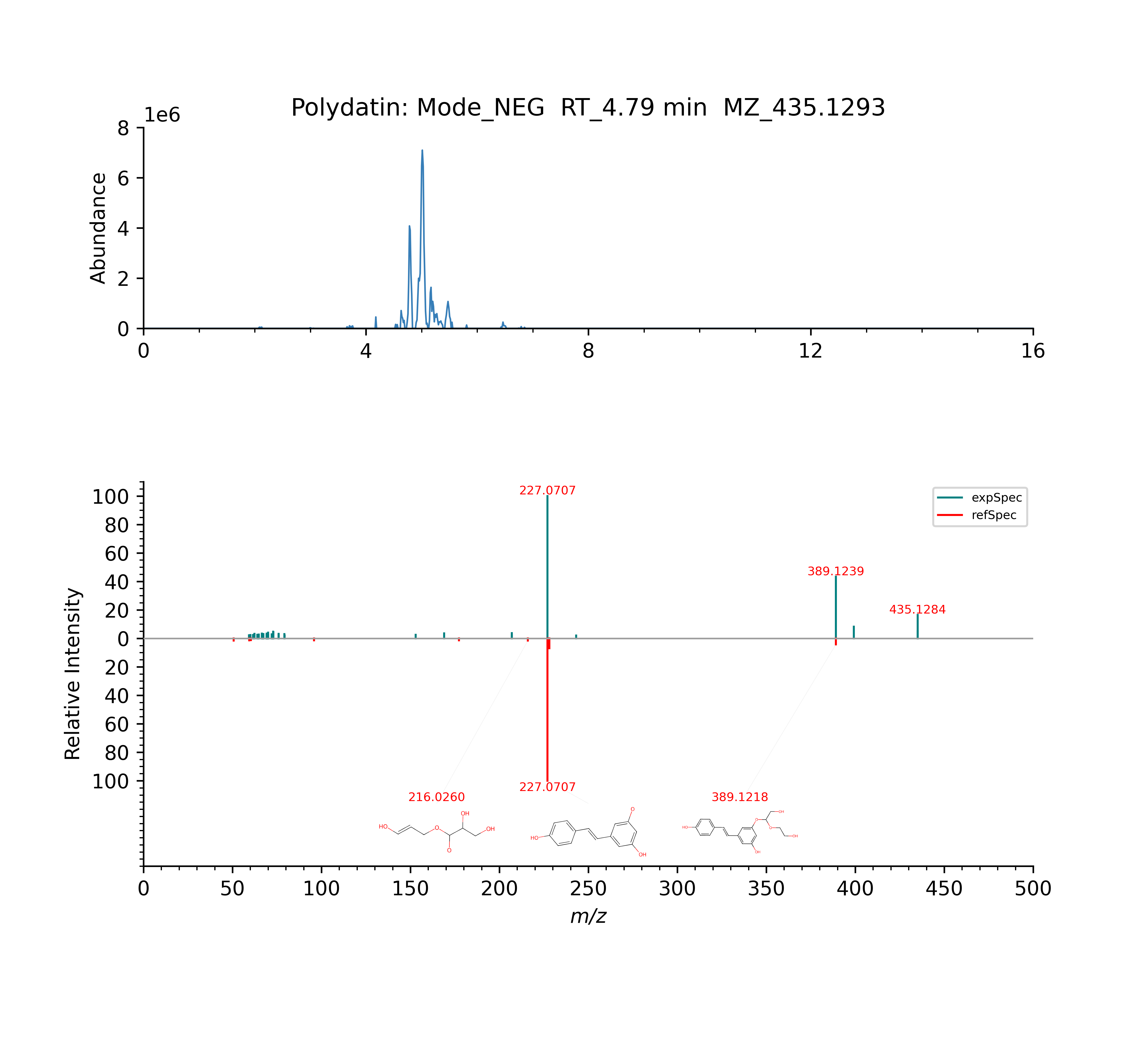

Supplement: Supplementary file 1 [file ijms-27-02203-s001.zip › ijms-4070482 Supplementary/Metabolite List Identified by LC-MS_MS from Rhodiola Species/40.png]

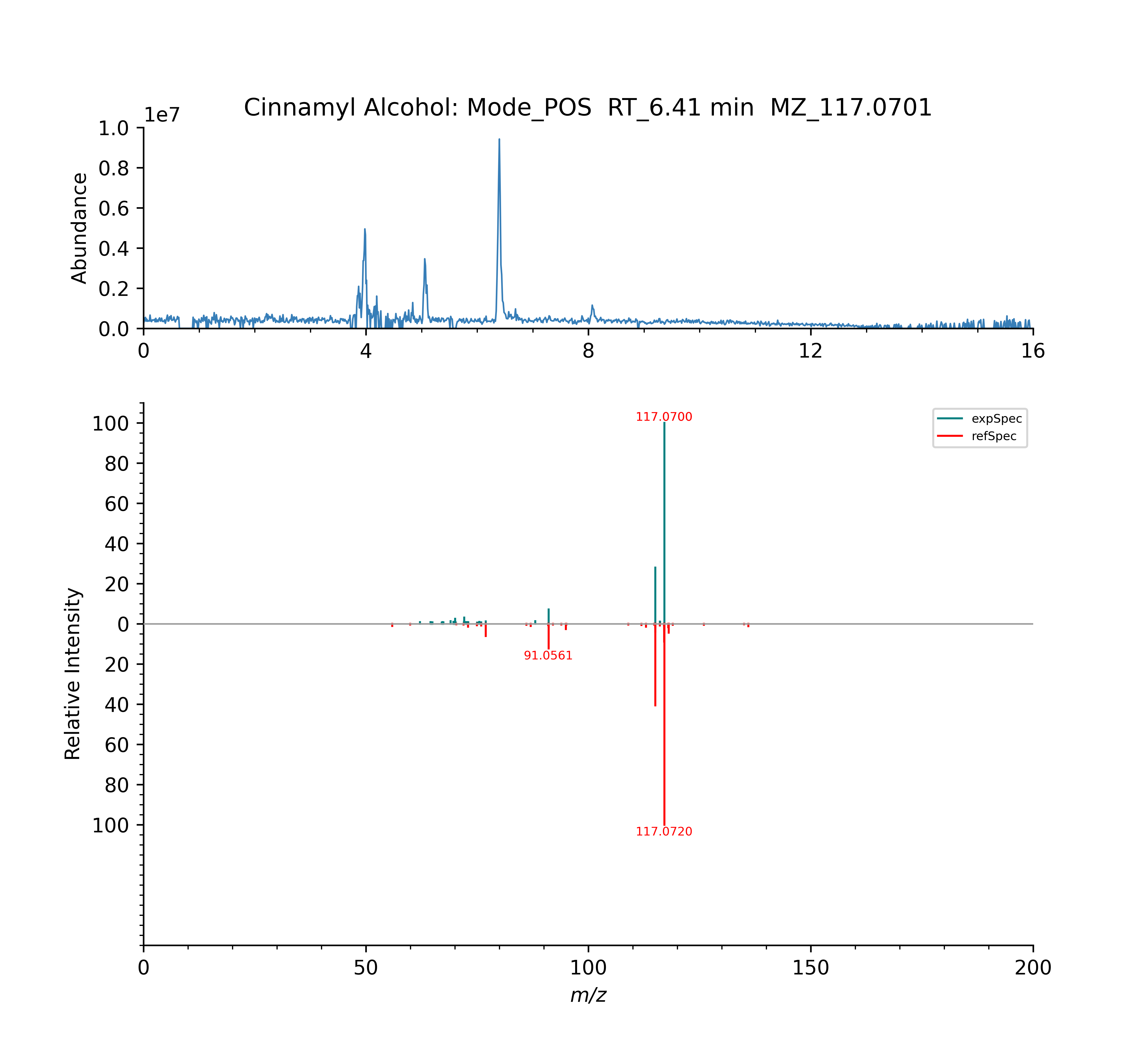

Supplement: Supplementary file 1 [file ijms-27-02203-s001.zip › ijms-4070482 Supplementary/Metabolite List Identified by LC-MS_MS from Rhodiola Species/41.png]

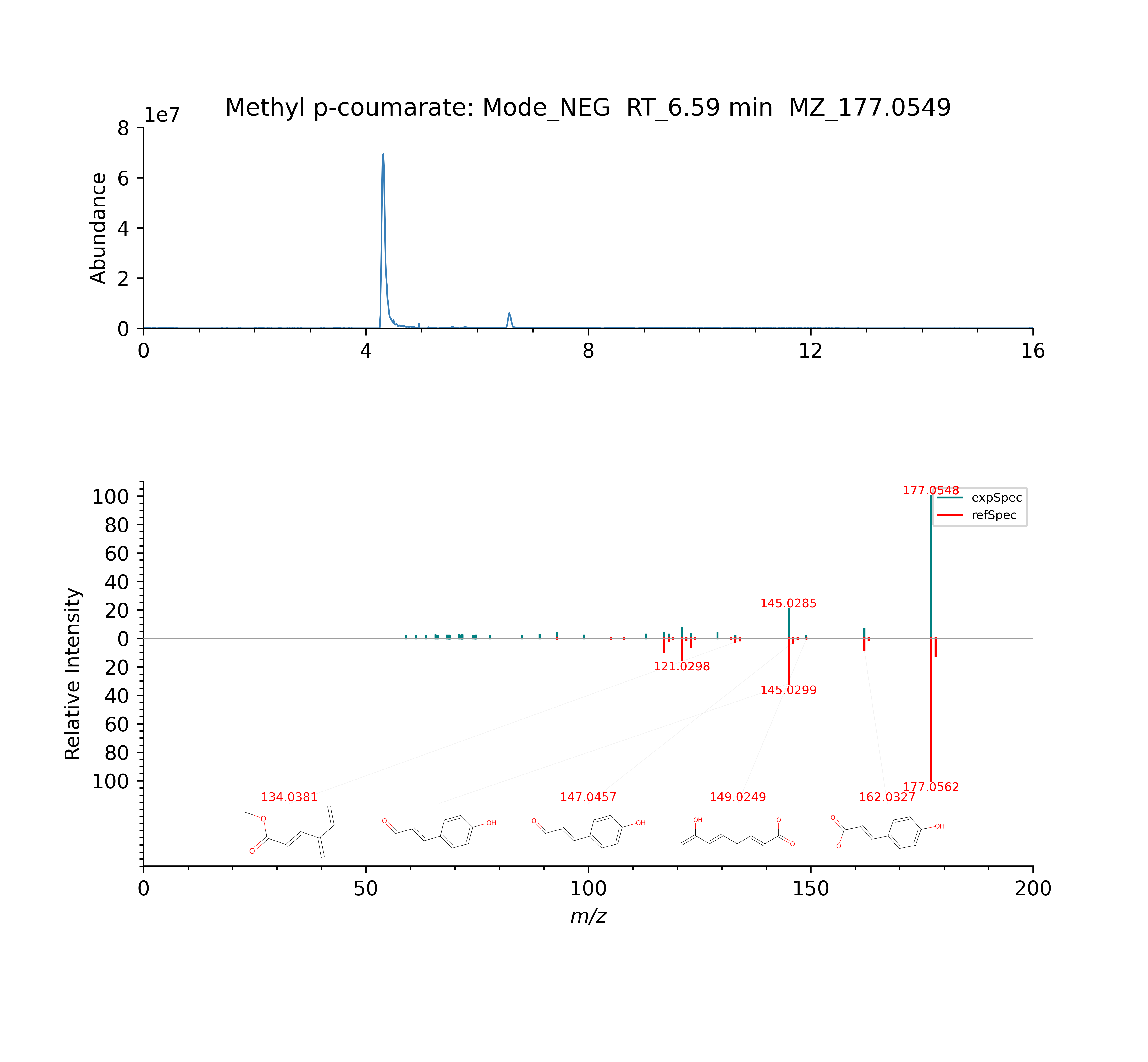

Supplement: Supplementary file 1 [file ijms-27-02203-s001.zip › ijms-4070482 Supplementary/Metabolite List Identified by LC-MS_MS from Rhodiola Species/42.png]

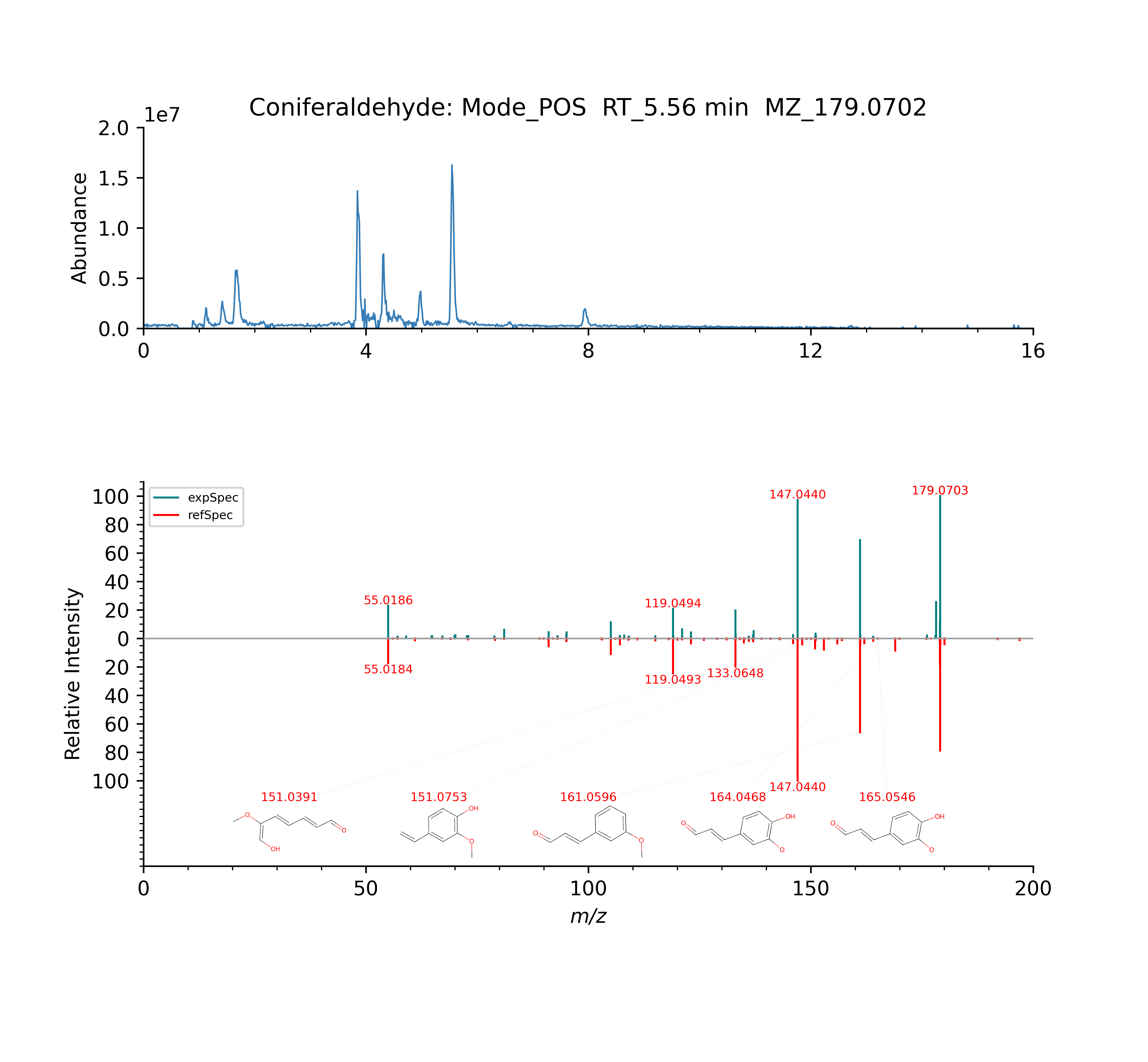

Supplement: Supplementary file 1 [file ijms-27-02203-s001.zip › ijms-4070482 Supplementary/Metabolite List Identified by LC-MS_MS from Rhodiola Species/43.png]

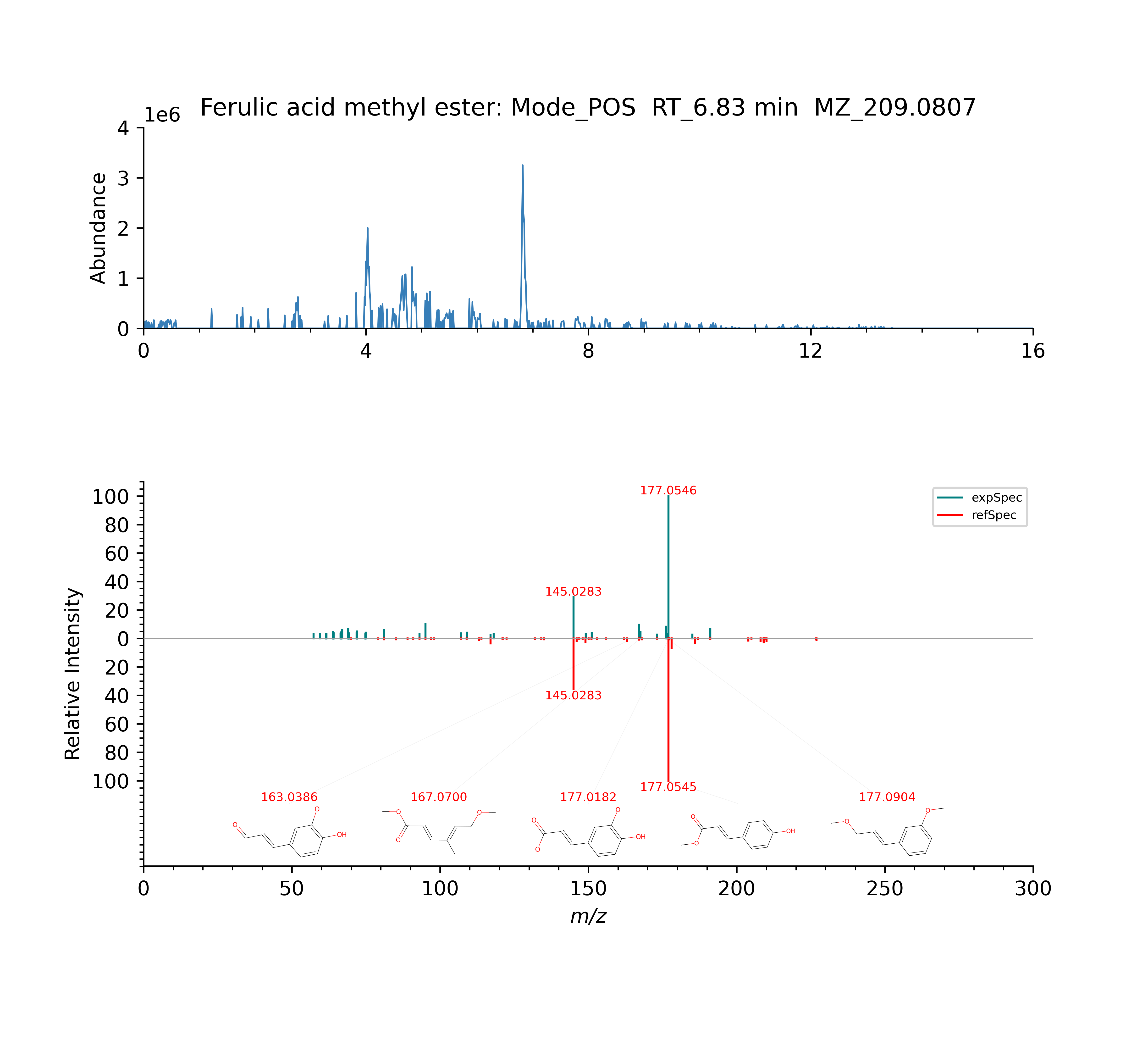

Supplement: Supplementary file 1 [file ijms-27-02203-s001.zip › ijms-4070482 Supplementary/Metabolite List Identified by LC-MS_MS from Rhodiola Species/44.png]

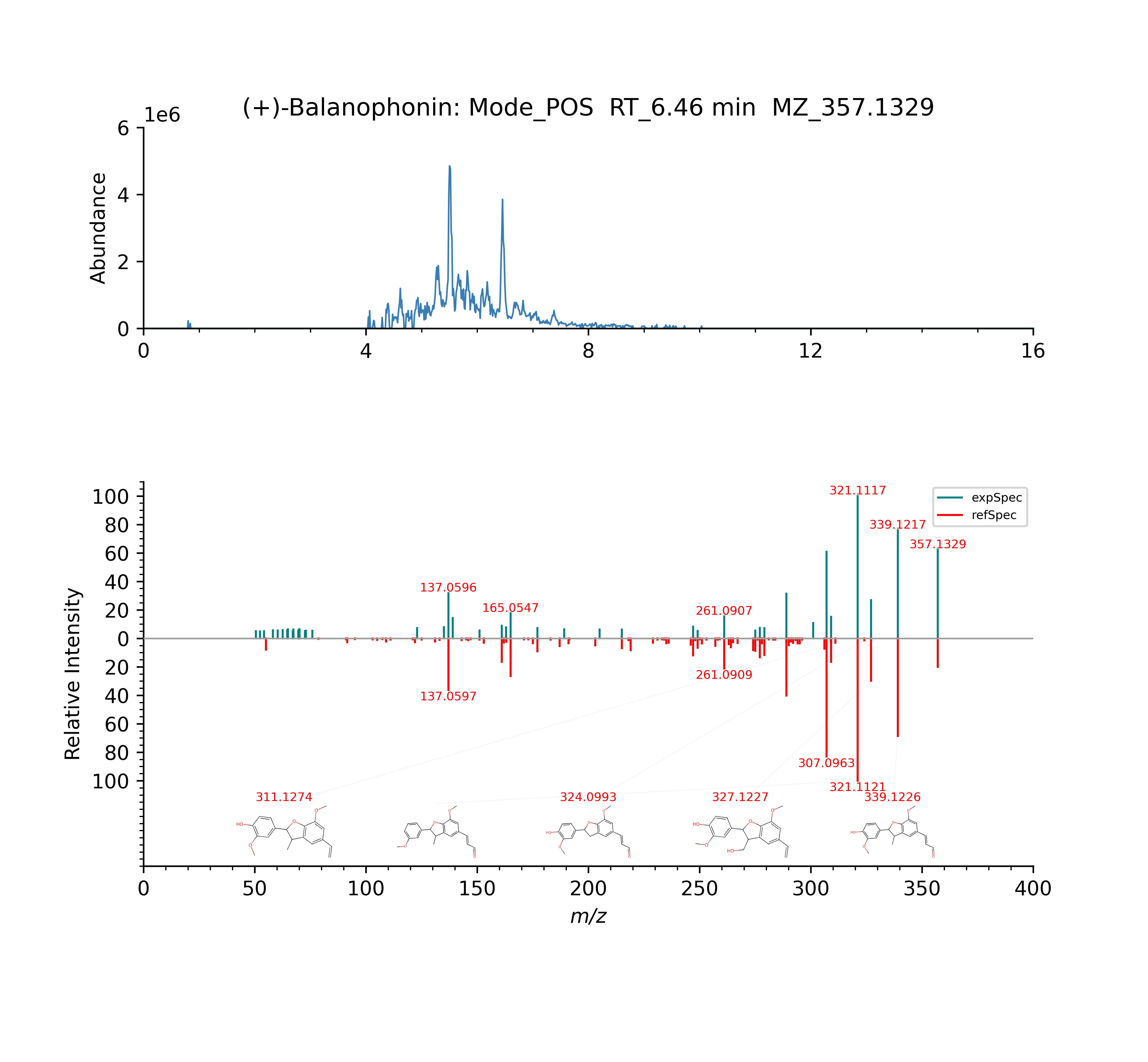

Supplement: Supplementary file 1 [file ijms-27-02203-s001.zip › ijms-4070482 Supplementary/Metabolite List Identified by LC-MS_MS from Rhodiola Species/45.png]

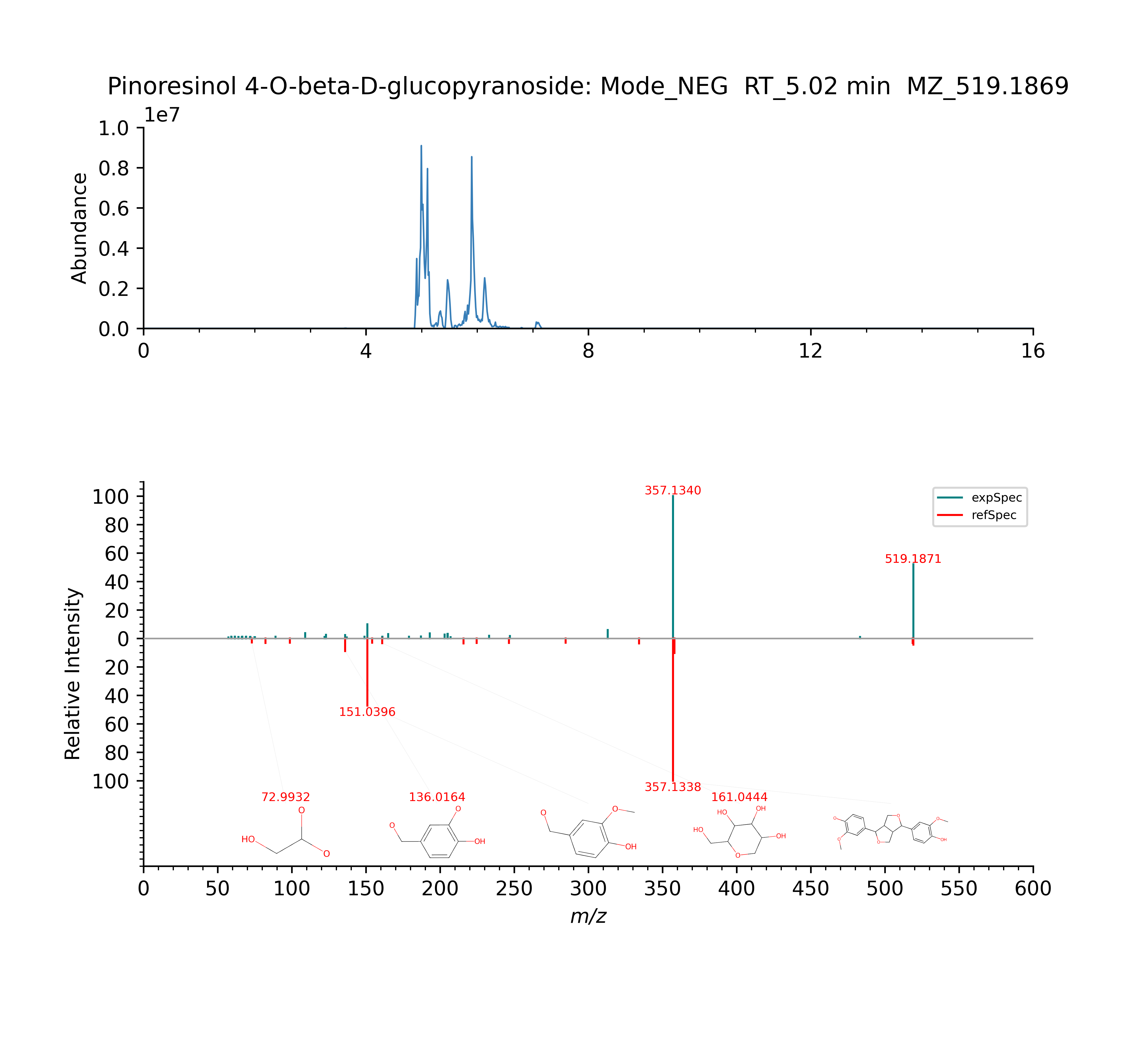

Supplement: Supplementary file 1 [file ijms-27-02203-s001.zip › ijms-4070482 Supplementary/Metabolite List Identified by LC-MS_MS from Rhodiola Species/46.png]

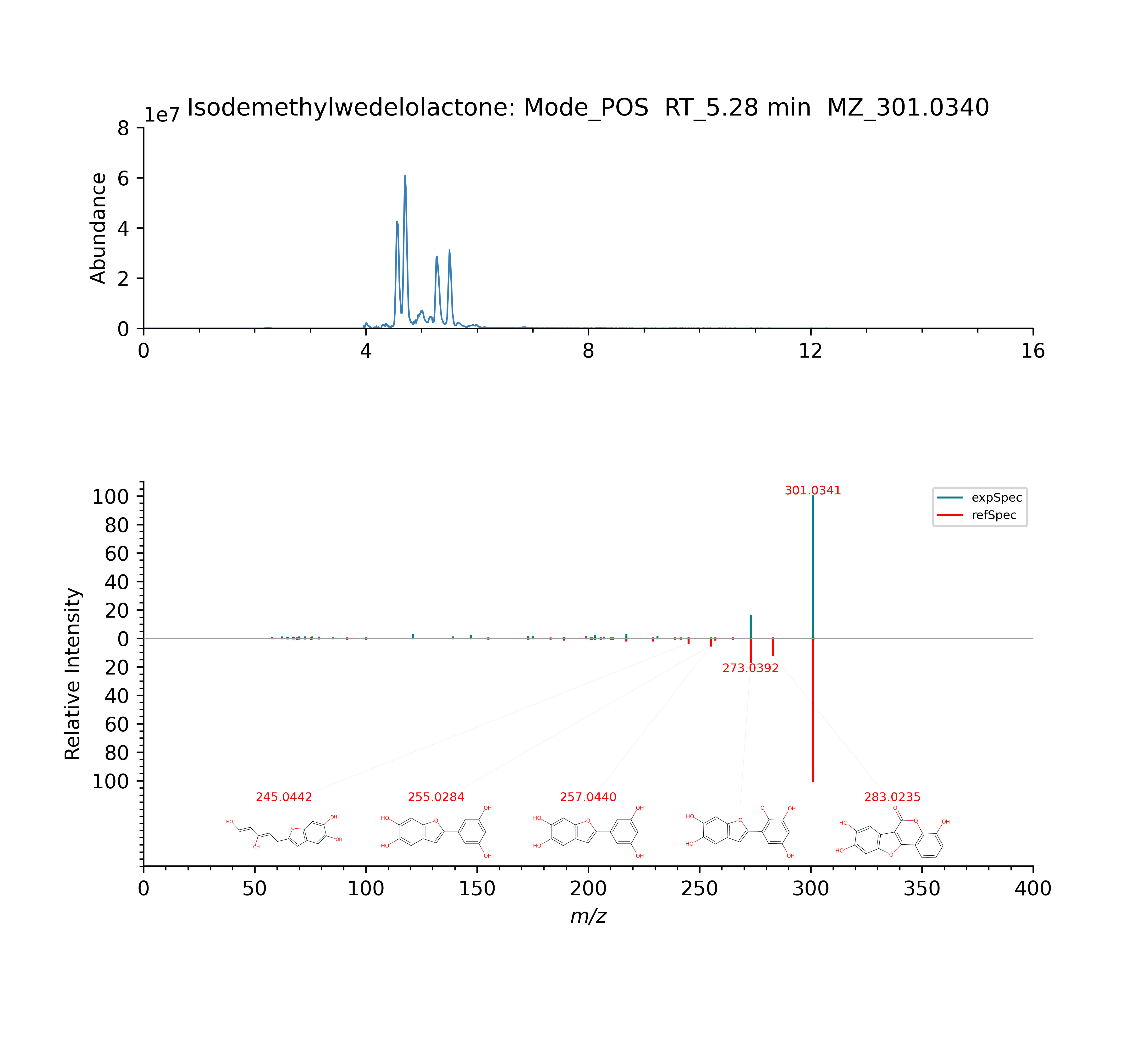

Supplement: Supplementary file 1 [file ijms-27-02203-s001.zip › ijms-4070482 Supplementary/Metabolite List Identified by LC-MS_MS from Rhodiola Species/47.png]

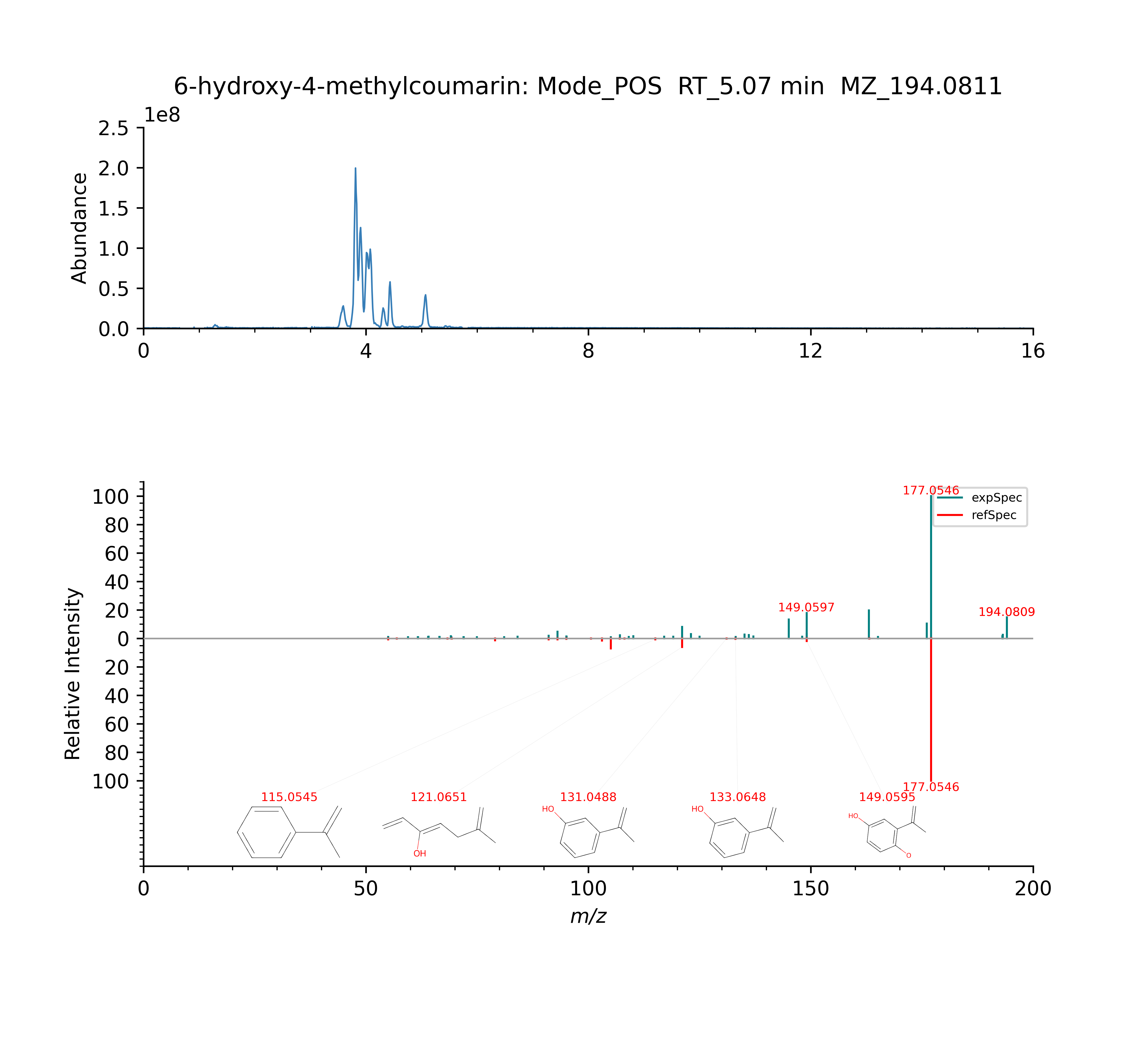

Supplement: Supplementary file 1 [file ijms-27-02203-s001.zip › ijms-4070482 Supplementary/Metabolite List Identified by LC-MS_MS from Rhodiola Species/48.png]

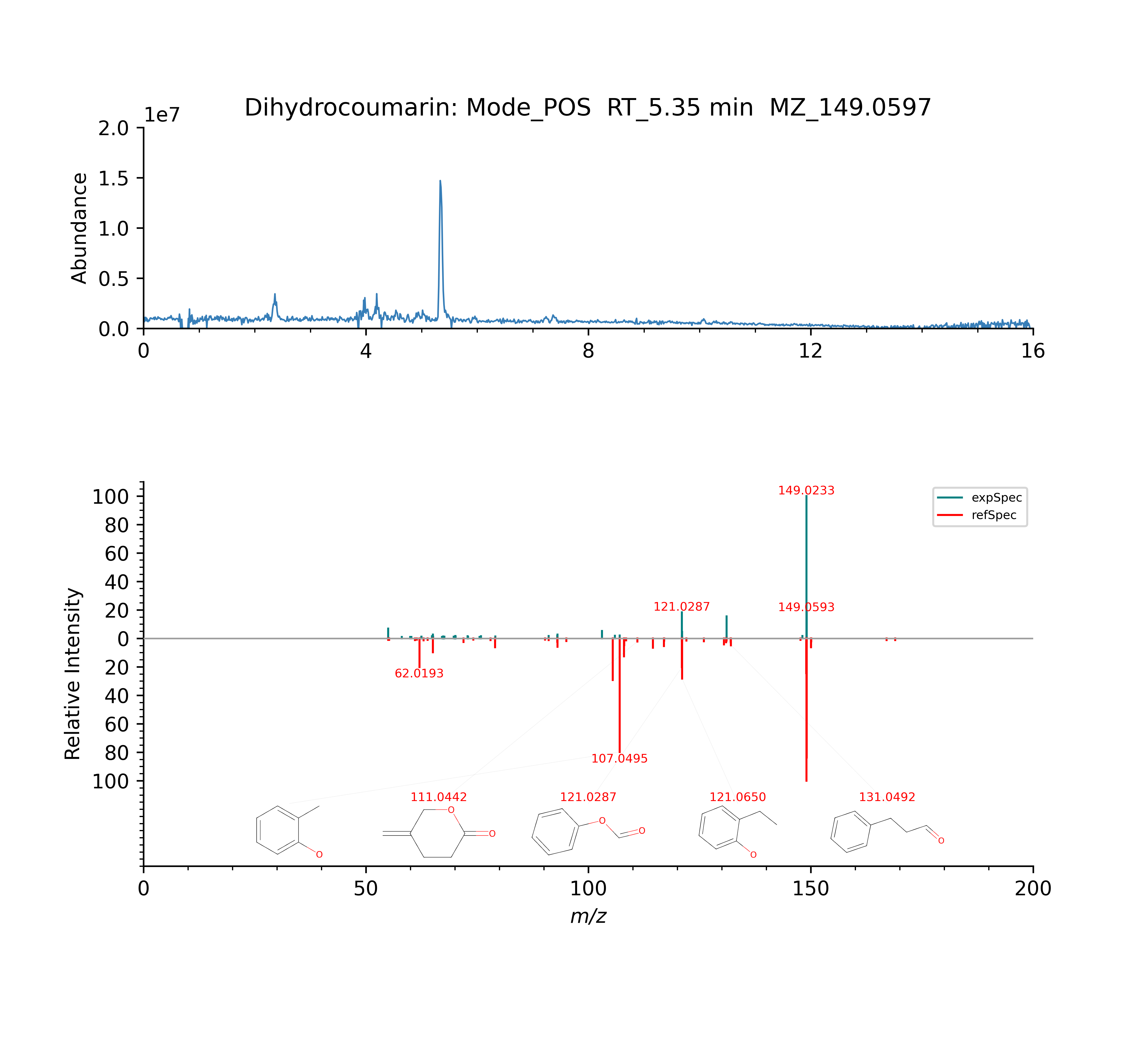

Supplement: Supplementary file 1 [file ijms-27-02203-s001.zip › ijms-4070482 Supplementary/Metabolite List Identified by LC-MS_MS from Rhodiola Species/49.png]

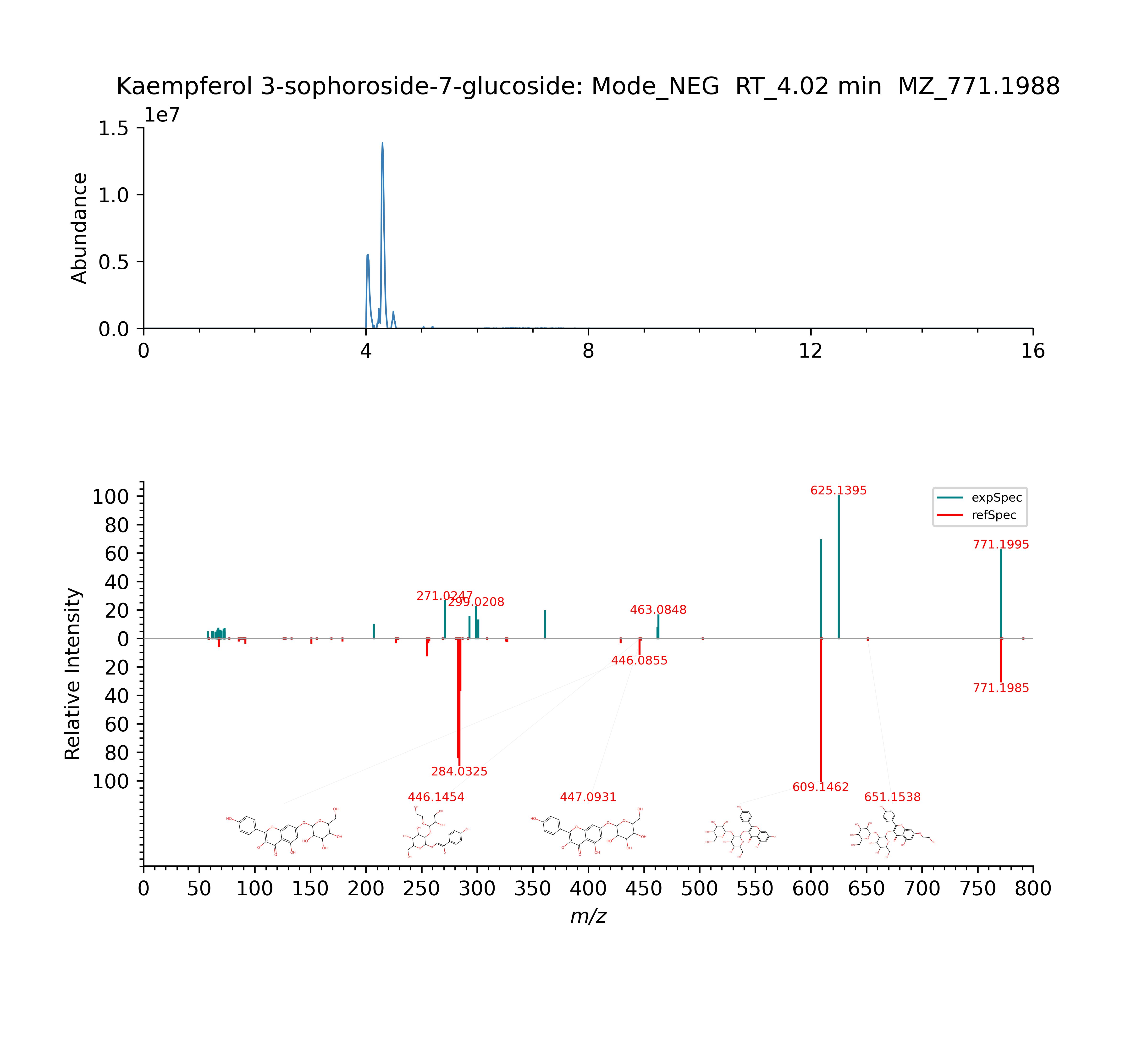

Supplement: Supplementary file 1 [file ijms-27-02203-s001.zip › ijms-4070482 Supplementary/Metabolite List Identified by LC-MS_MS from Rhodiola Species/5.png]

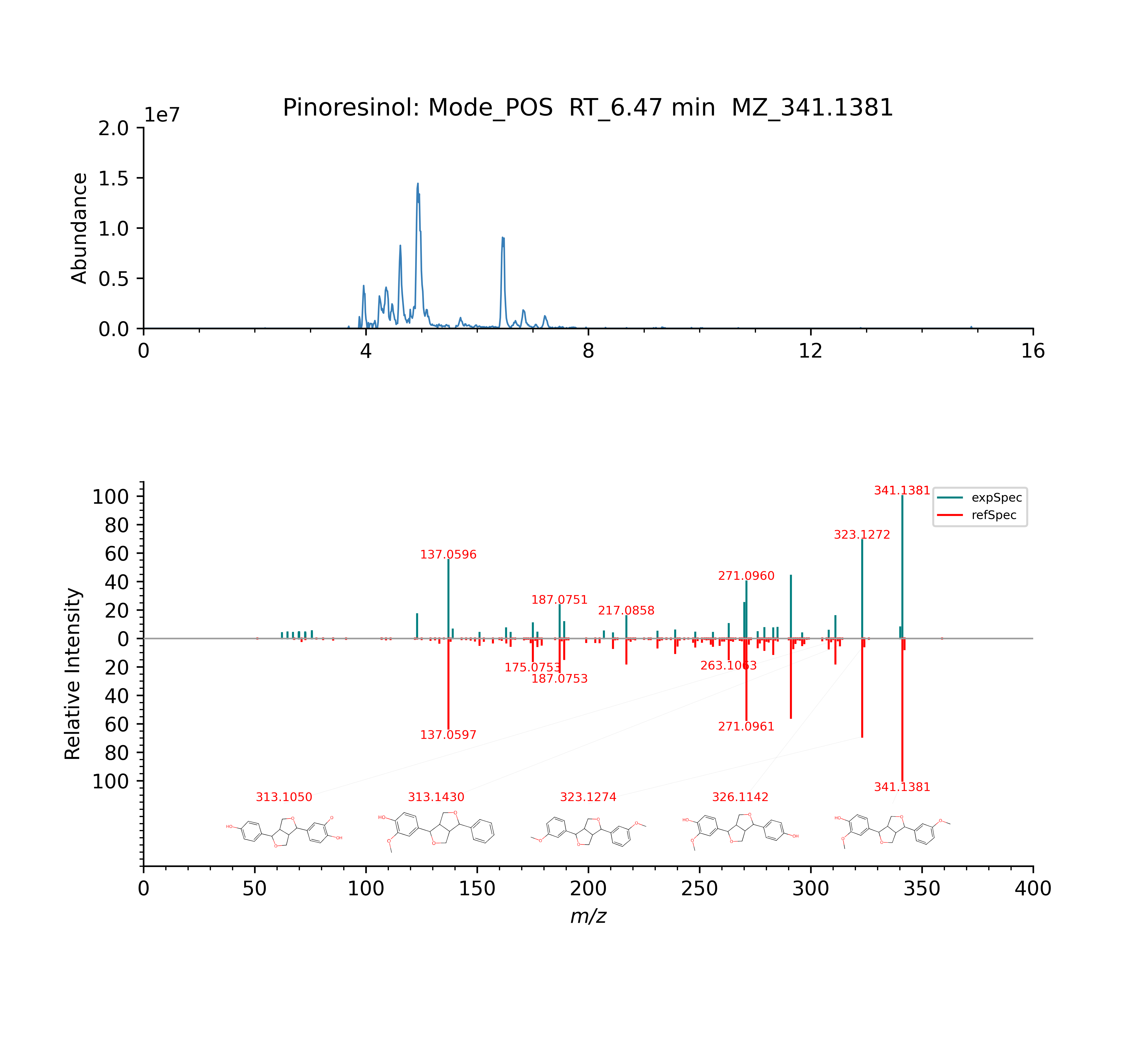

Supplement: Supplementary file 1 [file ijms-27-02203-s001.zip › ijms-4070482 Supplementary/Metabolite List Identified by LC-MS_MS from Rhodiola Species/50.png]

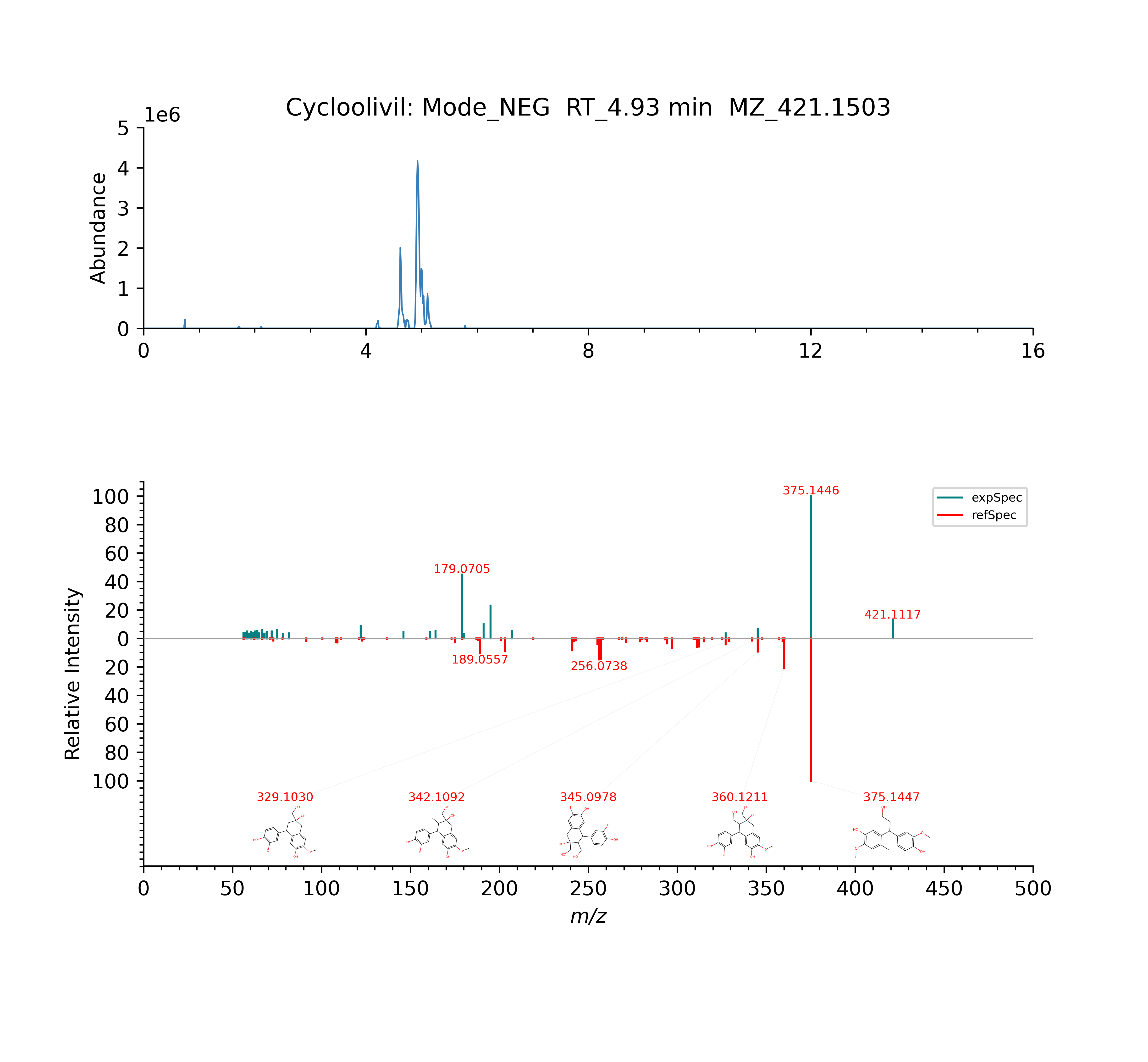

Supplement: Supplementary file 1 [file ijms-27-02203-s001.zip › ijms-4070482 Supplementary/Metabolite List Identified by LC-MS_MS from Rhodiola Species/51.png]

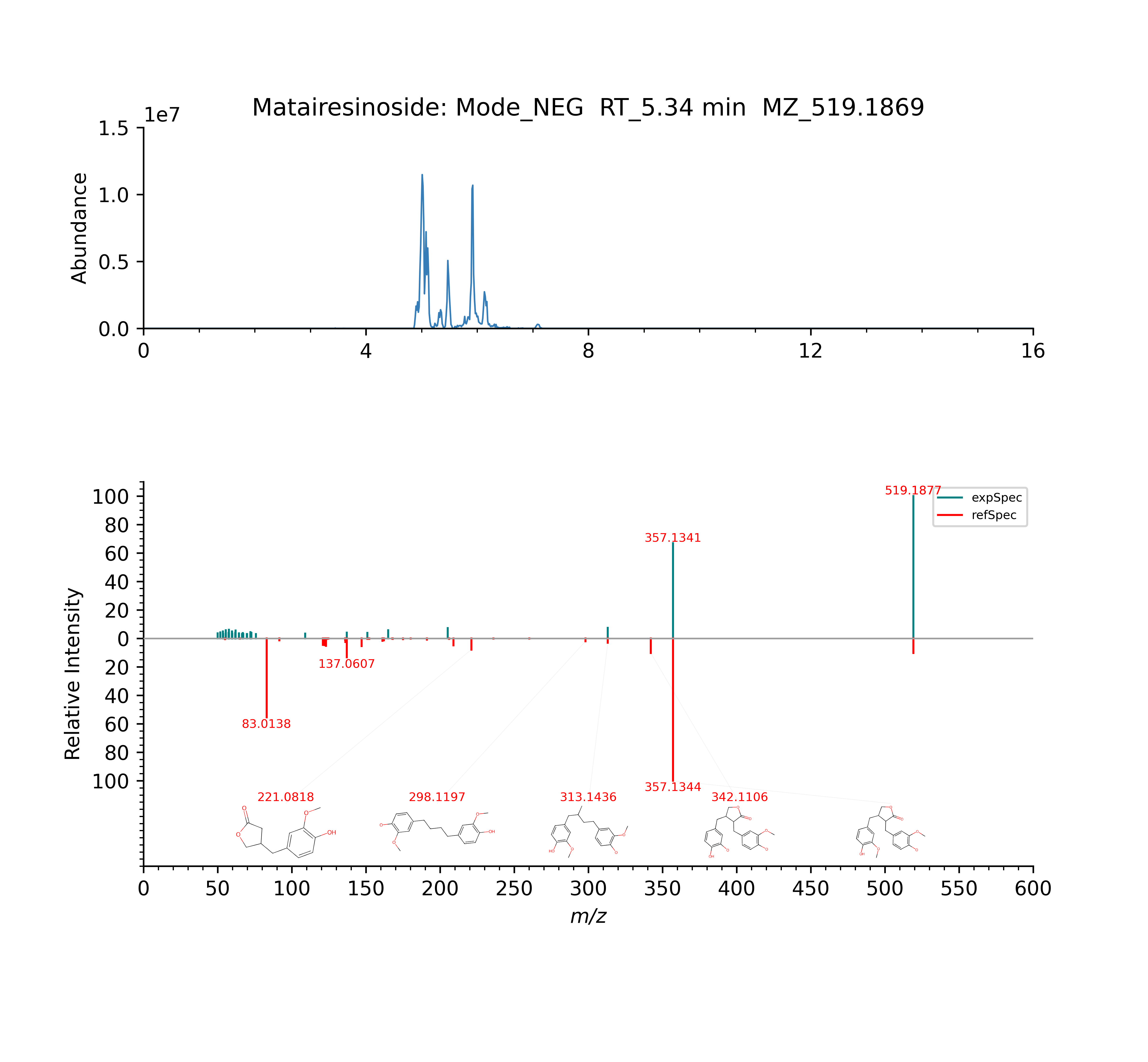

Supplement: Supplementary file 1 [file ijms-27-02203-s001.zip › ijms-4070482 Supplementary/Metabolite List Identified by LC-MS_MS from Rhodiola Species/52.png]

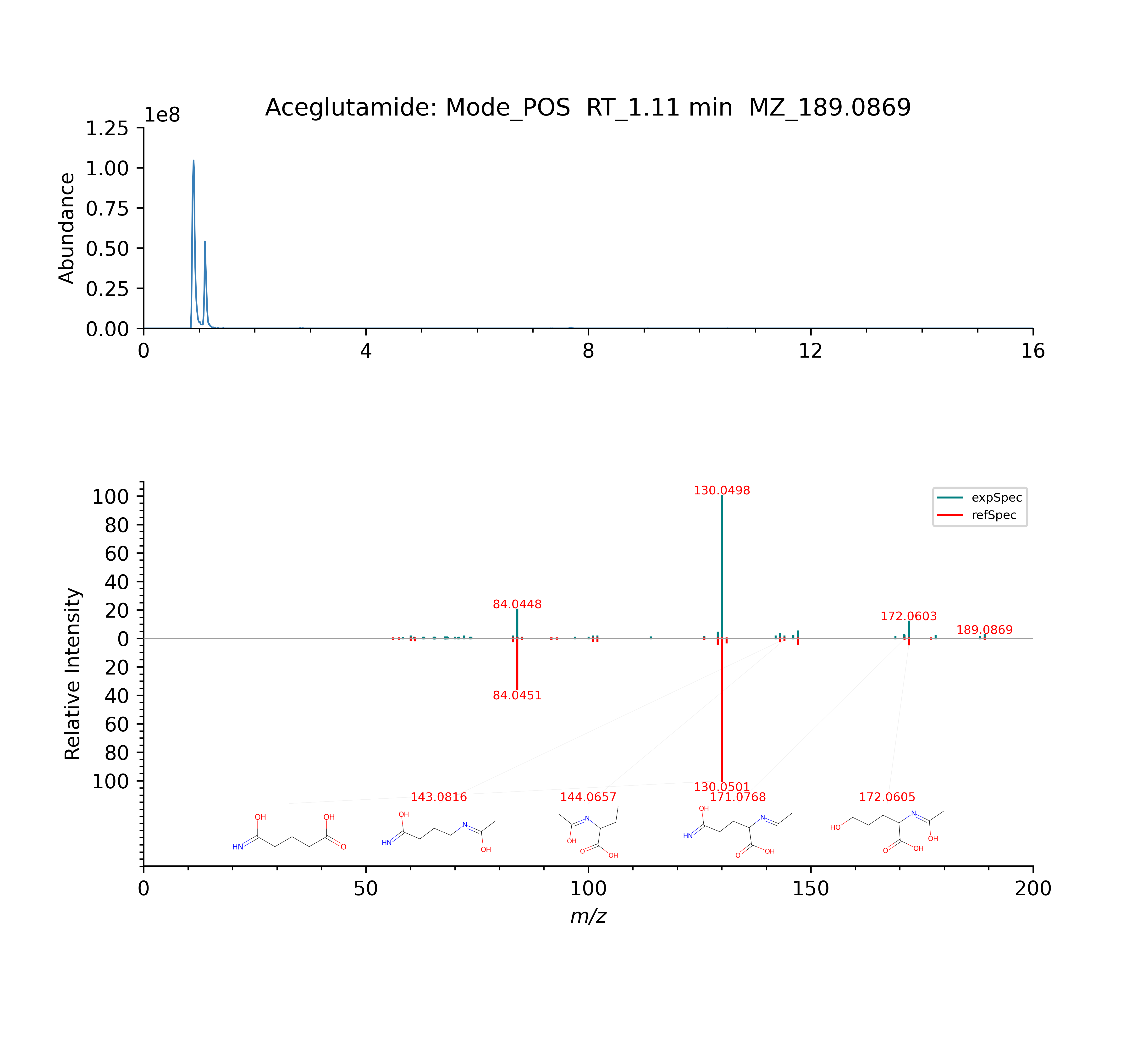

Supplement: Supplementary file 1 [file ijms-27-02203-s001.zip › ijms-4070482 Supplementary/Metabolite List Identified by LC-MS_MS from Rhodiola Species/53.png]

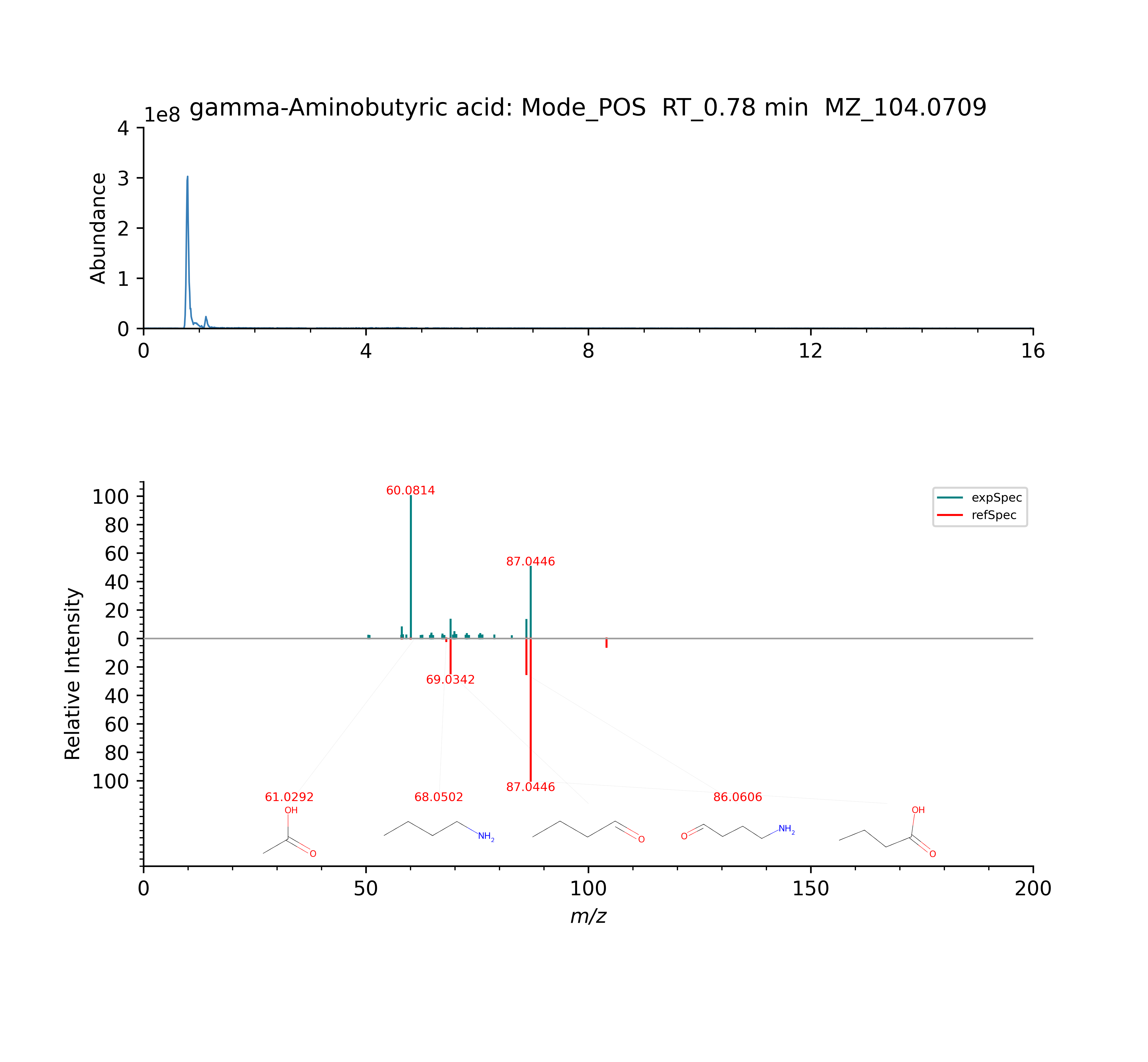

Supplement: Supplementary file 1 [file ijms-27-02203-s001.zip › ijms-4070482 Supplementary/Metabolite List Identified by LC-MS_MS from Rhodiola Species/54.png]

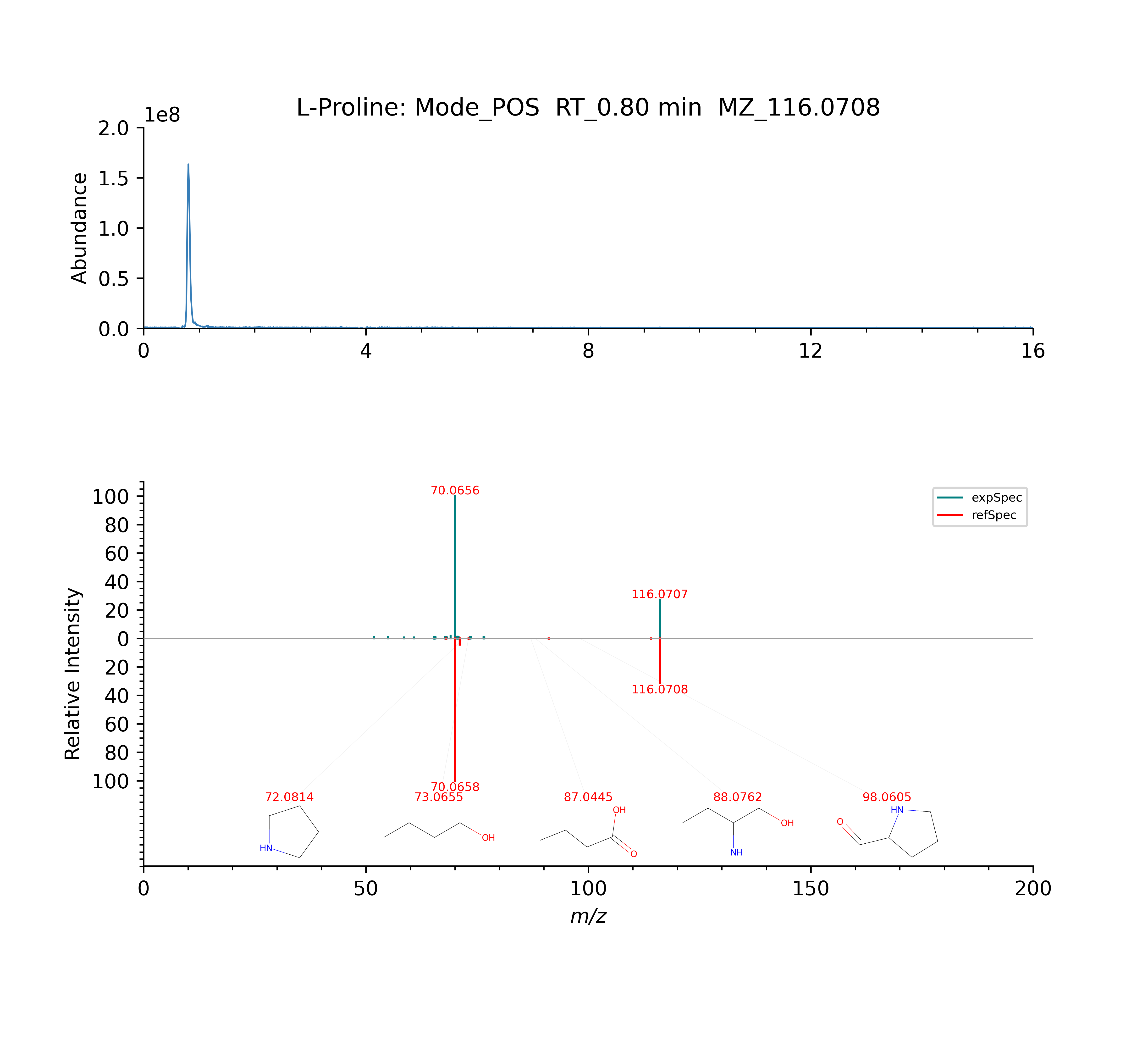

Supplement: Supplementary file 1 [file ijms-27-02203-s001.zip › ijms-4070482 Supplementary/Metabolite List Identified by LC-MS_MS from Rhodiola Species/55.png]

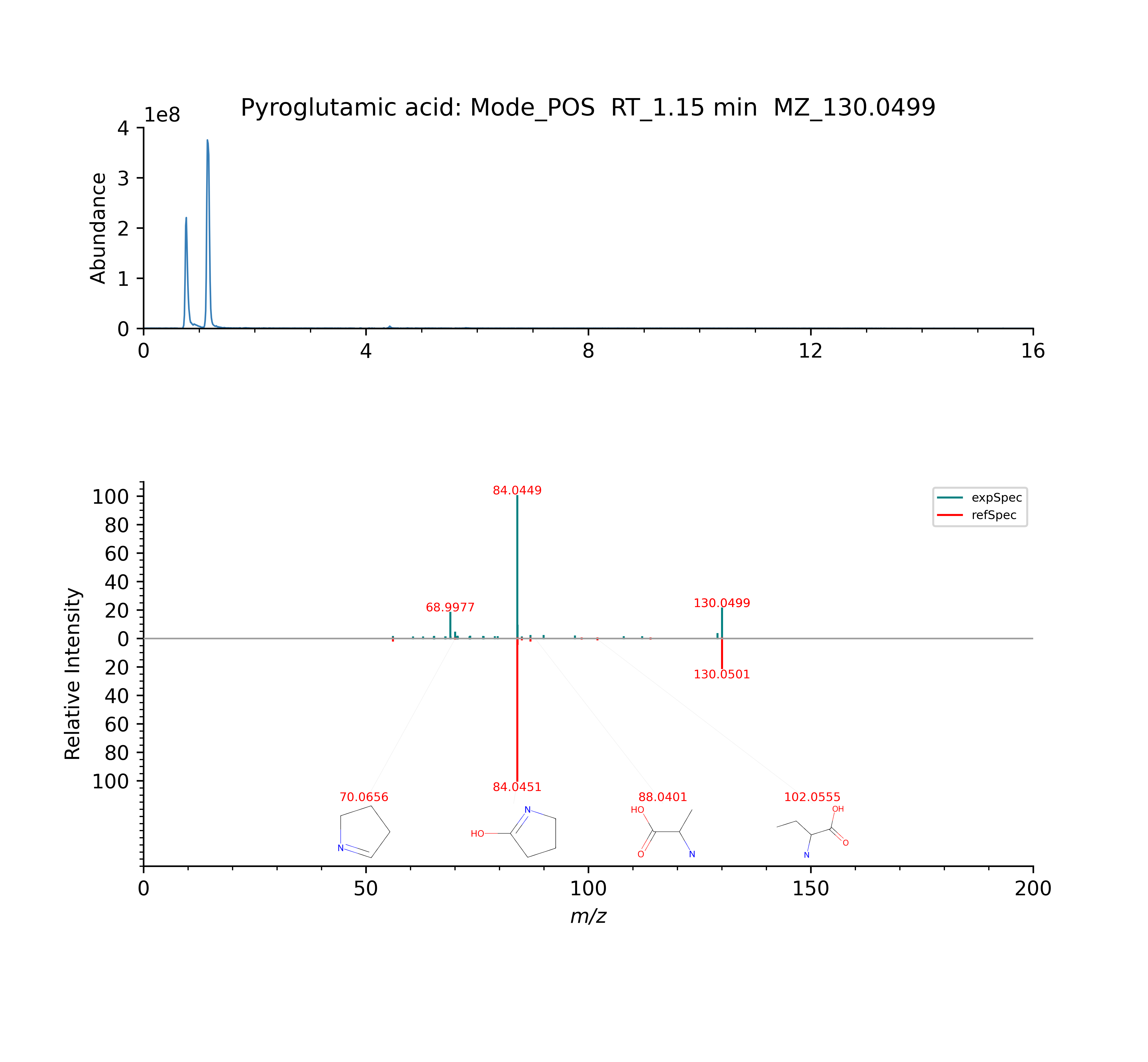

Supplement: Supplementary file 1 [file ijms-27-02203-s001.zip › ijms-4070482 Supplementary/Metabolite List Identified by LC-MS_MS from Rhodiola Species/56.png]

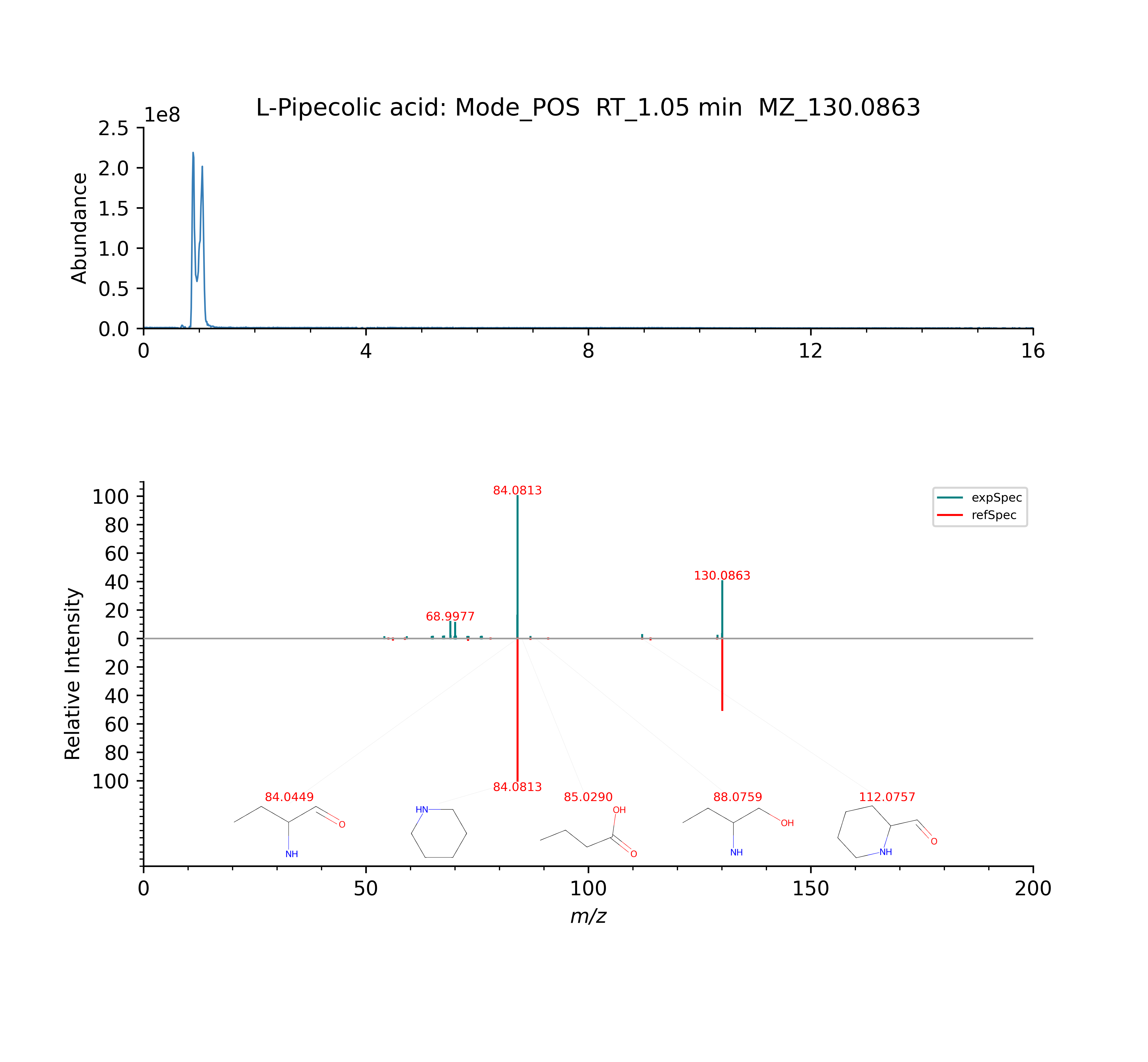

Supplement: Supplementary file 1 [file ijms-27-02203-s001.zip › ijms-4070482 Supplementary/Metabolite List Identified by LC-MS_MS from Rhodiola Species/57.png]

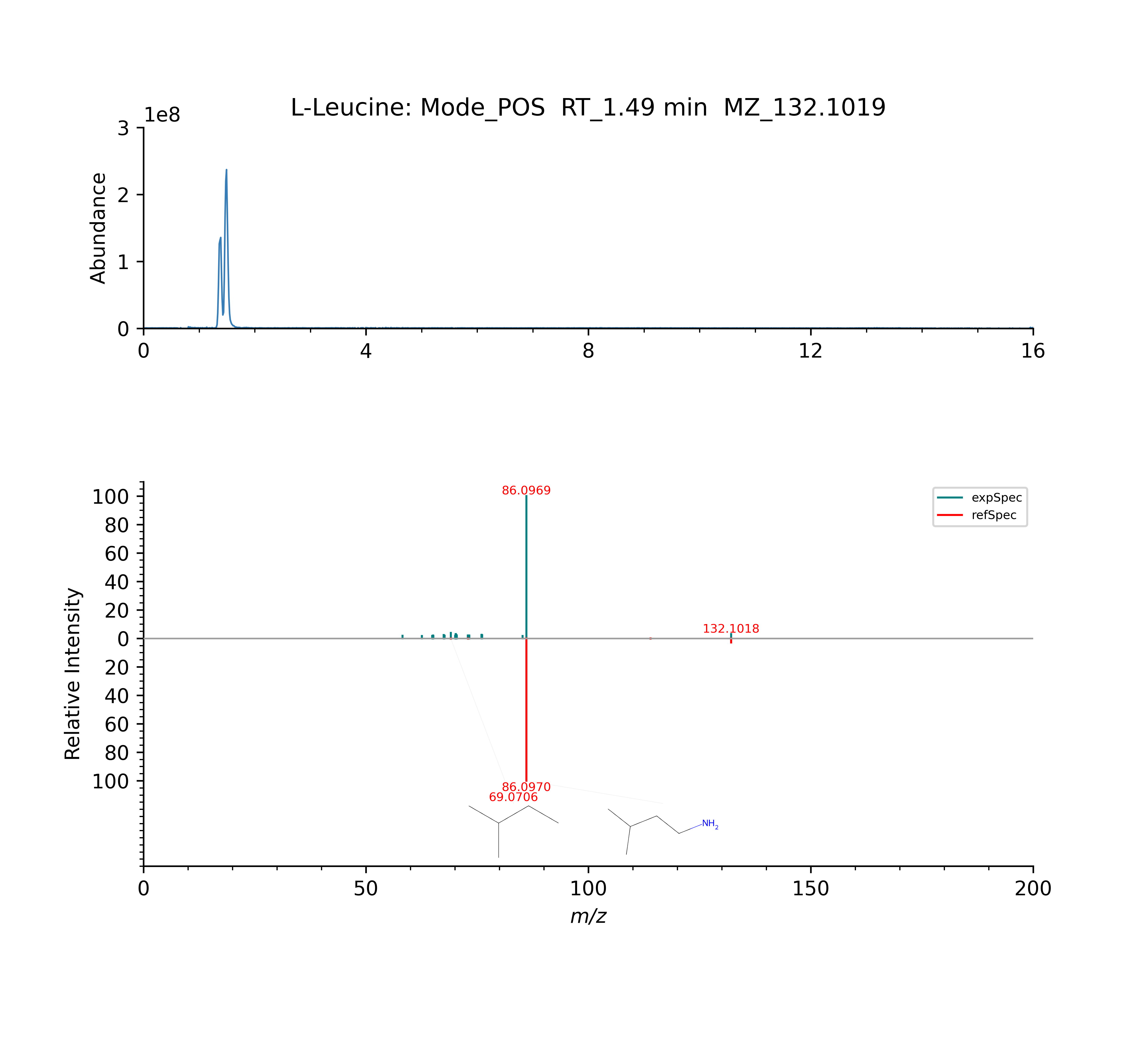

Supplement: Supplementary file 1 [file ijms-27-02203-s001.zip › ijms-4070482 Supplementary/Metabolite List Identified by LC-MS_MS from Rhodiola Species/58.png]

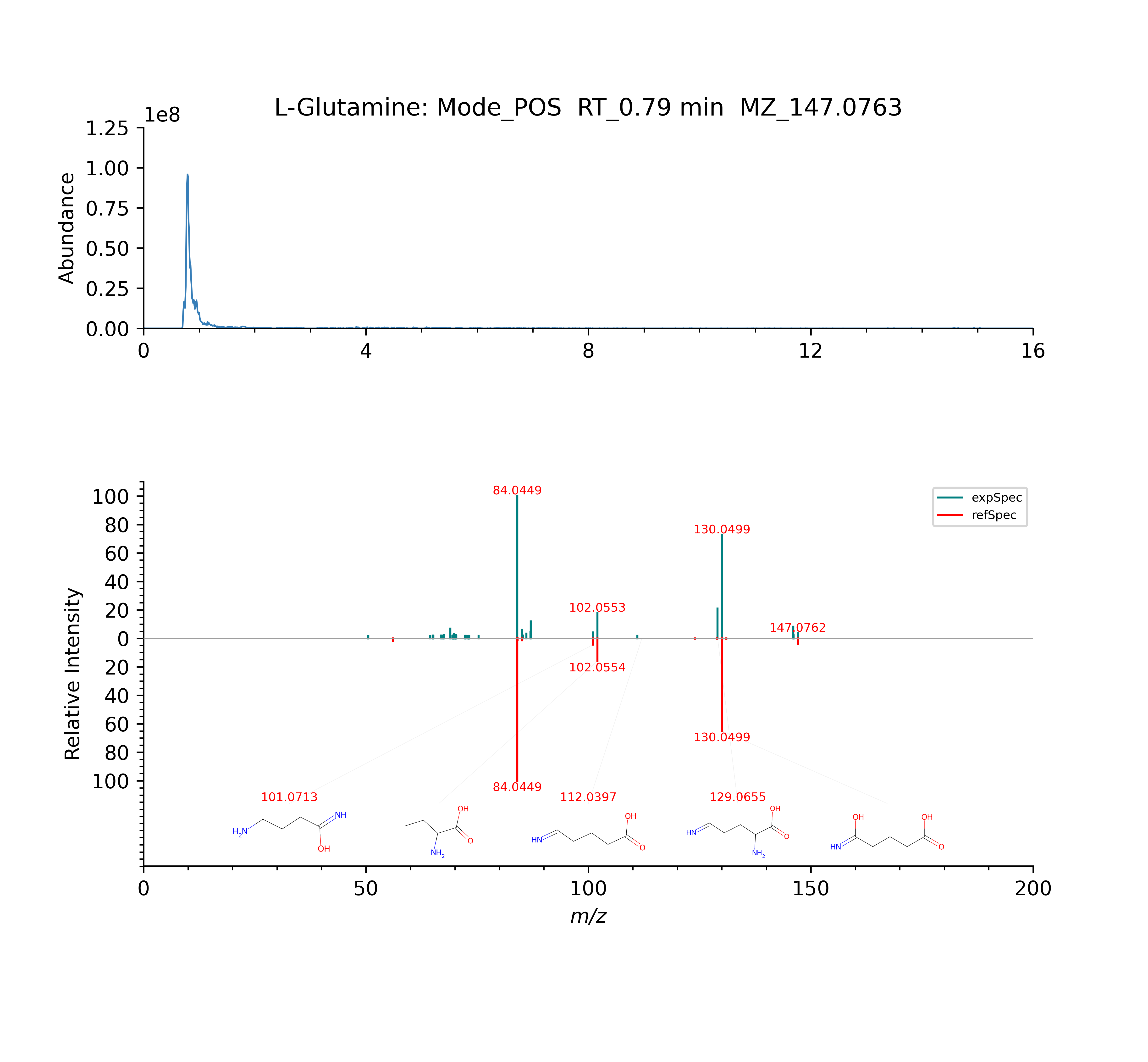

Supplement: Supplementary file 1 [file ijms-27-02203-s001.zip › ijms-4070482 Supplementary/Metabolite List Identified by LC-MS_MS from Rhodiola Species/59.png]

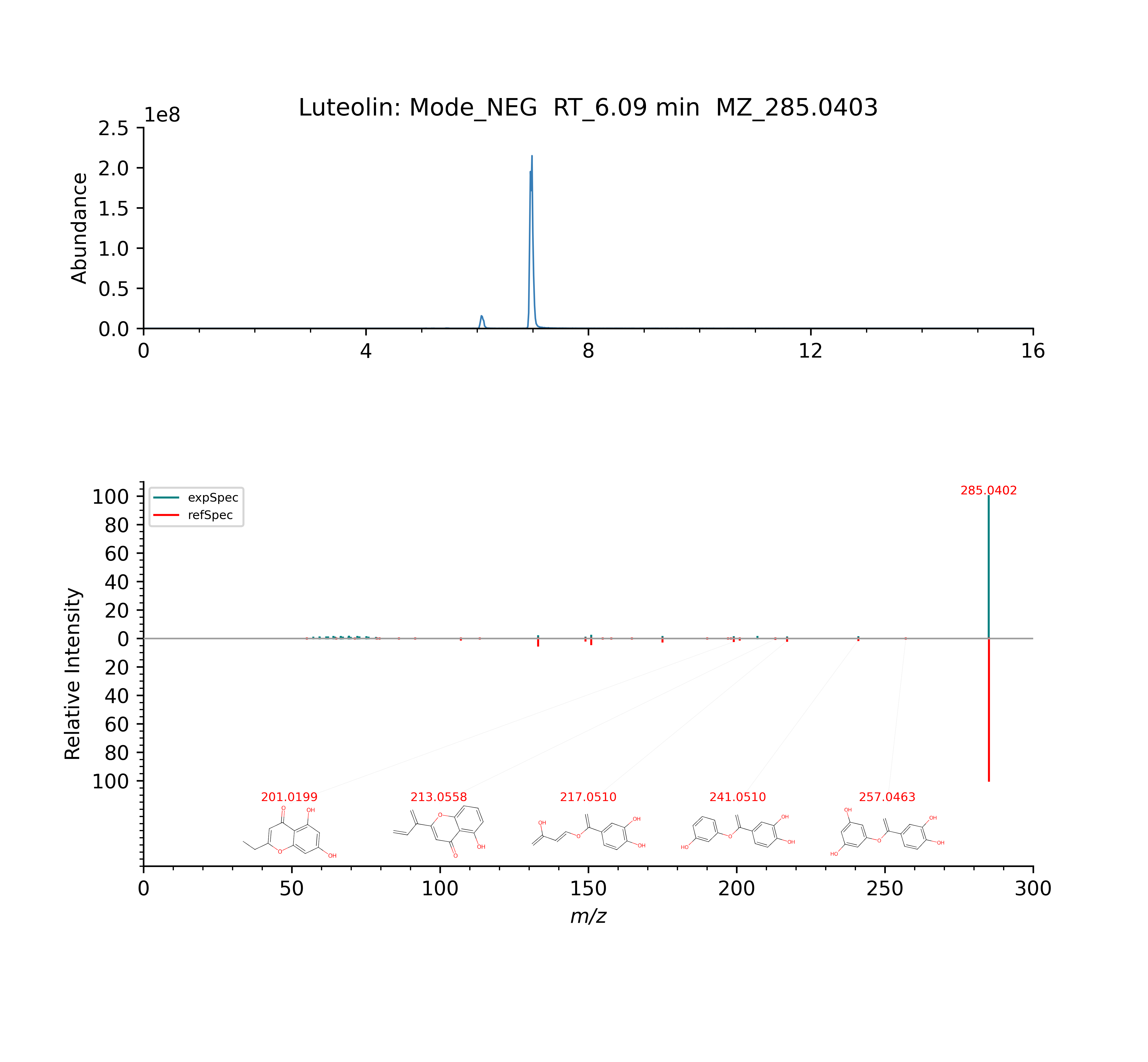

Supplement: Supplementary file 1 [file ijms-27-02203-s001.zip › ijms-4070482 Supplementary/Metabolite List Identified by LC-MS_MS from Rhodiola Species/6.png]

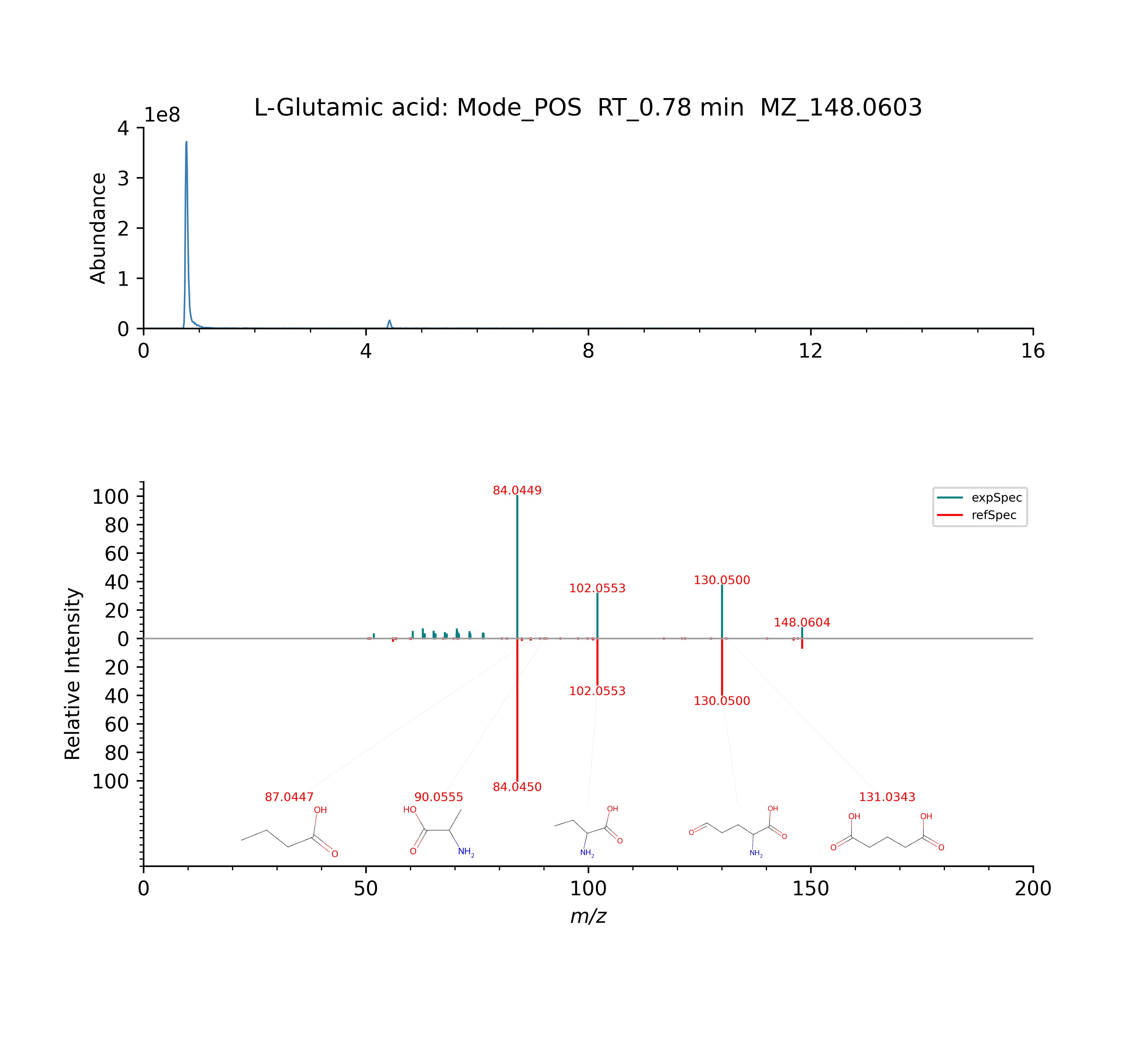

Supplement: Supplementary file 1 [file ijms-27-02203-s001.zip › ijms-4070482 Supplementary/Metabolite List Identified by LC-MS_MS from Rhodiola Species/60.png]

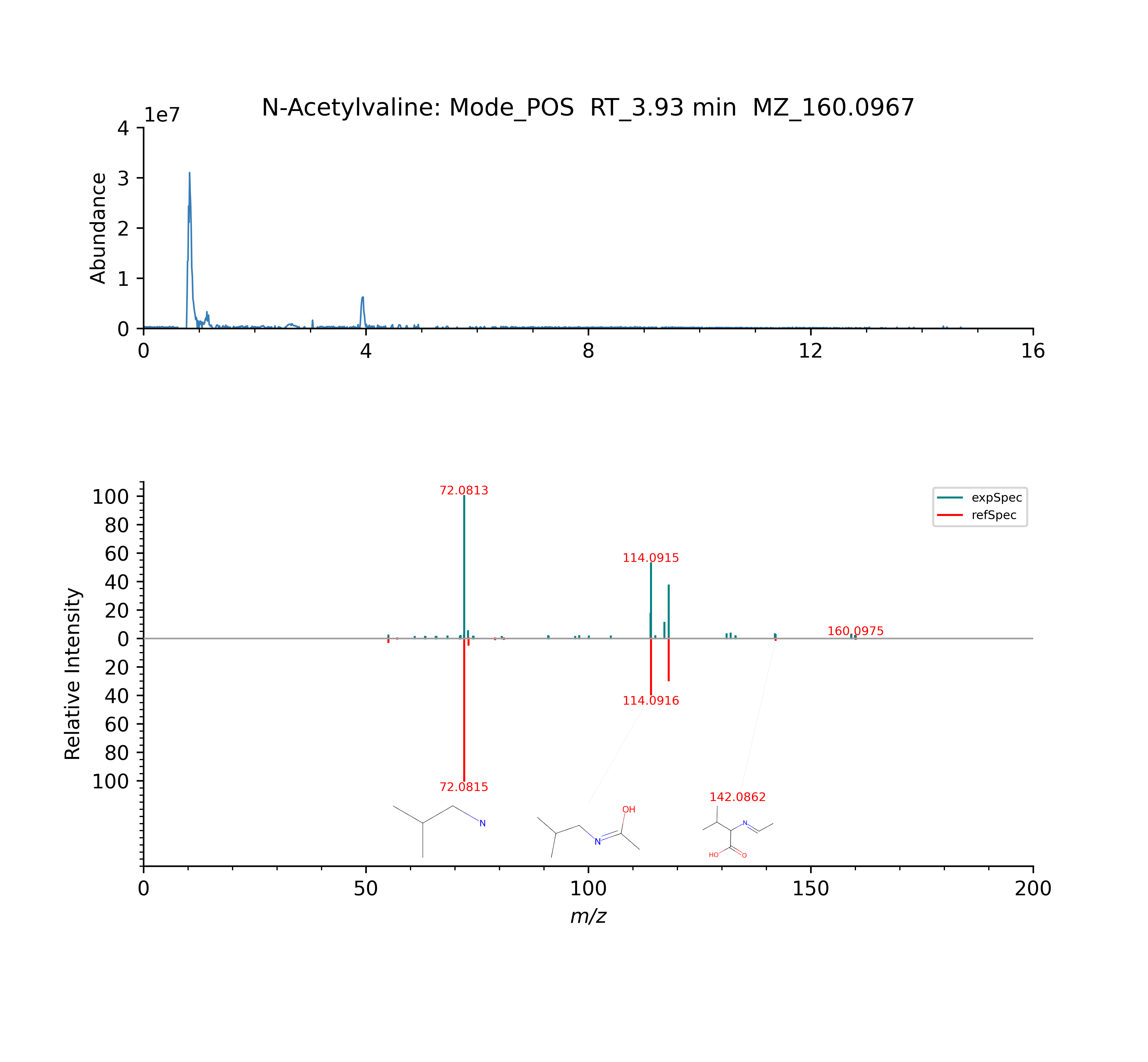

Supplement: Supplementary file 1 [file ijms-27-02203-s001.zip › ijms-4070482 Supplementary/Metabolite List Identified by LC-MS_MS from Rhodiola Species/61.png]

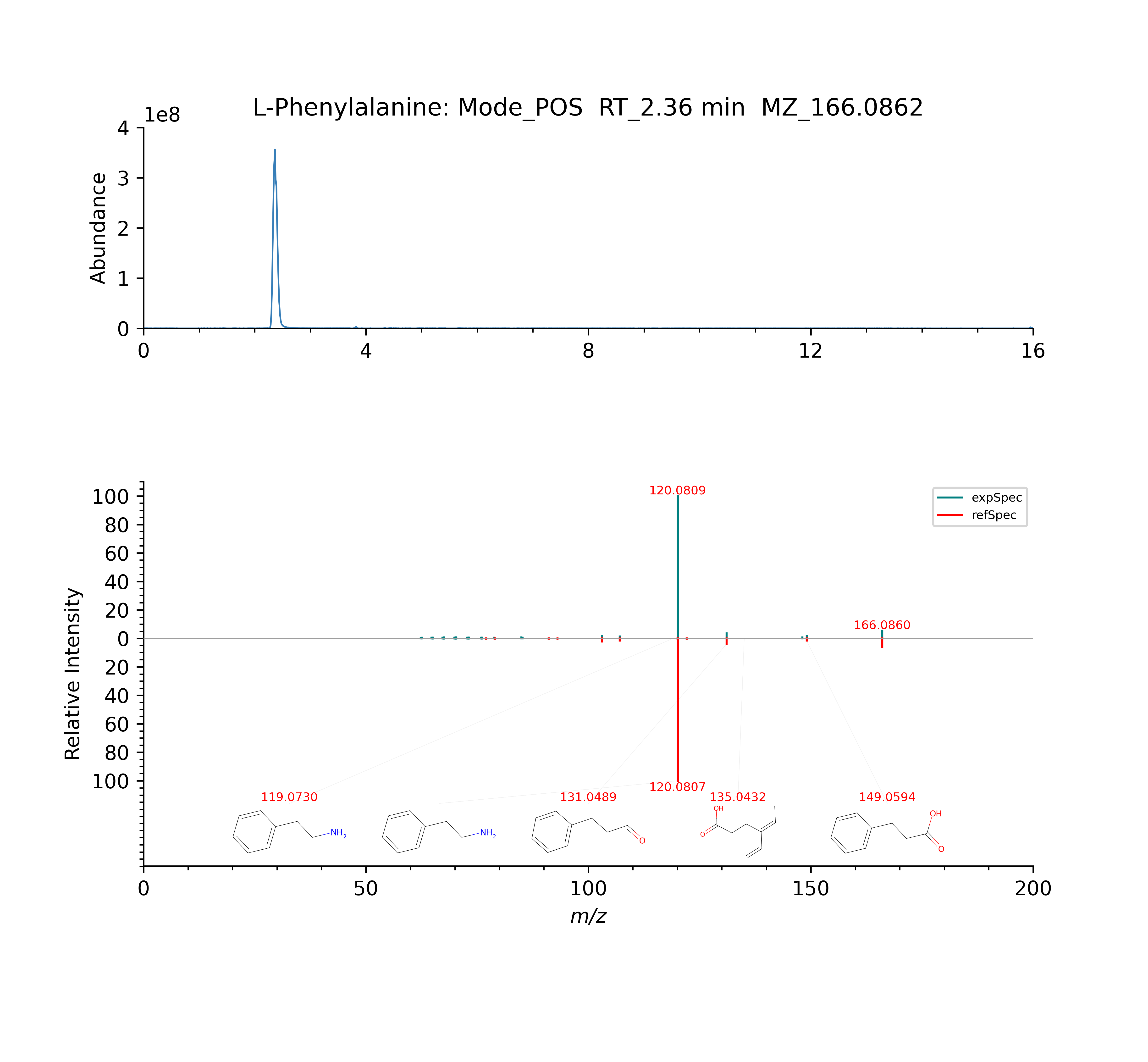

Supplement: Supplementary file 1 [file ijms-27-02203-s001.zip › ijms-4070482 Supplementary/Metabolite List Identified by LC-MS_MS from Rhodiola Species/62.png]

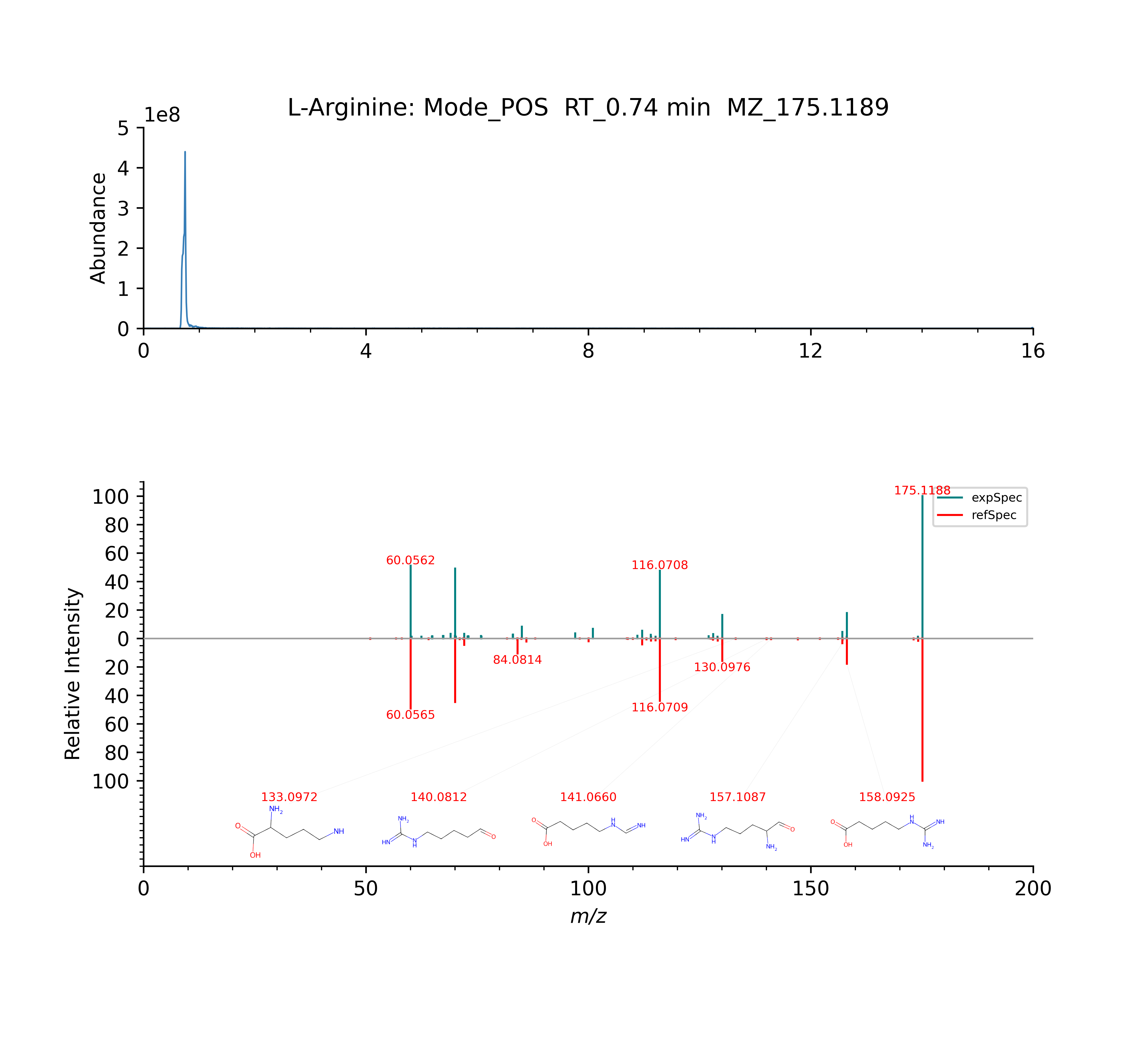

Supplement: Supplementary file 1 [file ijms-27-02203-s001.zip › ijms-4070482 Supplementary/Metabolite List Identified by LC-MS_MS from Rhodiola Species/63.png]

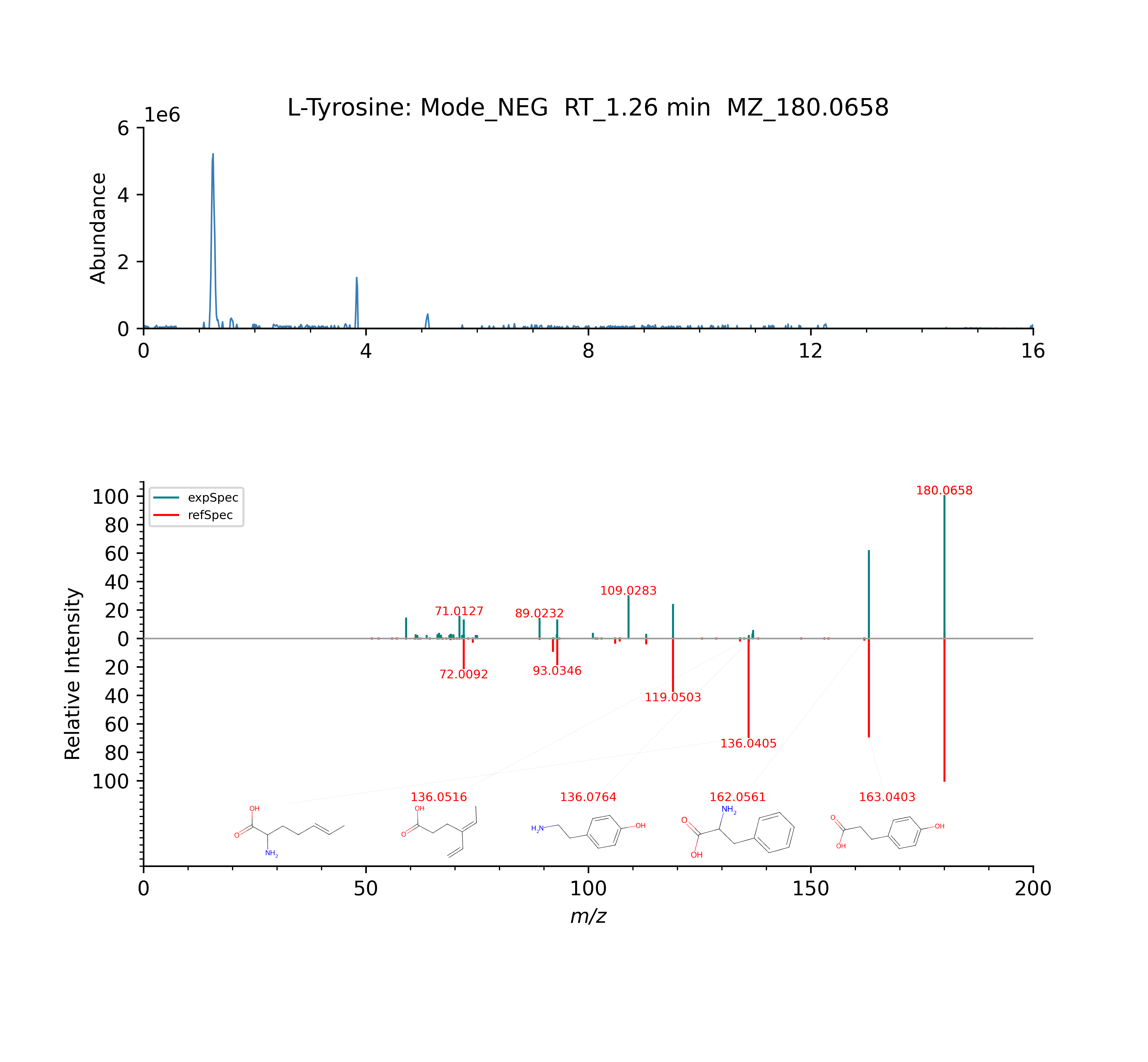

Supplement: Supplementary file 1 [file ijms-27-02203-s001.zip › ijms-4070482 Supplementary/Metabolite List Identified by LC-MS_MS from Rhodiola Species/64.png]

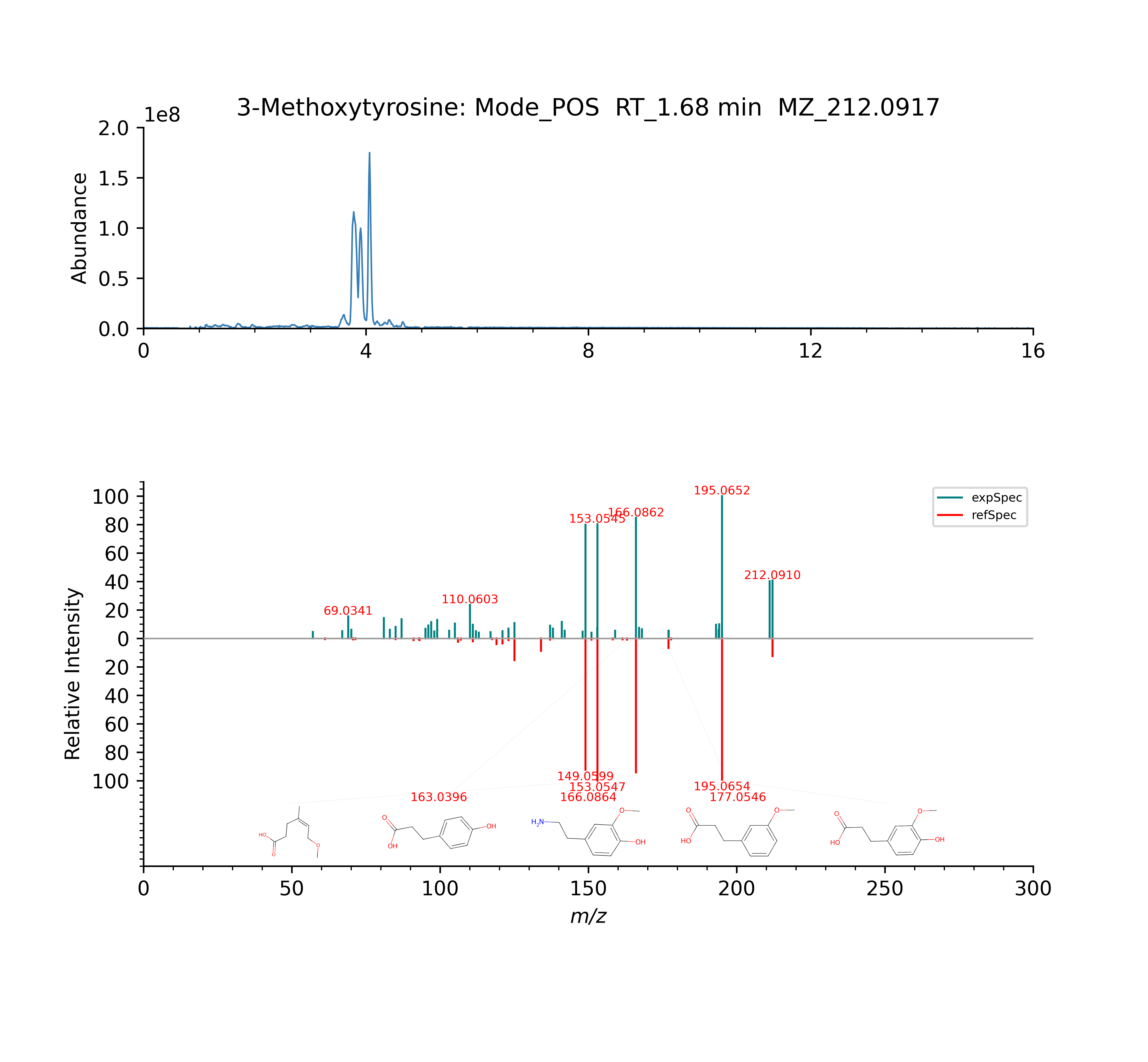

Supplement: Supplementary file 1 [file ijms-27-02203-s001.zip › ijms-4070482 Supplementary/Metabolite List Identified by LC-MS_MS from Rhodiola Species/65.png]

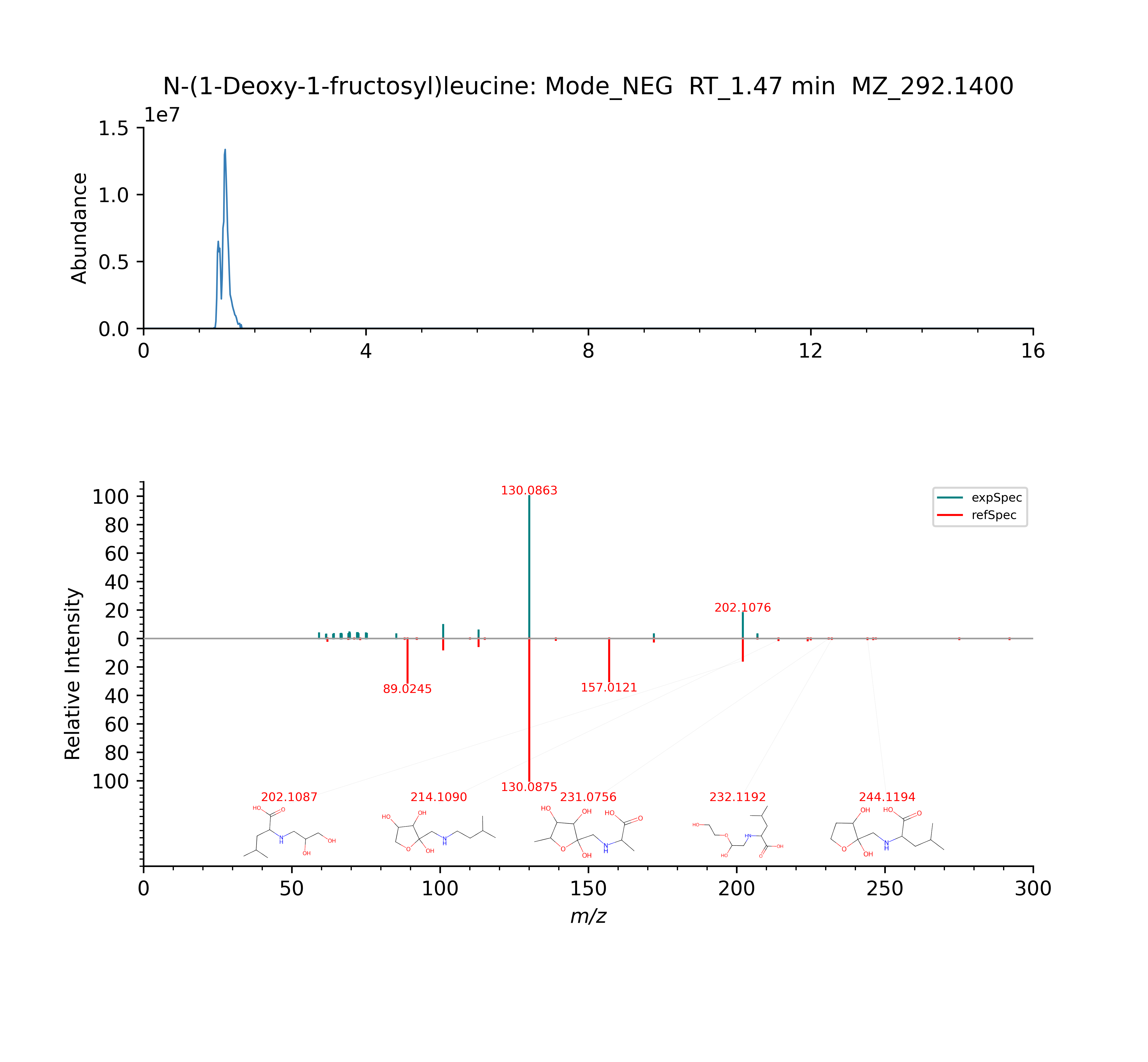

Supplement: Supplementary file 1 [file ijms-27-02203-s001.zip › ijms-4070482 Supplementary/Metabolite List Identified by LC-MS_MS from Rhodiola Species/66.png]

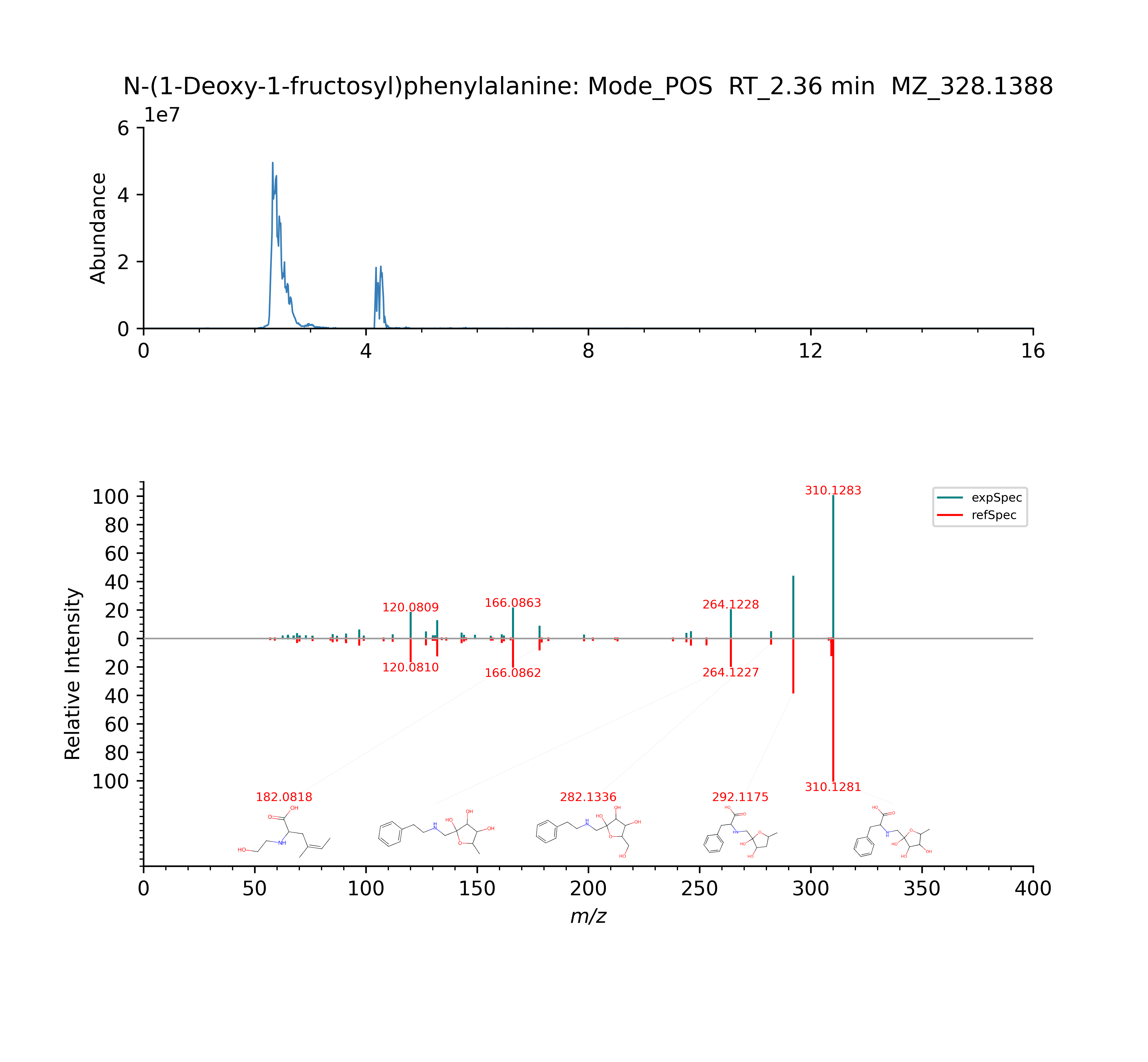

Supplement: Supplementary file 1 [file ijms-27-02203-s001.zip › ijms-4070482 Supplementary/Metabolite List Identified by LC-MS_MS from Rhodiola Species/67.png]

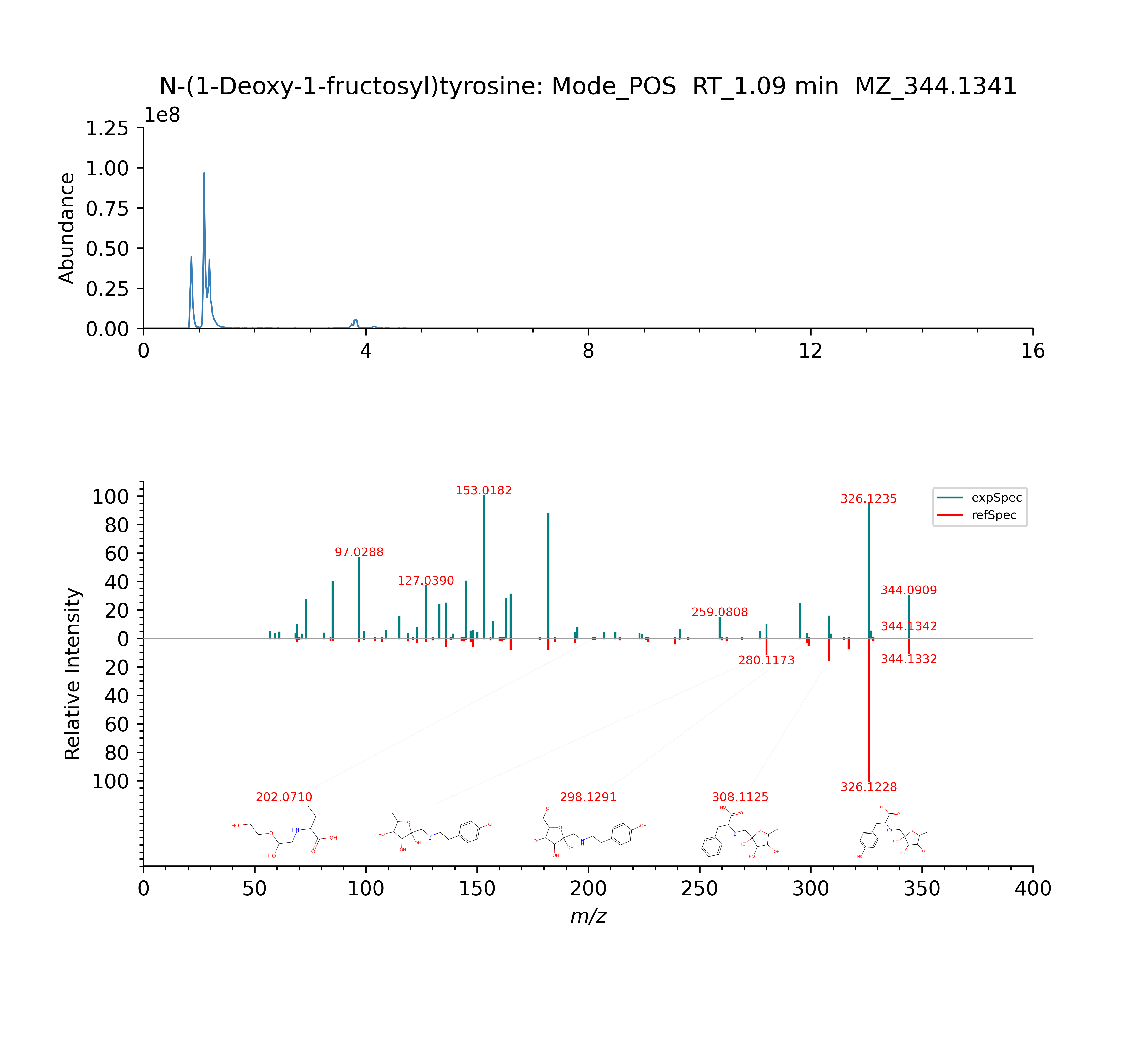

Supplement: Supplementary file 1 [file ijms-27-02203-s001.zip › ijms-4070482 Supplementary/Metabolite List Identified by LC-MS_MS from Rhodiola Species/68.png]

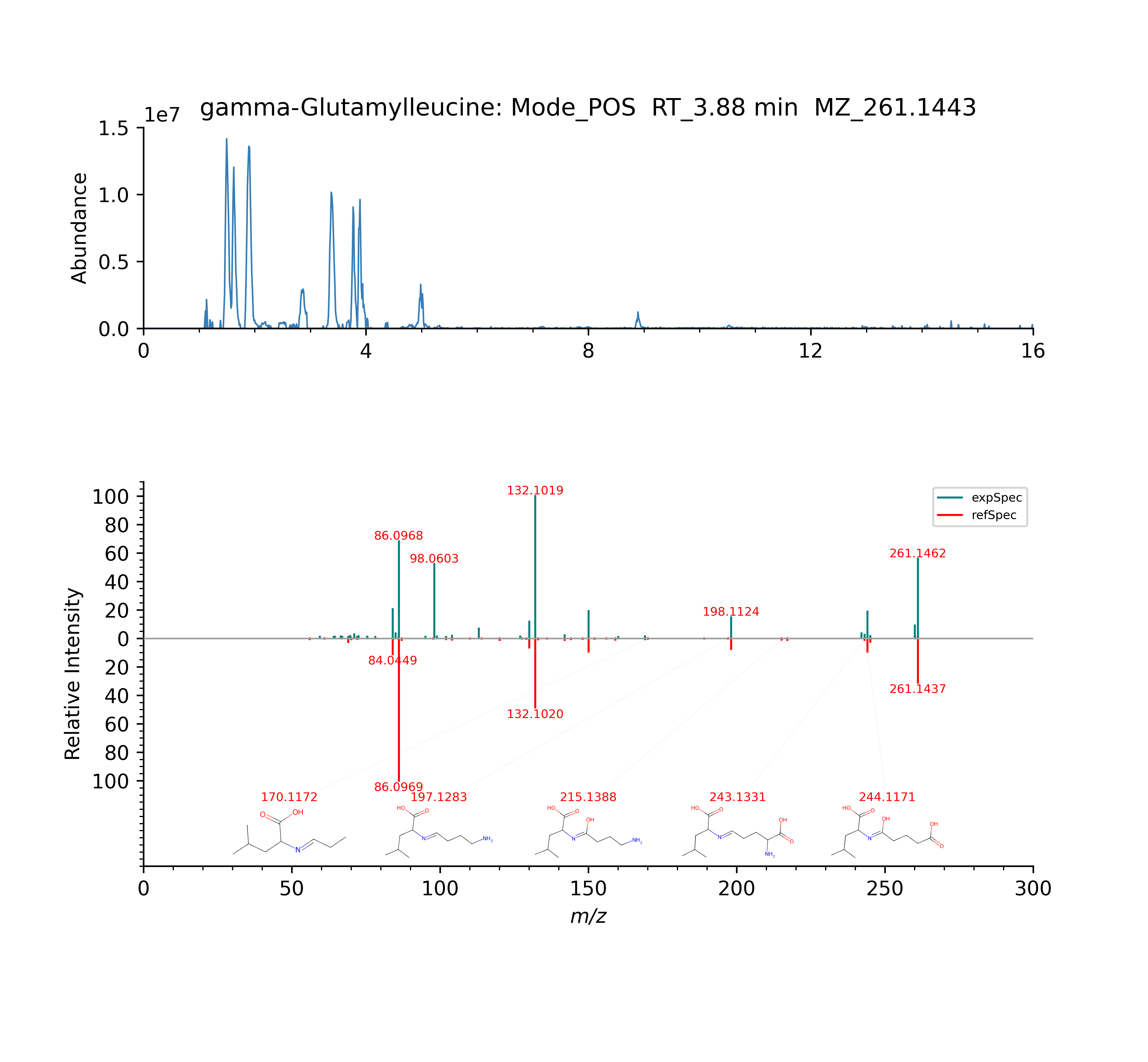

Supplement: Supplementary file 1 [file ijms-27-02203-s001.zip › ijms-4070482 Supplementary/Metabolite List Identified by LC-MS_MS from Rhodiola Species/69.png]

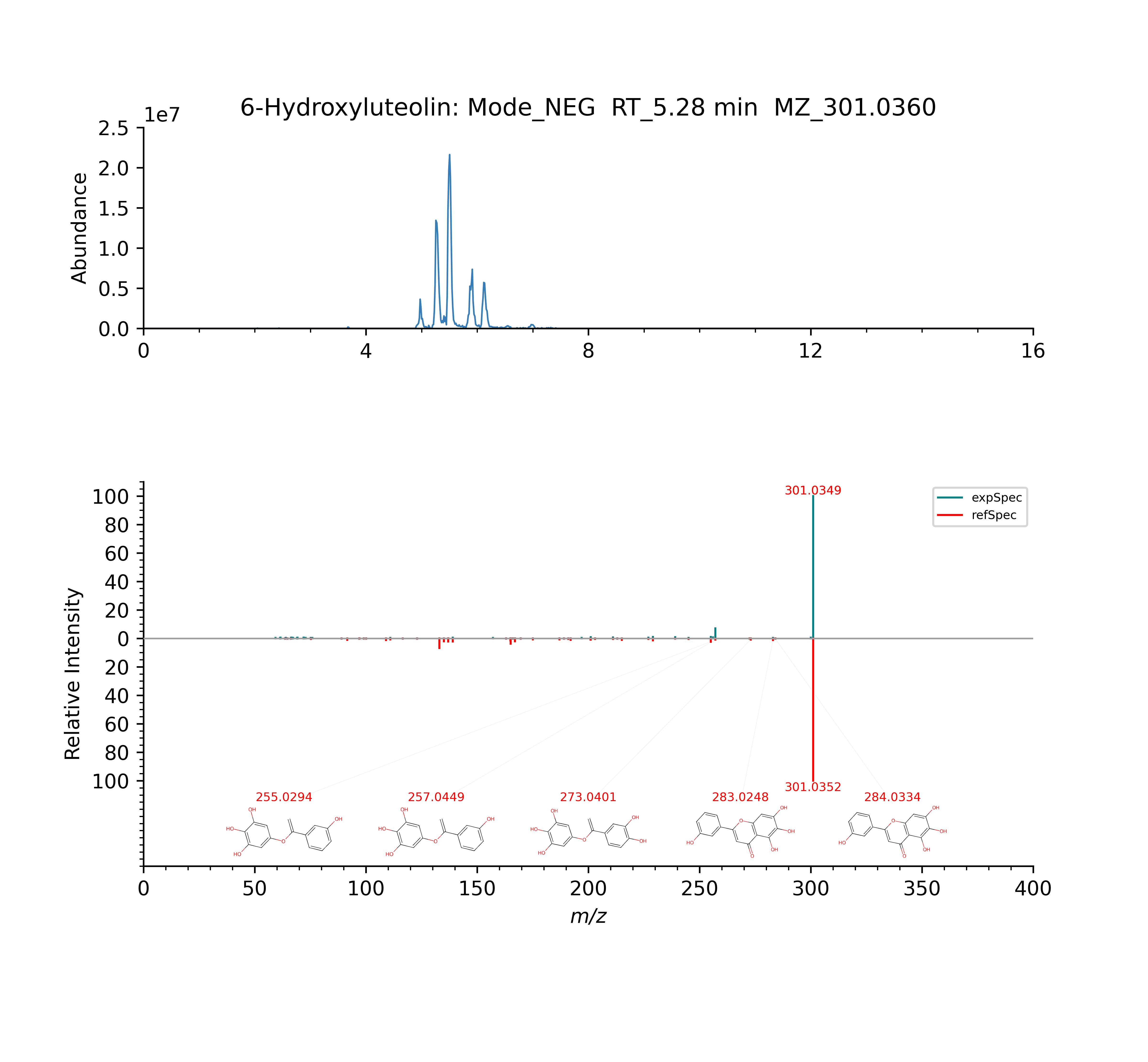

Supplement: Supplementary file 1 [file ijms-27-02203-s001.zip › ijms-4070482 Supplementary/Metabolite List Identified by LC-MS_MS from Rhodiola Species/7.png]

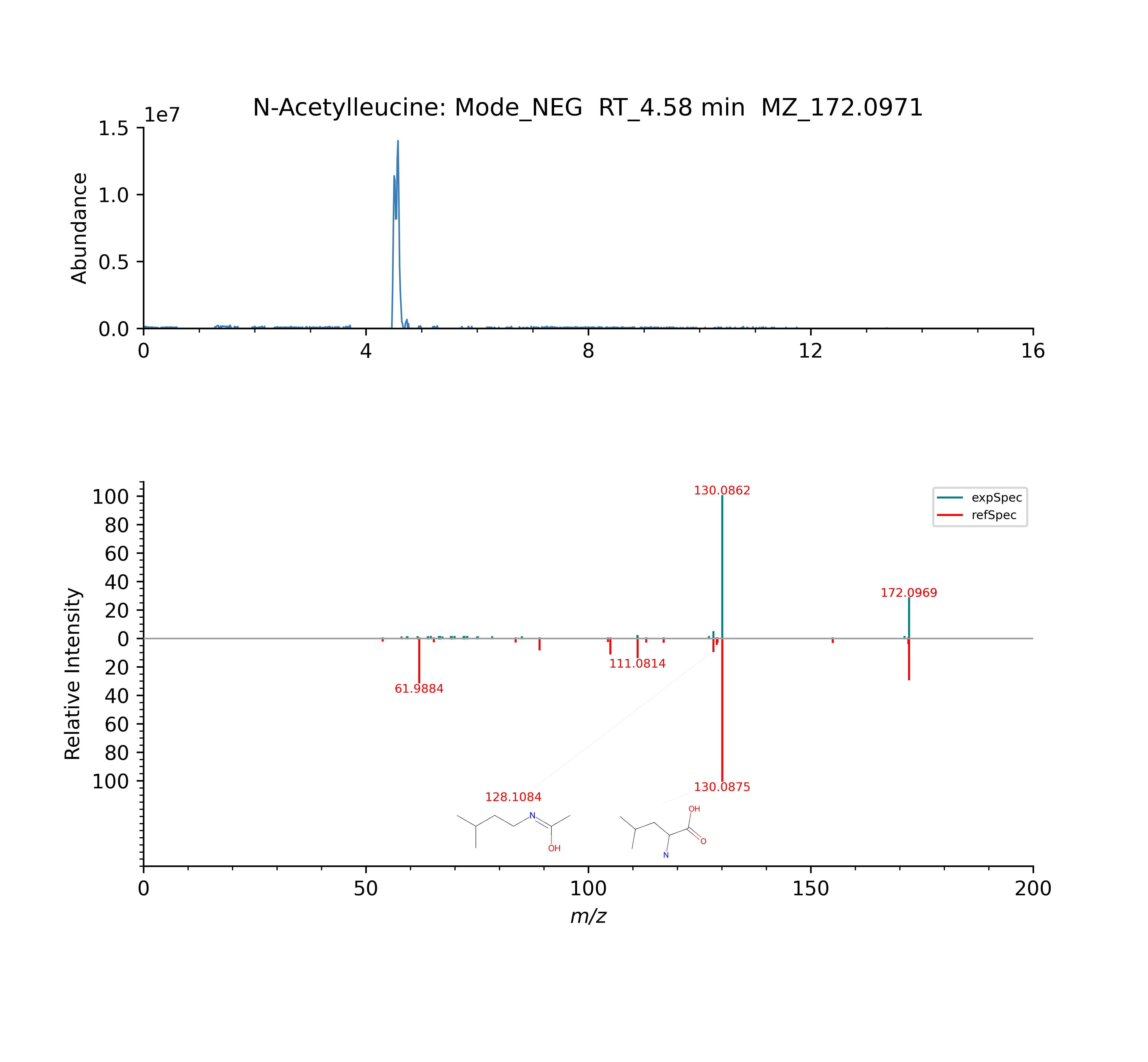

Supplement: Supplementary file 1 [file ijms-27-02203-s001.zip › ijms-4070482 Supplementary/Metabolite List Identified by LC-MS_MS from Rhodiola Species/70.png]

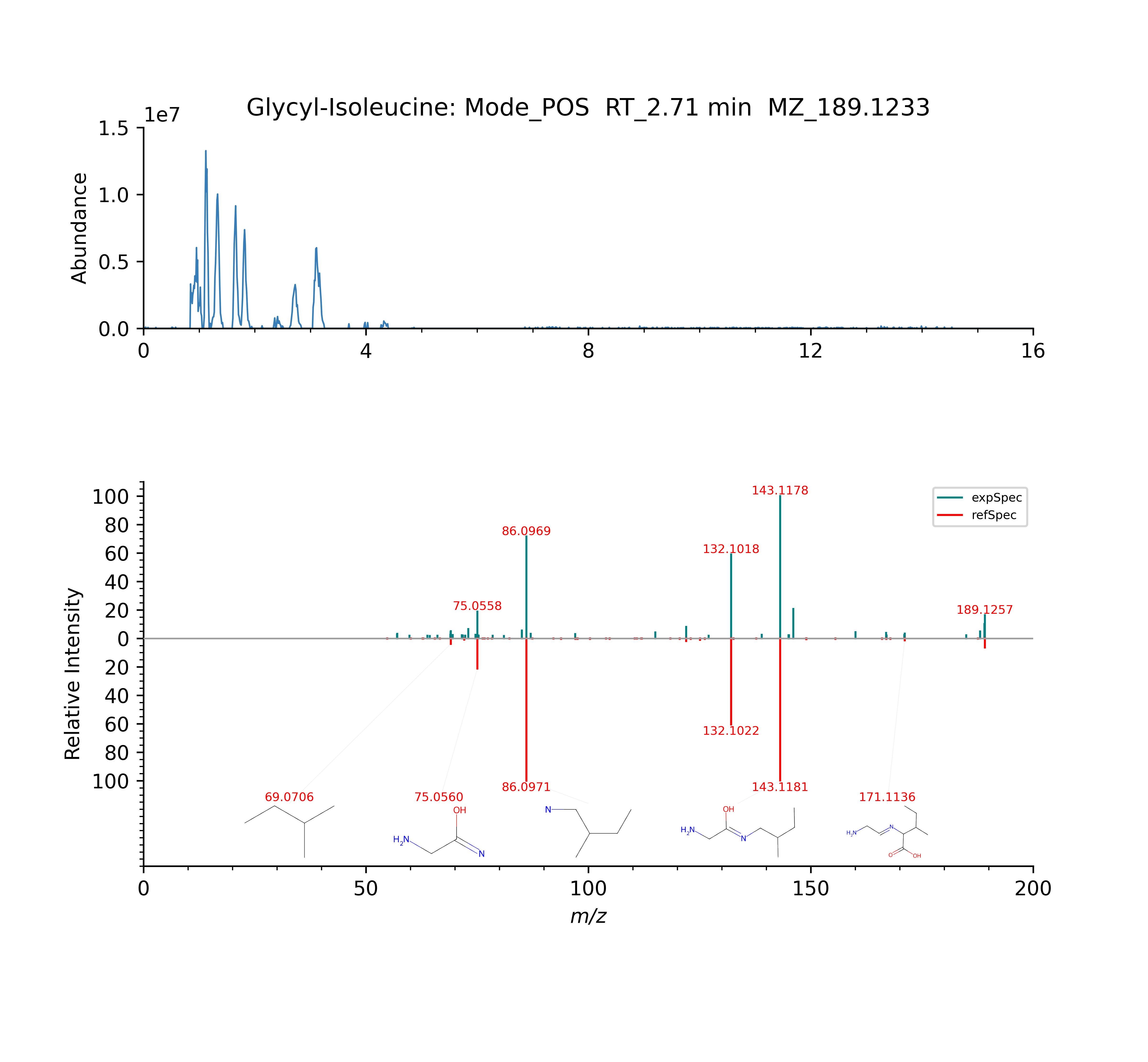

Supplement: Supplementary file 1 [file ijms-27-02203-s001.zip › ijms-4070482 Supplementary/Metabolite List Identified by LC-MS_MS from Rhodiola Species/71.png]

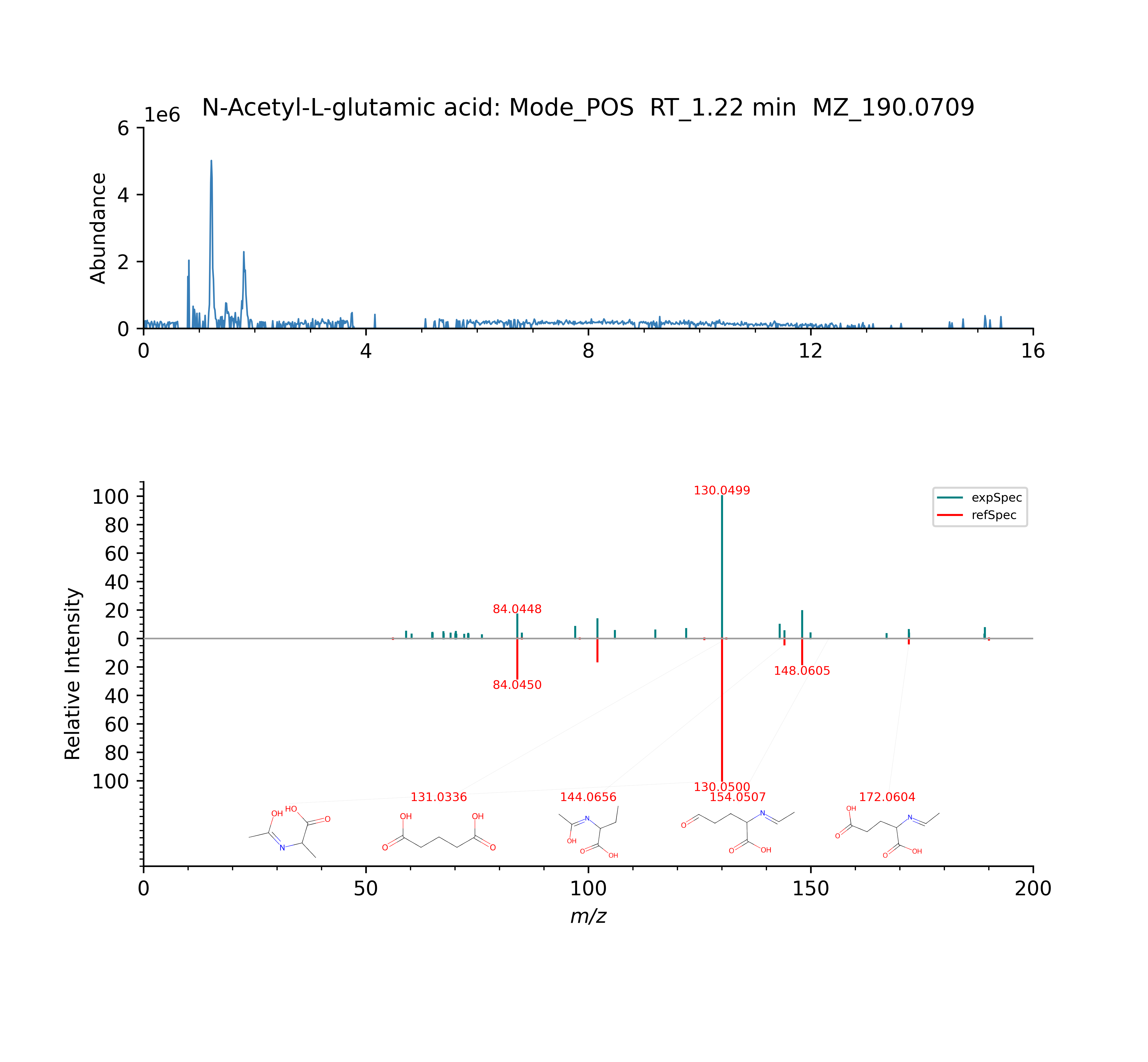

Supplement: Supplementary file 1 [file ijms-27-02203-s001.zip › ijms-4070482 Supplementary/Metabolite List Identified by LC-MS_MS from Rhodiola Species/72.png]

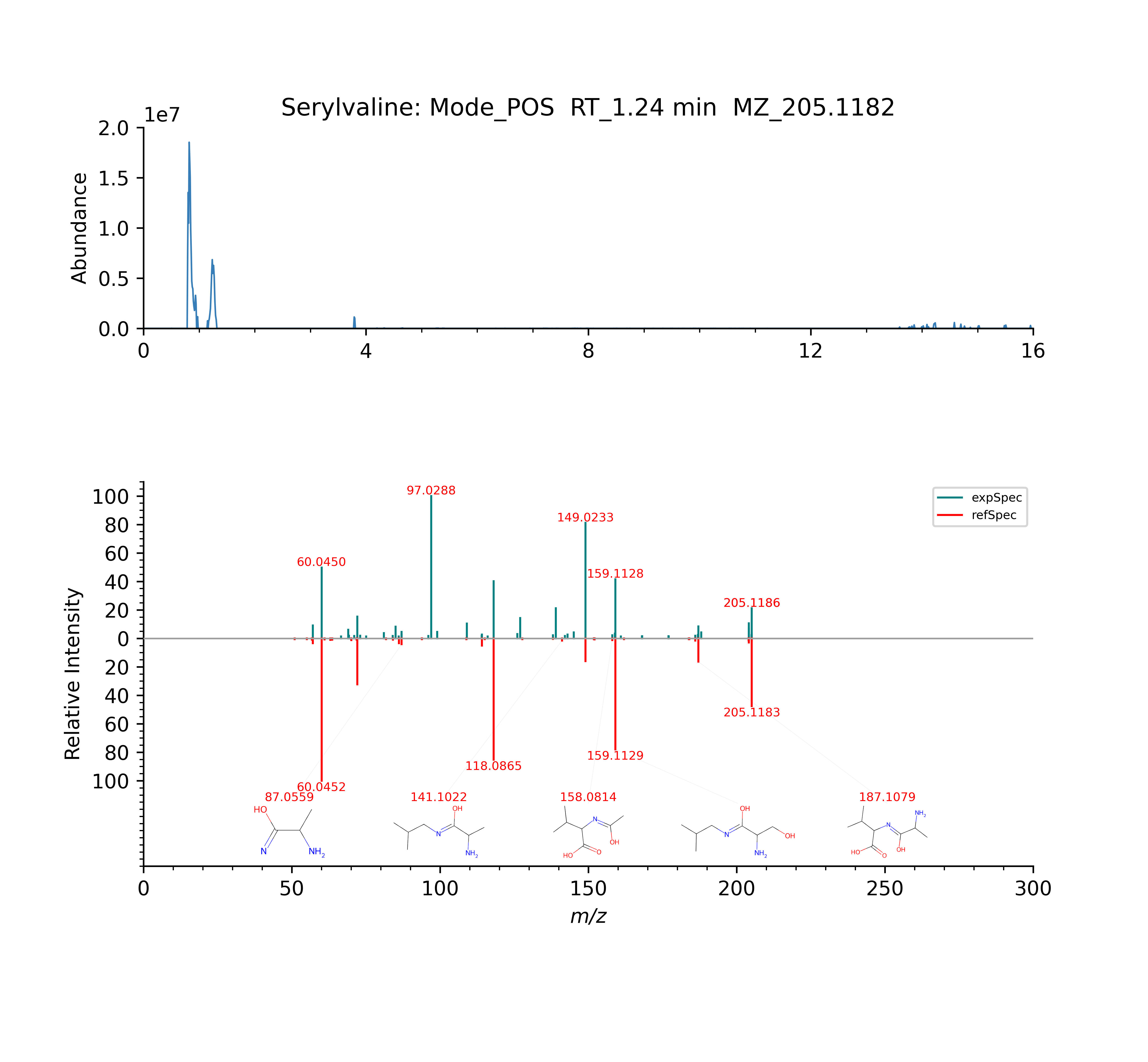

Supplement: Supplementary file 1 [file ijms-27-02203-s001.zip › ijms-4070482 Supplementary/Metabolite List Identified by LC-MS_MS from Rhodiola Species/73.png]

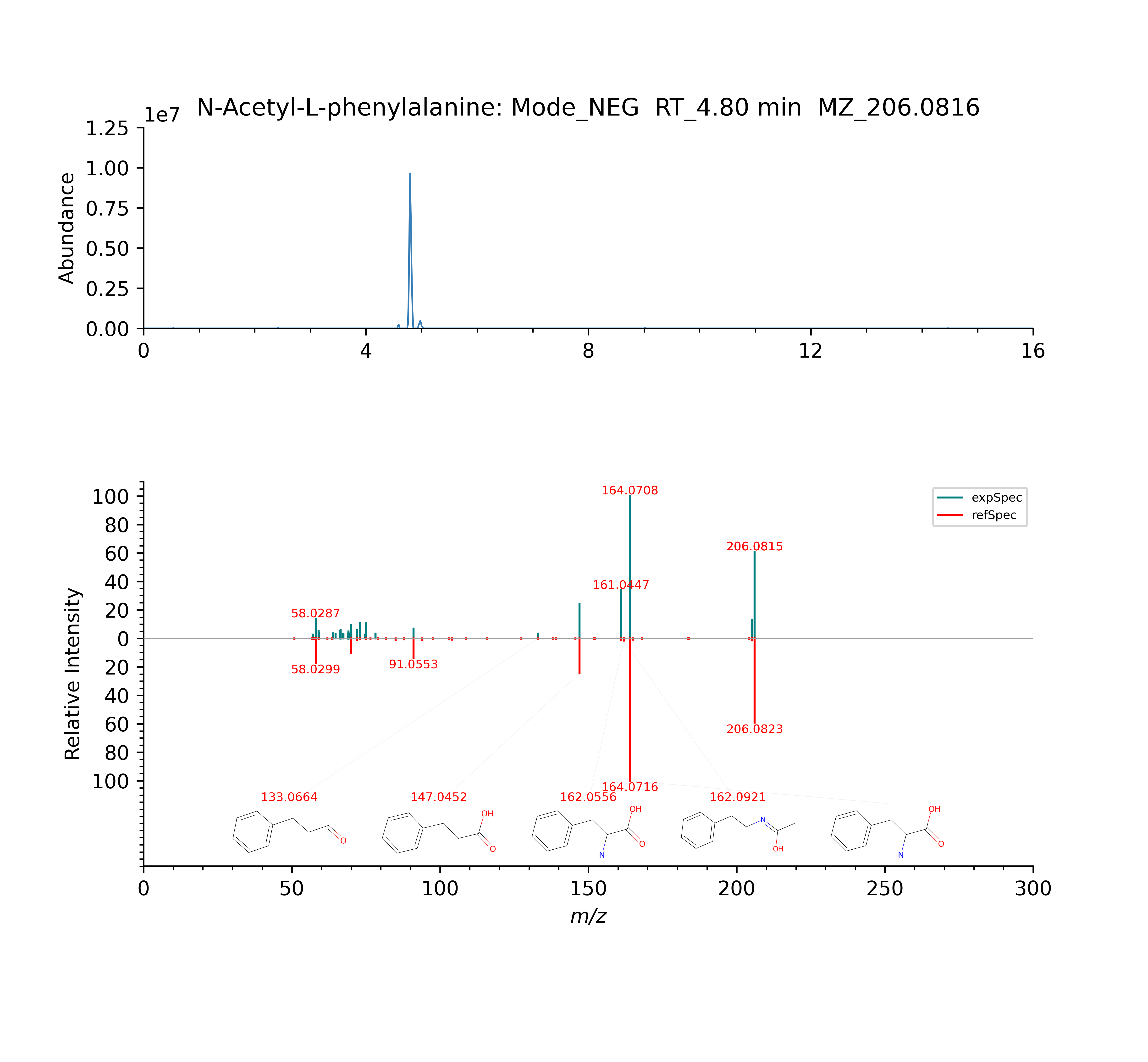

Supplement: Supplementary file 1 [file ijms-27-02203-s001.zip › ijms-4070482 Supplementary/Metabolite List Identified by LC-MS_MS from Rhodiola Species/74.png]

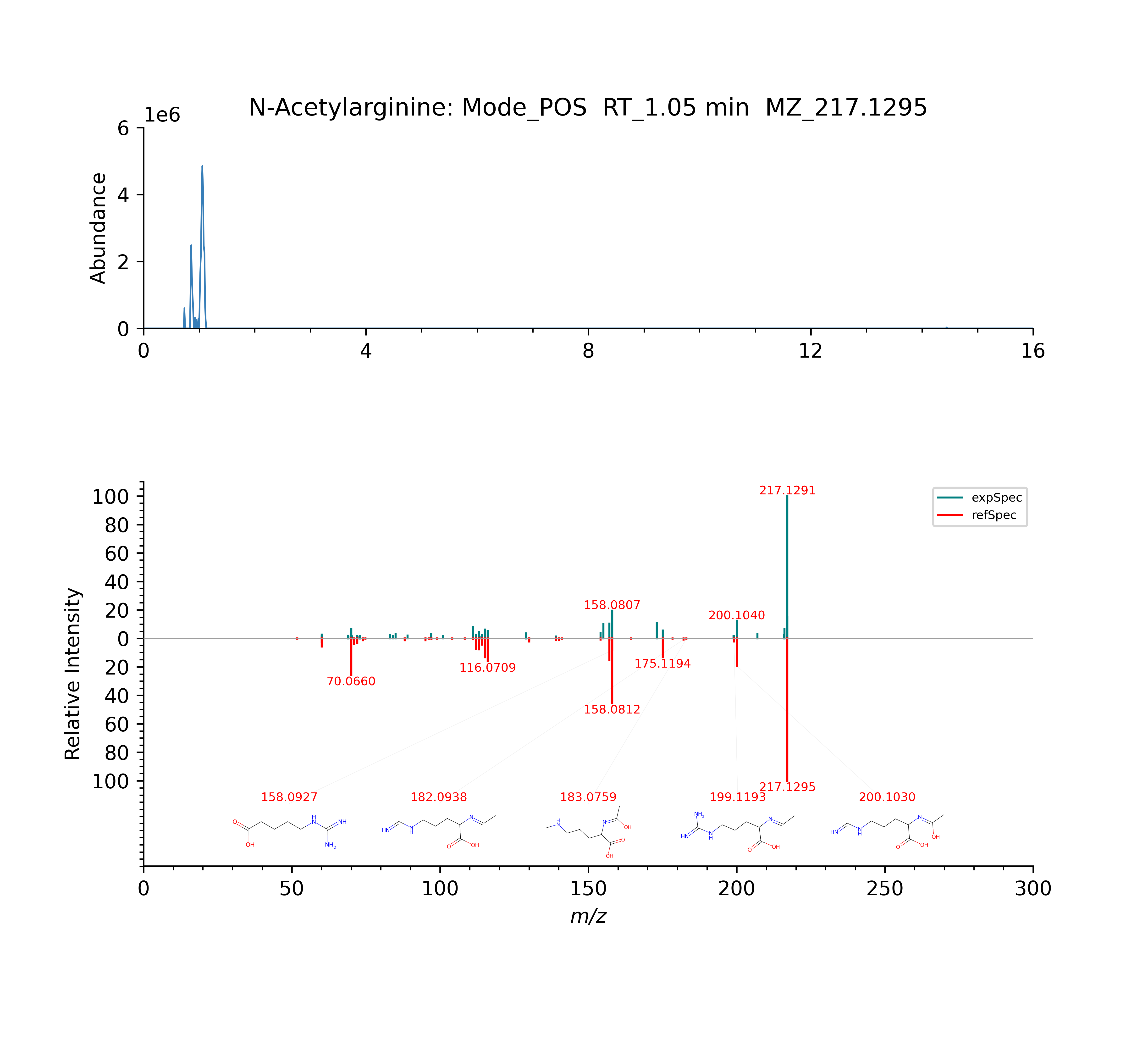

Supplement: Supplementary file 1 [file ijms-27-02203-s001.zip › ijms-4070482 Supplementary/Metabolite List Identified by LC-MS_MS from Rhodiola Species/75.png]

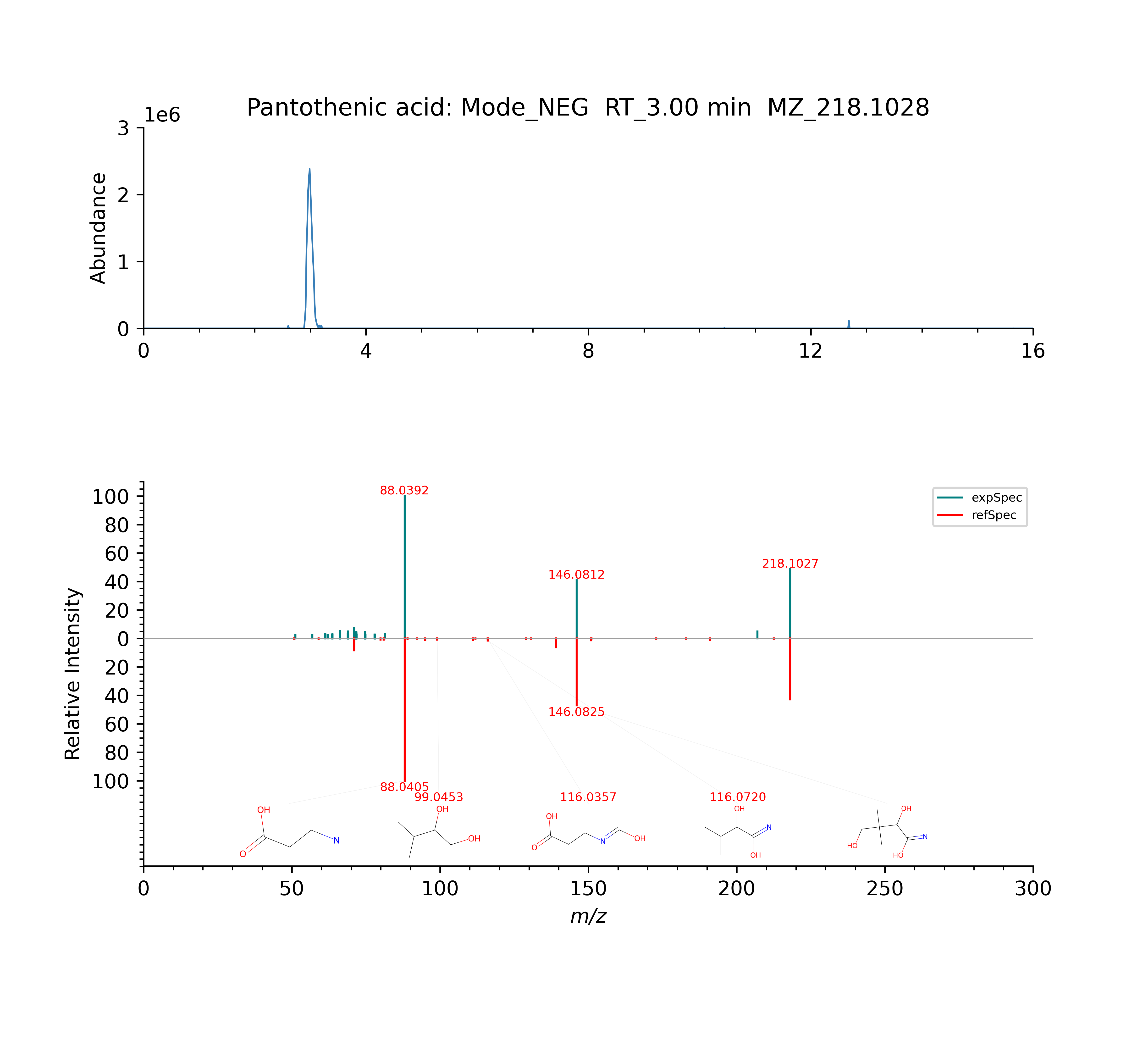

Supplement: Supplementary file 1 [file ijms-27-02203-s001.zip › ijms-4070482 Supplementary/Metabolite List Identified by LC-MS_MS from Rhodiola Species/76.png]

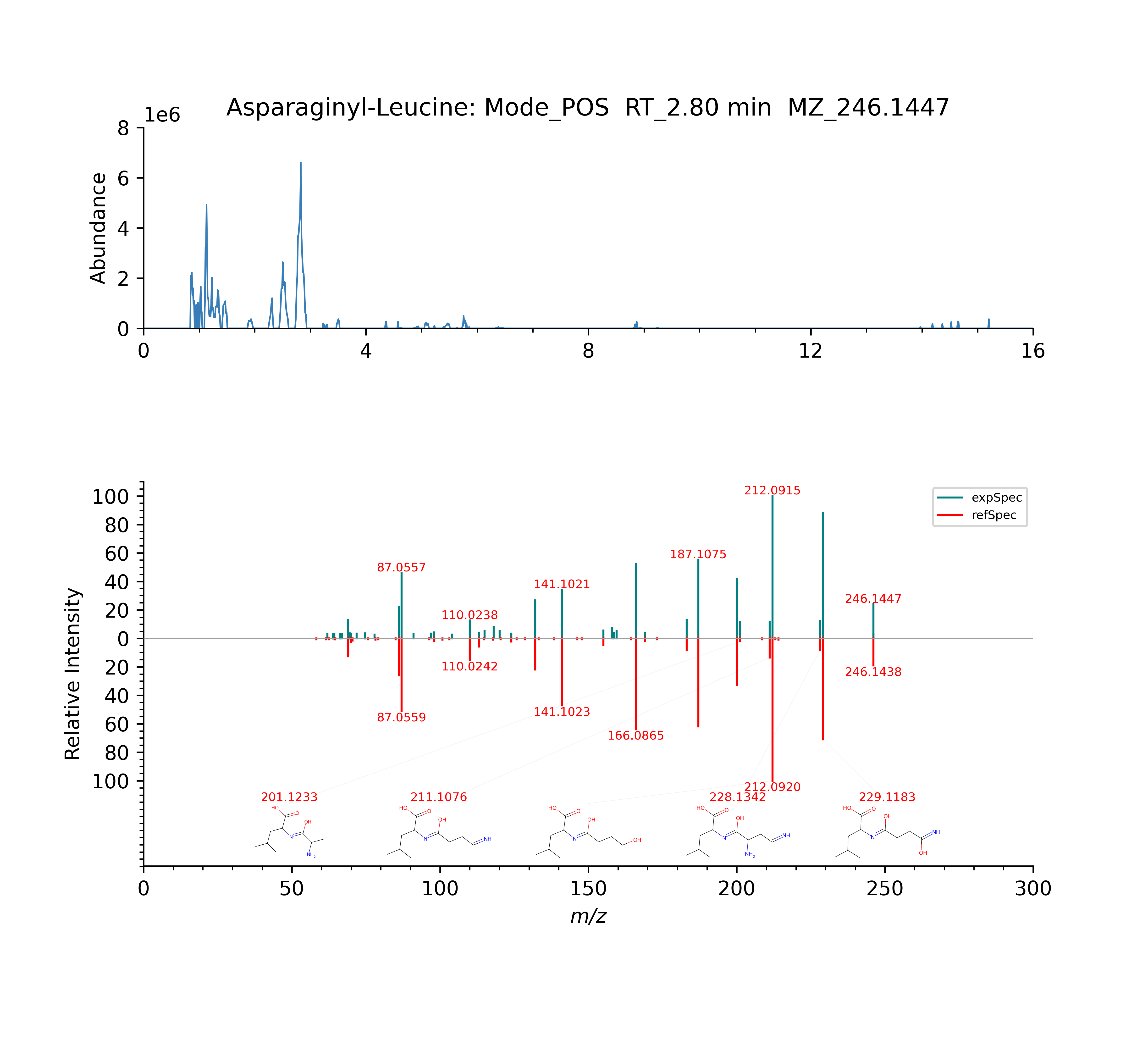

Supplement: Supplementary file 1 [file ijms-27-02203-s001.zip › ijms-4070482 Supplementary/Metabolite List Identified by LC-MS_MS from Rhodiola Species/77.png]

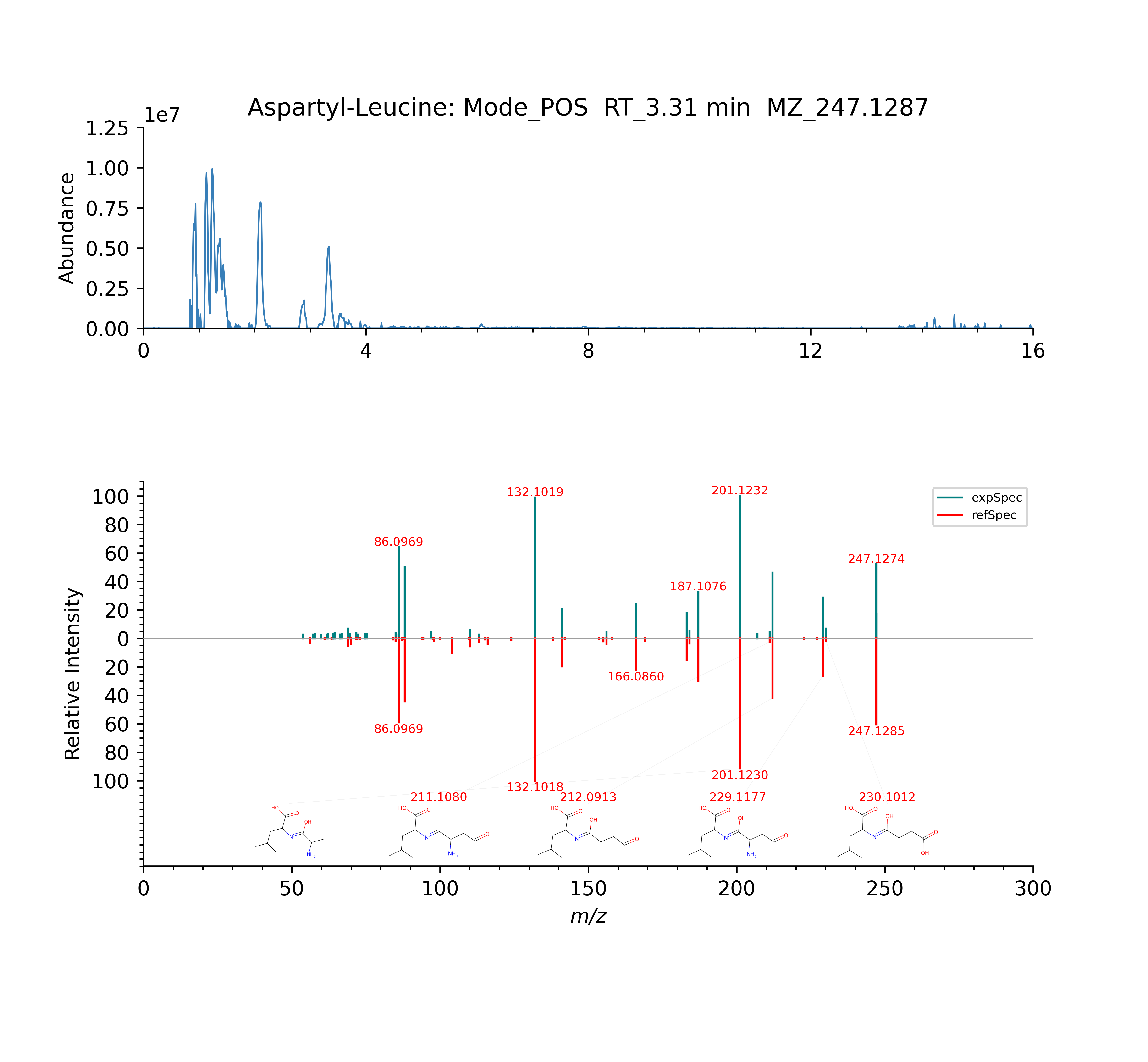

Supplement: Supplementary file 1 [file ijms-27-02203-s001.zip › ijms-4070482 Supplementary/Metabolite List Identified by LC-MS_MS from Rhodiola Species/78.png]

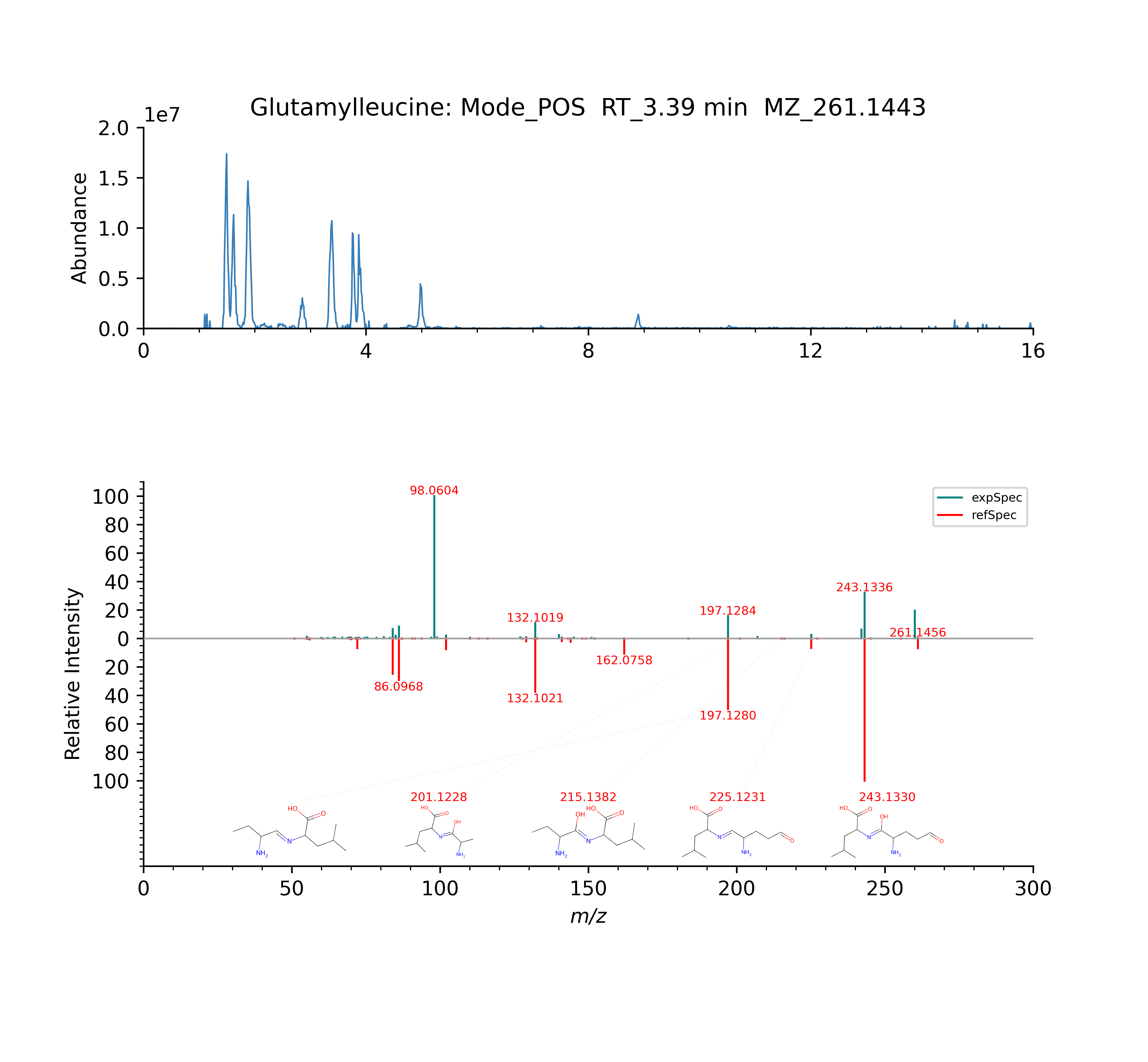

Supplement: Supplementary file 1 [file ijms-27-02203-s001.zip › ijms-4070482 Supplementary/Metabolite List Identified by LC-MS_MS from Rhodiola Species/79.png]

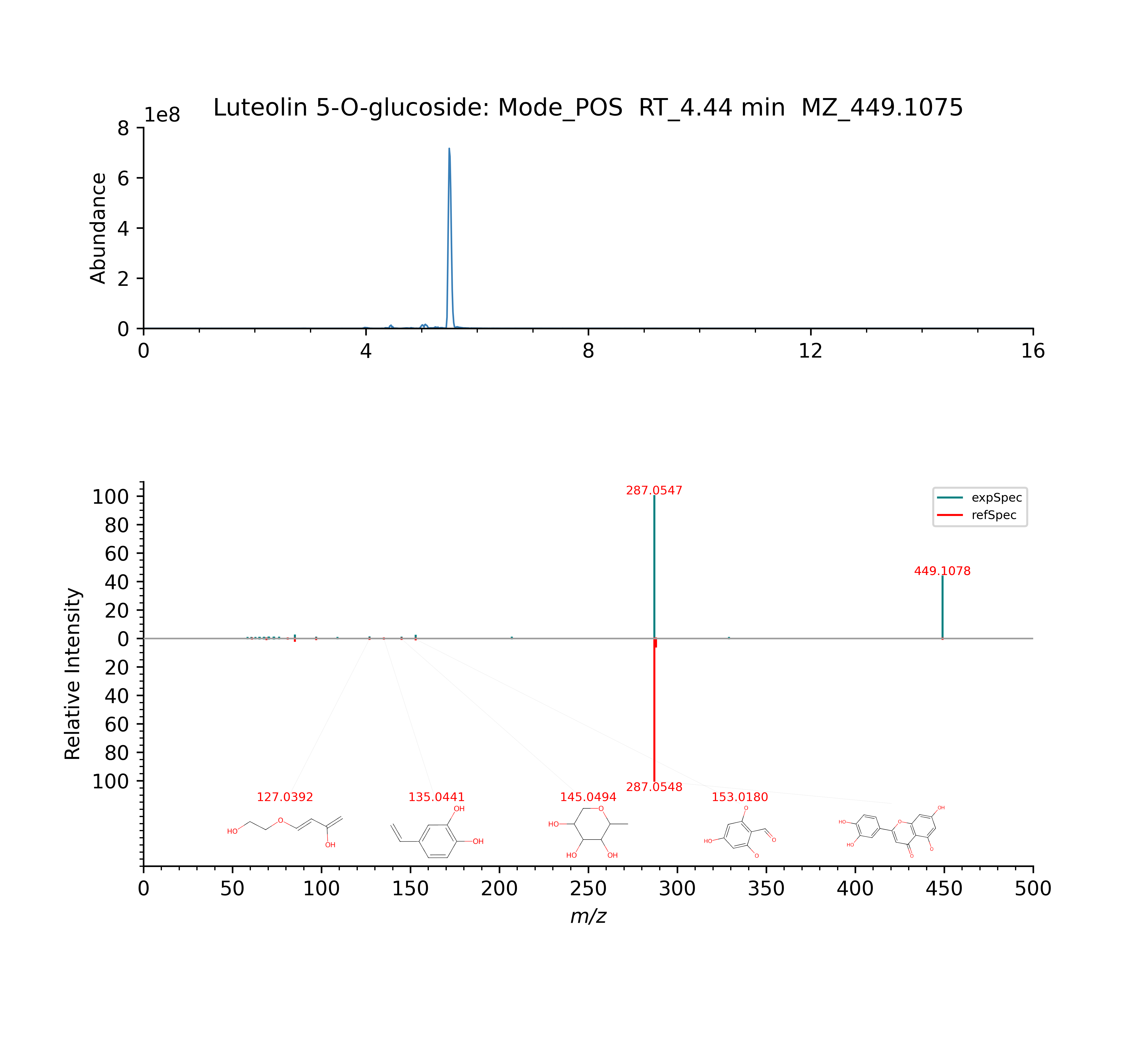

Supplement: Supplementary file 1 [file ijms-27-02203-s001.zip › ijms-4070482 Supplementary/Metabolite List Identified by LC-MS_MS from Rhodiola Species/8.png]

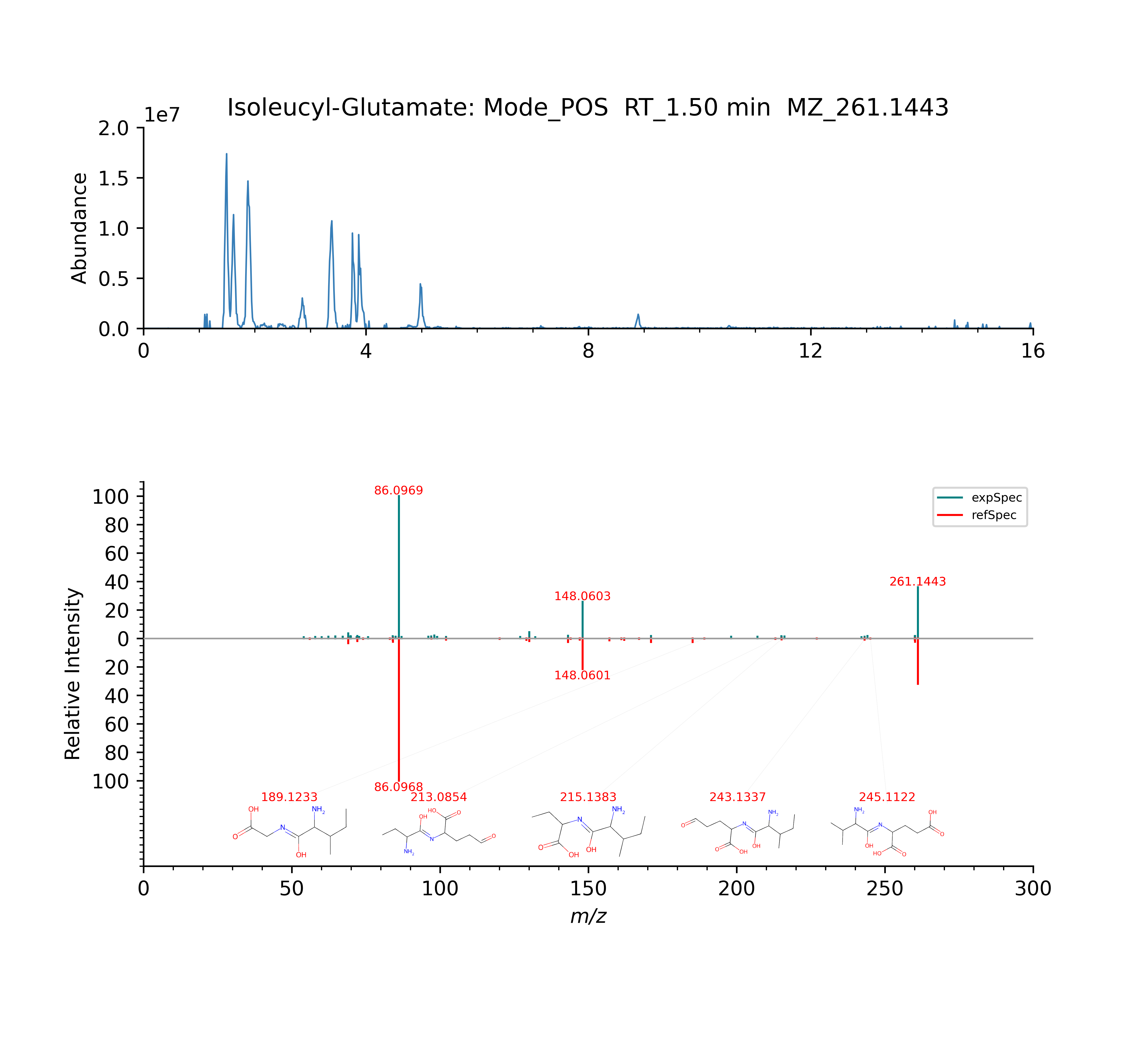

Supplement: Supplementary file 1 [file ijms-27-02203-s001.zip › ijms-4070482 Supplementary/Metabolite List Identified by LC-MS_MS from Rhodiola Species/80.png]

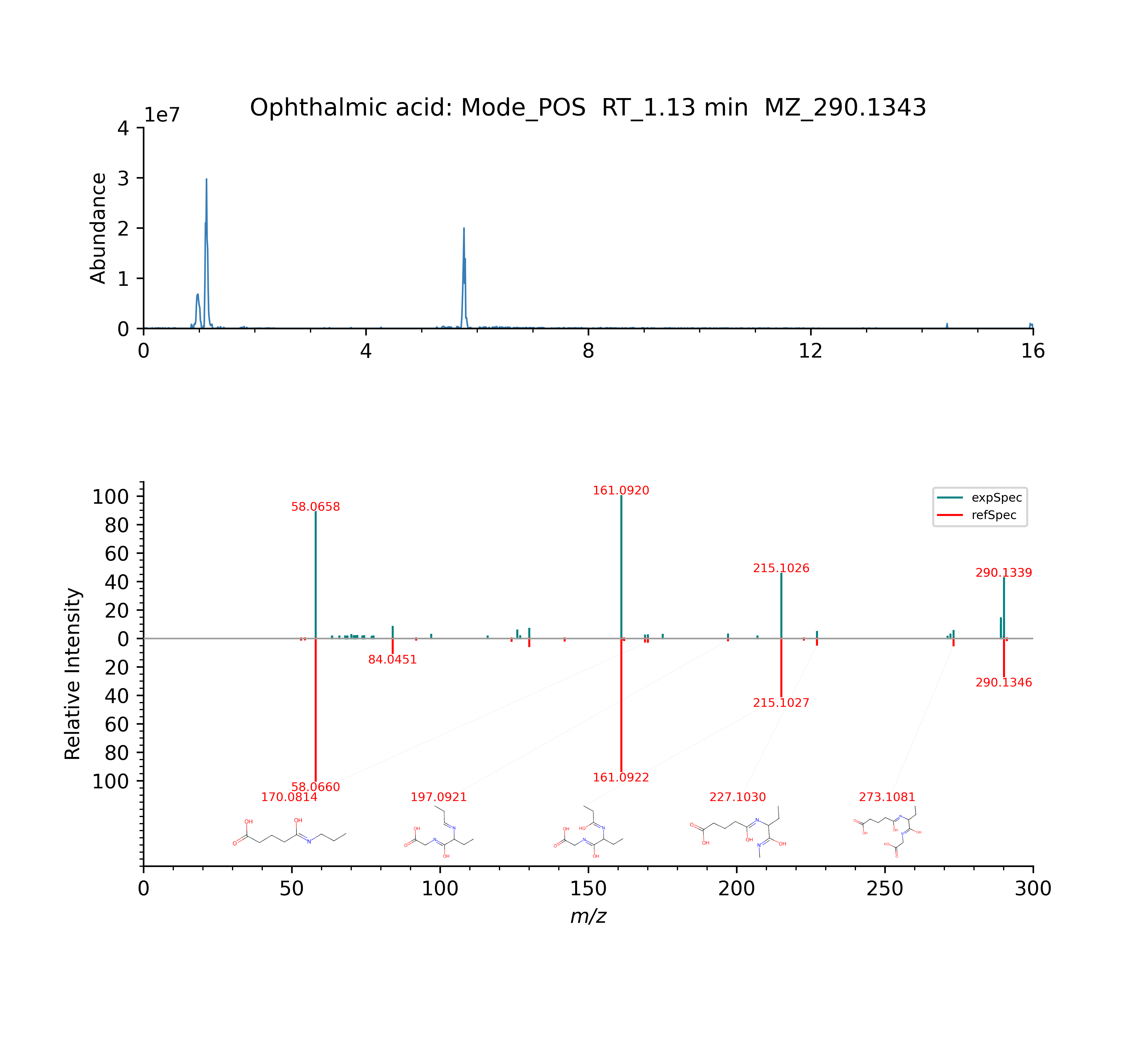

Supplement: Supplementary file 1 [file ijms-27-02203-s001.zip › ijms-4070482 Supplementary/Metabolite List Identified by LC-MS_MS from Rhodiola Species/81.png]

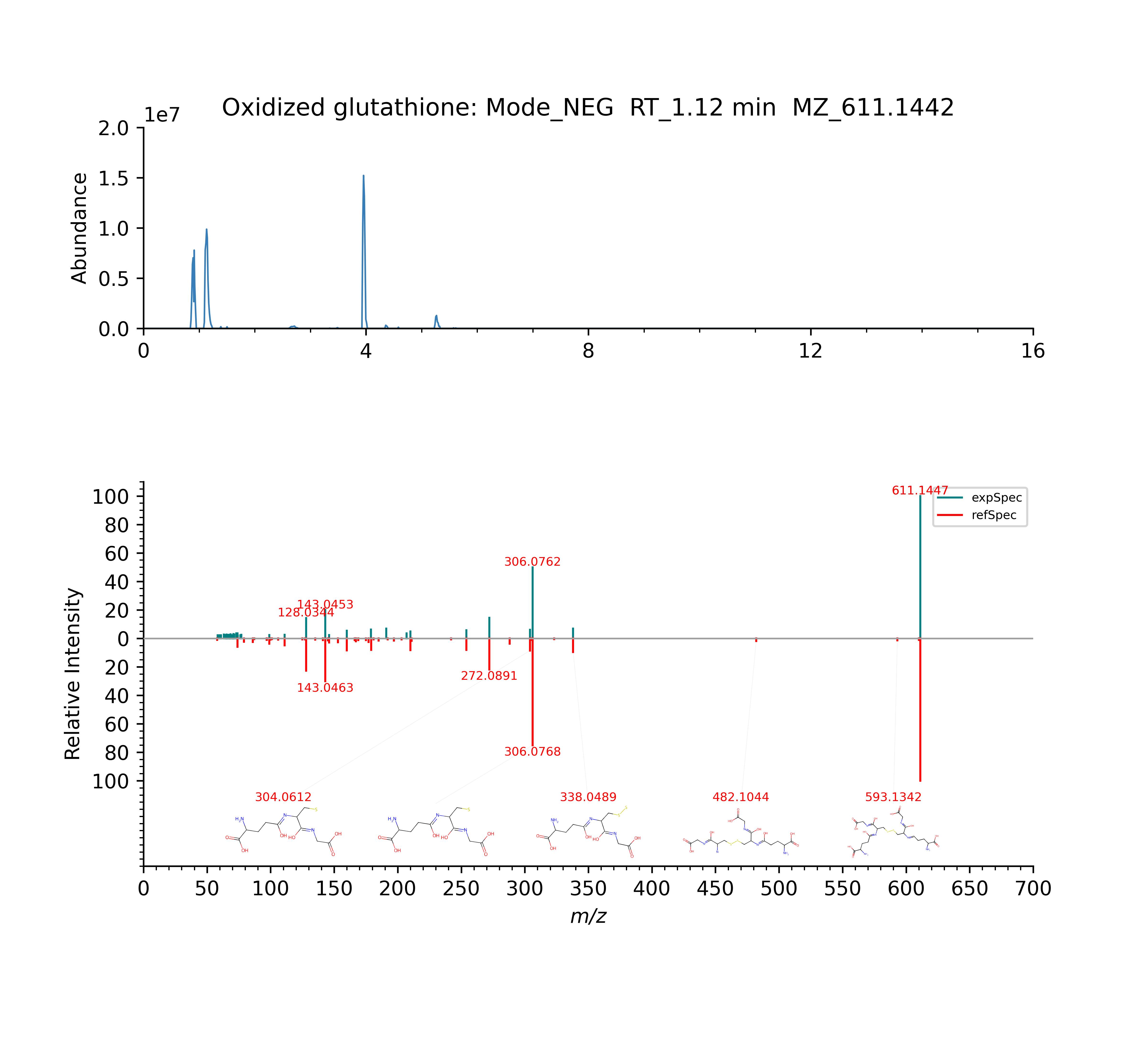

Supplement: Supplementary file 1 [file ijms-27-02203-s001.zip › ijms-4070482 Supplementary/Metabolite List Identified by LC-MS_MS from Rhodiola Species/82.png]

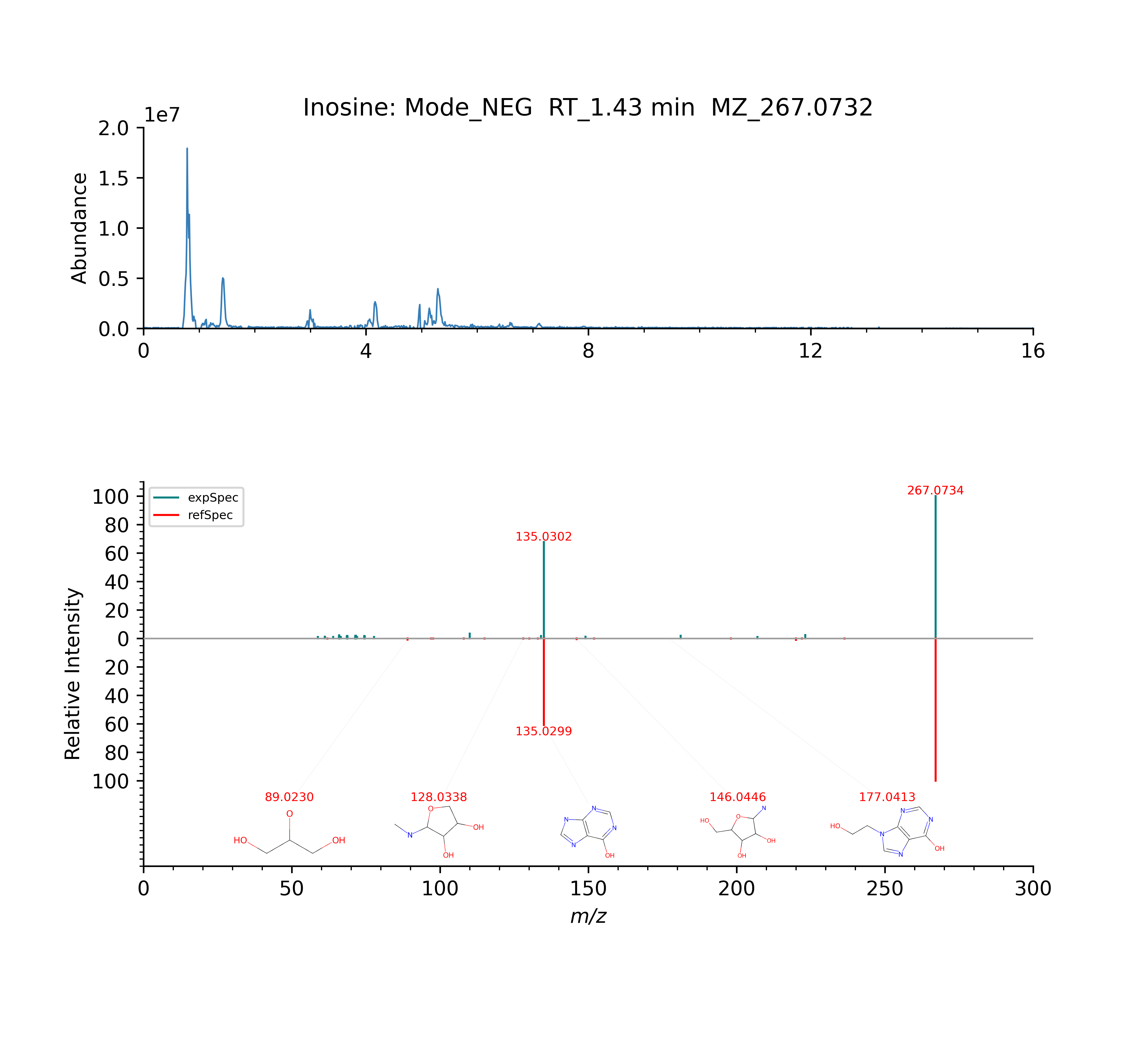

Supplement: Supplementary file 1 [file ijms-27-02203-s001.zip › ijms-4070482 Supplementary/Metabolite List Identified by LC-MS_MS from Rhodiola Species/83.png]

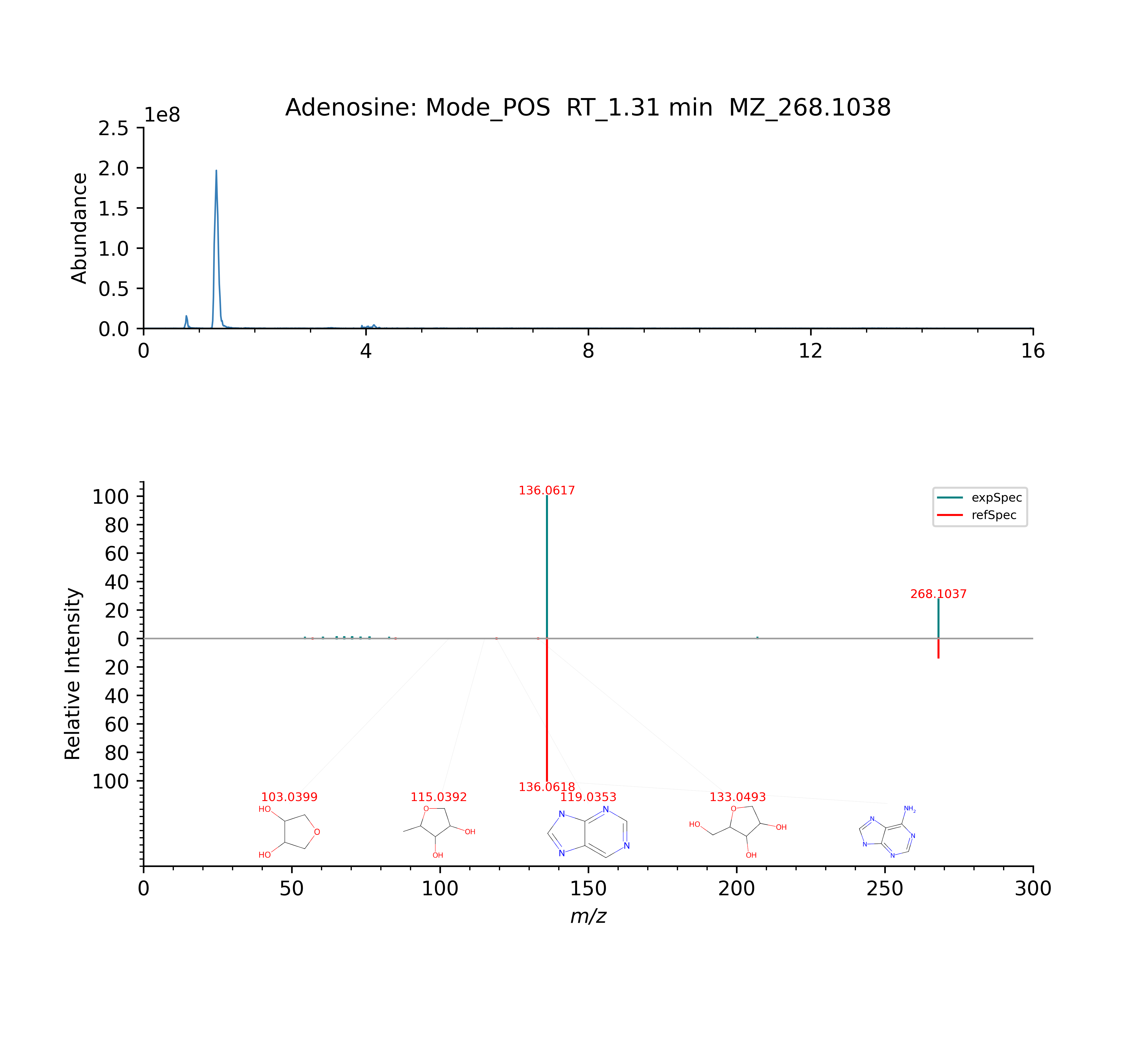

Supplement: Supplementary file 1 [file ijms-27-02203-s001.zip › ijms-4070482 Supplementary/Metabolite List Identified by LC-MS_MS from Rhodiola Species/84.png]

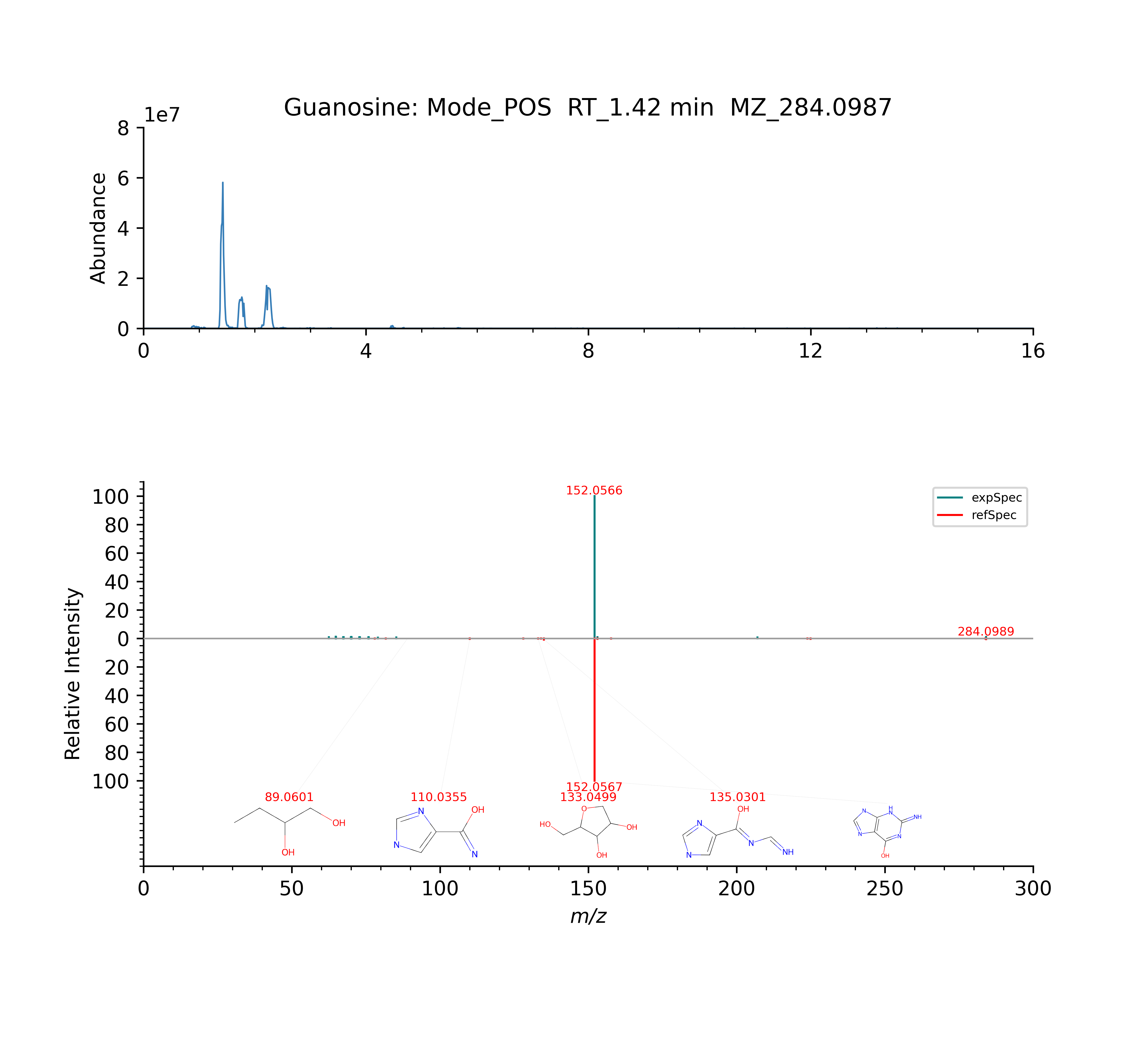

Supplement: Supplementary file 1 [file ijms-27-02203-s001.zip › ijms-4070482 Supplementary/Metabolite List Identified by LC-MS_MS from Rhodiola Species/85.png]

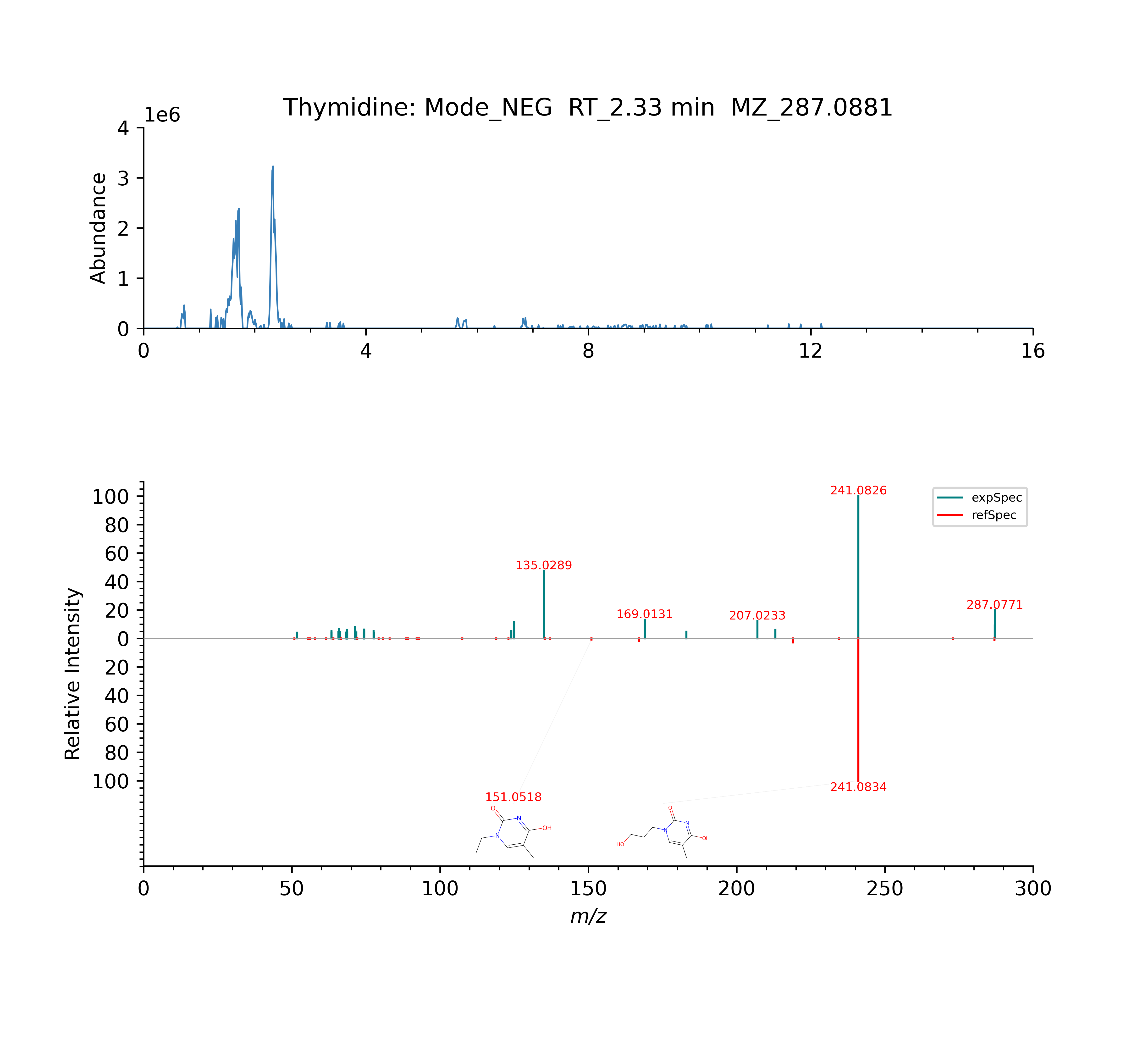

Supplement: Supplementary file 1 [file ijms-27-02203-s001.zip › ijms-4070482 Supplementary/Metabolite List Identified by LC-MS_MS from Rhodiola Species/86.png]

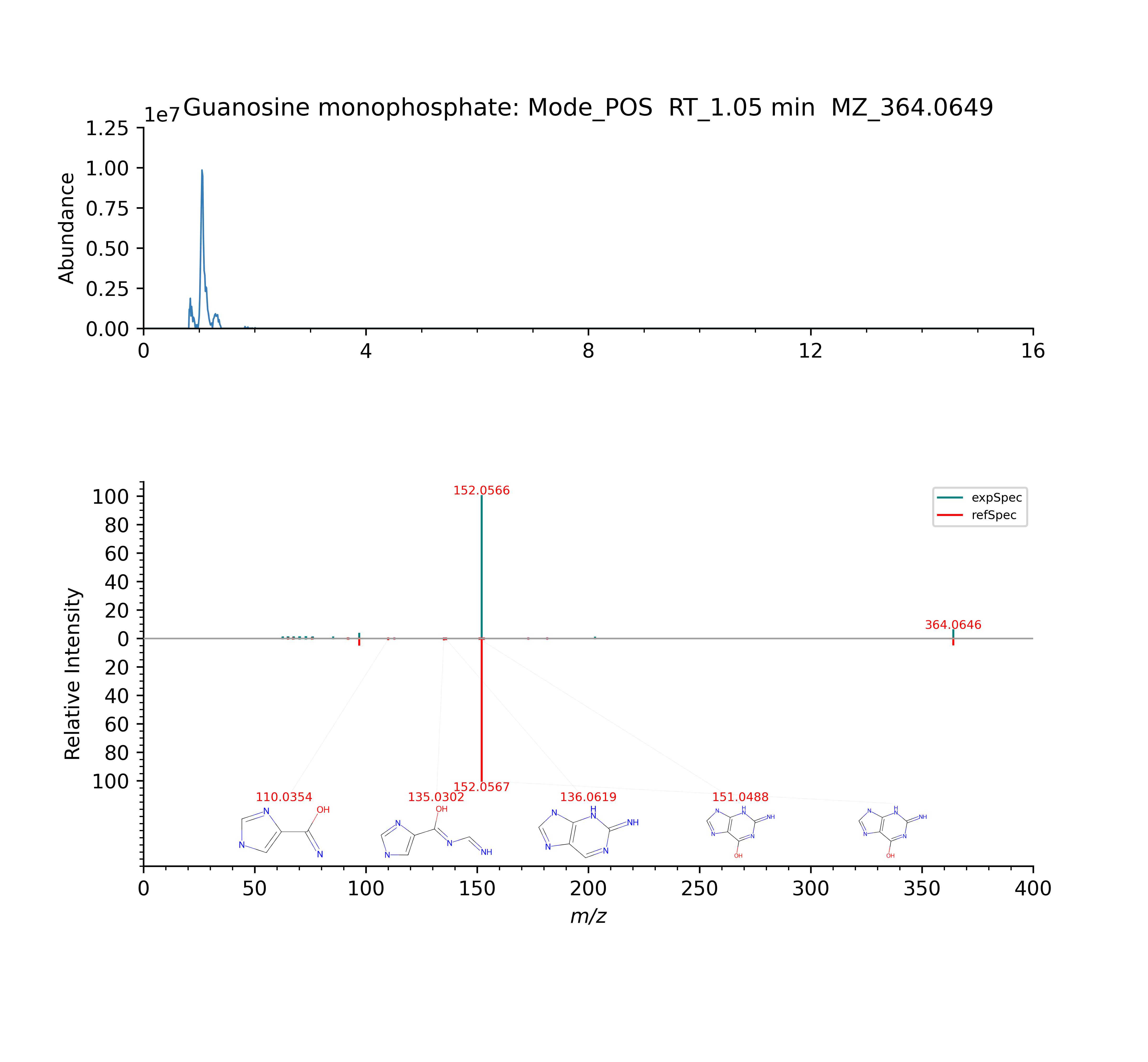

Supplement: Supplementary file 1 [file ijms-27-02203-s001.zip › ijms-4070482 Supplementary/Metabolite List Identified by LC-MS_MS from Rhodiola Species/87.png]

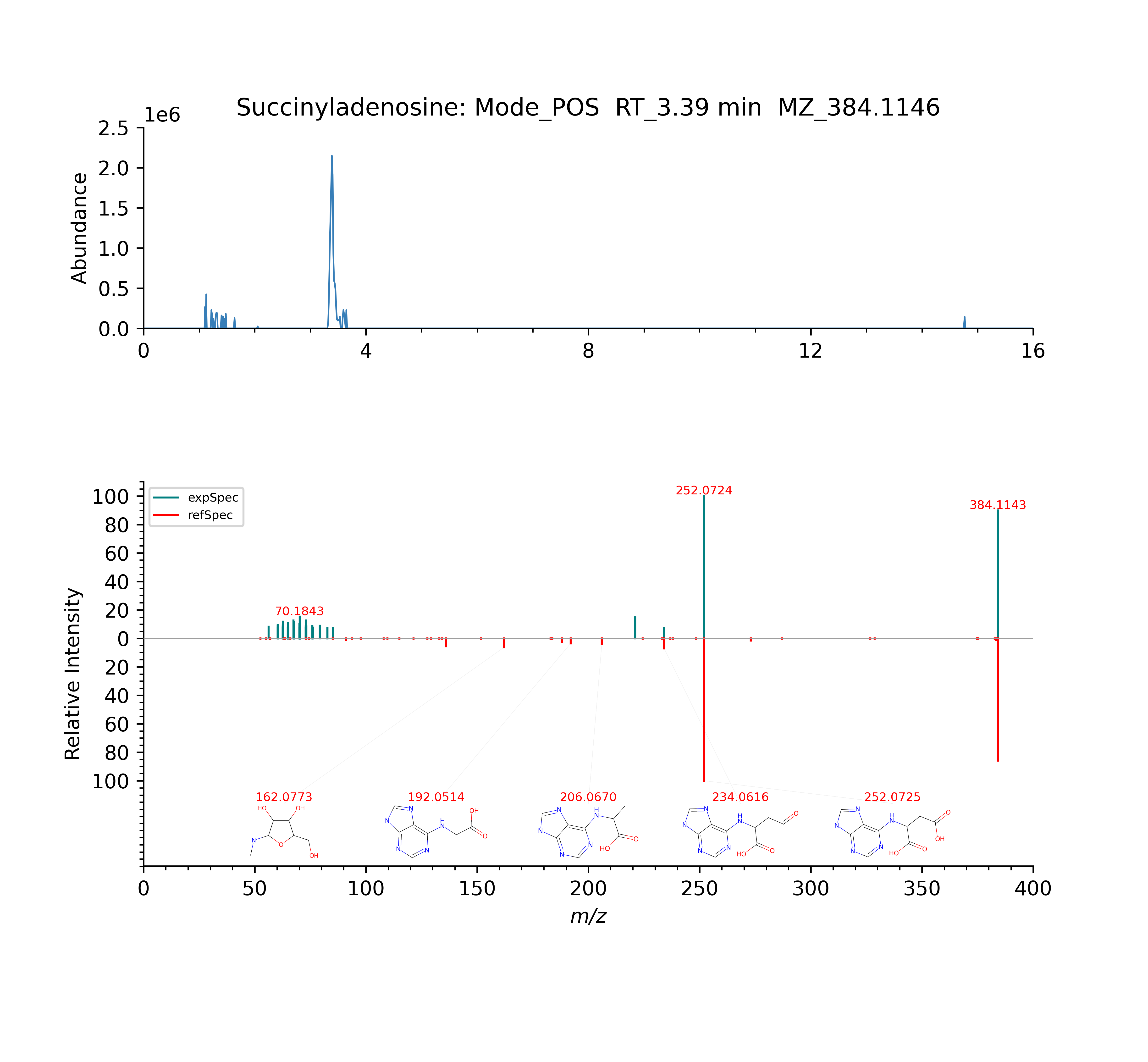

Supplement: Supplementary file 1 [file ijms-27-02203-s001.zip › ijms-4070482 Supplementary/Metabolite List Identified by LC-MS_MS from Rhodiola Species/88.png]

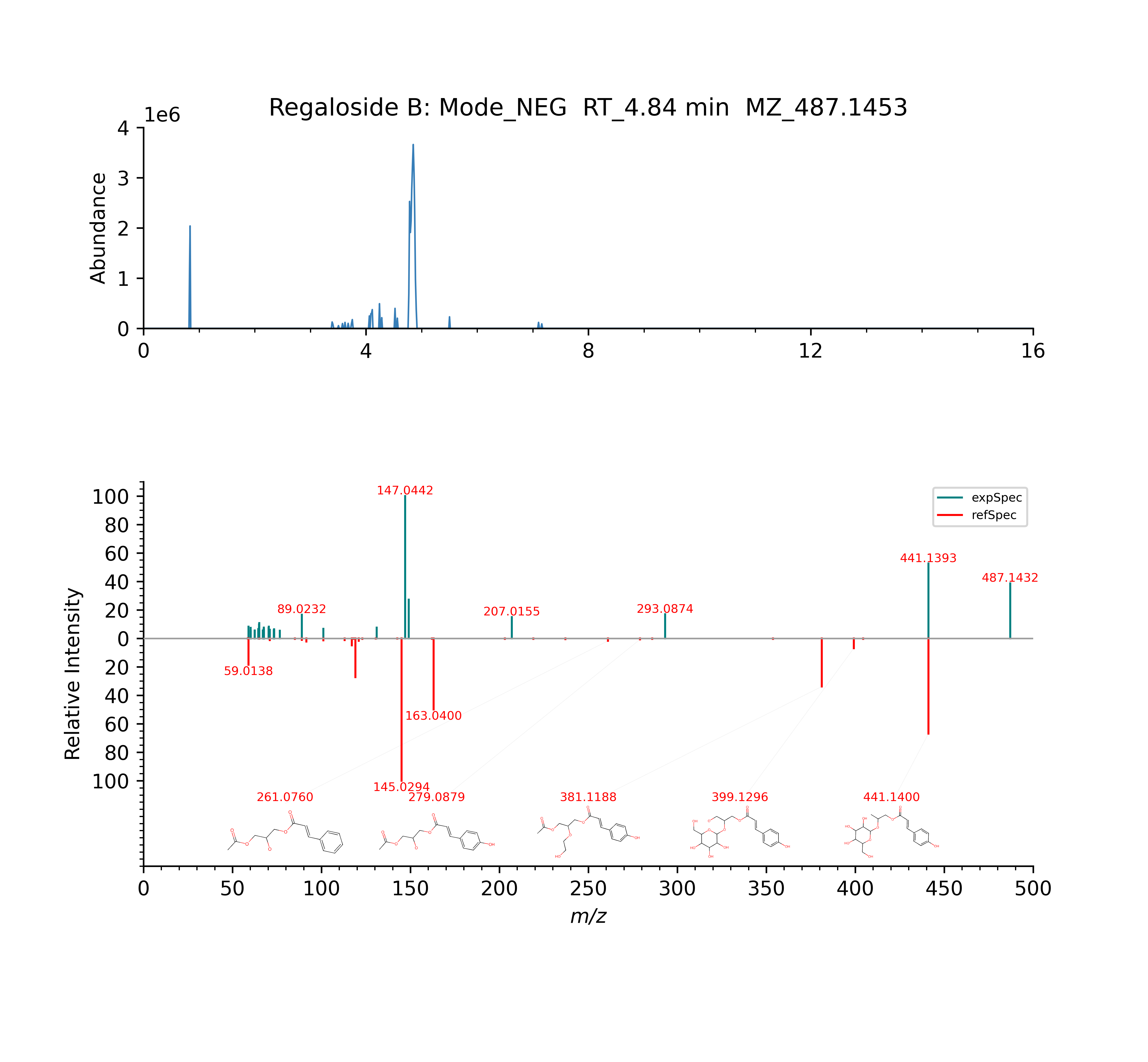

Supplement: Supplementary file 1 [file ijms-27-02203-s001.zip › ijms-4070482 Supplementary/Metabolite List Identified by LC-MS_MS from Rhodiola Species/89.png]

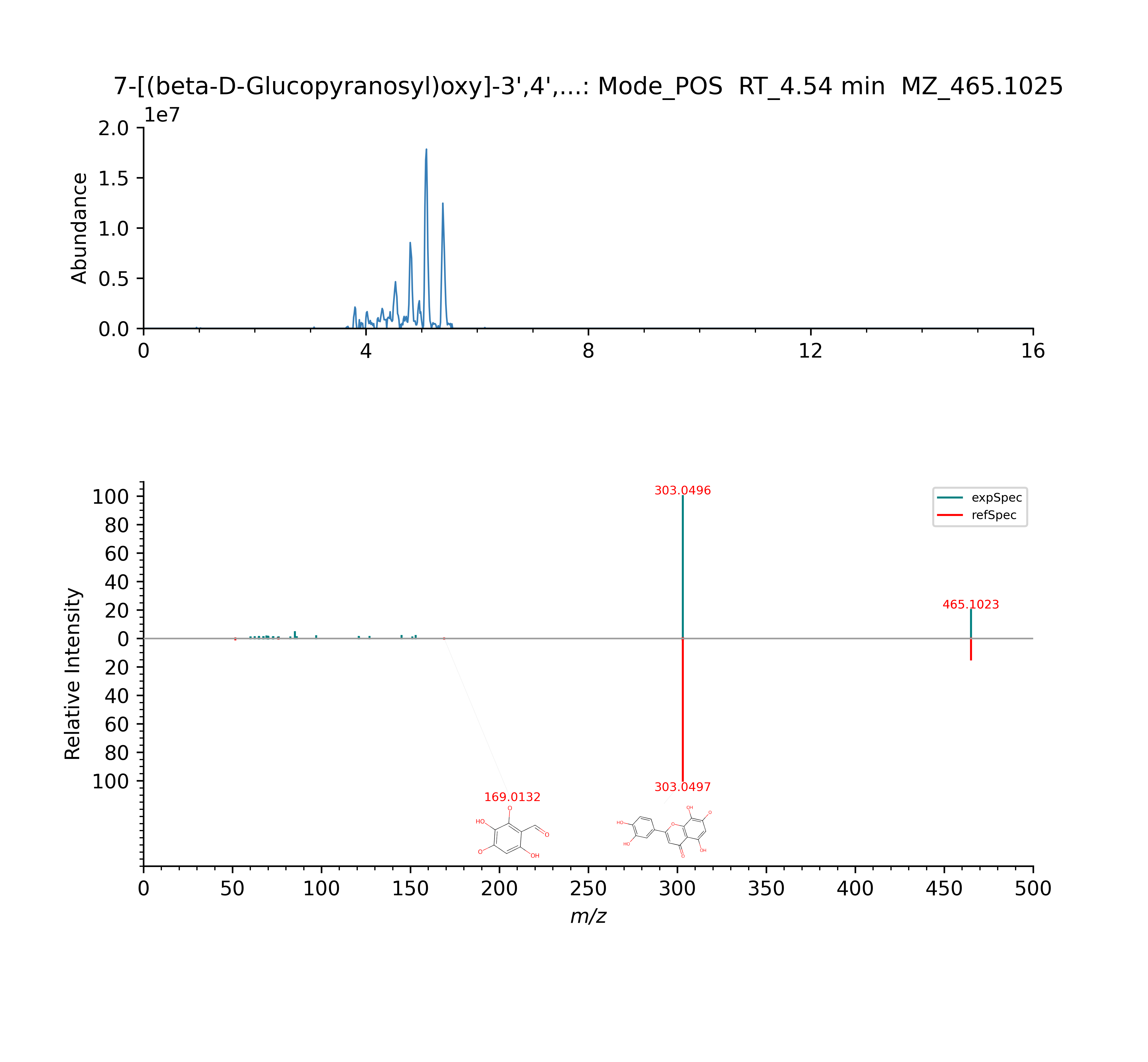

Supplement: Supplementary file 1 [file ijms-27-02203-s001.zip › ijms-4070482 Supplementary/Metabolite List Identified by LC-MS_MS from Rhodiola Species/9.png]

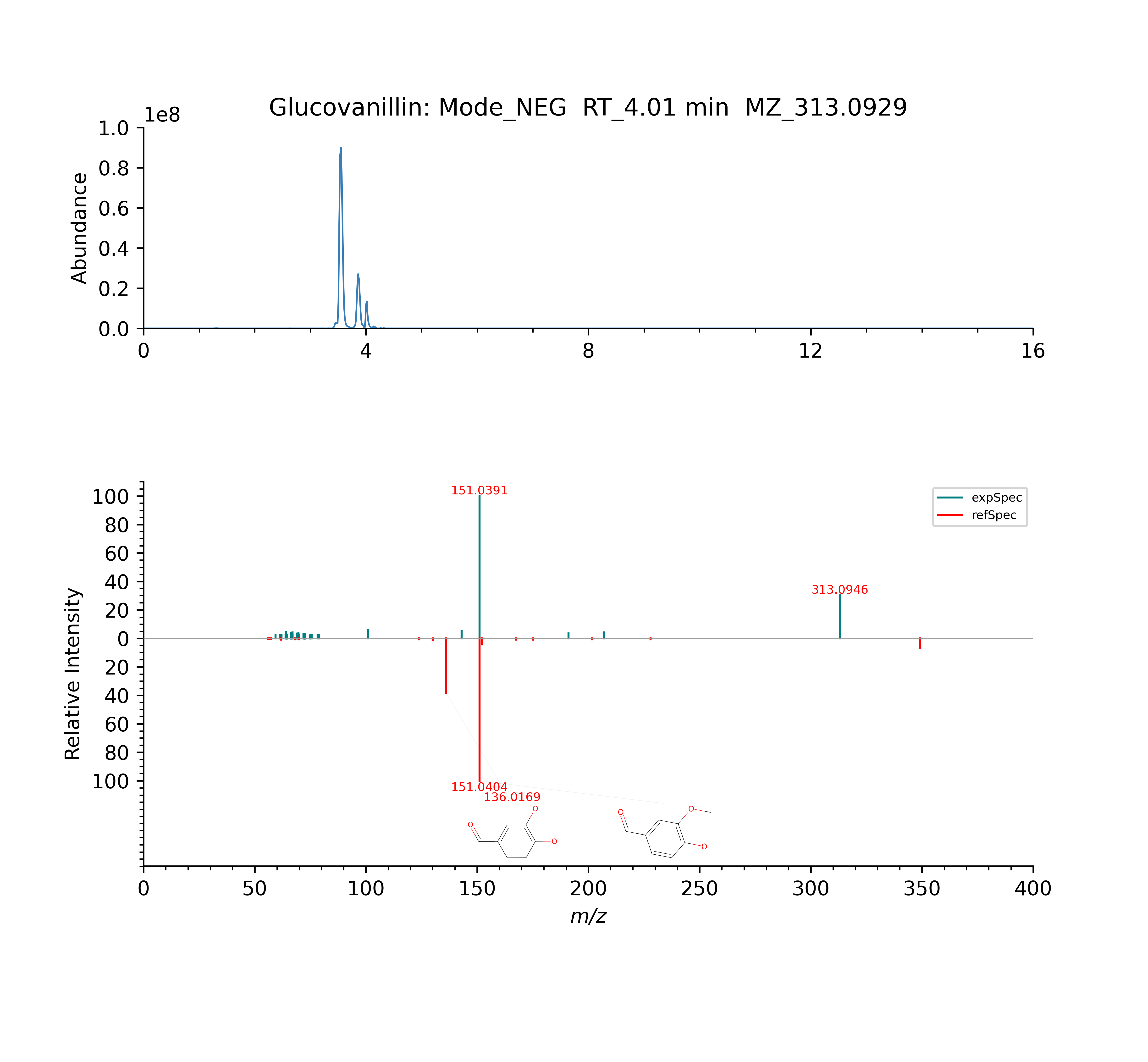

Supplement: Supplementary file 1 [file ijms-27-02203-s001.zip › ijms-4070482 Supplementary/Metabolite List Identified by LC-MS_MS from Rhodiola Species/90.png]

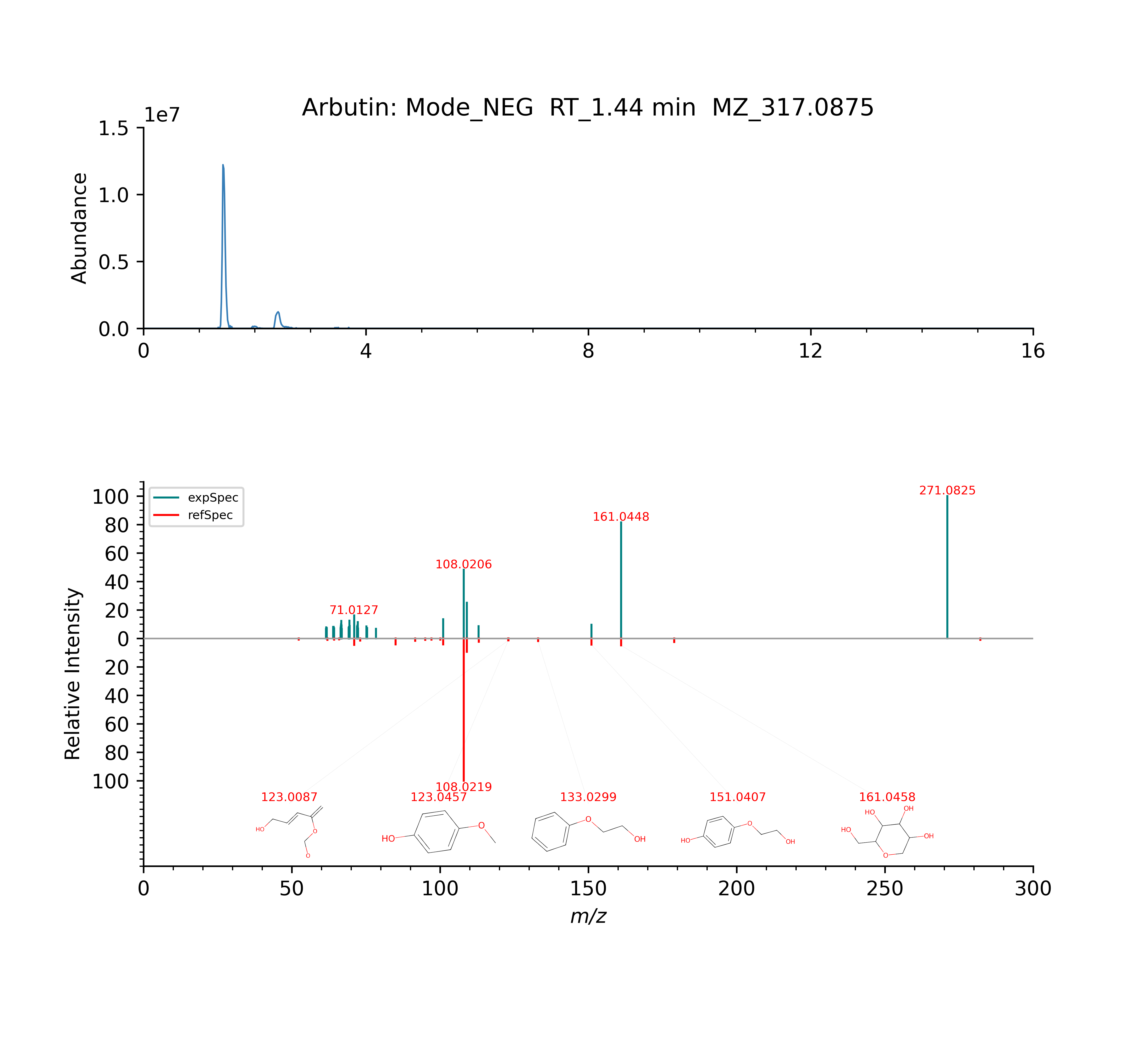

Supplement: Supplementary file 1 [file ijms-27-02203-s001.zip › ijms-4070482 Supplementary/Metabolite List Identified by LC-MS_MS from Rhodiola Species/91.png]

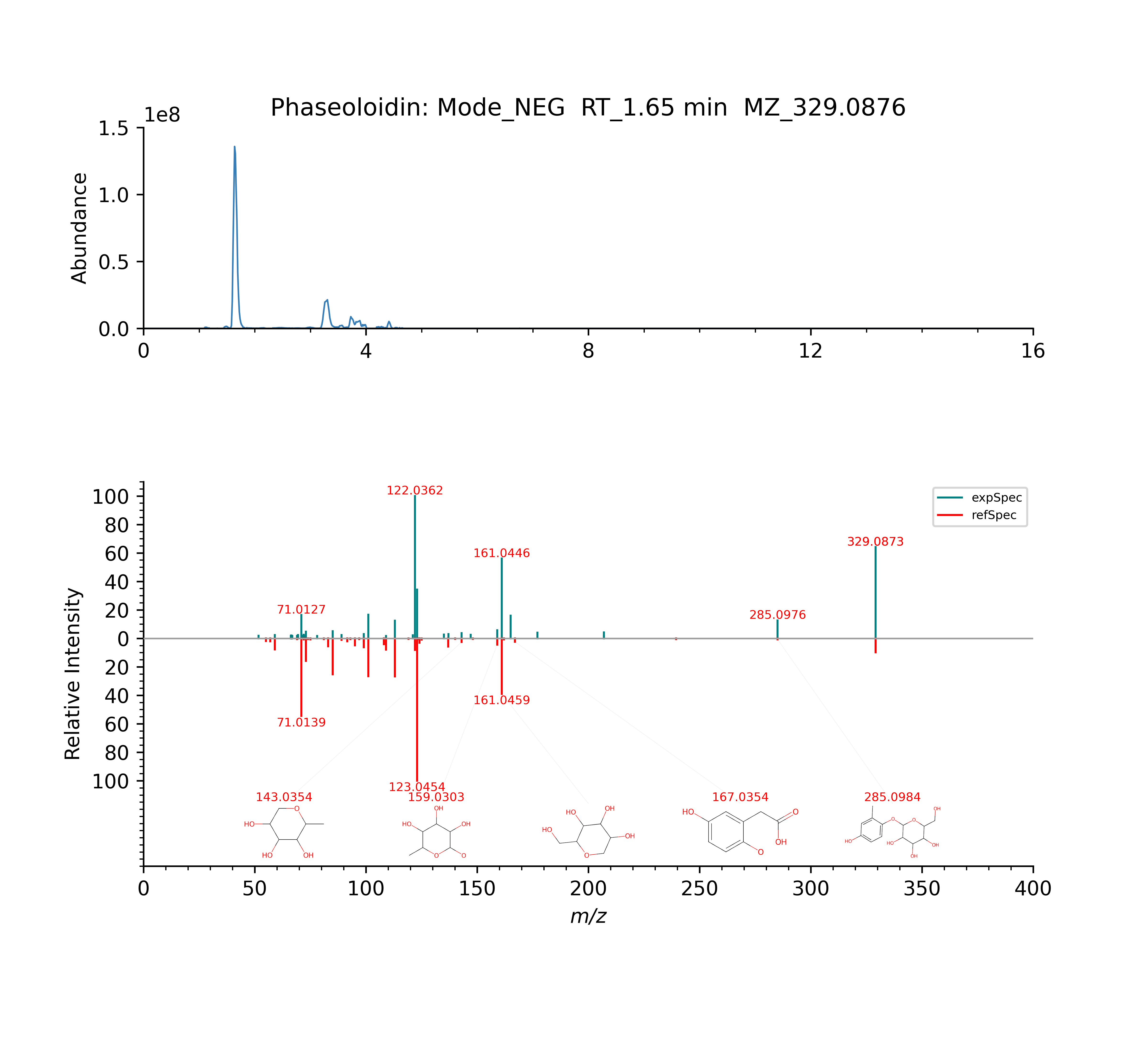

Supplement: Supplementary file 1 [file ijms-27-02203-s001.zip › ijms-4070482 Supplementary/Metabolite List Identified by LC-MS_MS from Rhodiola Species/92.png]

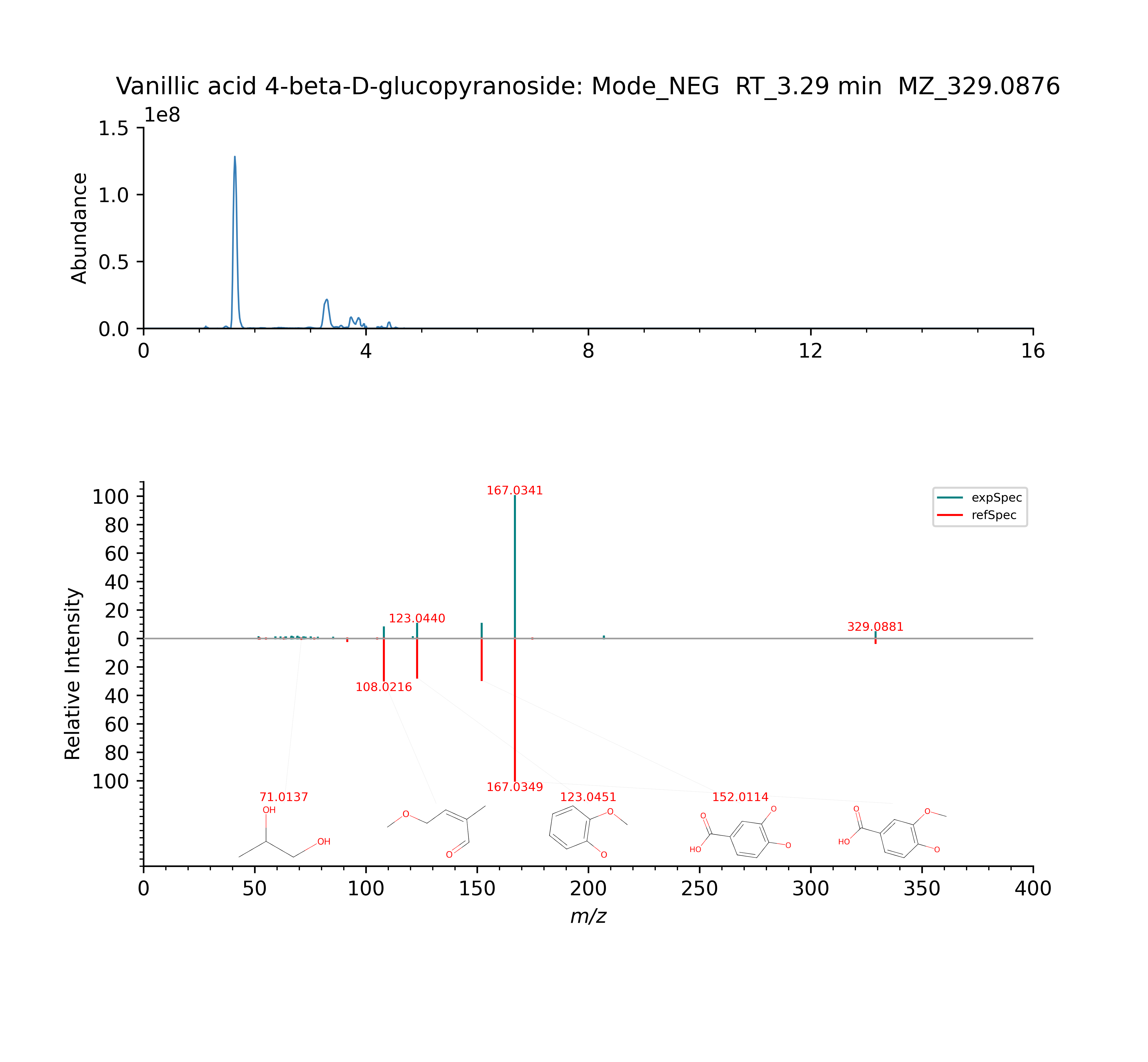

Supplement: Supplementary file 1 [file ijms-27-02203-s001.zip › ijms-4070482 Supplementary/Metabolite List Identified by LC-MS_MS from Rhodiola Species/93.png]

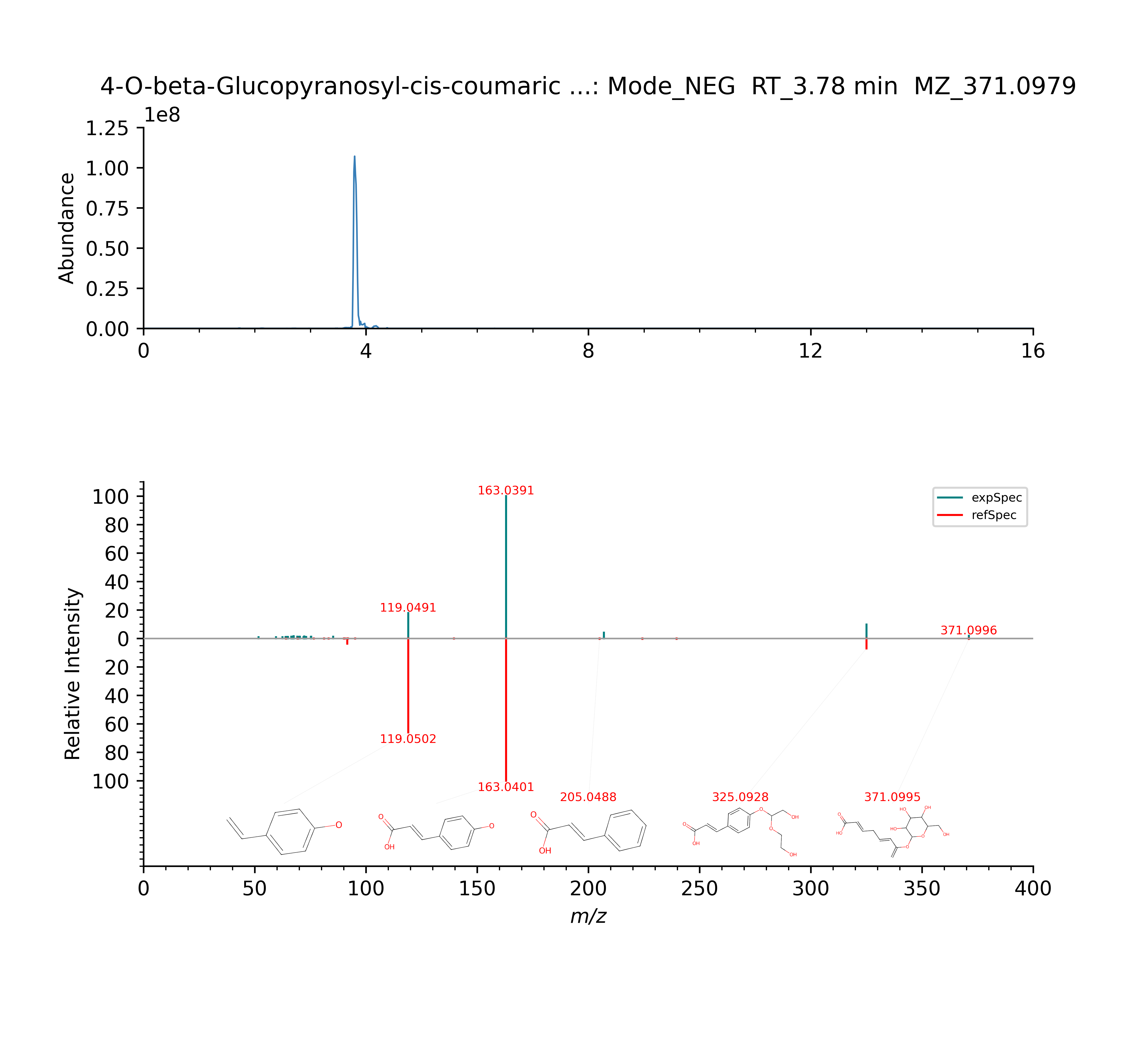

Supplement: Supplementary file 1 [file ijms-27-02203-s001.zip › ijms-4070482 Supplementary/Metabolite List Identified by LC-MS_MS from Rhodiola Species/94.png]

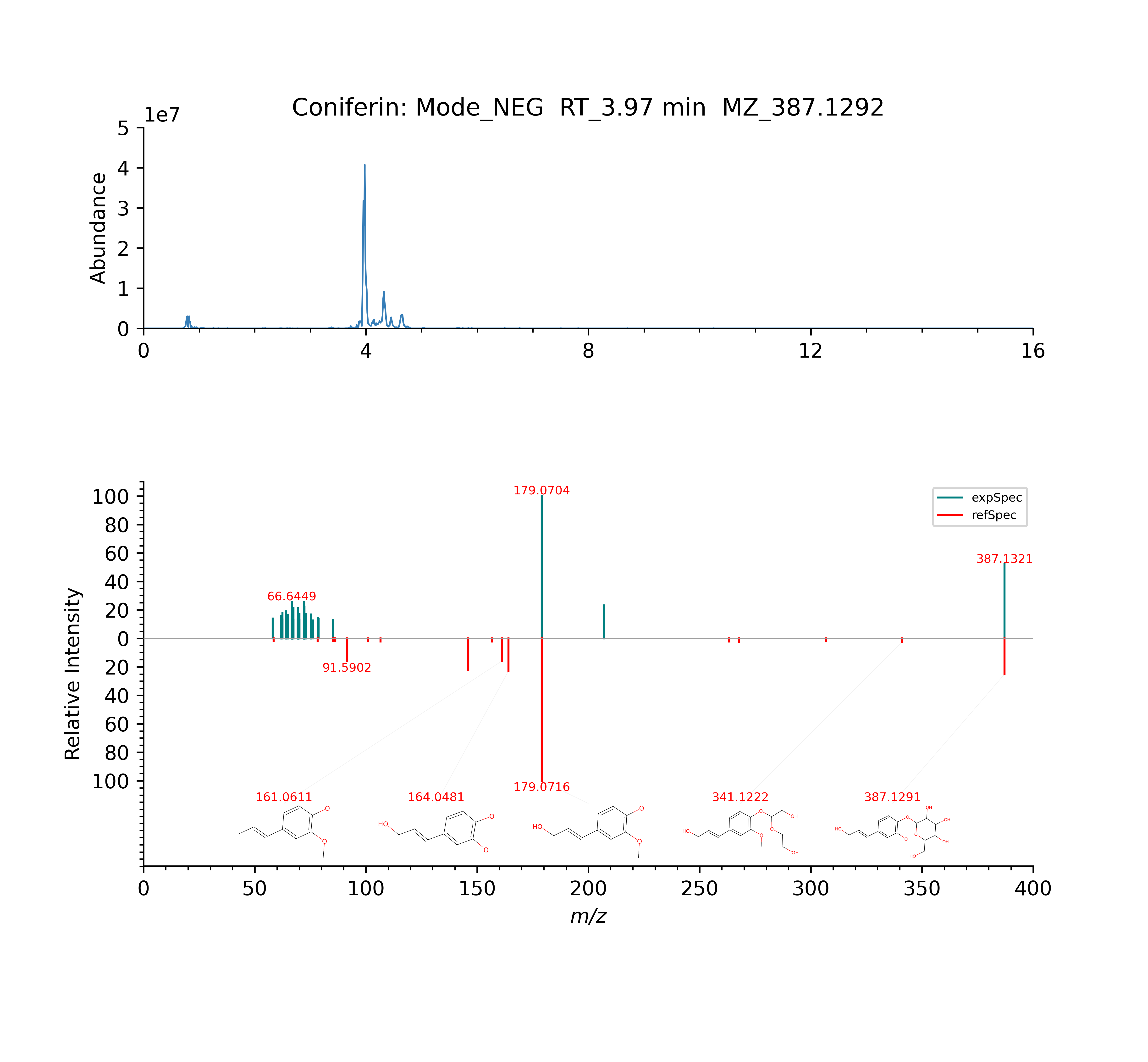

Supplement: Supplementary file 1 [file ijms-27-02203-s001.zip › ijms-4070482 Supplementary/Metabolite List Identified by LC-MS_MS from Rhodiola Species/95.png]

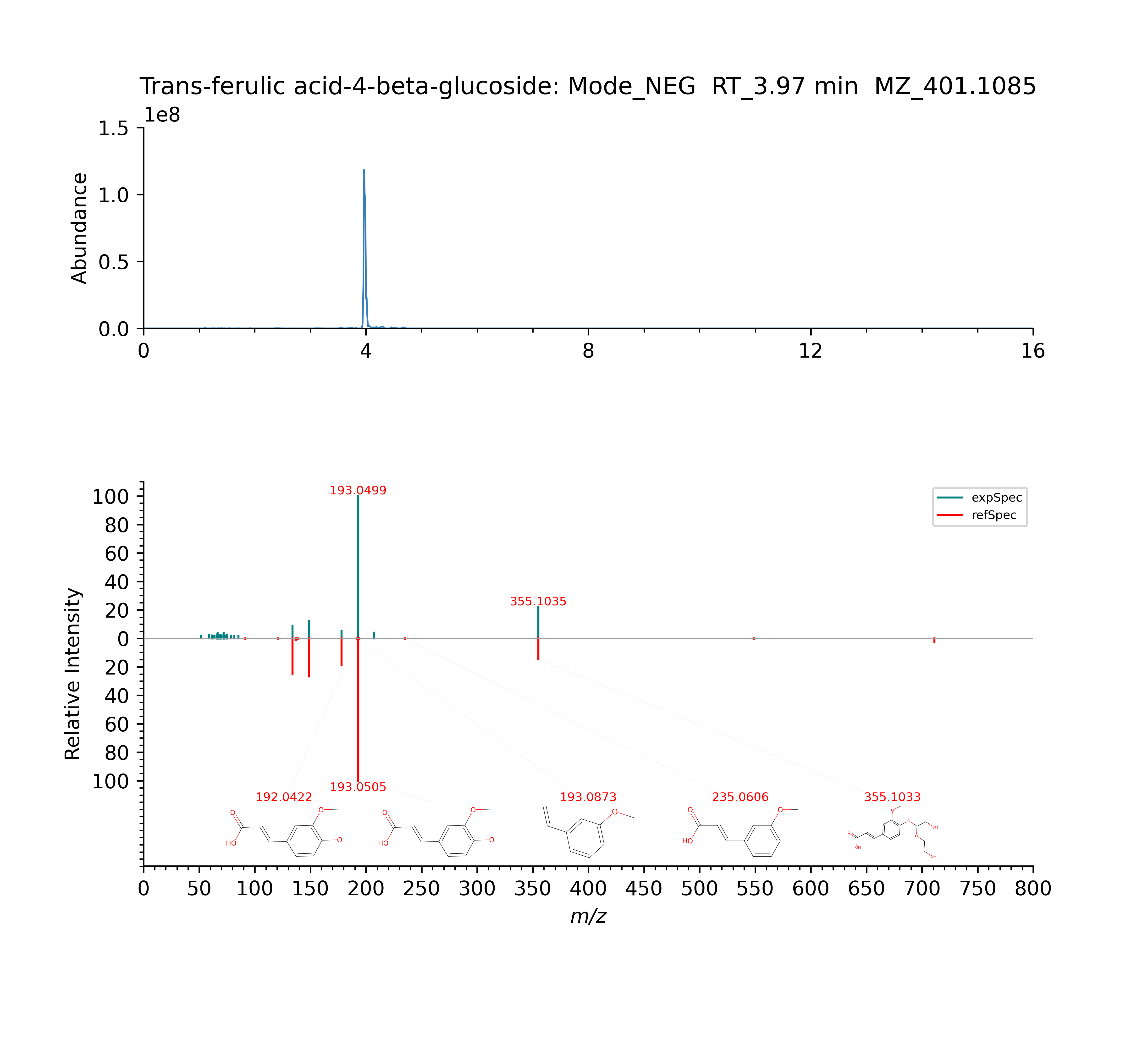

Supplement: Supplementary file 1 [file ijms-27-02203-s001.zip › ijms-4070482 Supplementary/Metabolite List Identified by LC-MS_MS from Rhodiola Species/96.png]

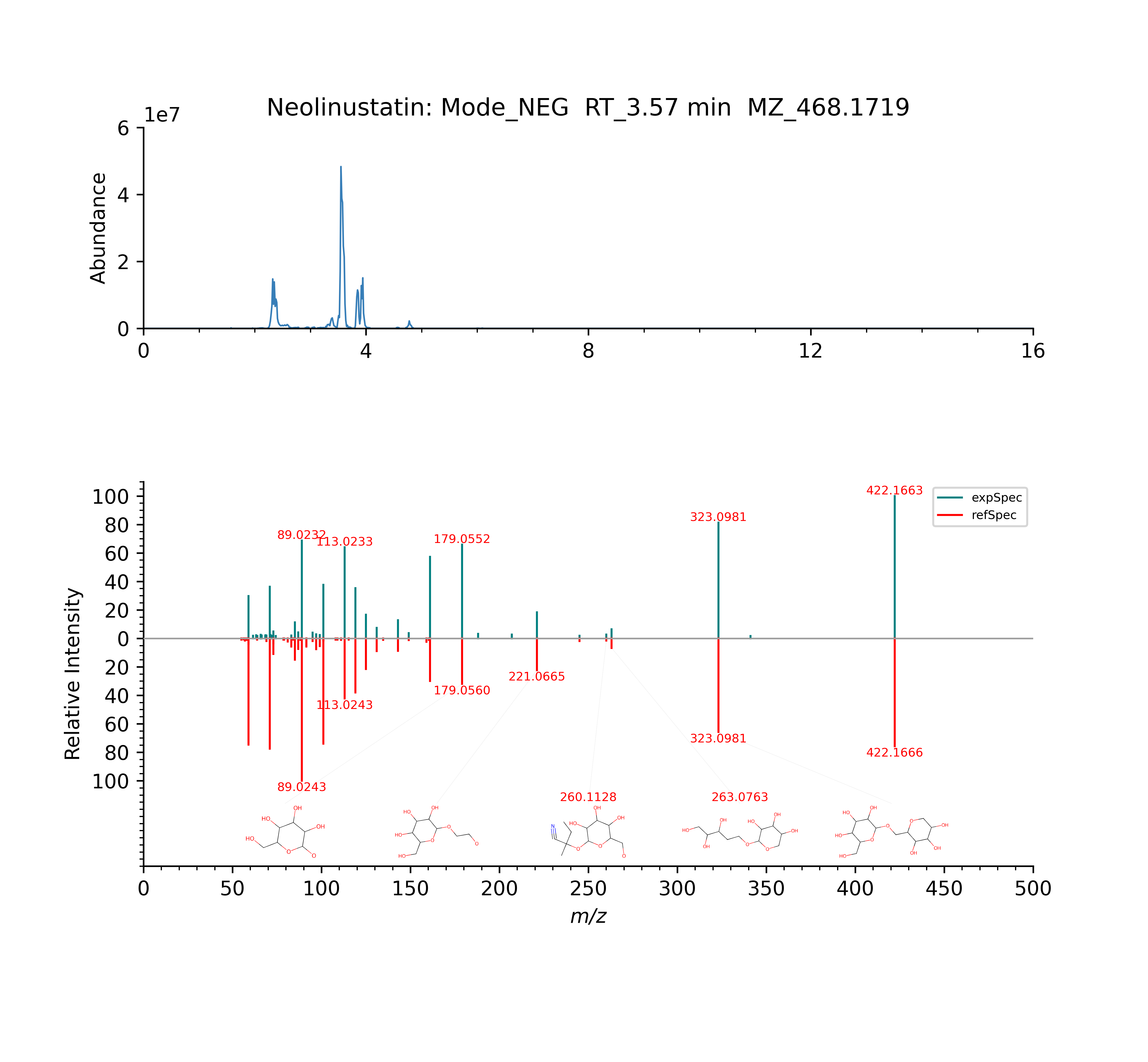

Supplement: Supplementary file 1 [file ijms-27-02203-s001.zip › ijms-4070482 Supplementary/Metabolite List Identified by LC-MS_MS from Rhodiola Species/97.png]

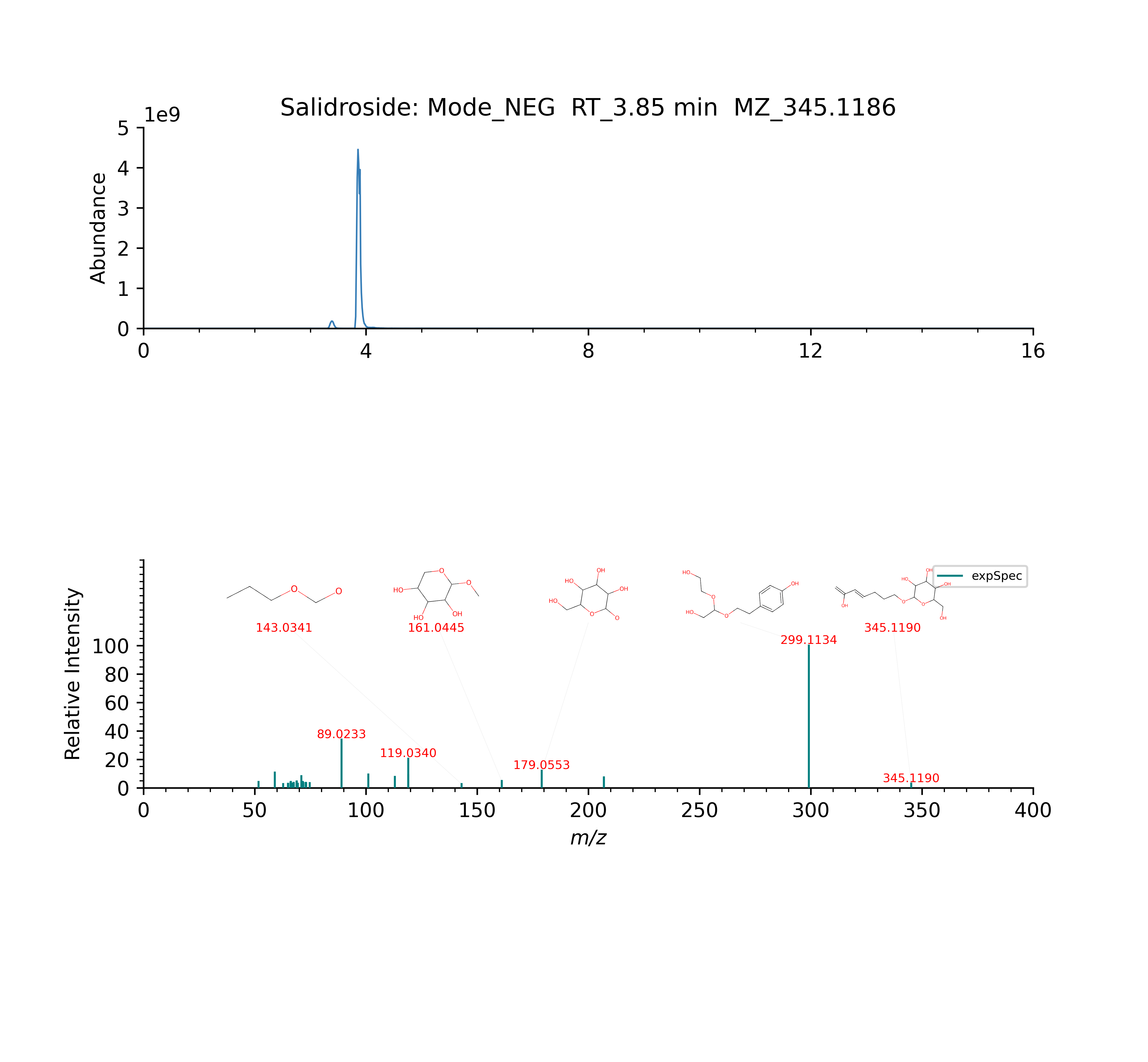

Supplement: Supplementary file 1 [file ijms-27-02203-s001.zip › ijms-4070482 Supplementary/Metabolite List Identified by LC-MS_MS from Rhodiola Species/98.png]

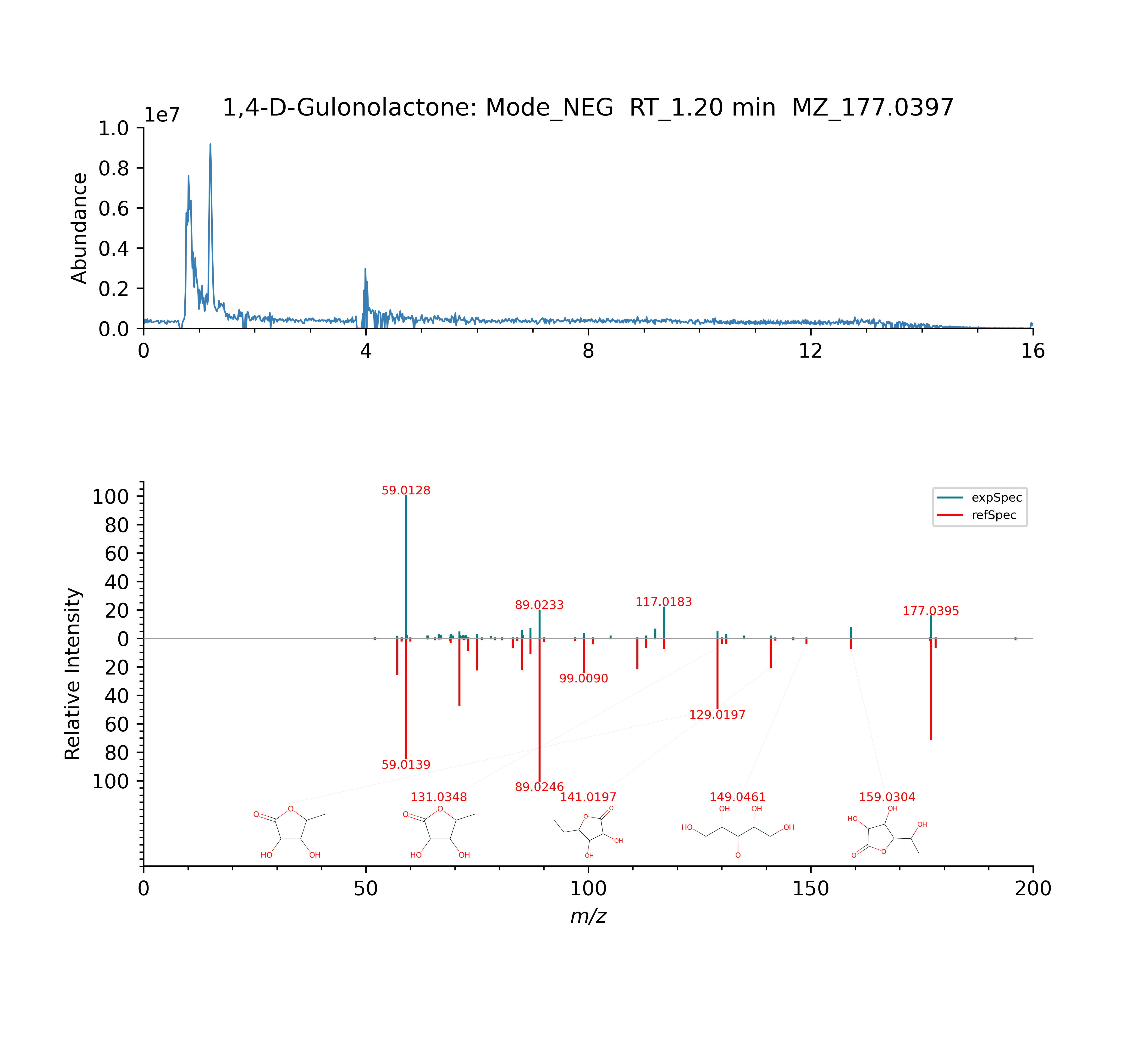

Supplement: Supplementary file 1 [file ijms-27-02203-s001.zip › ijms-4070482 Supplementary/Metabolite List Identified by LC-MS_MS from Rhodiola Species/99.png]
