# Supplementary figures and images for: A “Qualitative–Pharmacological–Correlation–Molecular” Integrated Workflow Reveals HIF-1α–Relevant Anti-Hypoxia Metabolites in Rhodiola Species (part 1 of 2)
Source: Int J Mol Sci. 2026 Feb 26;27(5):2203. doi: 10.3390/ijms27052203 (PMC12984455; doi:10.3390/ijms27052203)

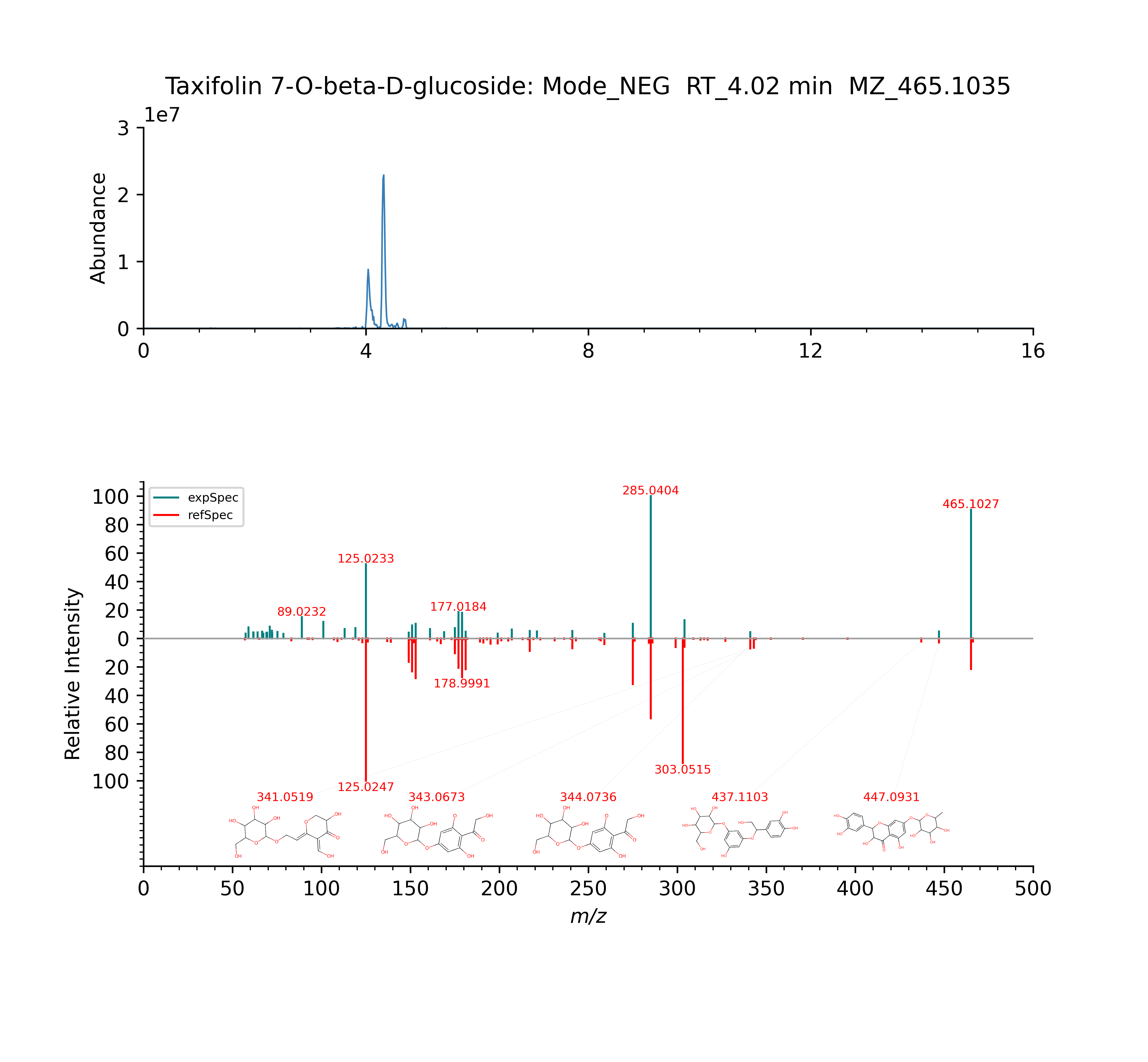

Supplement: Supplementary file 1 [file ijms-27-02203-s001.zip › ijms-4070482 Supplementary/Metabolite List Identified by LC-MS_MS from Rhodiola Species/1.png]

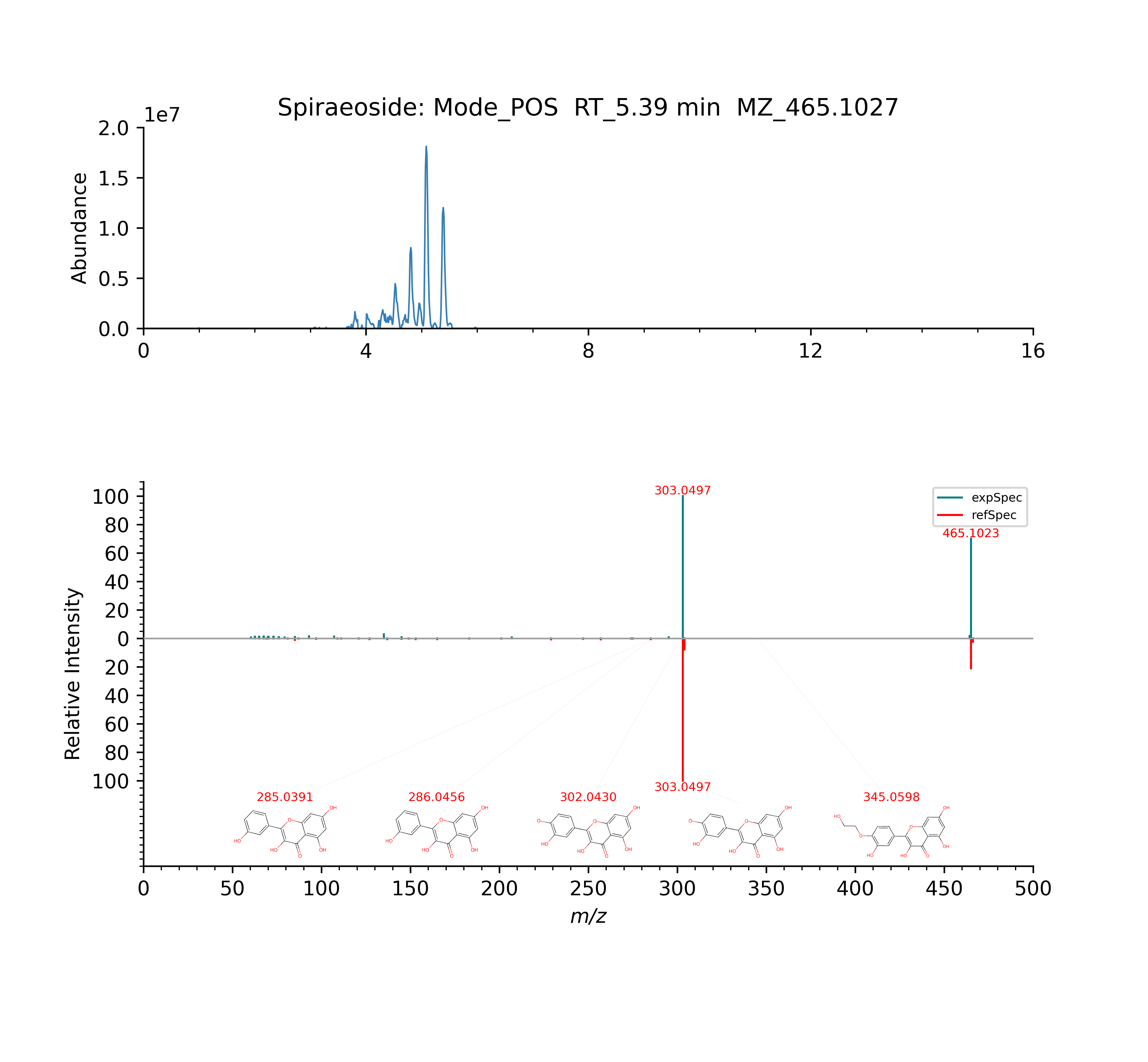

Supplement: Supplementary file 1 [file ijms-27-02203-s001.zip › ijms-4070482 Supplementary/Metabolite List Identified by LC-MS_MS from Rhodiola Species/10.png]

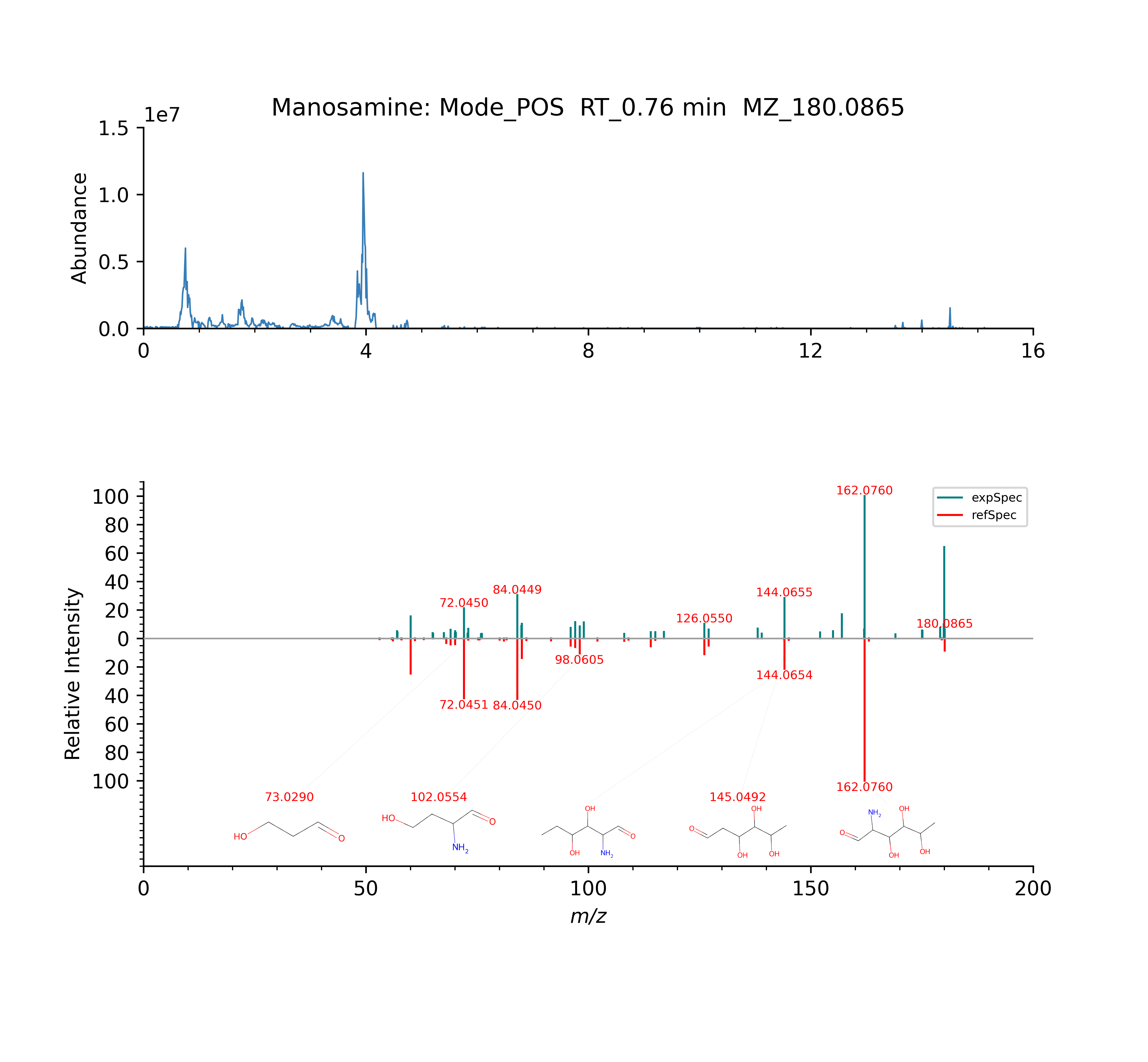

Supplement: Supplementary file 1 [file ijms-27-02203-s001.zip › ijms-4070482 Supplementary/Metabolite List Identified by LC-MS_MS from Rhodiola Species/100.png]

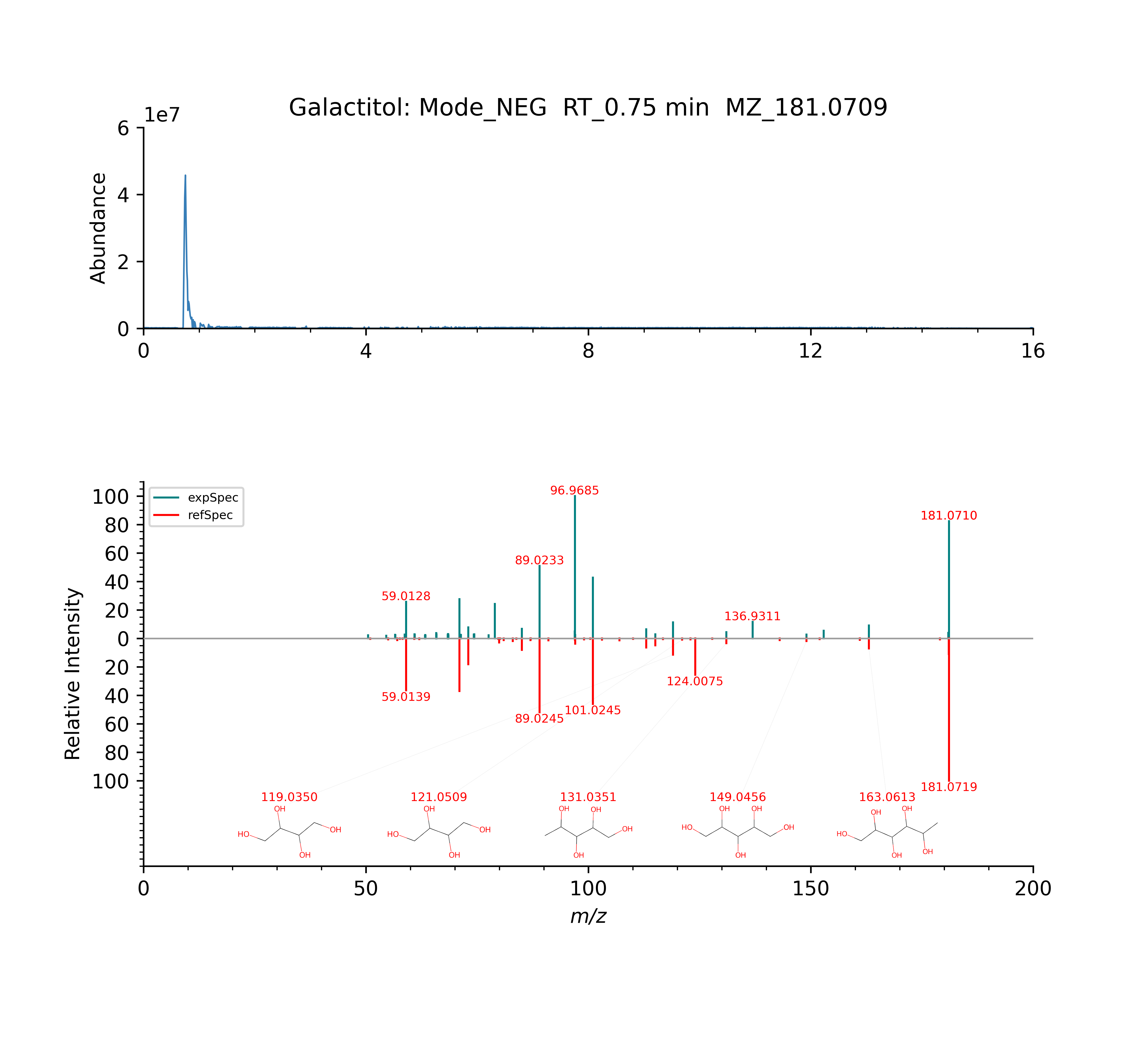

Supplement: Supplementary file 1 [file ijms-27-02203-s001.zip › ijms-4070482 Supplementary/Metabolite List Identified by LC-MS_MS from Rhodiola Species/101.png]

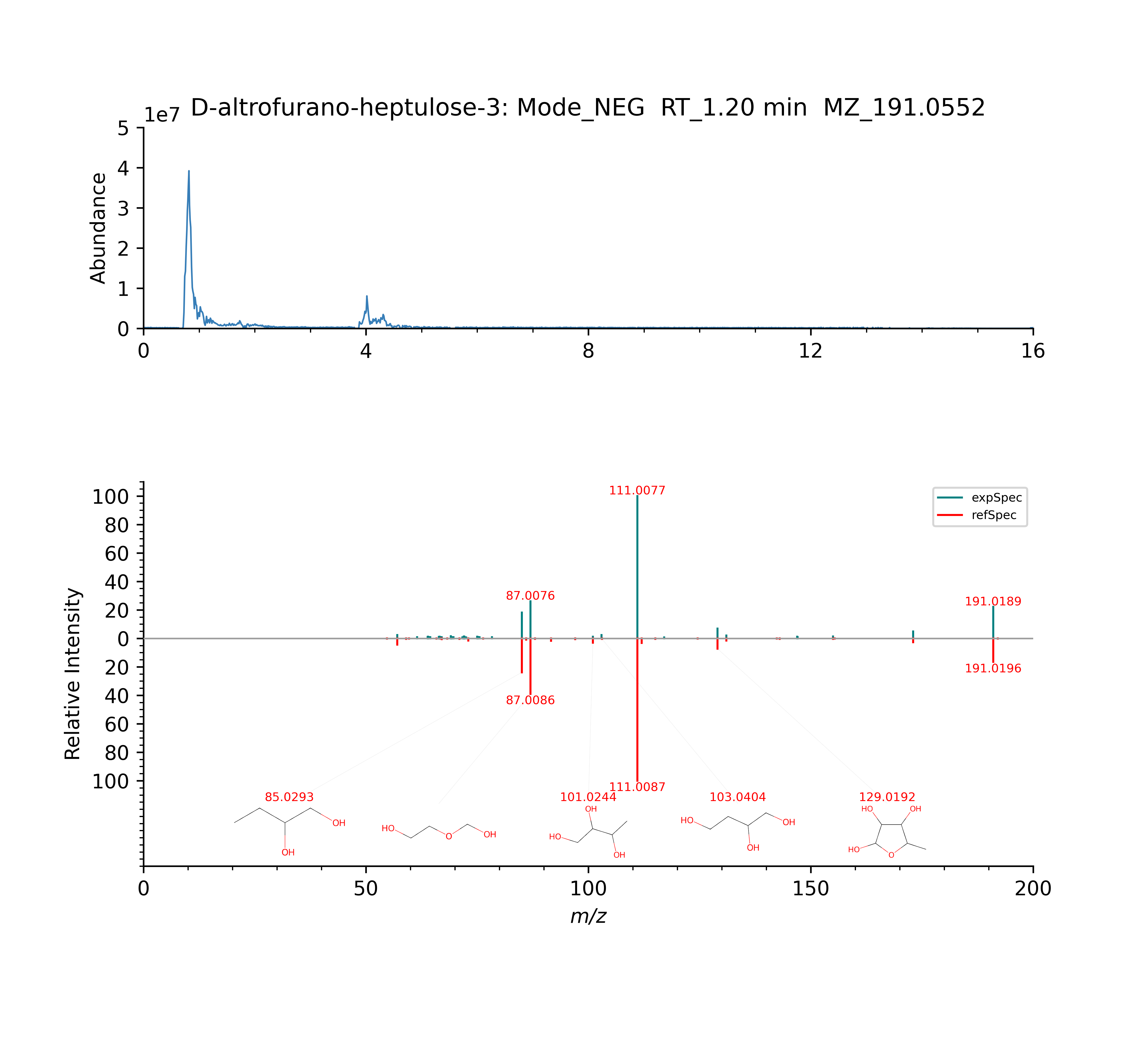

Supplement: Supplementary file 1 [file ijms-27-02203-s001.zip › ijms-4070482 Supplementary/Metabolite List Identified by LC-MS_MS from Rhodiola Species/102.png]

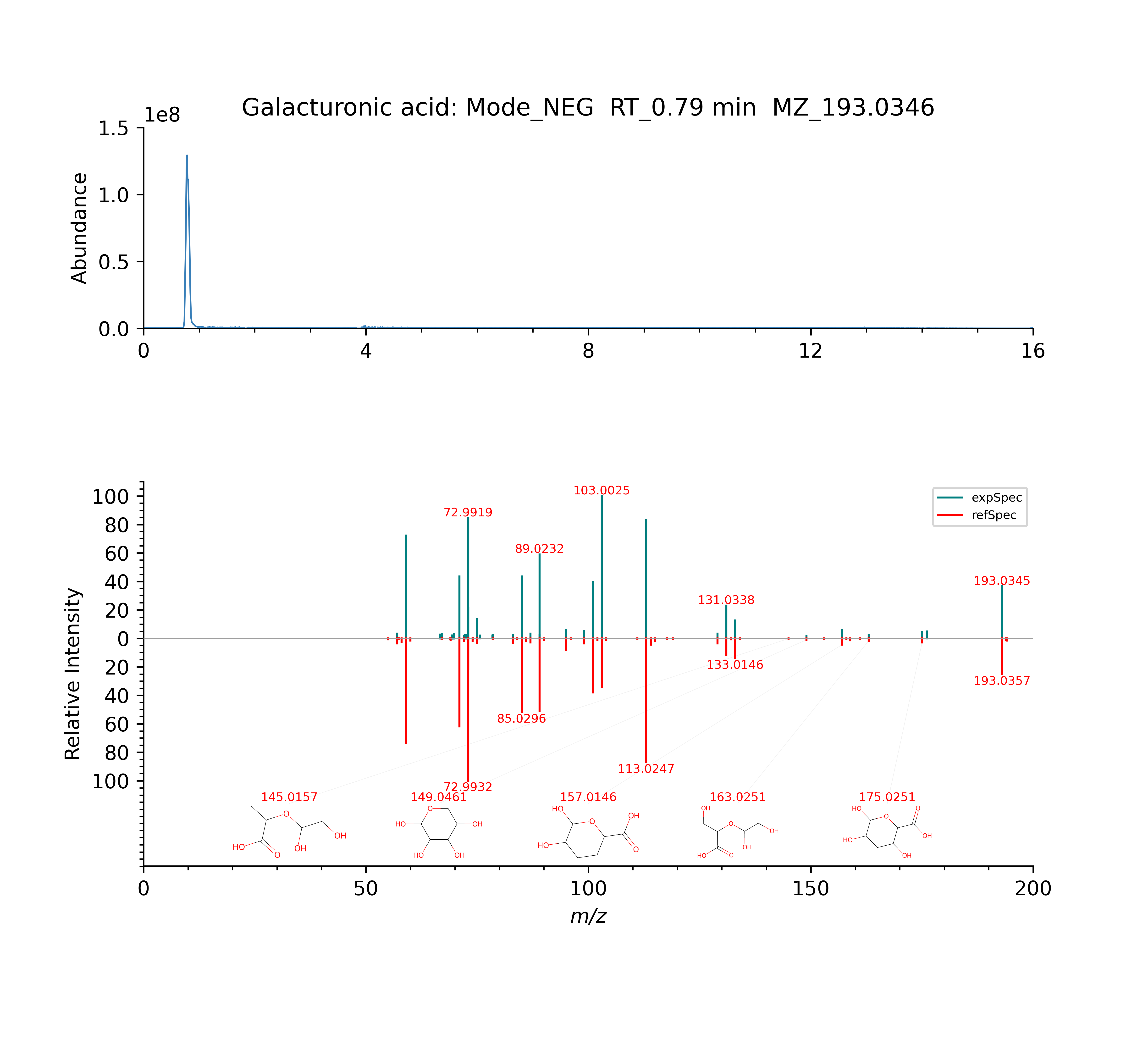

Supplement: Supplementary file 1 [file ijms-27-02203-s001.zip › ijms-4070482 Supplementary/Metabolite List Identified by LC-MS_MS from Rhodiola Species/103.png]

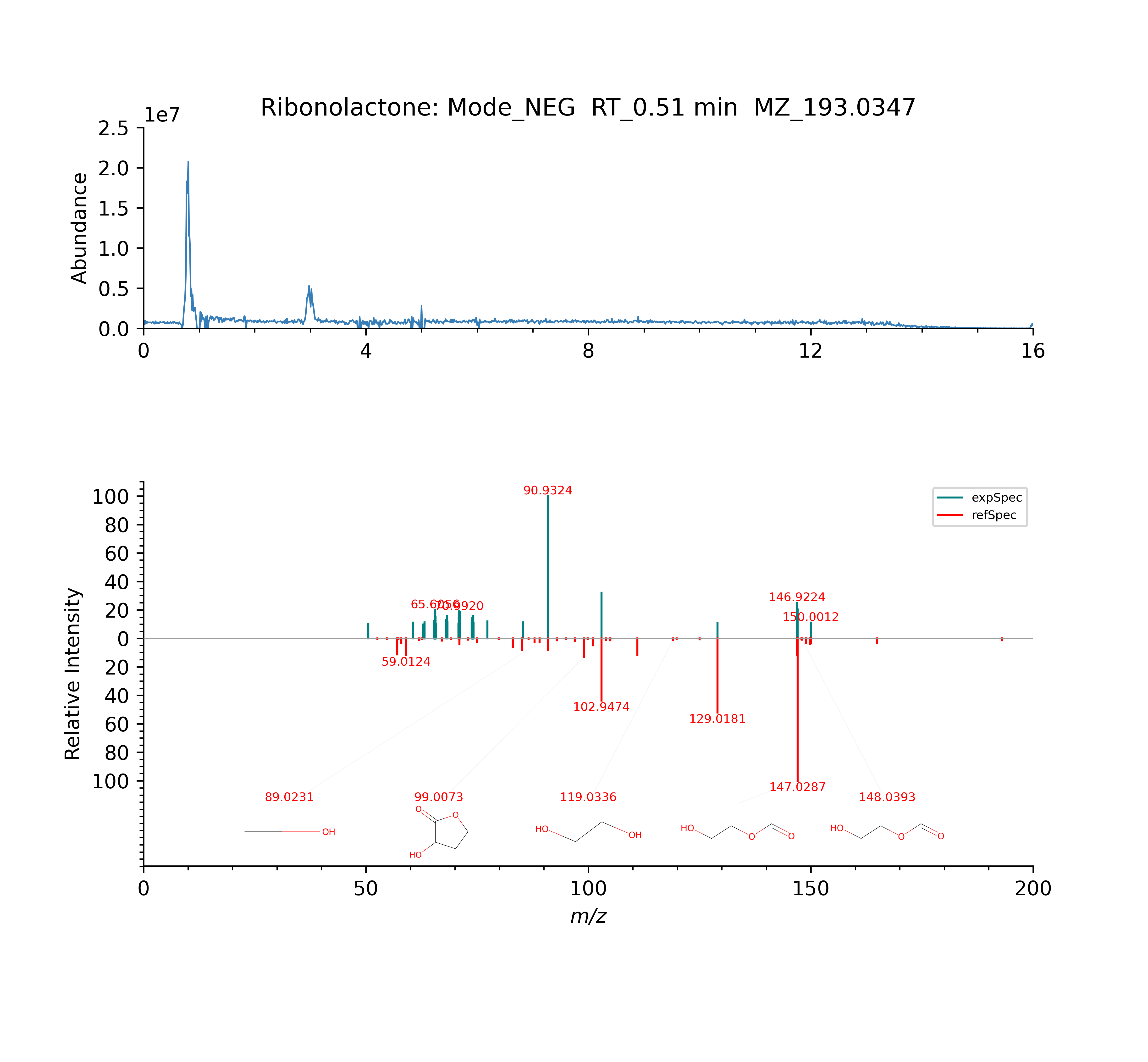

Supplement: Supplementary file 1 [file ijms-27-02203-s001.zip › ijms-4070482 Supplementary/Metabolite List Identified by LC-MS_MS from Rhodiola Species/104.png]

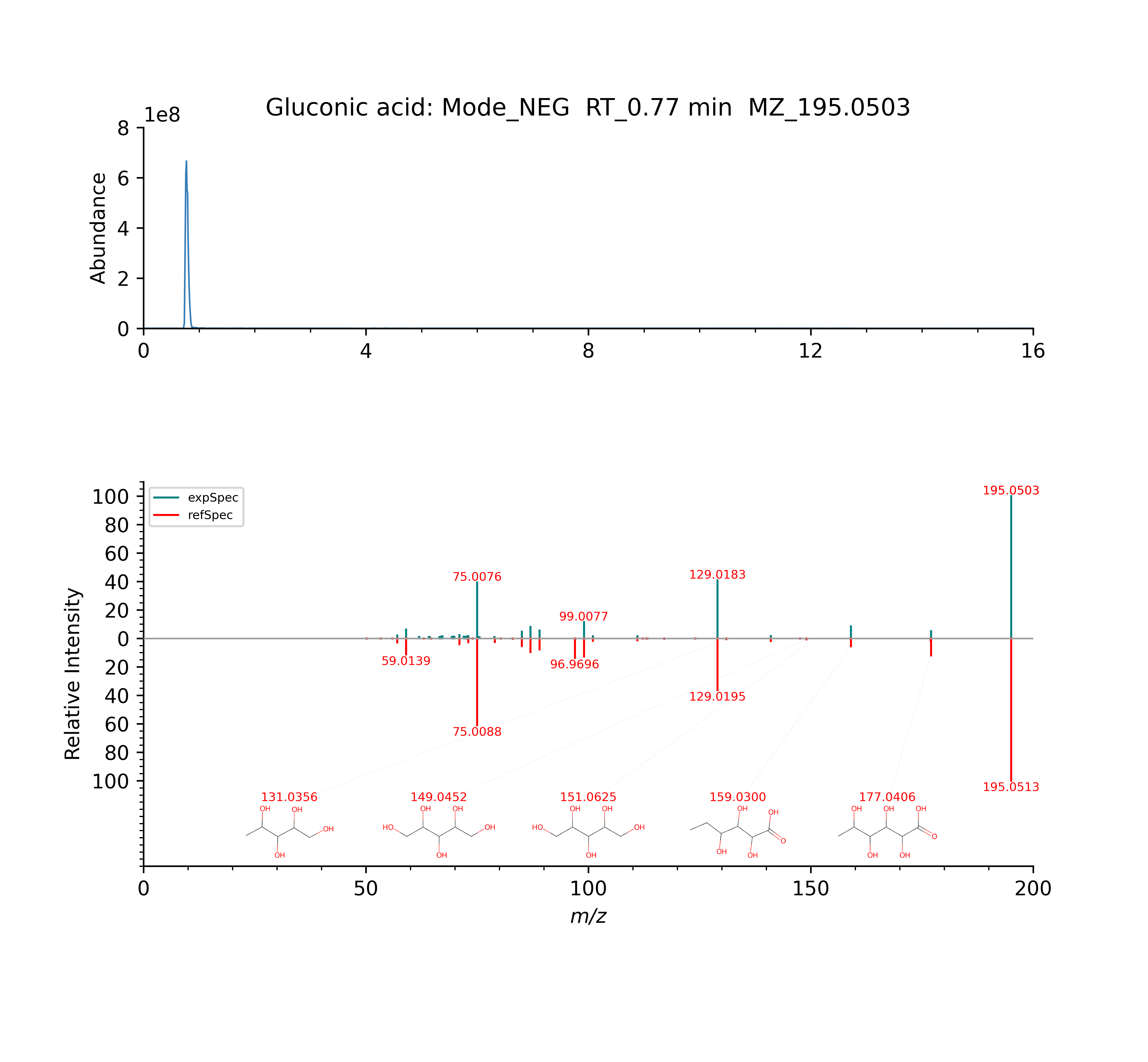

Supplement: Supplementary file 1 [file ijms-27-02203-s001.zip › ijms-4070482 Supplementary/Metabolite List Identified by LC-MS_MS from Rhodiola Species/105.png]

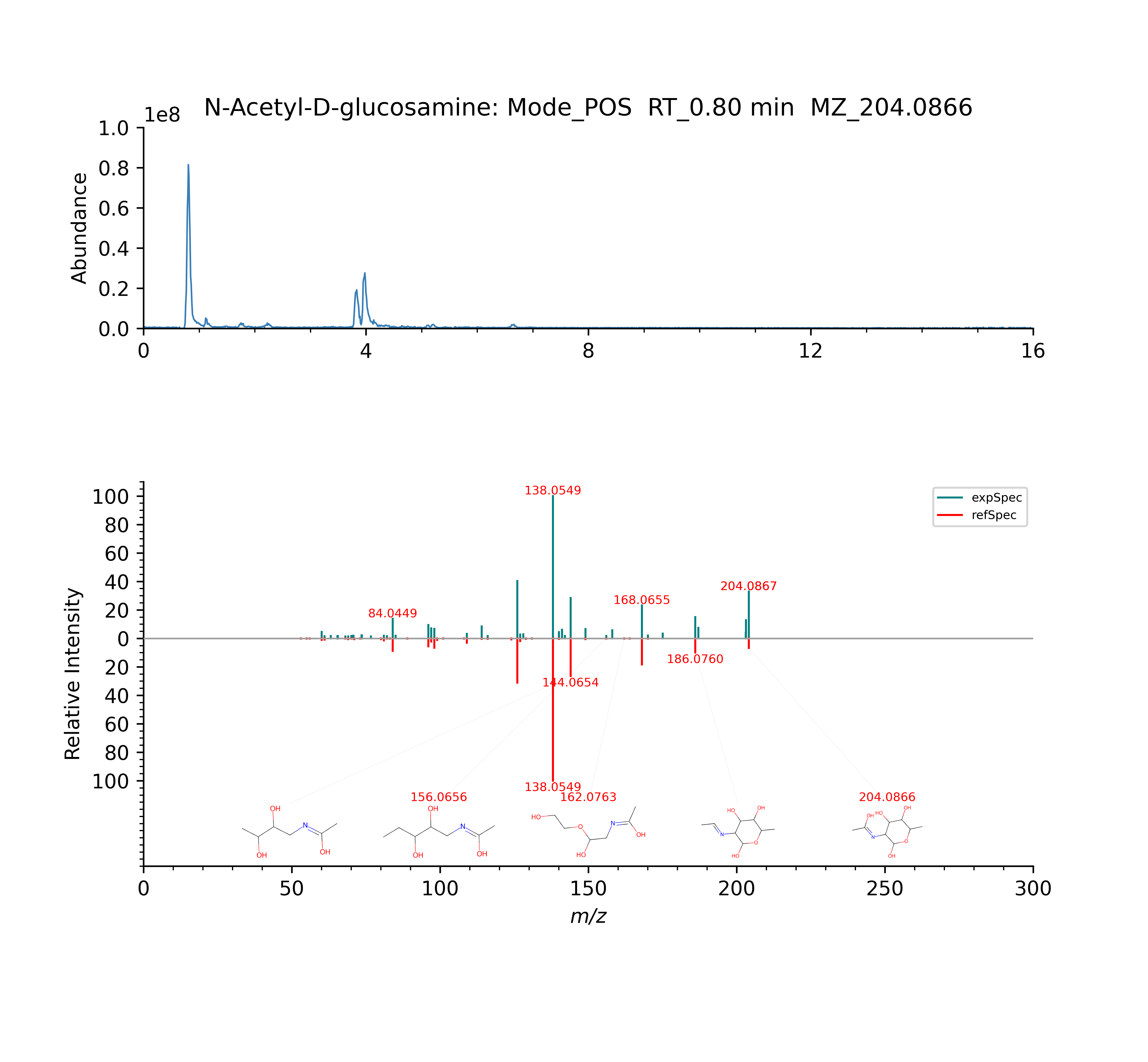

Supplement: Supplementary file 1 [file ijms-27-02203-s001.zip › ijms-4070482 Supplementary/Metabolite List Identified by LC-MS_MS from Rhodiola Species/106.png]

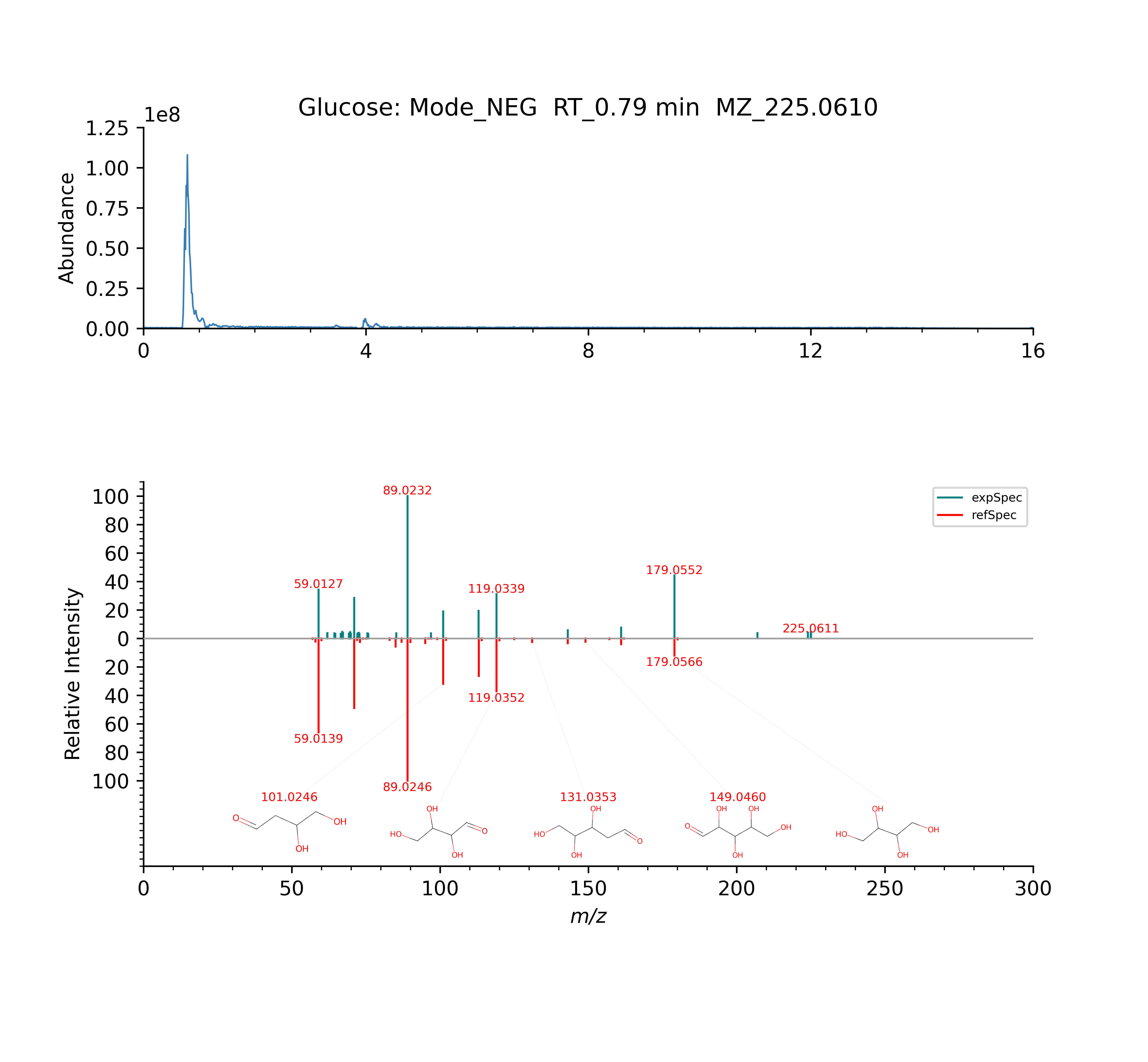

Supplement: Supplementary file 1 [file ijms-27-02203-s001.zip › ijms-4070482 Supplementary/Metabolite List Identified by LC-MS_MS from Rhodiola Species/107.png]

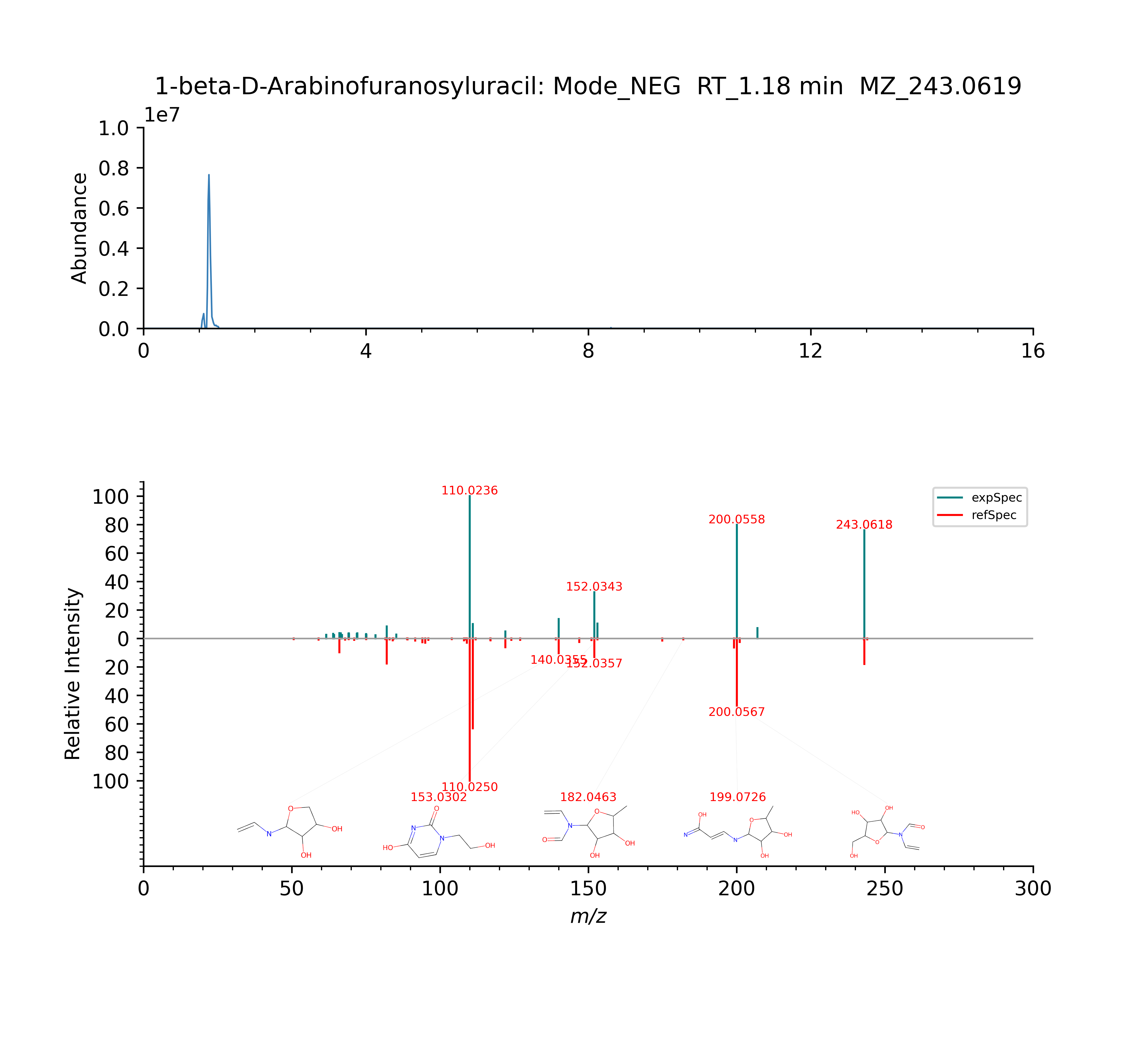

Supplement: Supplementary file 1 [file ijms-27-02203-s001.zip › ijms-4070482 Supplementary/Metabolite List Identified by LC-MS_MS from Rhodiola Species/108.png]

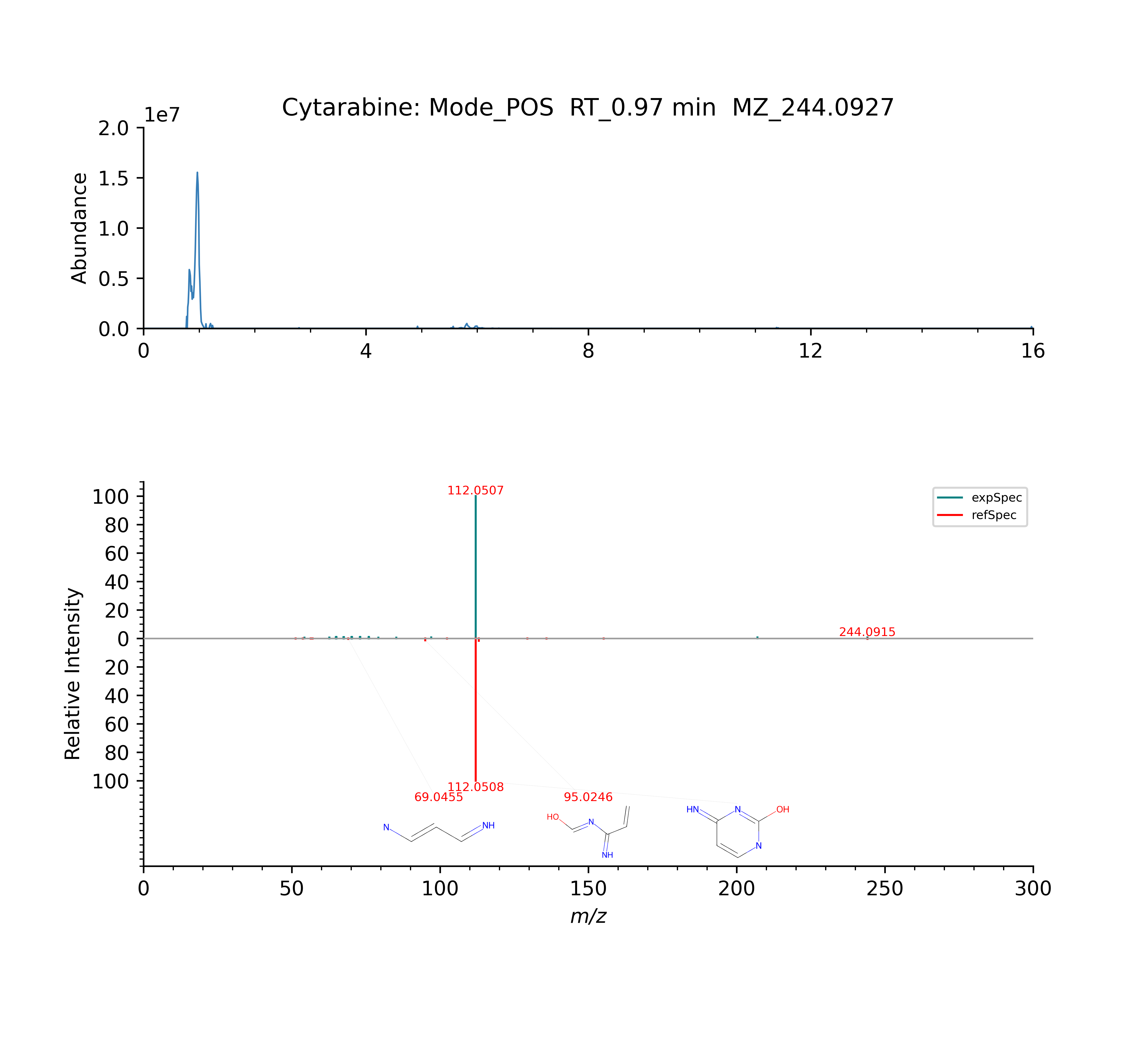

Supplement: Supplementary file 1 [file ijms-27-02203-s001.zip › ijms-4070482 Supplementary/Metabolite List Identified by LC-MS_MS from Rhodiola Species/109.png]

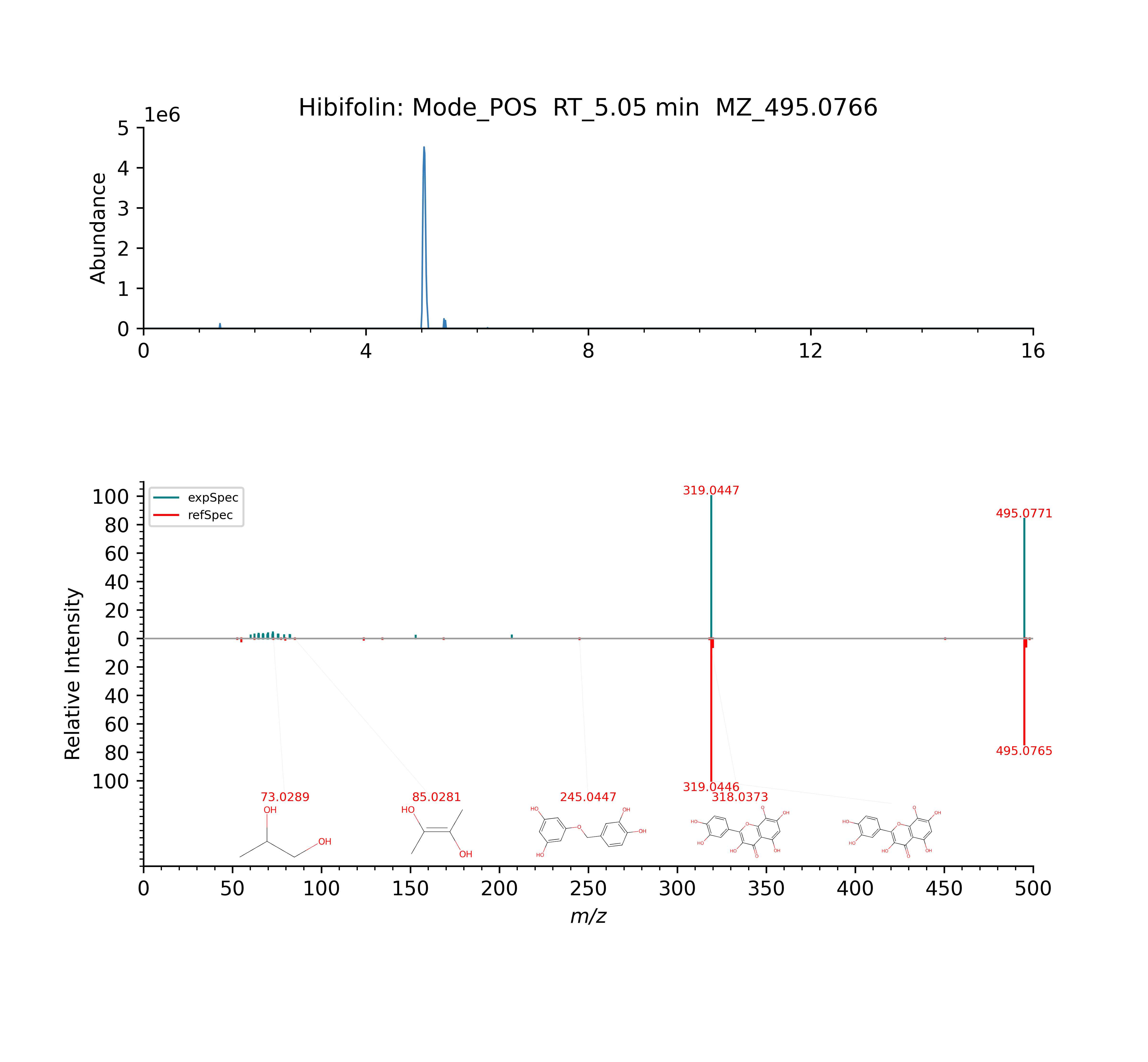

Supplement: Supplementary file 1 [file ijms-27-02203-s001.zip › ijms-4070482 Supplementary/Metabolite List Identified by LC-MS_MS from Rhodiola Species/11.png]

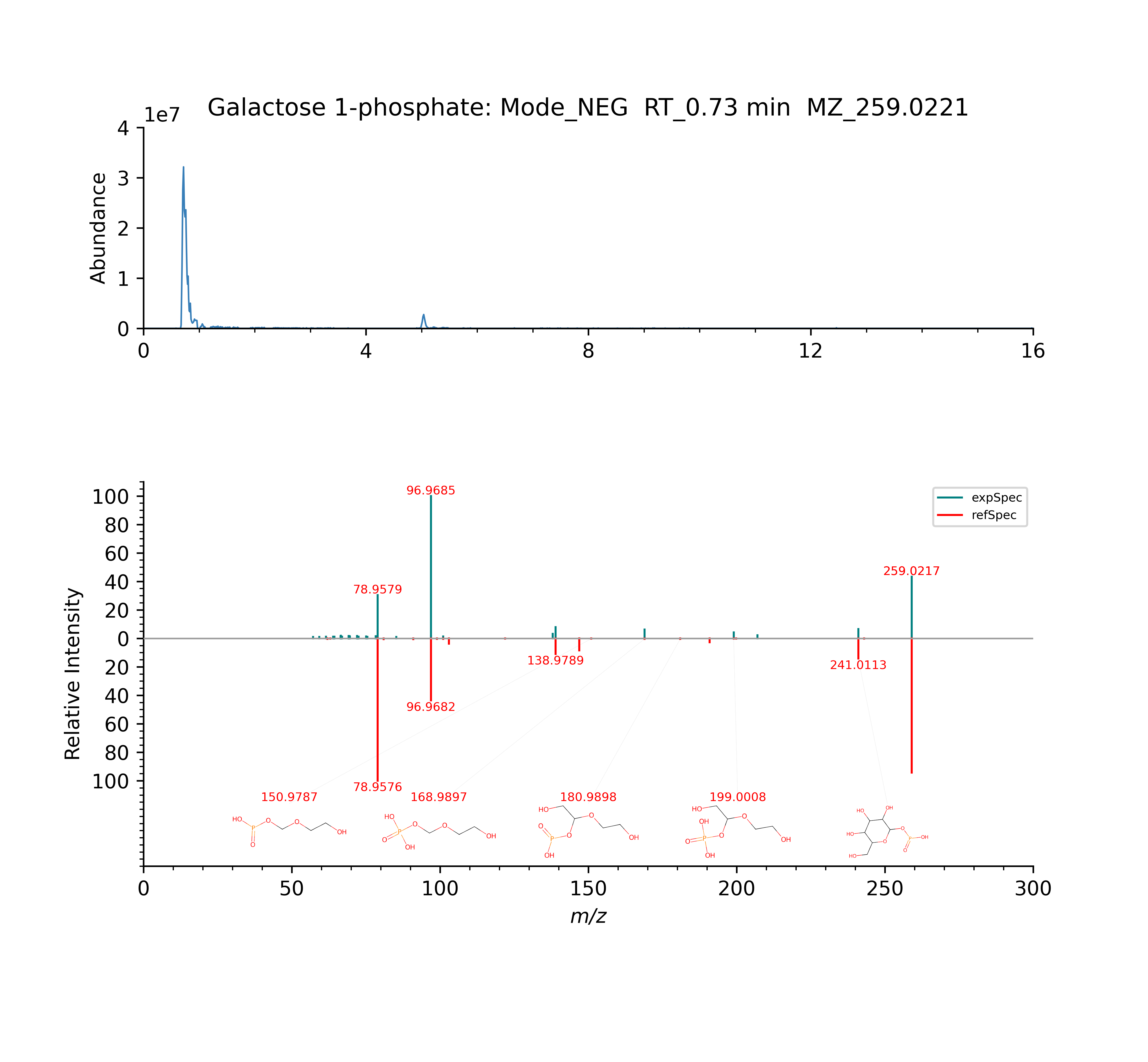

Supplement: Supplementary file 1 [file ijms-27-02203-s001.zip › ijms-4070482 Supplementary/Metabolite List Identified by LC-MS_MS from Rhodiola Species/110.png]

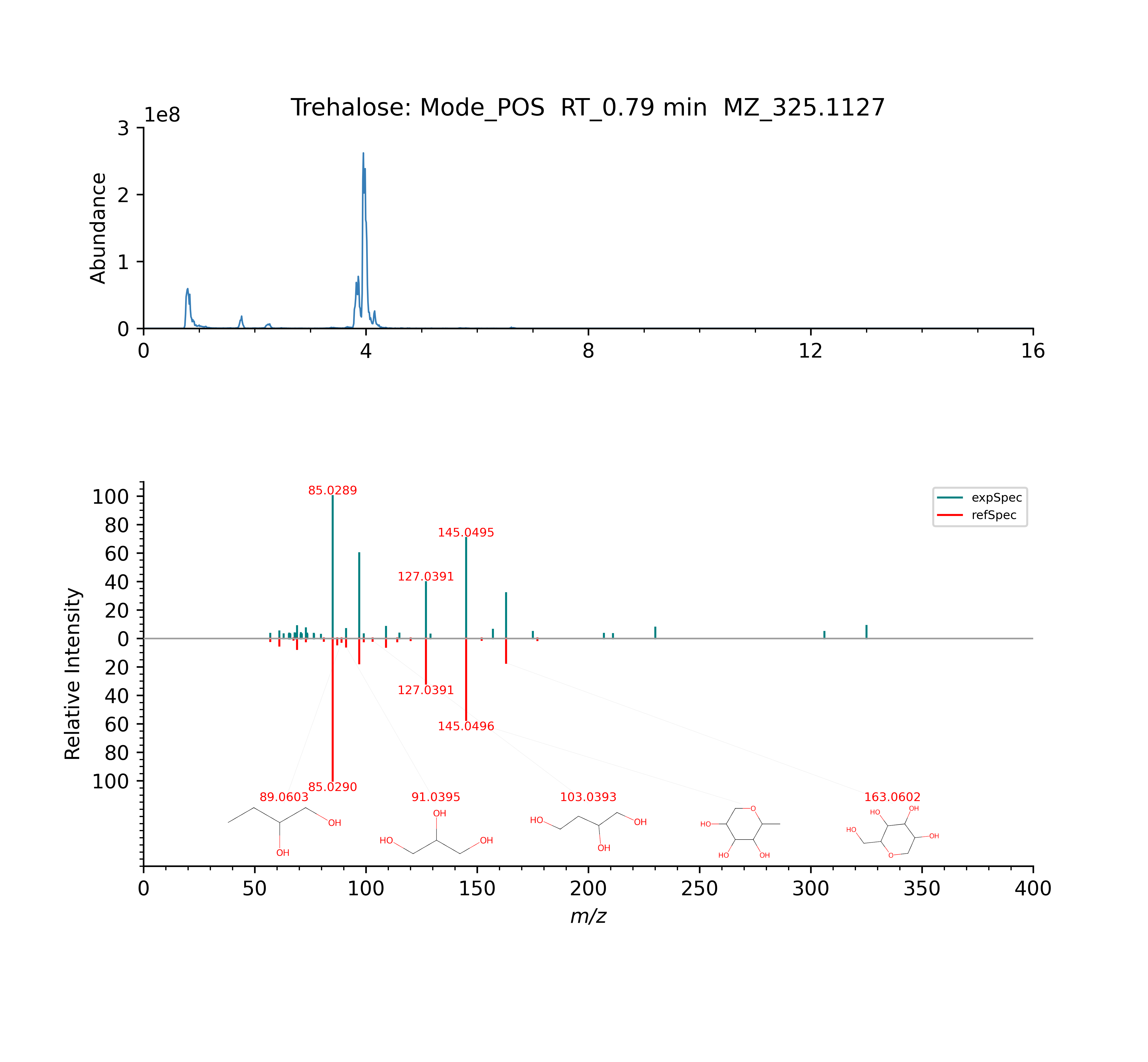

Supplement: Supplementary file 1 [file ijms-27-02203-s001.zip › ijms-4070482 Supplementary/Metabolite List Identified by LC-MS_MS from Rhodiola Species/111.png]

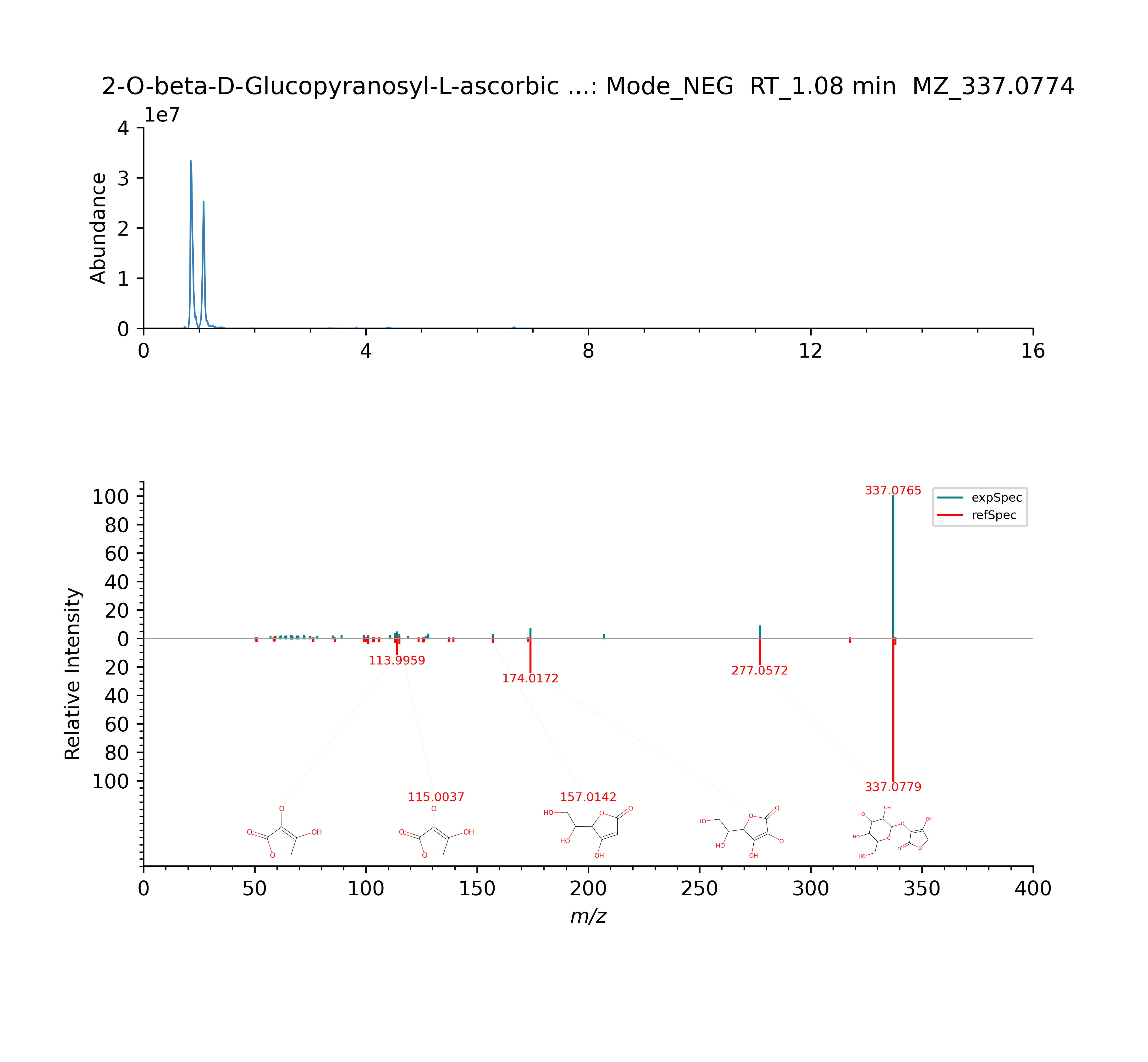

Supplement: Supplementary file 1 [file ijms-27-02203-s001.zip › ijms-4070482 Supplementary/Metabolite List Identified by LC-MS_MS from Rhodiola Species/112.png]

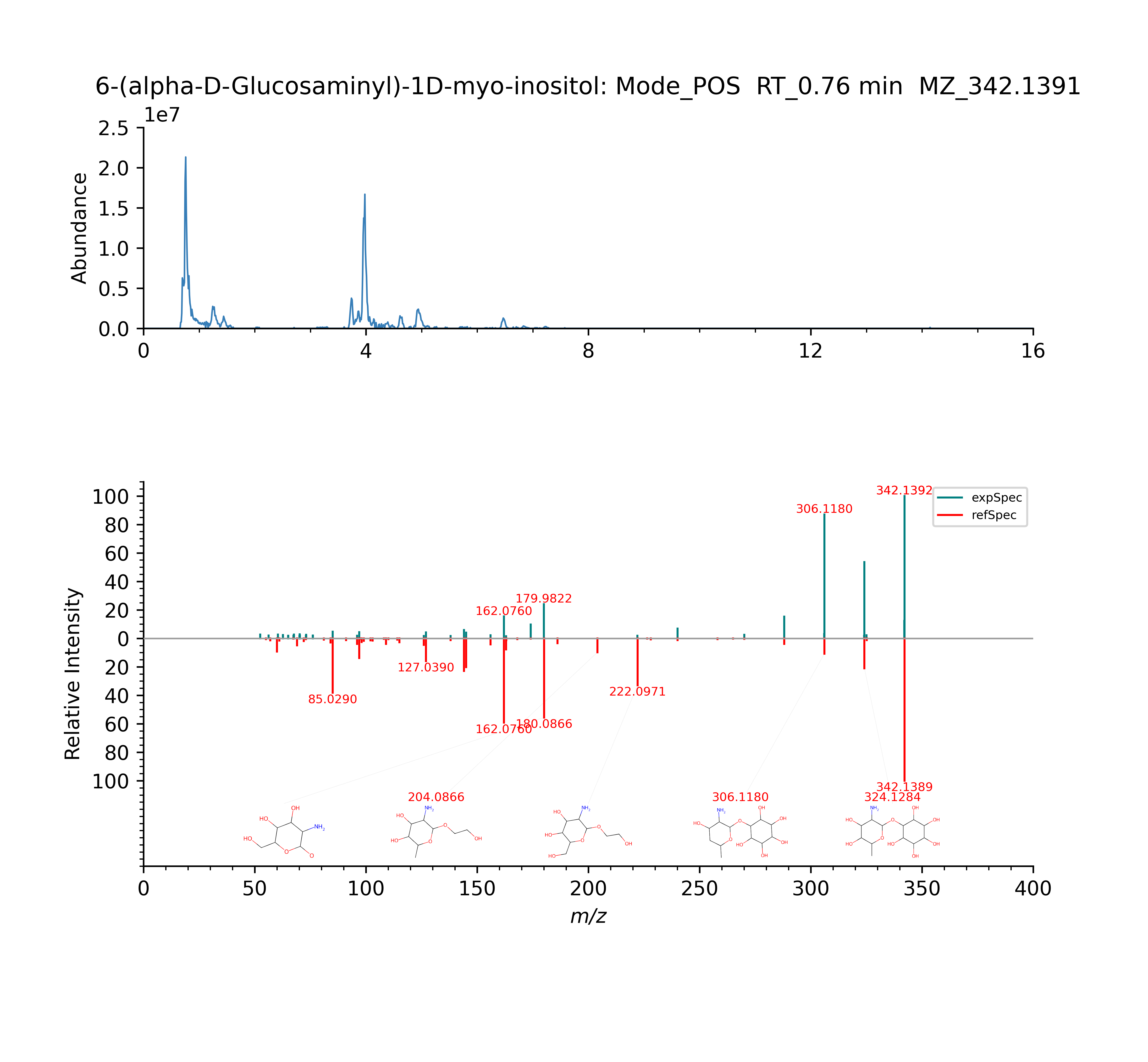

Supplement: Supplementary file 1 [file ijms-27-02203-s001.zip › ijms-4070482 Supplementary/Metabolite List Identified by LC-MS_MS from Rhodiola Species/113.png]

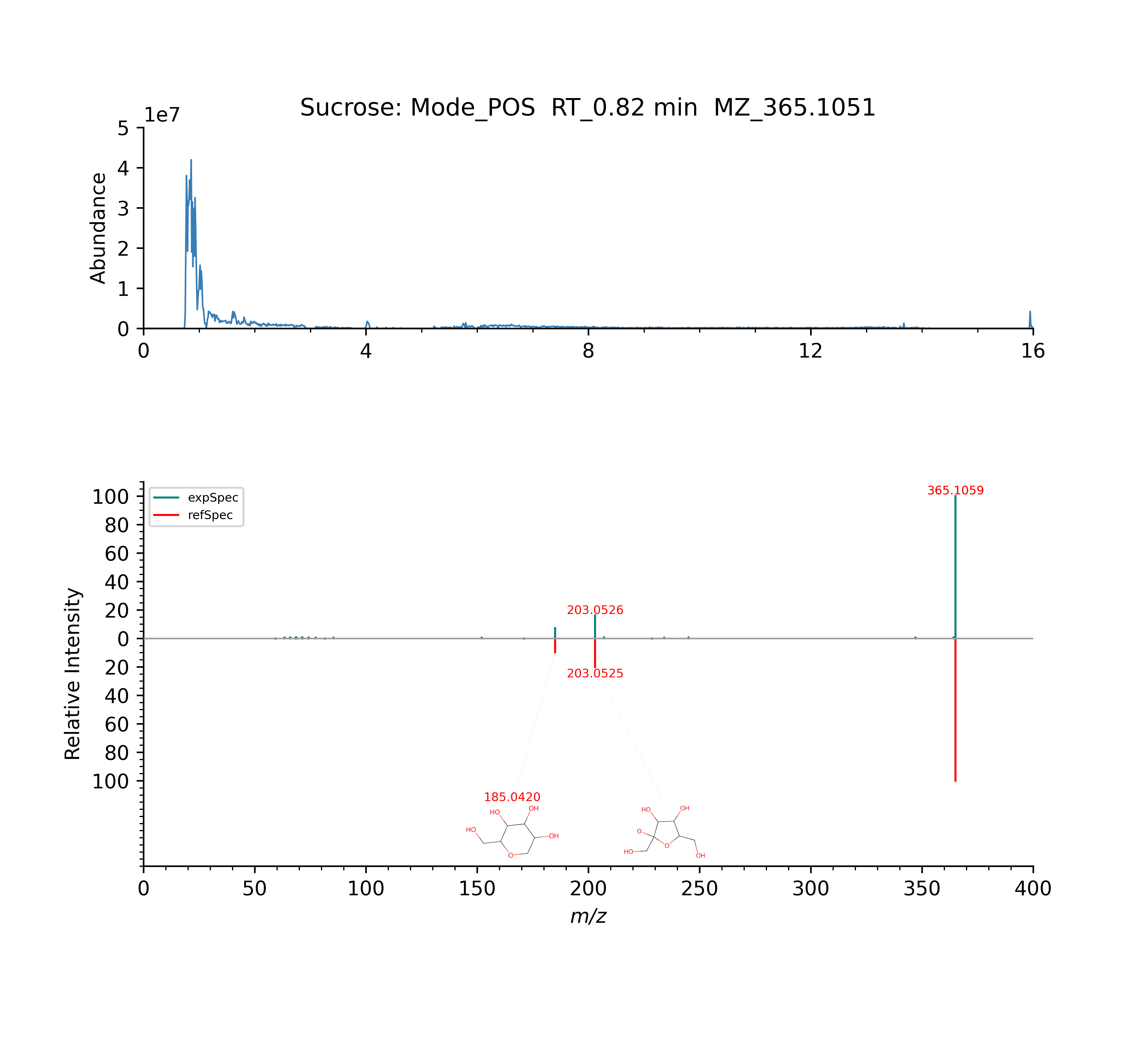

Supplement: Supplementary file 1 [file ijms-27-02203-s001.zip › ijms-4070482 Supplementary/Metabolite List Identified by LC-MS_MS from Rhodiola Species/114.png]

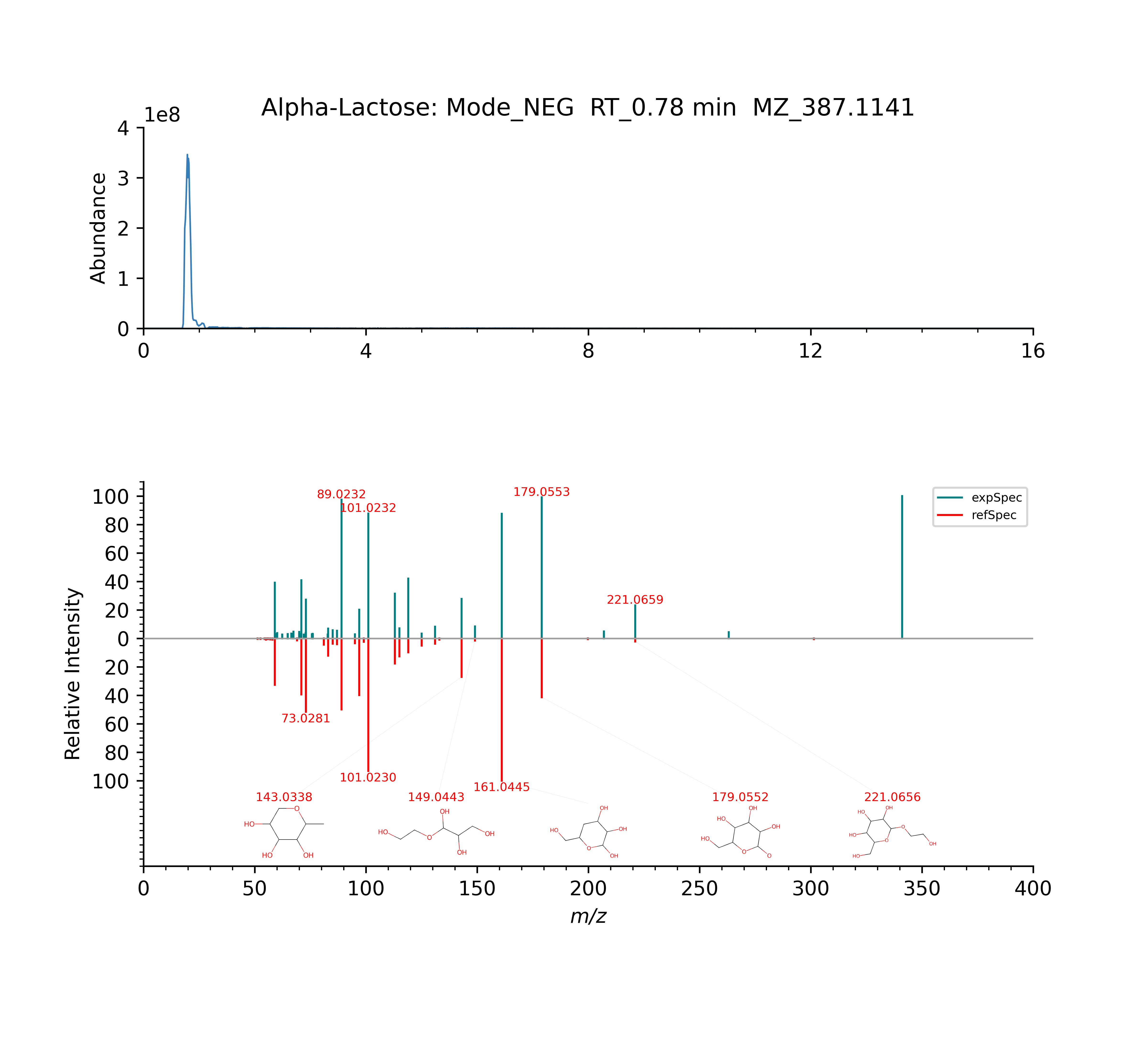

Supplement: Supplementary file 1 [file ijms-27-02203-s001.zip › ijms-4070482 Supplementary/Metabolite List Identified by LC-MS_MS from Rhodiola Species/115.png]

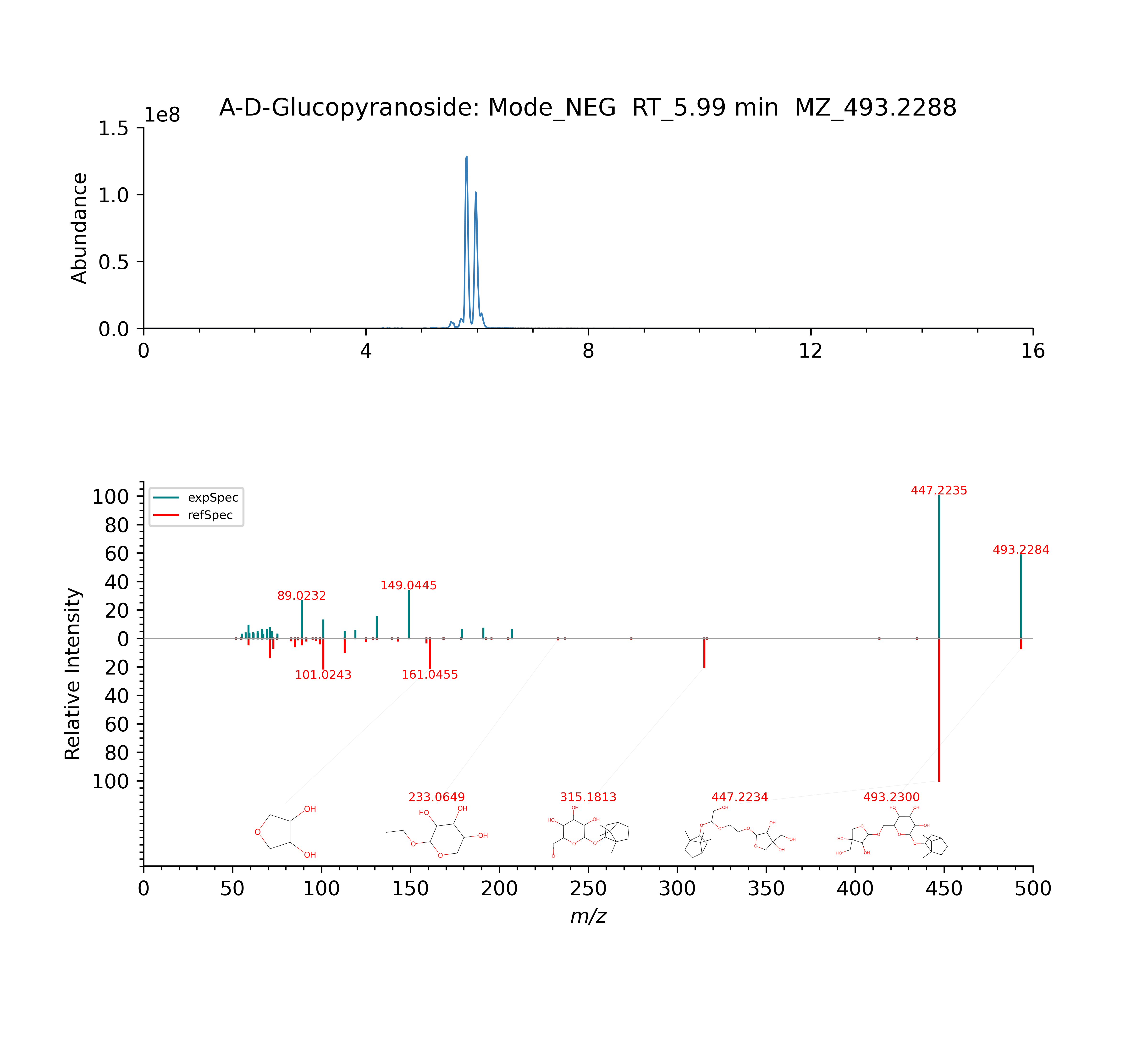

Supplement: Supplementary file 1 [file ijms-27-02203-s001.zip › ijms-4070482 Supplementary/Metabolite List Identified by LC-MS_MS from Rhodiola Species/116.png]

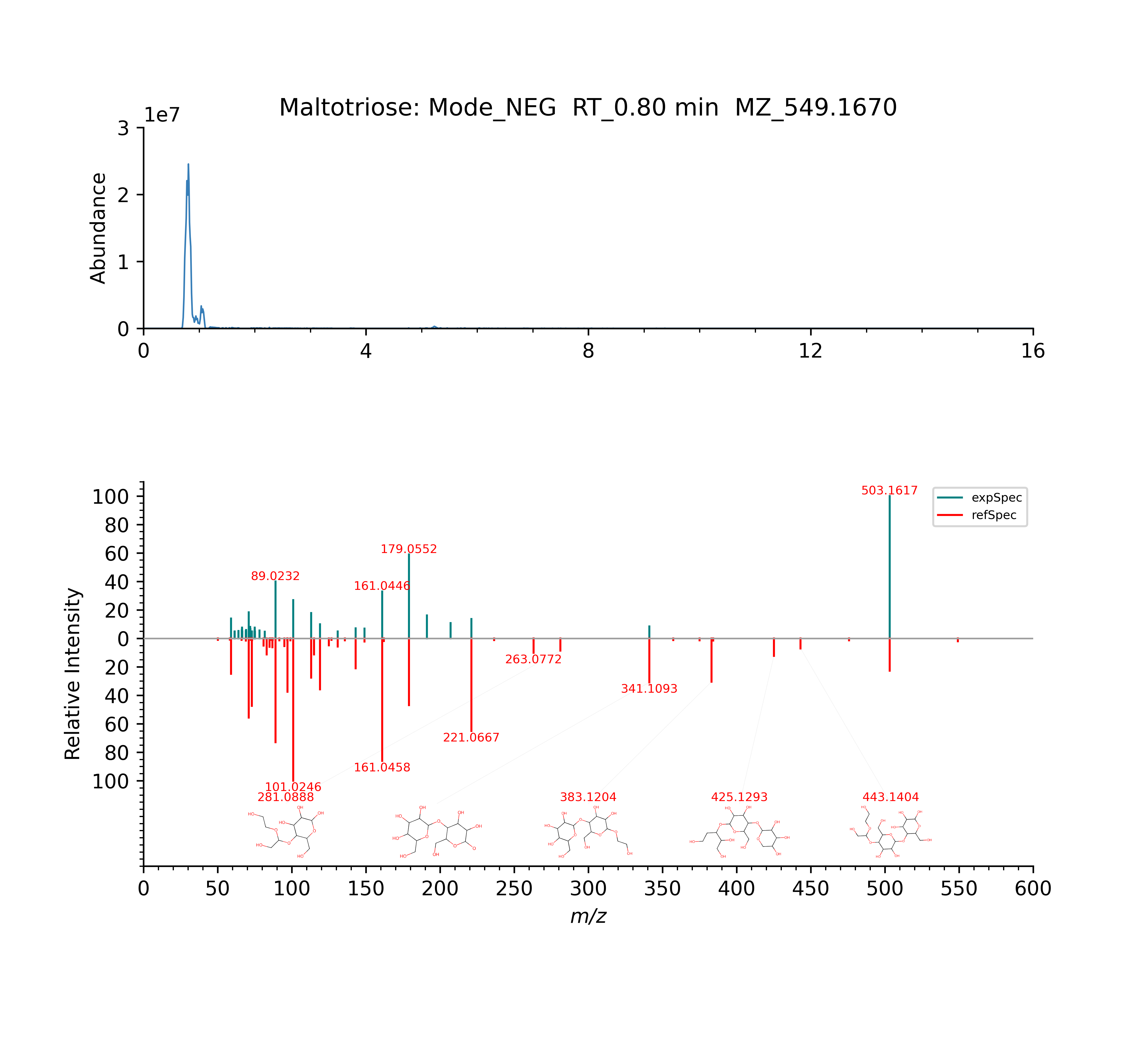

Supplement: Supplementary file 1 [file ijms-27-02203-s001.zip › ijms-4070482 Supplementary/Metabolite List Identified by LC-MS_MS from Rhodiola Species/117.png]

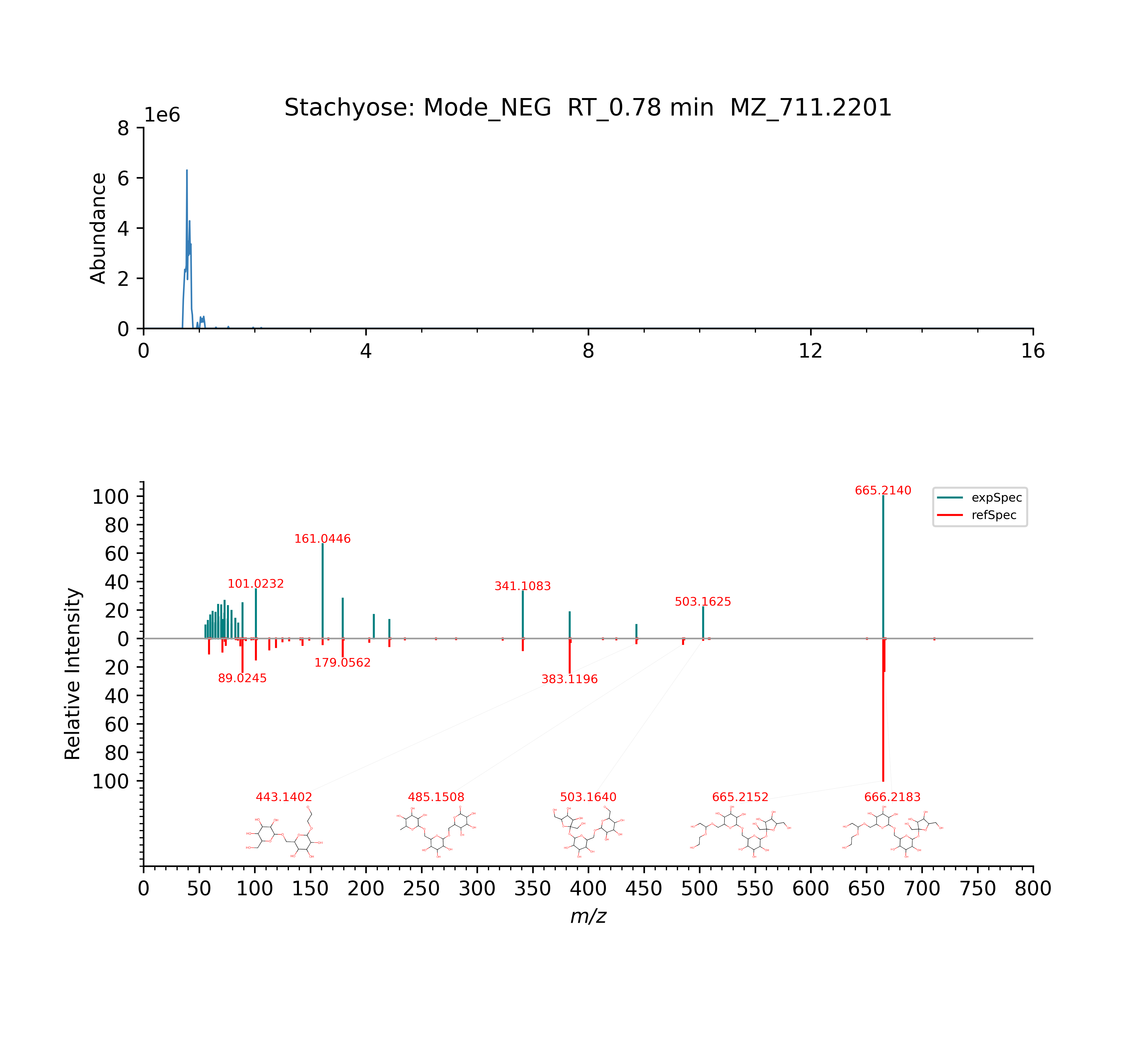

Supplement: Supplementary file 1 [file ijms-27-02203-s001.zip › ijms-4070482 Supplementary/Metabolite List Identified by LC-MS_MS from Rhodiola Species/118.png]

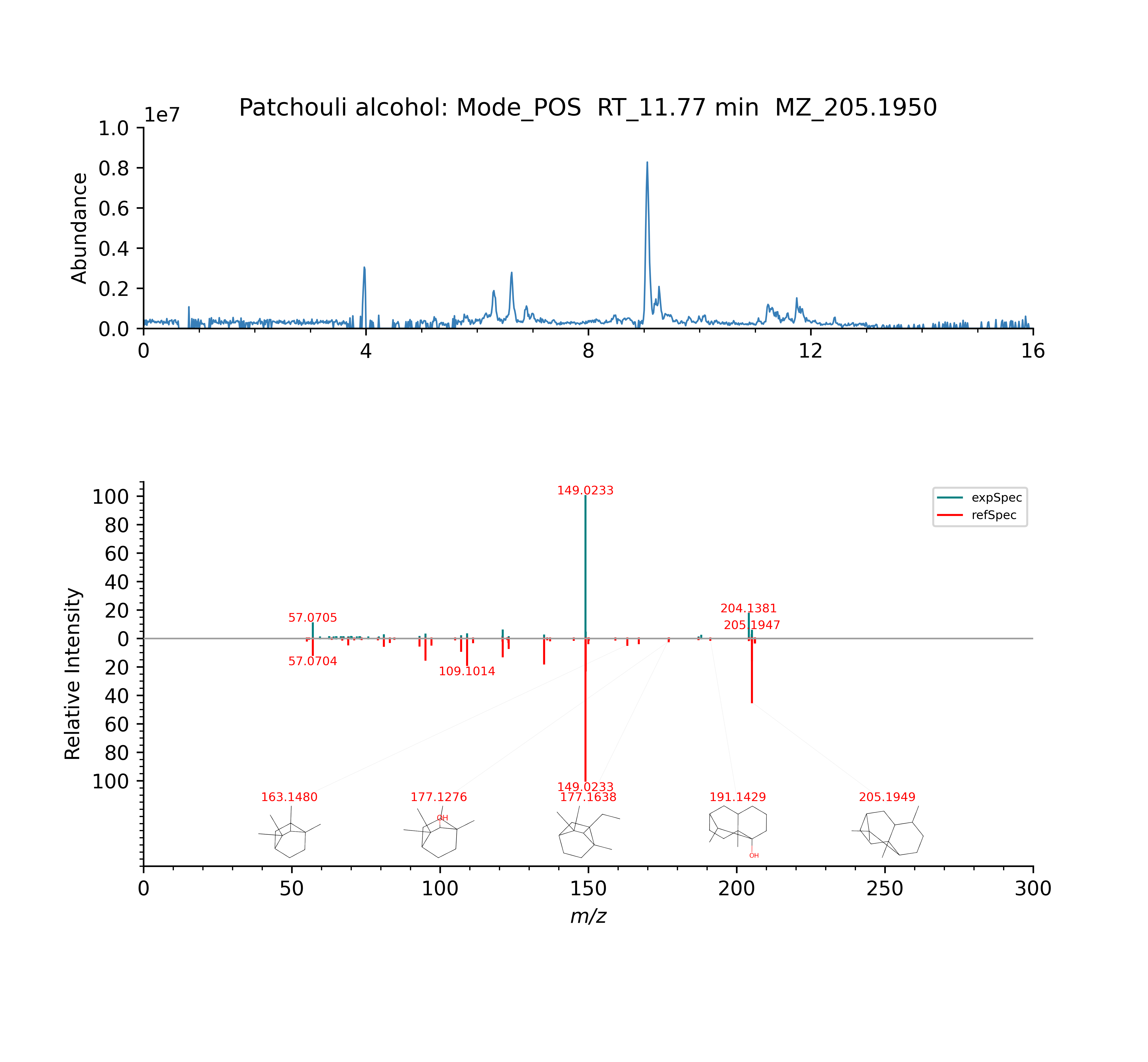

Supplement: Supplementary file 1 [file ijms-27-02203-s001.zip › ijms-4070482 Supplementary/Metabolite List Identified by LC-MS_MS from Rhodiola Species/119.png]

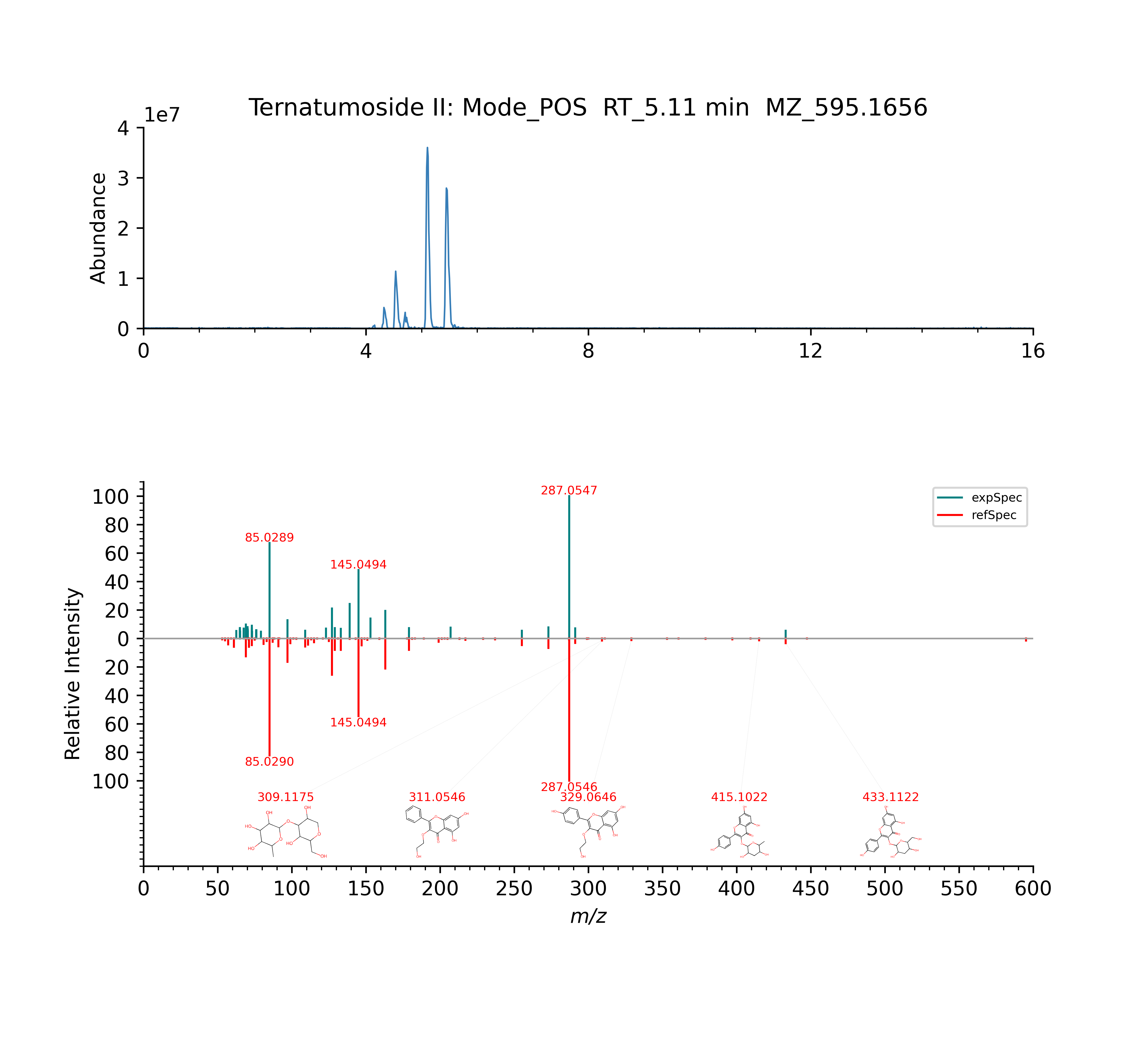

Supplement: Supplementary file 1 [file ijms-27-02203-s001.zip › ijms-4070482 Supplementary/Metabolite List Identified by LC-MS_MS from Rhodiola Species/12.png]

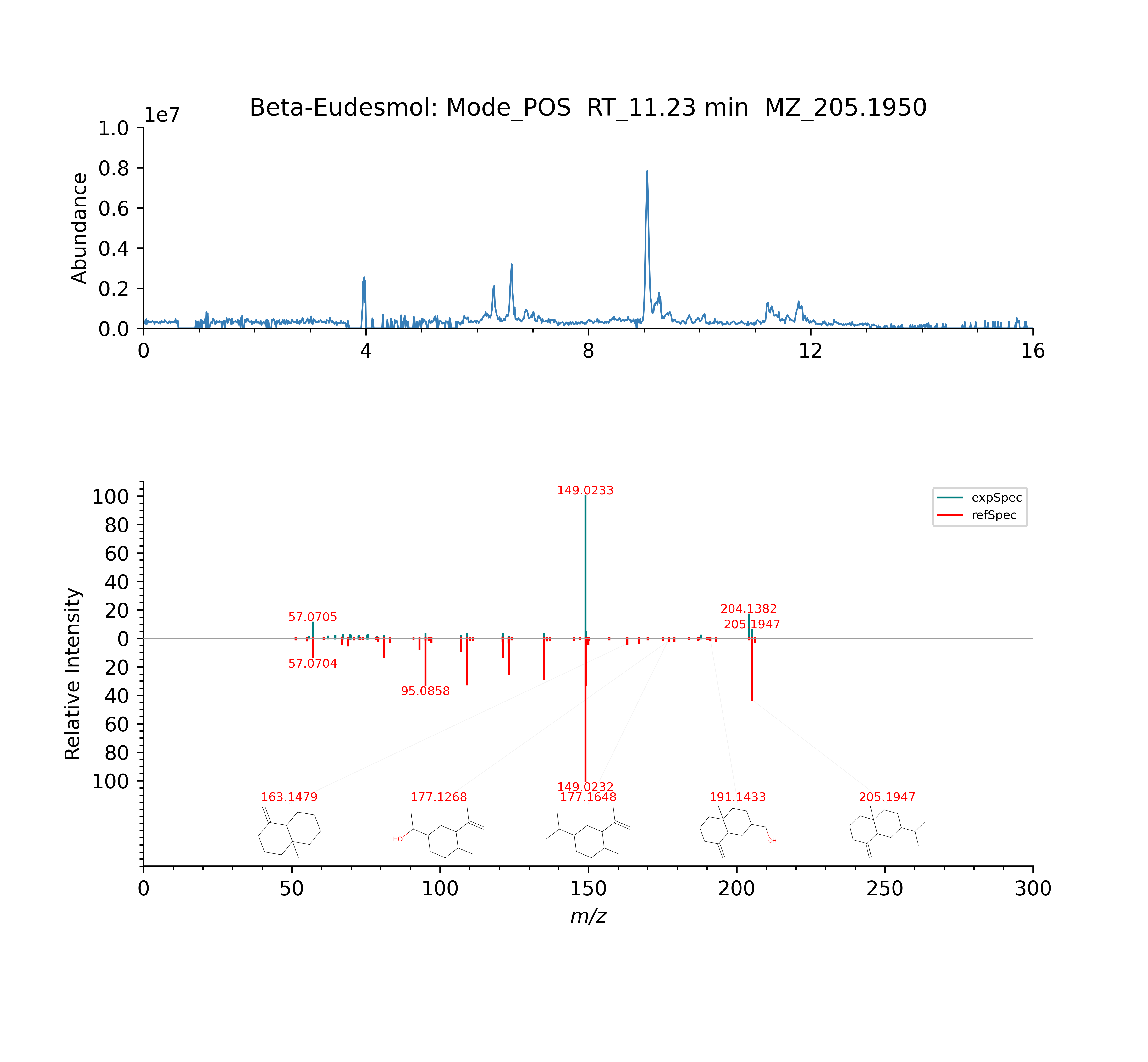

Supplement: Supplementary file 1 [file ijms-27-02203-s001.zip › ijms-4070482 Supplementary/Metabolite List Identified by LC-MS_MS from Rhodiola Species/120.png]

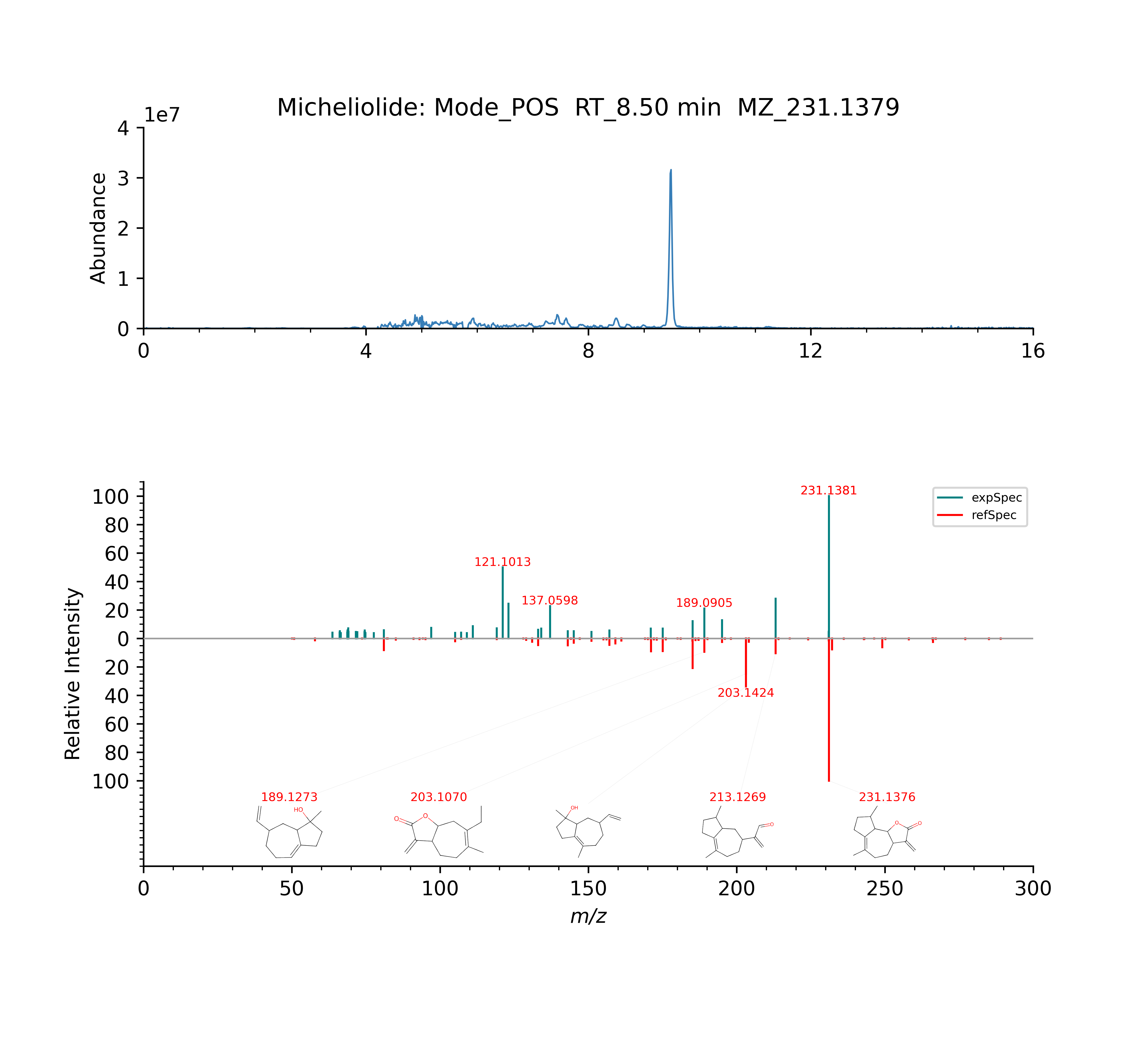

Supplement: Supplementary file 1 [file ijms-27-02203-s001.zip › ijms-4070482 Supplementary/Metabolite List Identified by LC-MS_MS from Rhodiola Species/121.png]

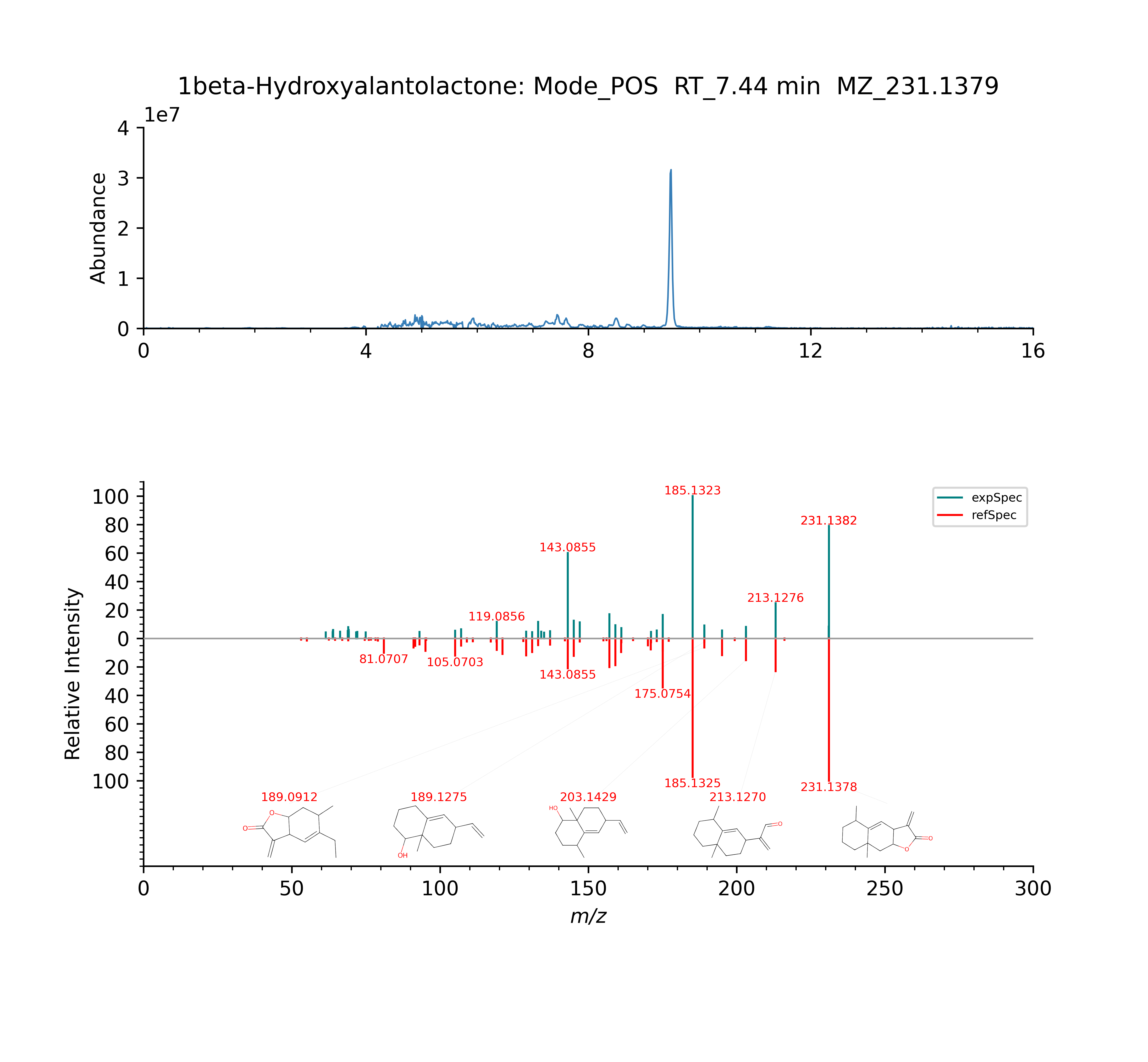

Supplement: Supplementary file 1 [file ijms-27-02203-s001.zip › ijms-4070482 Supplementary/Metabolite List Identified by LC-MS_MS from Rhodiola Species/122.png]

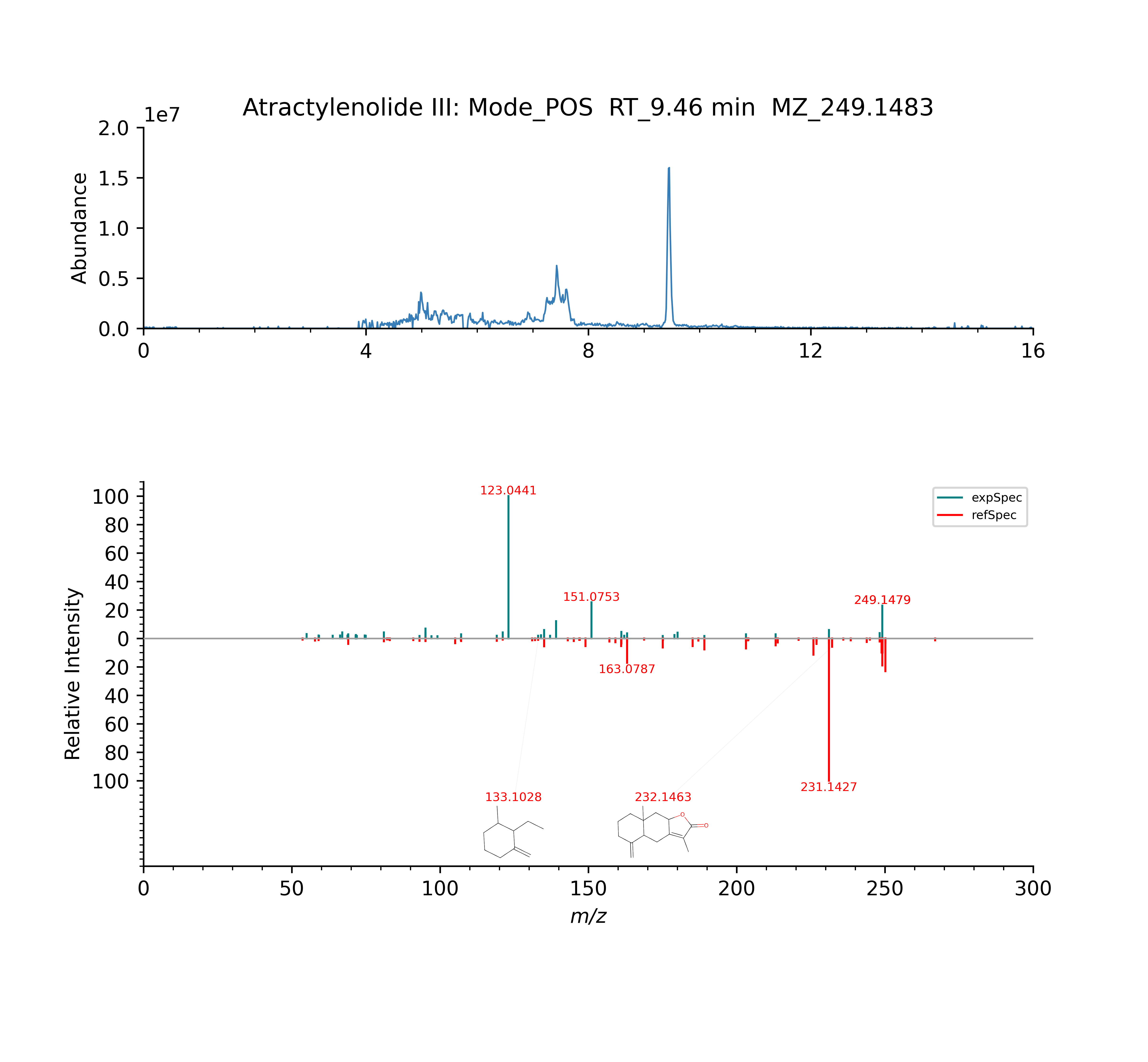

Supplement: Supplementary file 1 [file ijms-27-02203-s001.zip › ijms-4070482 Supplementary/Metabolite List Identified by LC-MS_MS from Rhodiola Species/123.png]

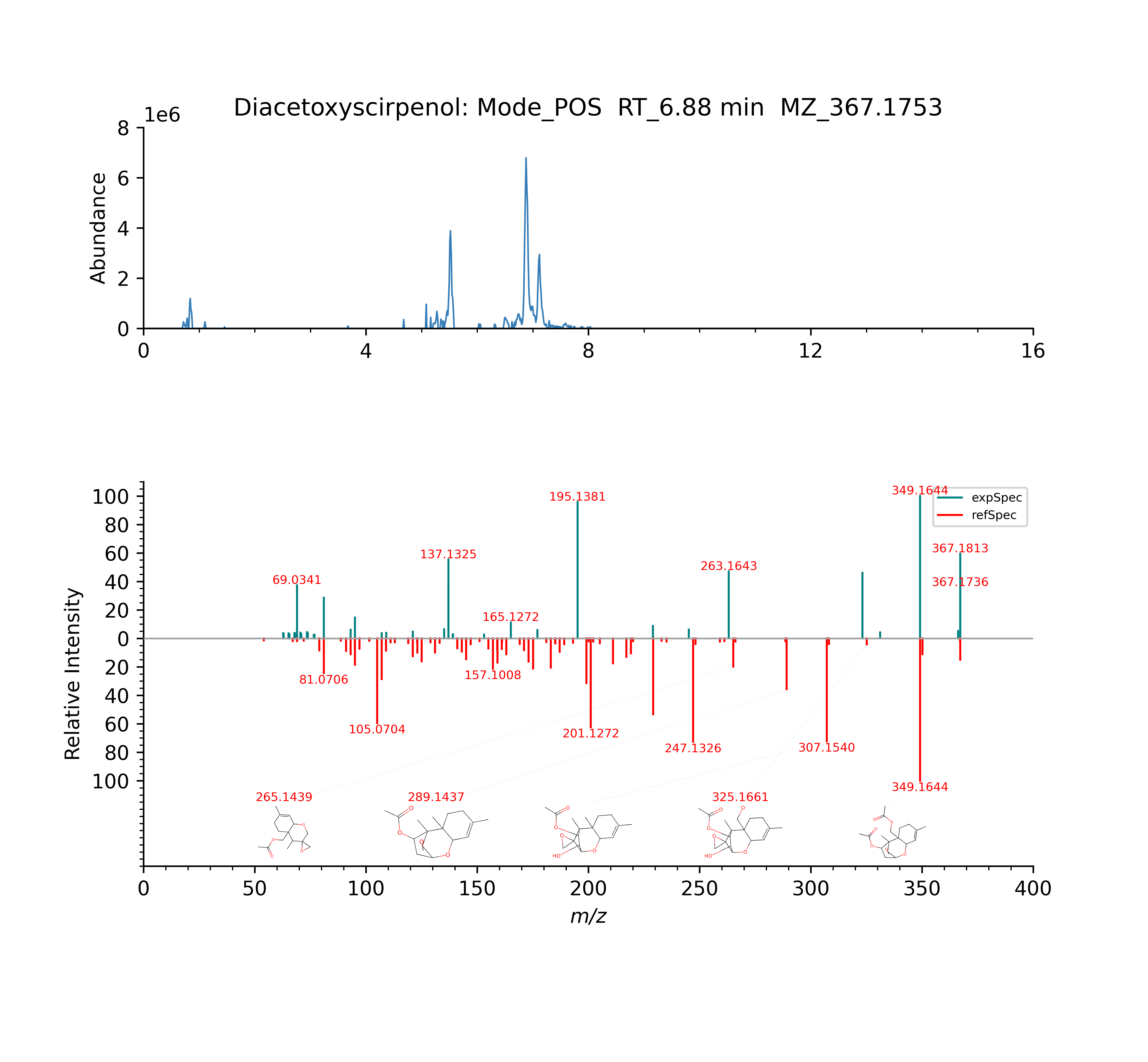

Supplement: Supplementary file 1 [file ijms-27-02203-s001.zip › ijms-4070482 Supplementary/Metabolite List Identified by LC-MS_MS from Rhodiola Species/124.png]

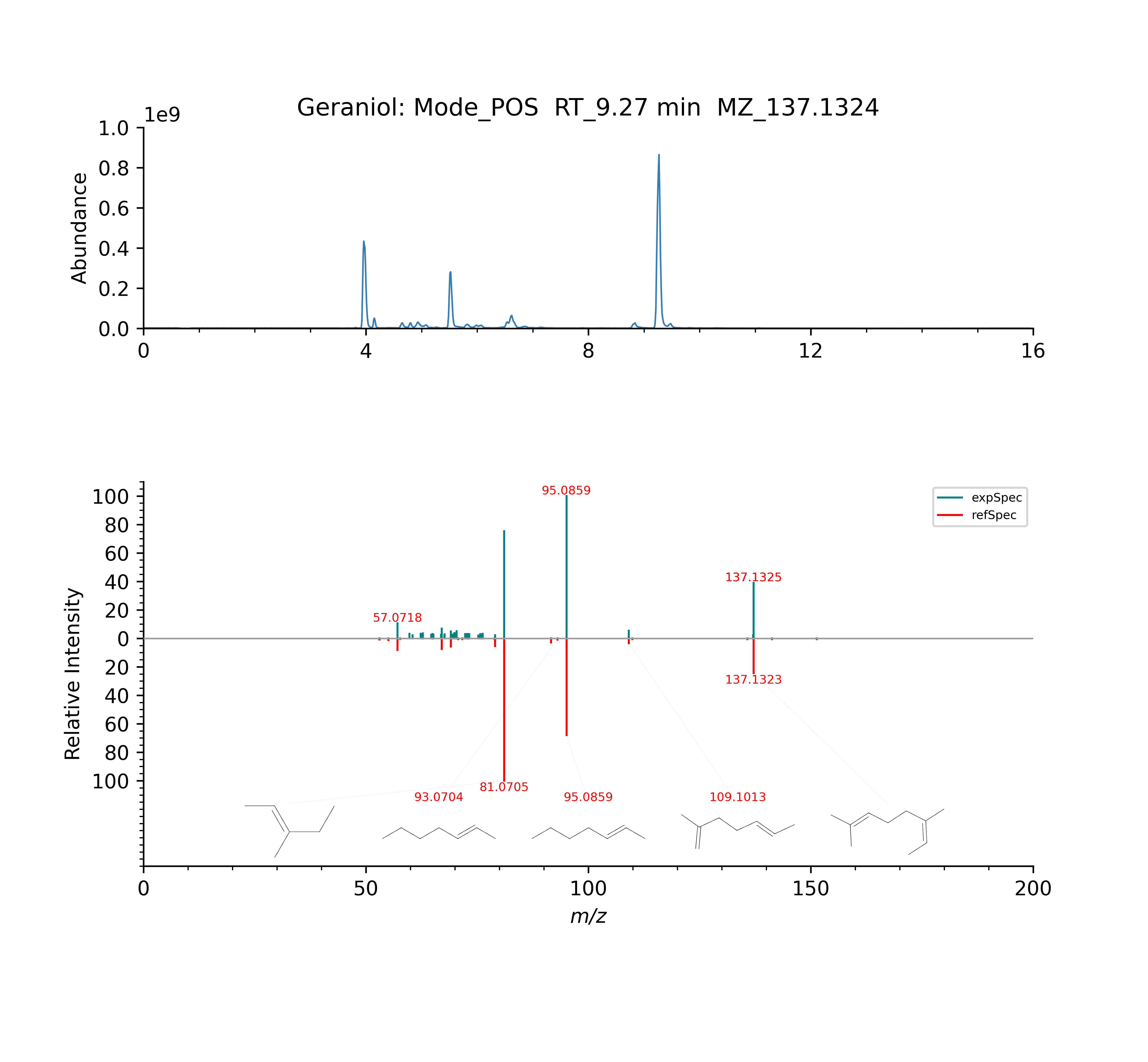

Supplement: Supplementary file 1 [file ijms-27-02203-s001.zip › ijms-4070482 Supplementary/Metabolite List Identified by LC-MS_MS from Rhodiola Species/125.png]

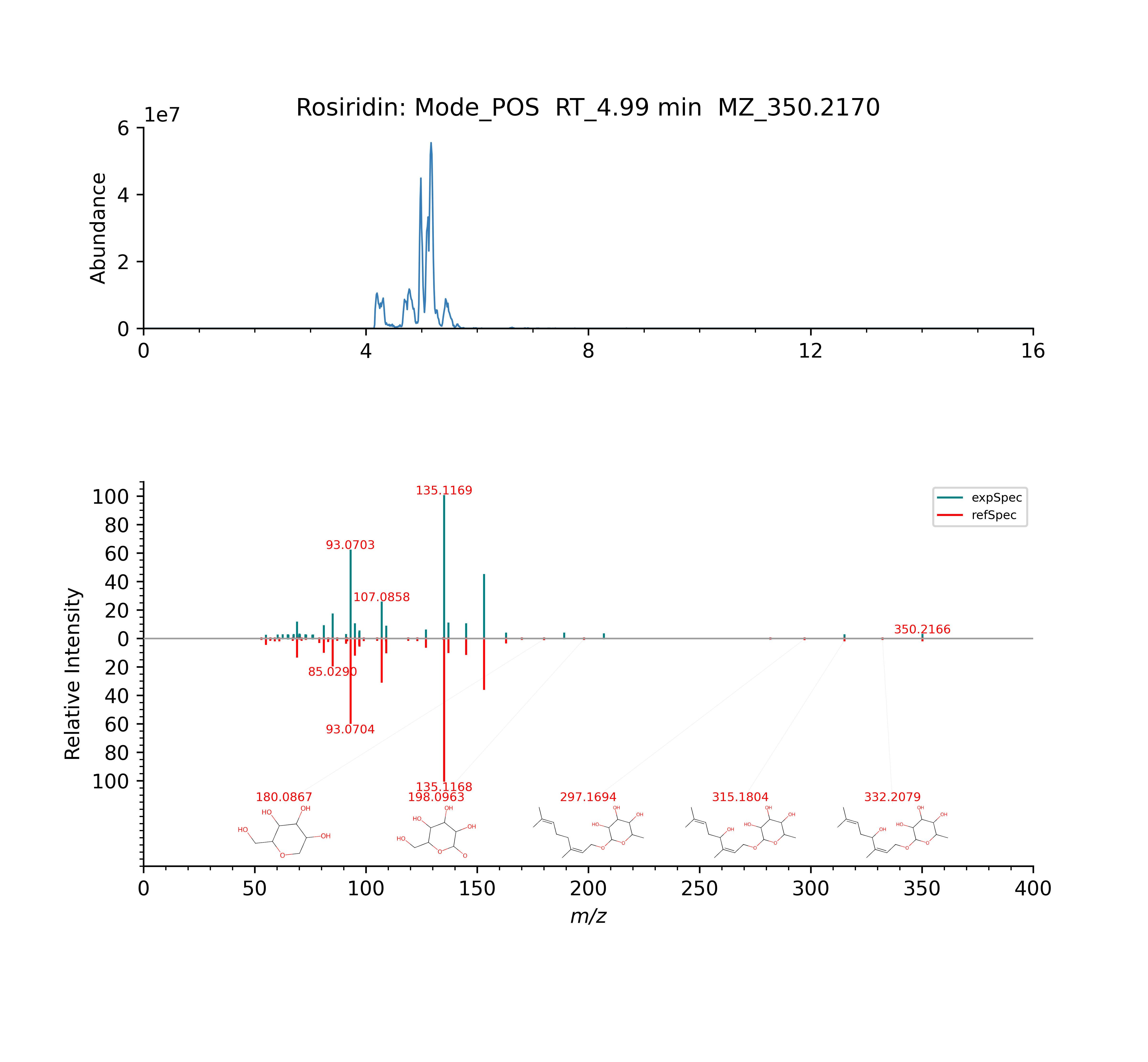

Supplement: Supplementary file 1 [file ijms-27-02203-s001.zip › ijms-4070482 Supplementary/Metabolite List Identified by LC-MS_MS from Rhodiola Species/126.png]

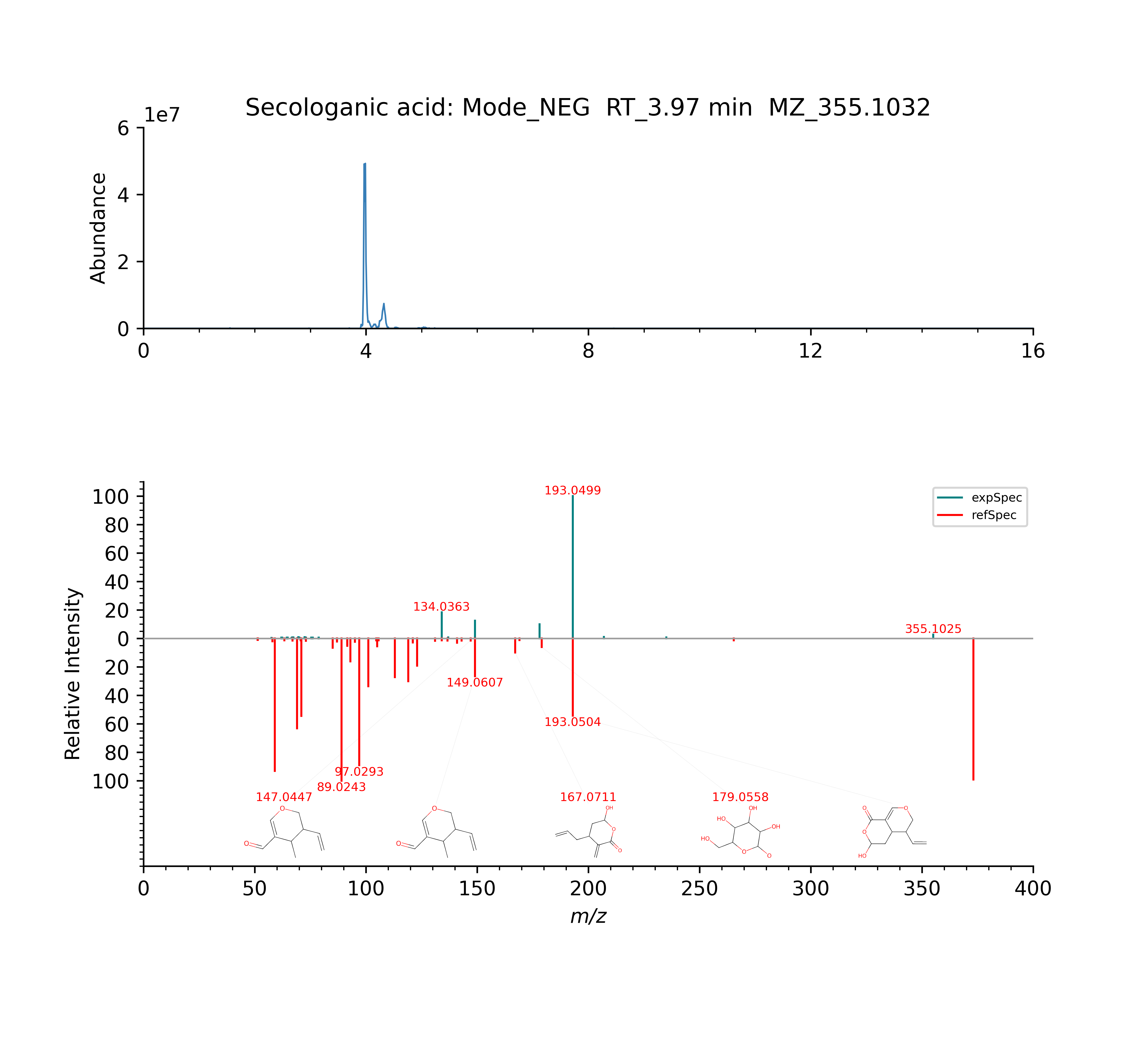

Supplement: Supplementary file 1 [file ijms-27-02203-s001.zip › ijms-4070482 Supplementary/Metabolite List Identified by LC-MS_MS from Rhodiola Species/127.png]

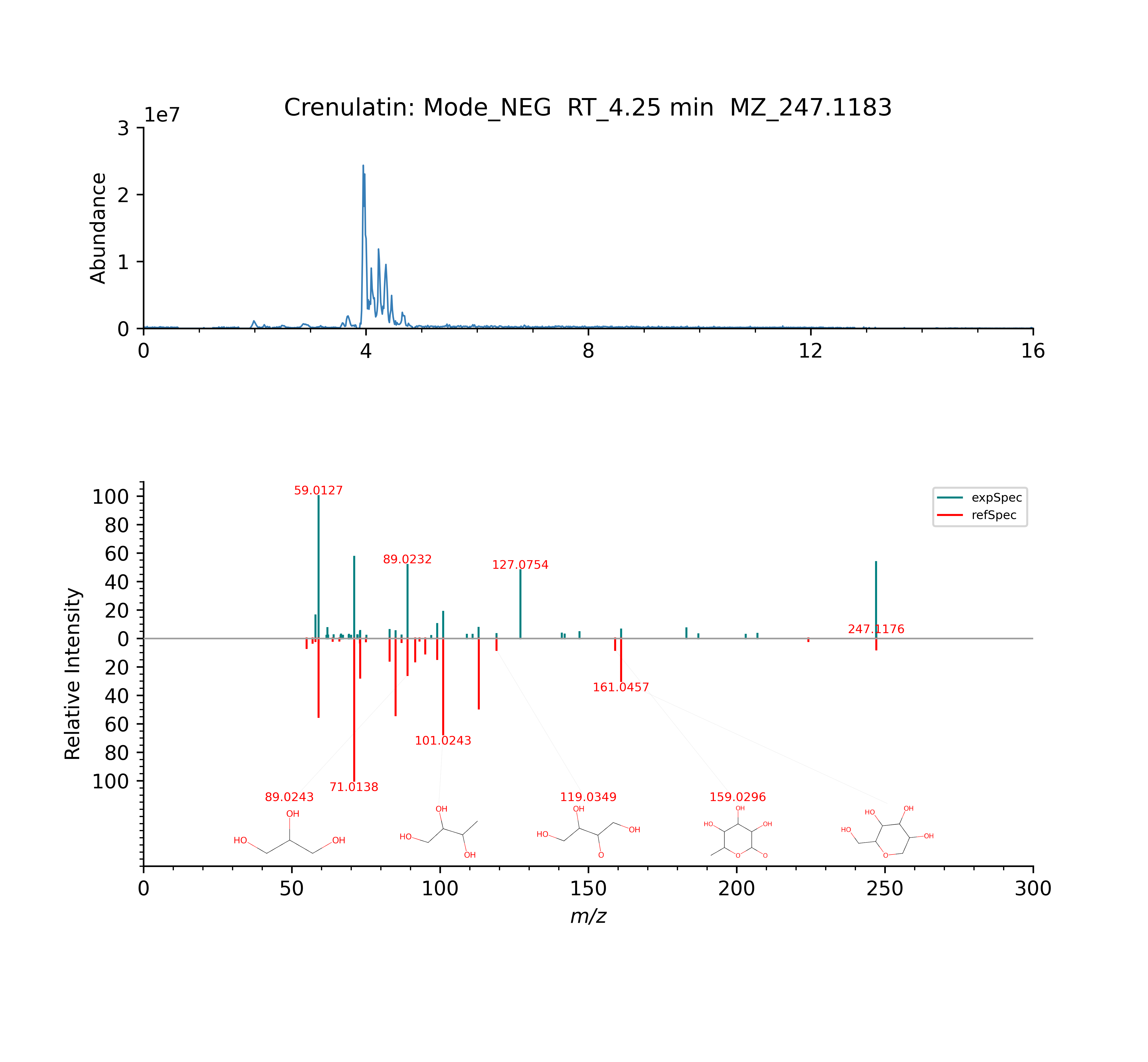

Supplement: Supplementary file 1 [file ijms-27-02203-s001.zip › ijms-4070482 Supplementary/Metabolite List Identified by LC-MS_MS from Rhodiola Species/128.png]

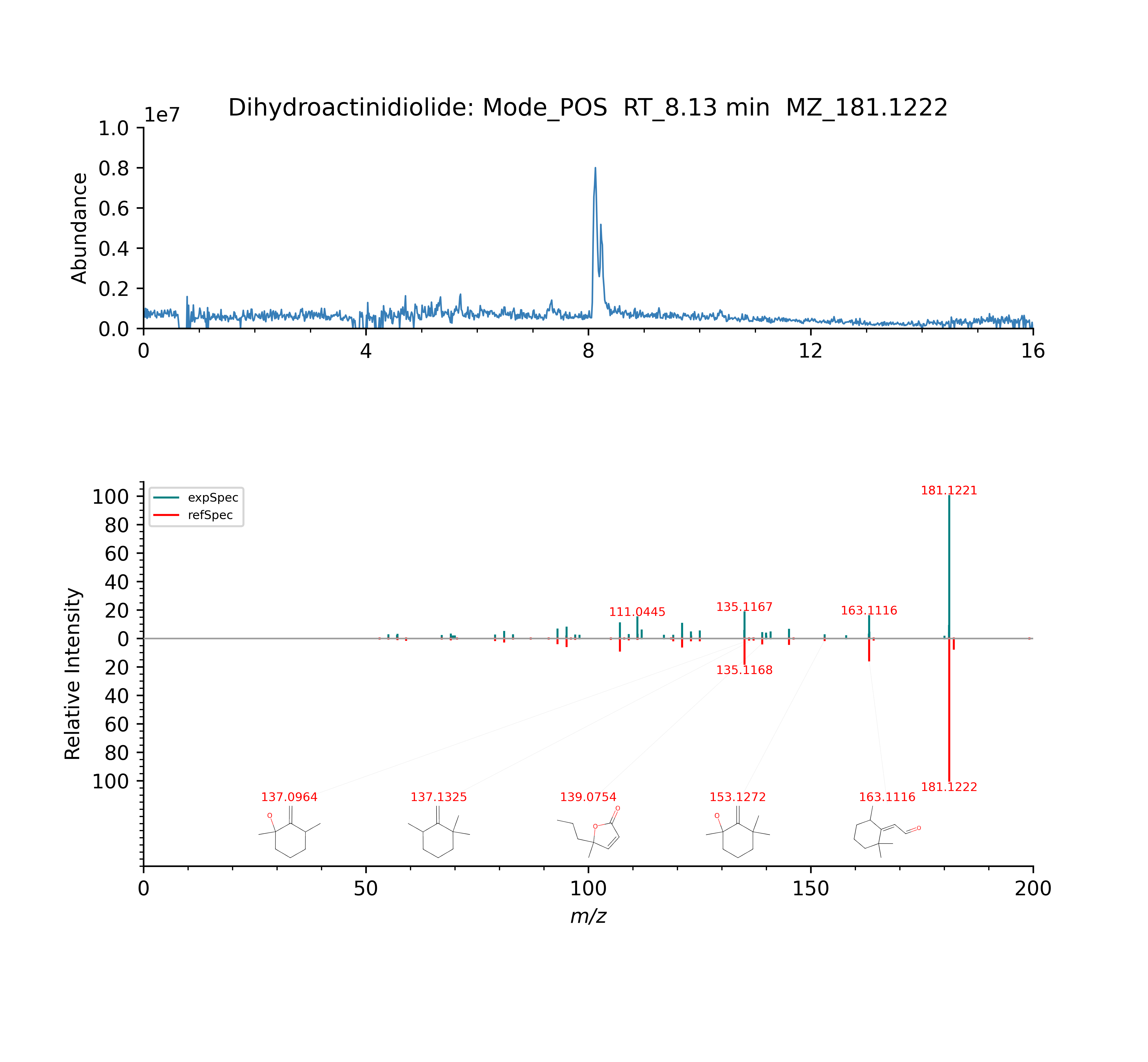

Supplement: Supplementary file 1 [file ijms-27-02203-s001.zip › ijms-4070482 Supplementary/Metabolite List Identified by LC-MS_MS from Rhodiola Species/129.png]

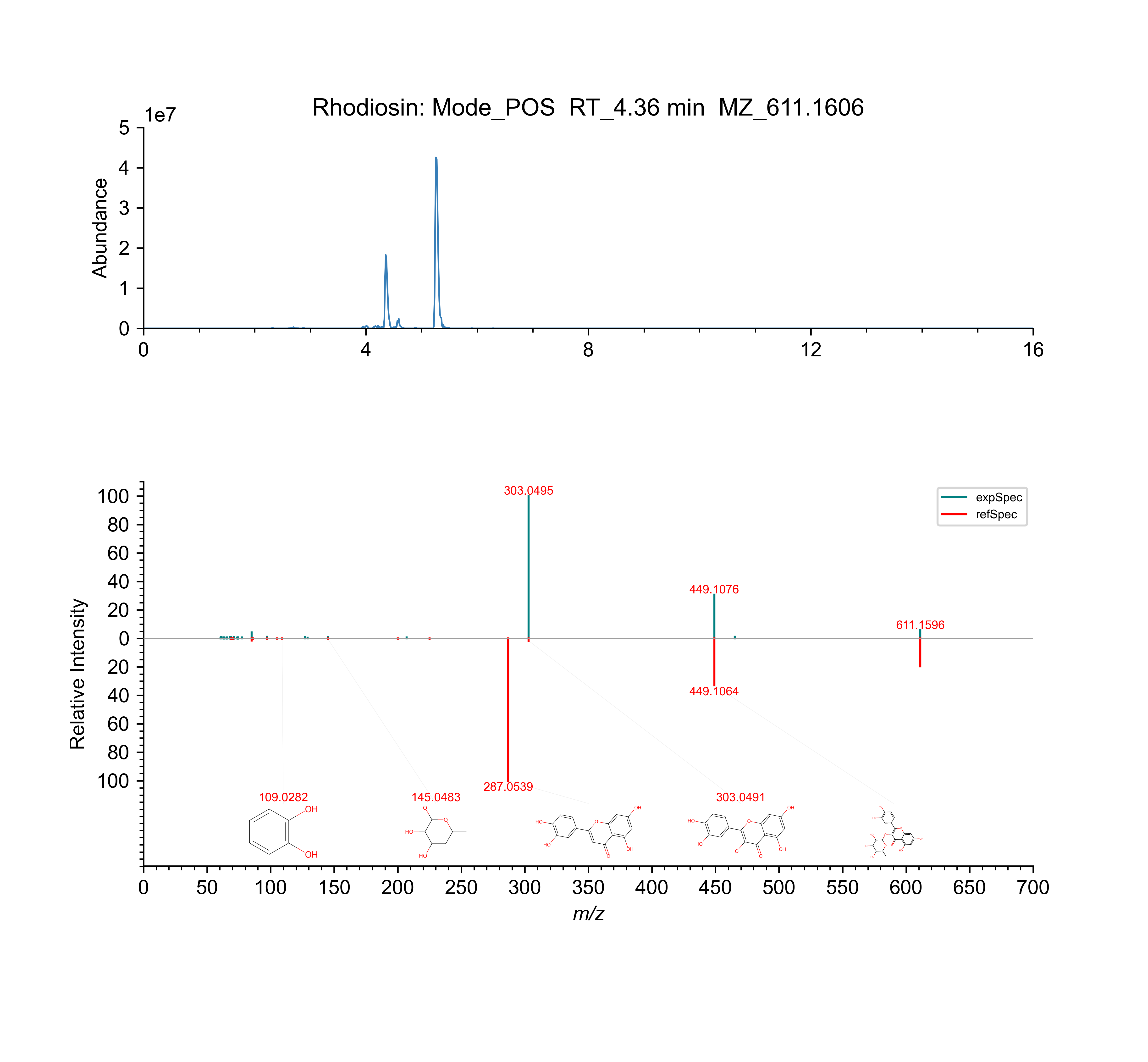

Supplement: Supplementary file 1 [file ijms-27-02203-s001.zip › ijms-4070482 Supplementary/Metabolite List Identified by LC-MS_MS from Rhodiola Species/13.png]

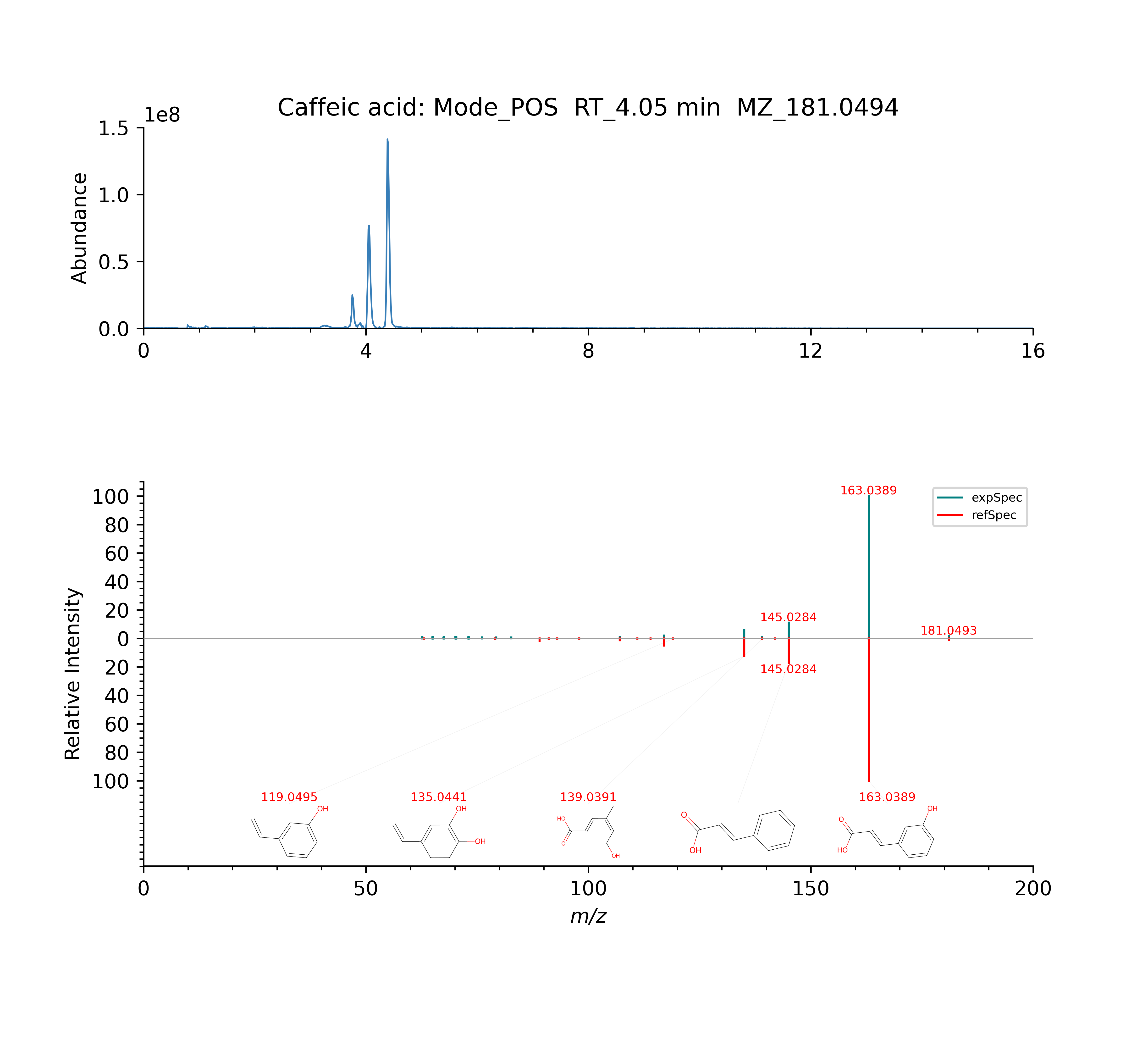

Supplement: Supplementary file 1 [file ijms-27-02203-s001.zip › ijms-4070482 Supplementary/Metabolite List Identified by LC-MS_MS from Rhodiola Species/130.png]

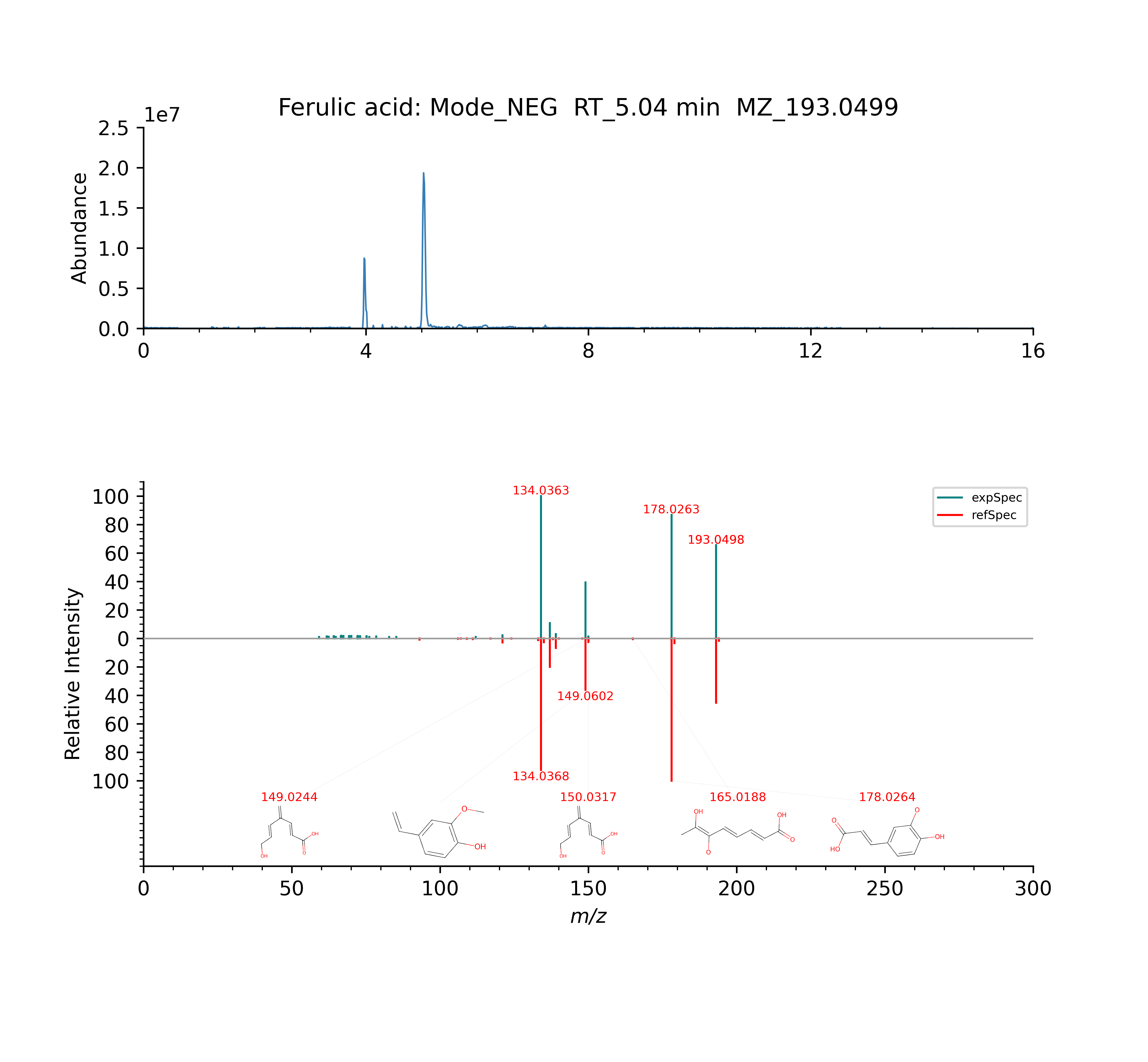

Supplement: Supplementary file 1 [file ijms-27-02203-s001.zip › ijms-4070482 Supplementary/Metabolite List Identified by LC-MS_MS from Rhodiola Species/131.png]

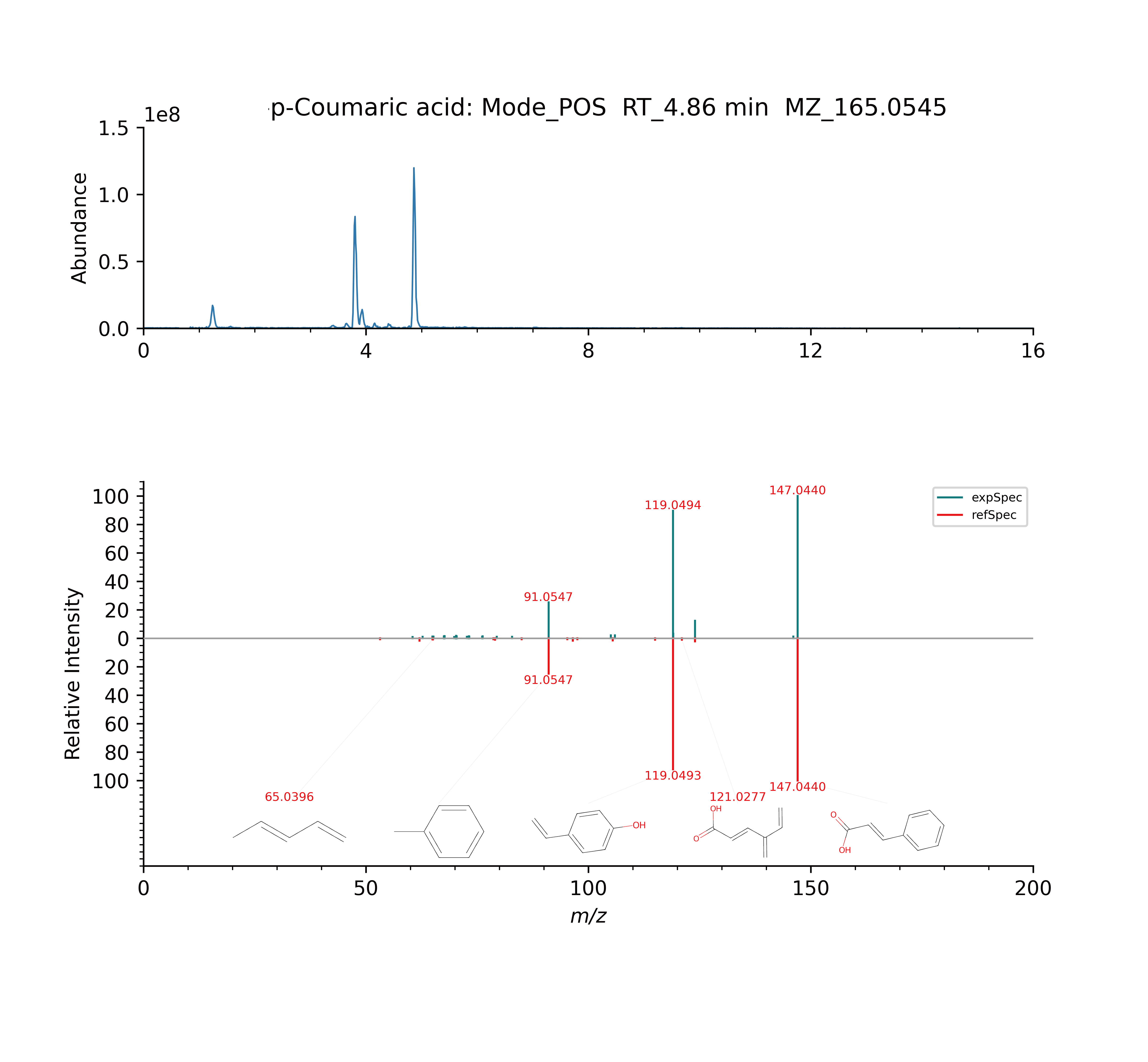

Supplement: Supplementary file 1 [file ijms-27-02203-s001.zip › ijms-4070482 Supplementary/Metabolite List Identified by LC-MS_MS from Rhodiola Species/132.png]

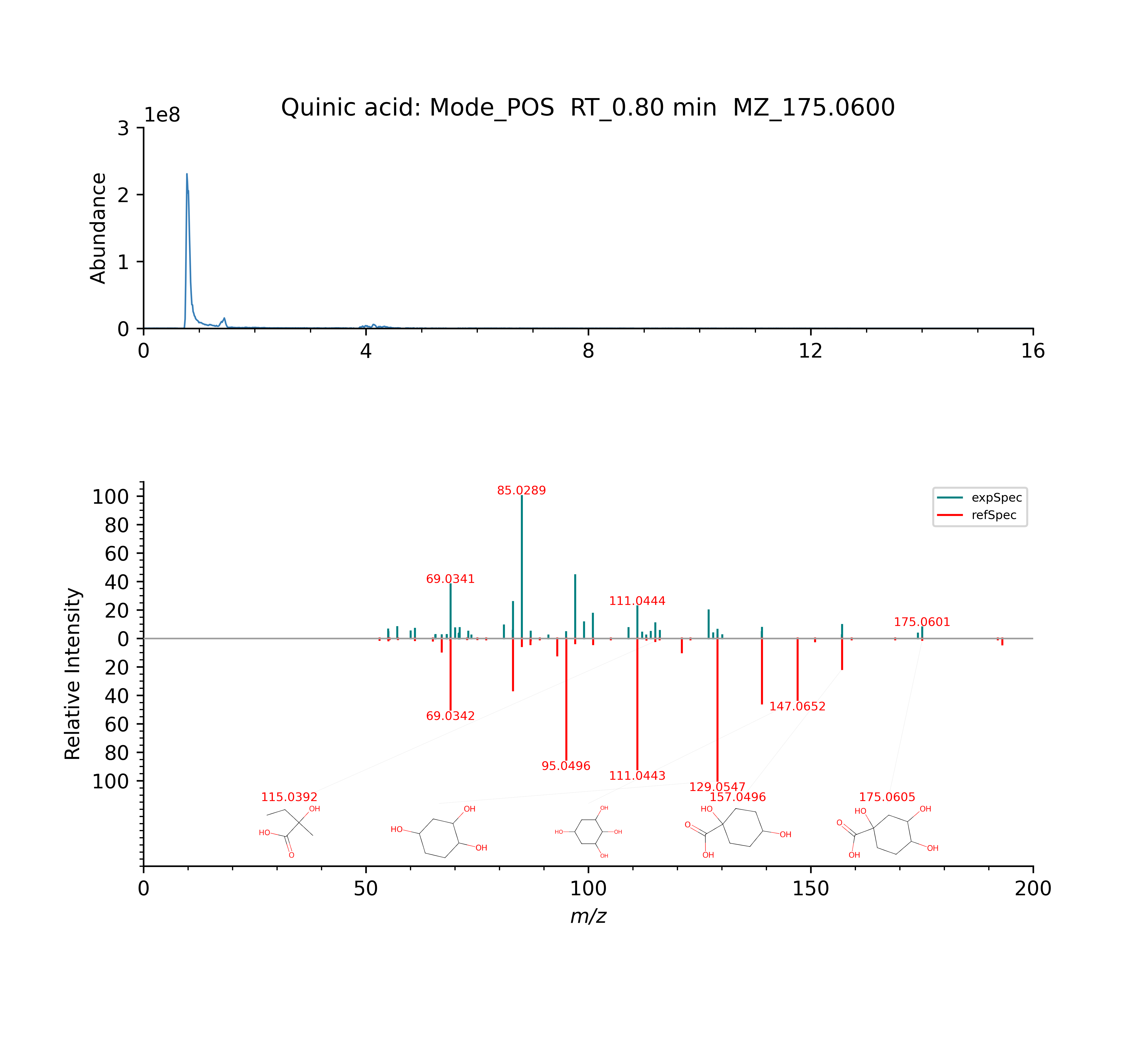

Supplement: Supplementary file 1 [file ijms-27-02203-s001.zip › ijms-4070482 Supplementary/Metabolite List Identified by LC-MS_MS from Rhodiola Species/133.png]

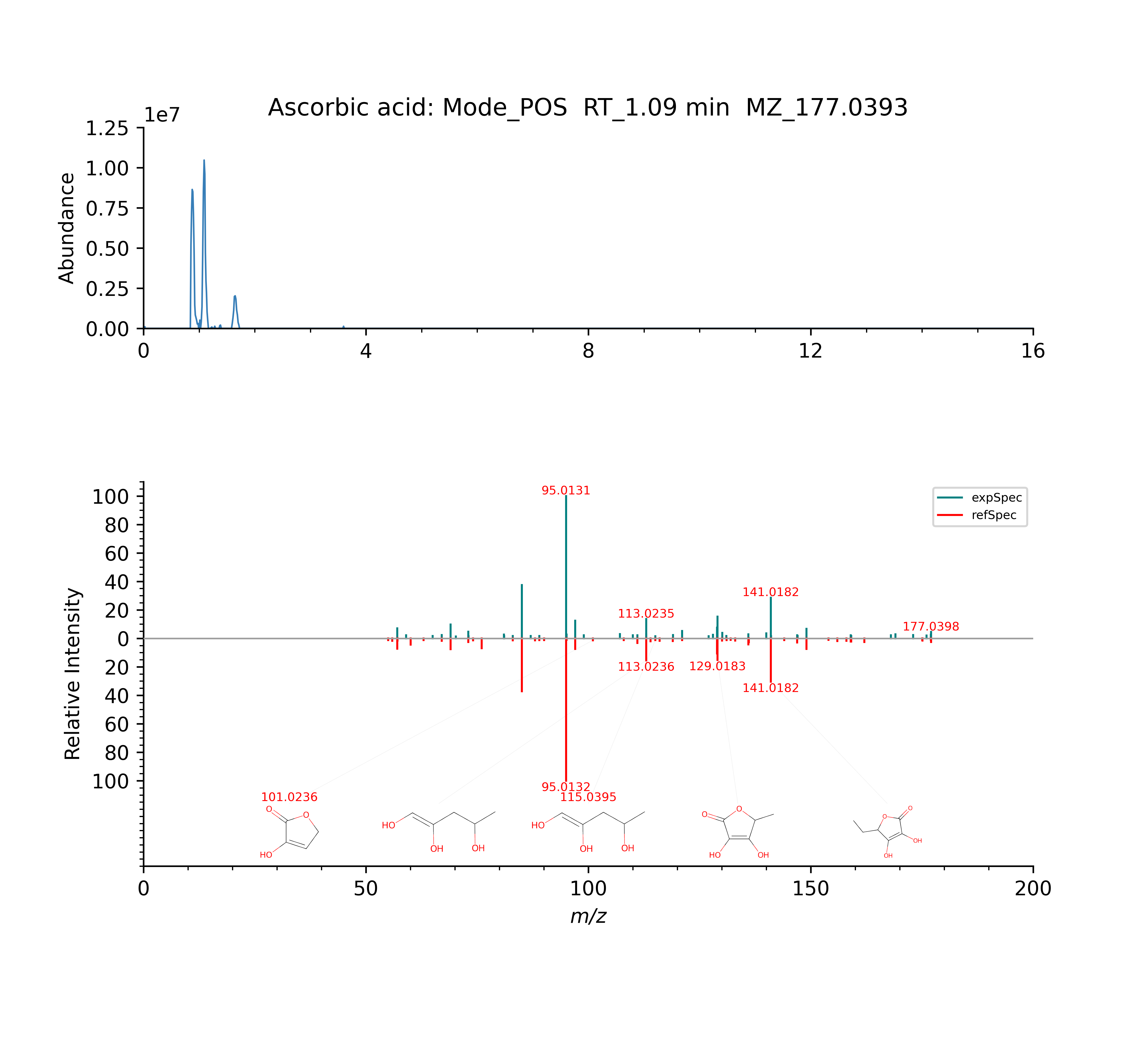

Supplement: Supplementary file 1 [file ijms-27-02203-s001.zip › ijms-4070482 Supplementary/Metabolite List Identified by LC-MS_MS from Rhodiola Species/134.png]

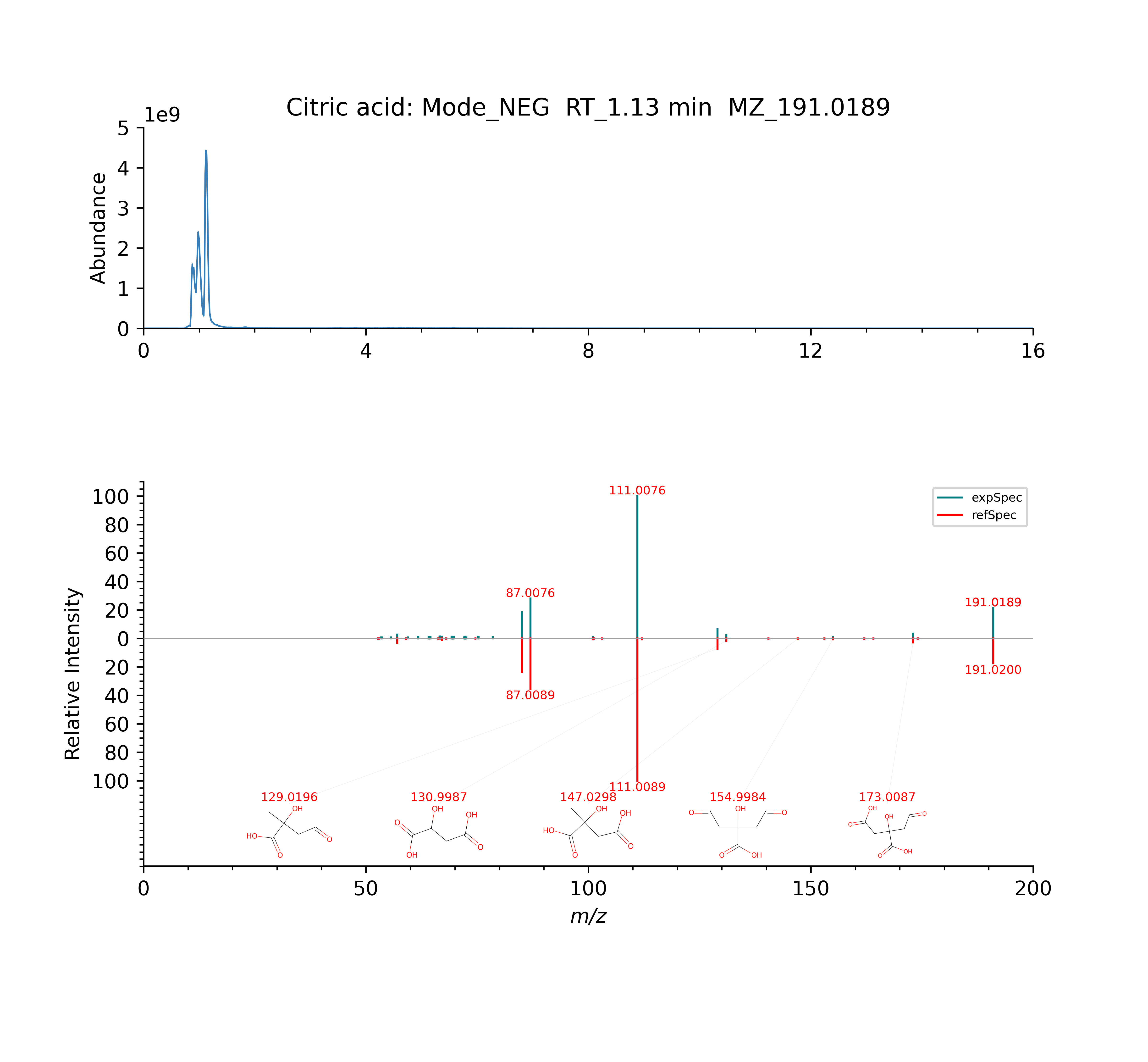

Supplement: Supplementary file 1 [file ijms-27-02203-s001.zip › ijms-4070482 Supplementary/Metabolite List Identified by LC-MS_MS from Rhodiola Species/135.png]

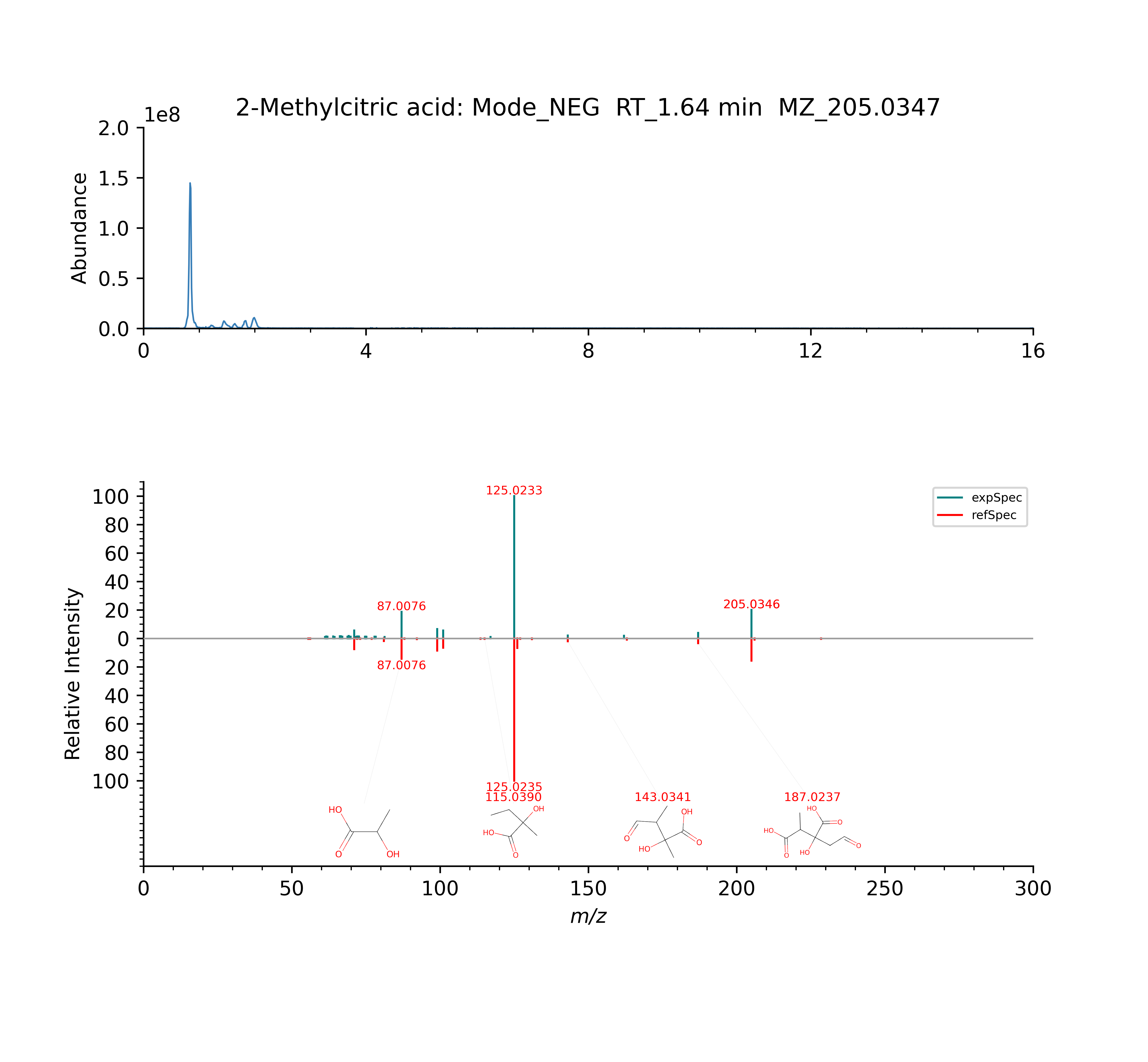

Supplement: Supplementary file 1 [file ijms-27-02203-s001.zip › ijms-4070482 Supplementary/Metabolite List Identified by LC-MS_MS from Rhodiola Species/136.png]

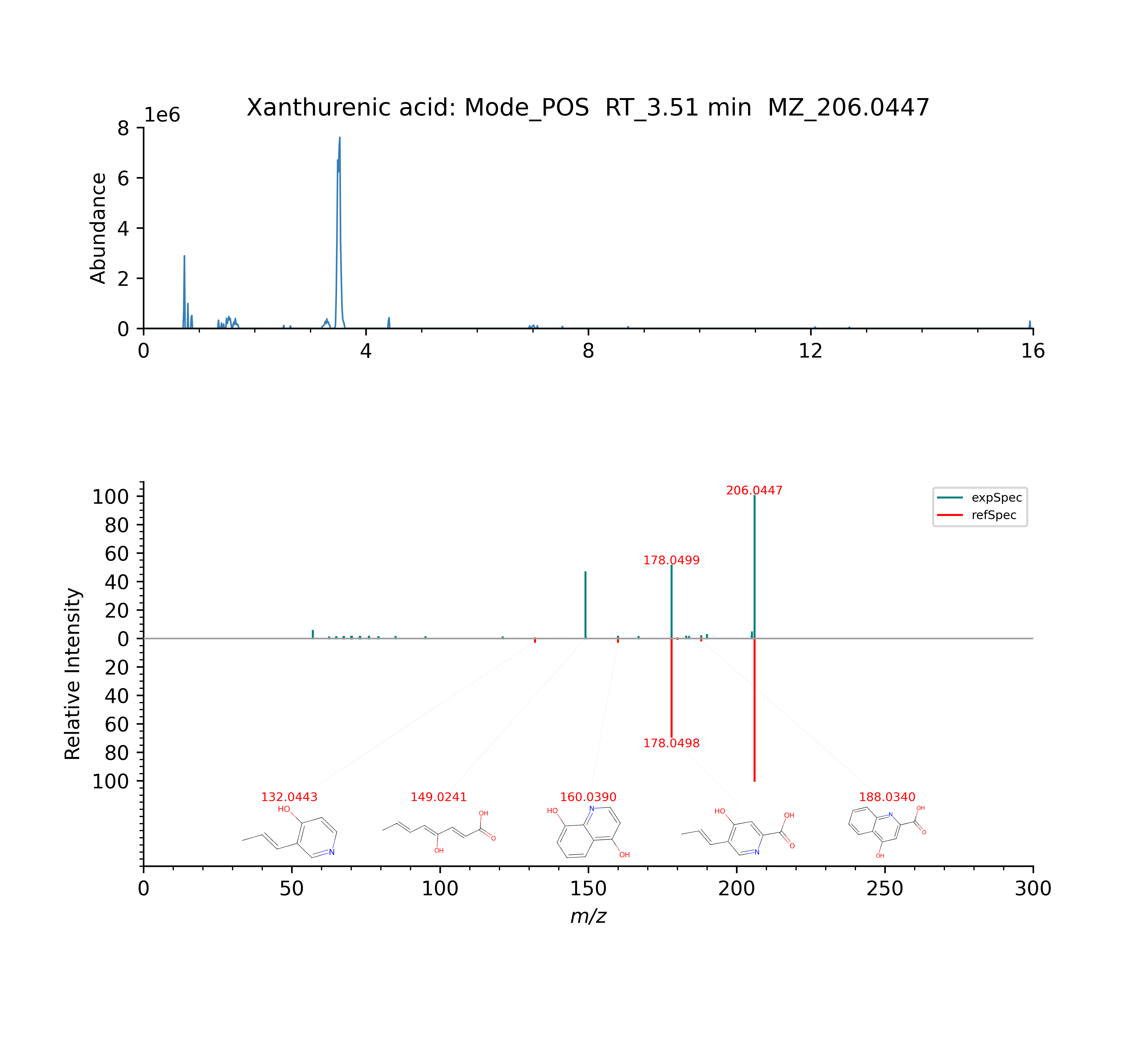

Supplement: Supplementary file 1 [file ijms-27-02203-s001.zip › ijms-4070482 Supplementary/Metabolite List Identified by LC-MS_MS from Rhodiola Species/137.png]

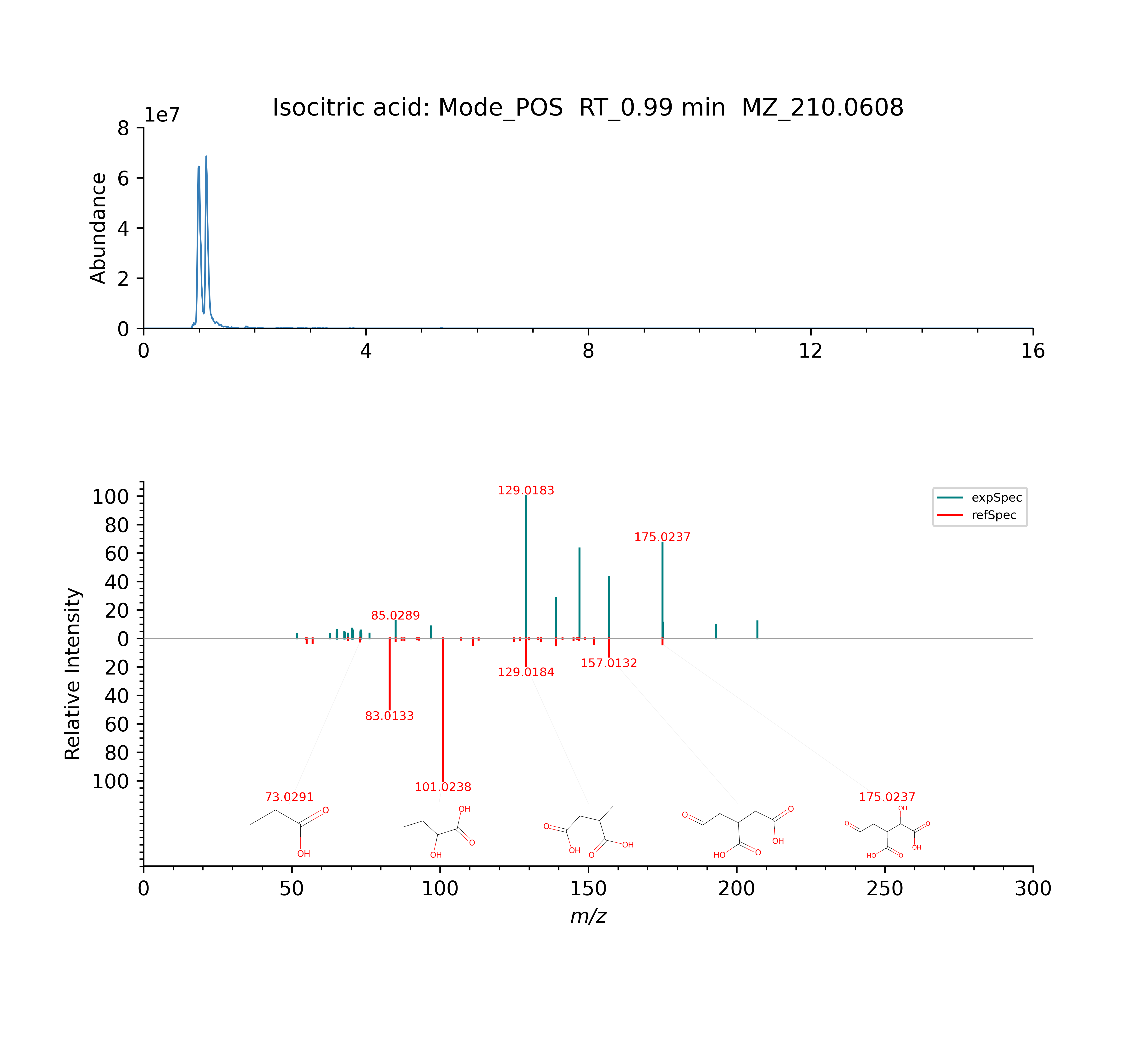

Supplement: Supplementary file 1 [file ijms-27-02203-s001.zip › ijms-4070482 Supplementary/Metabolite List Identified by LC-MS_MS from Rhodiola Species/138.png]

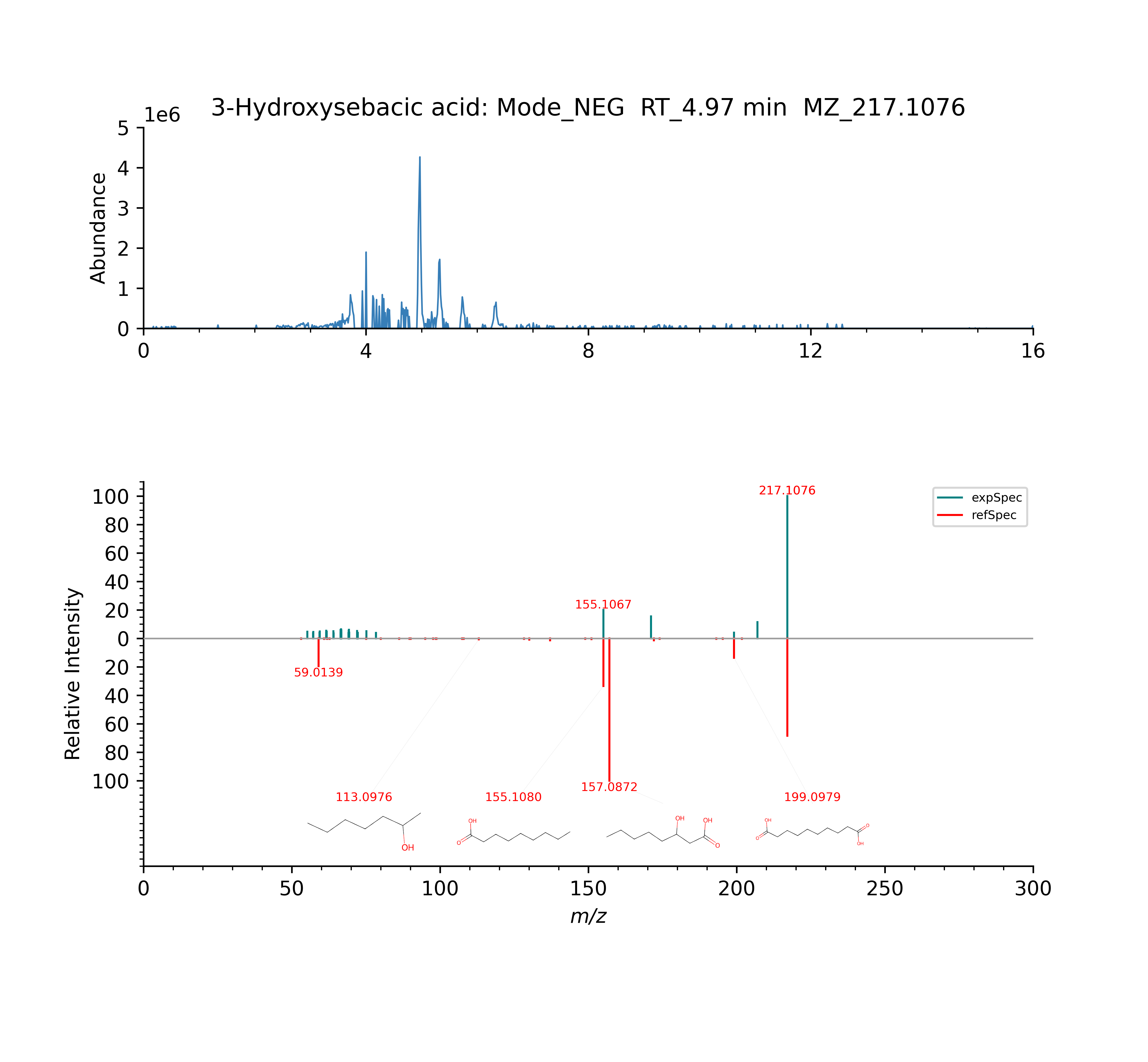

Supplement: Supplementary file 1 [file ijms-27-02203-s001.zip › ijms-4070482 Supplementary/Metabolite List Identified by LC-MS_MS from Rhodiola Species/139.png]

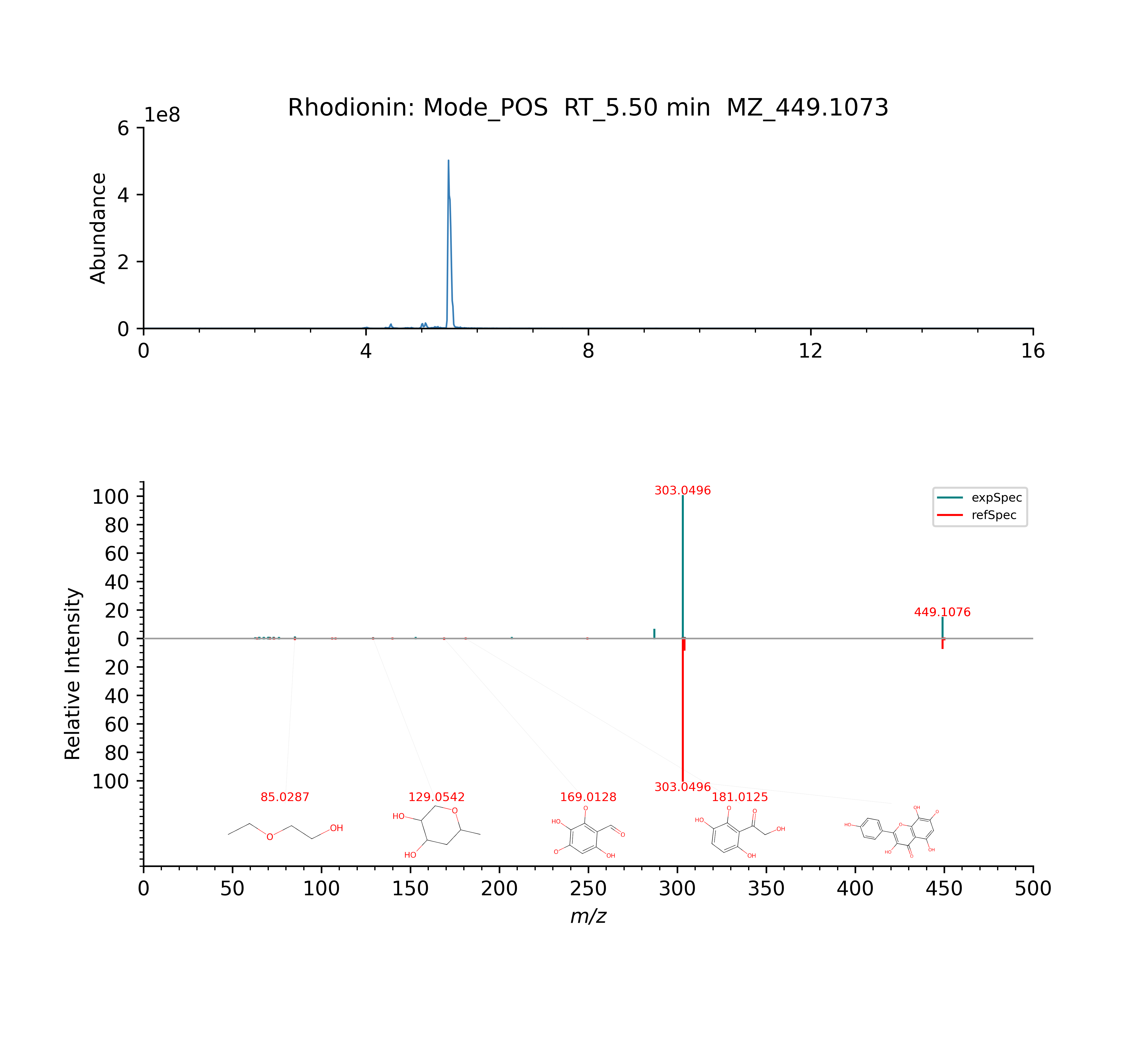

Supplement: Supplementary file 1 [file ijms-27-02203-s001.zip › ijms-4070482 Supplementary/Metabolite List Identified by LC-MS_MS from Rhodiola Species/14.png]

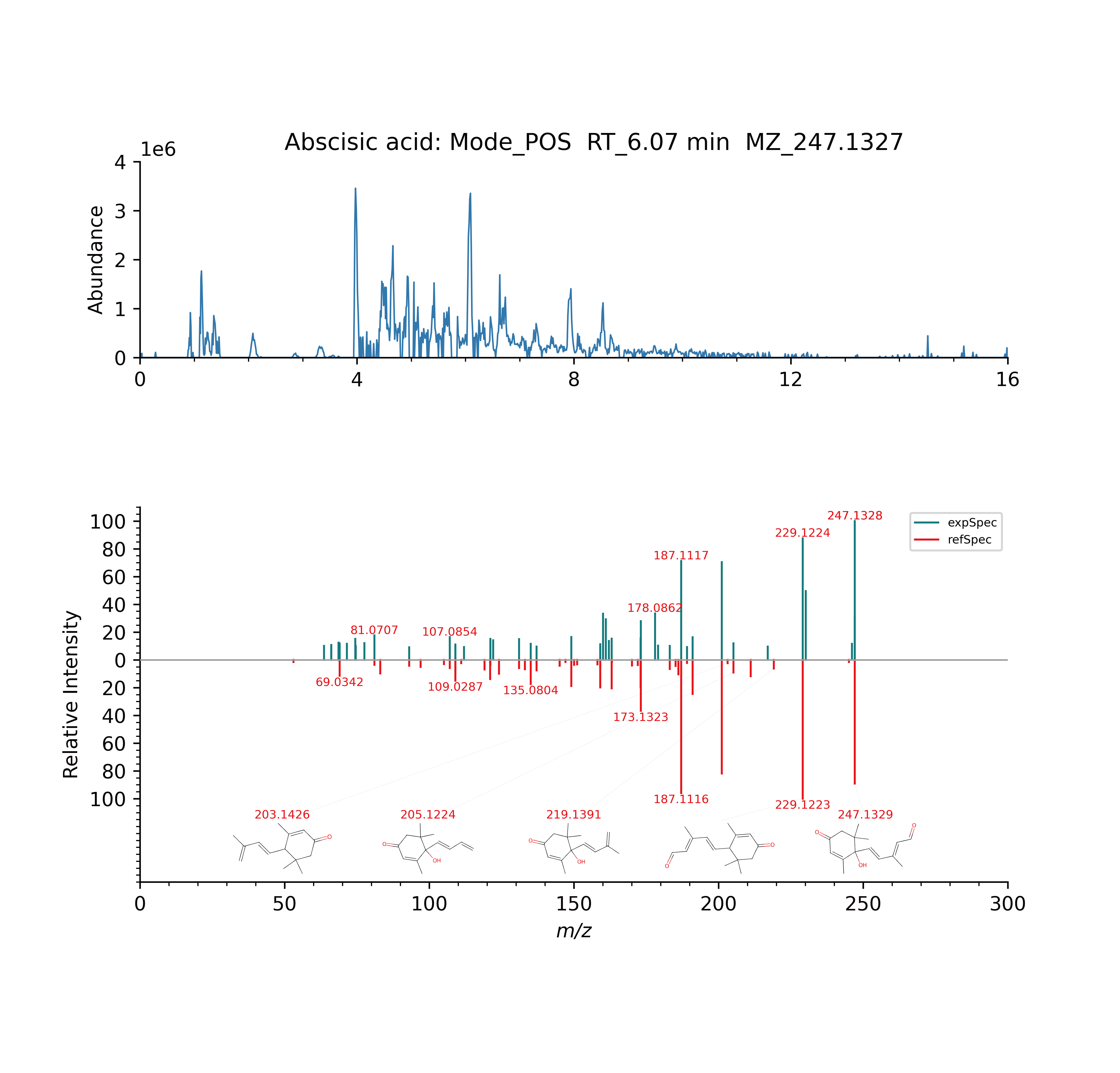

Supplement: Supplementary file 1 [file ijms-27-02203-s001.zip › ijms-4070482 Supplementary/Metabolite List Identified by LC-MS_MS from Rhodiola Species/140.png]

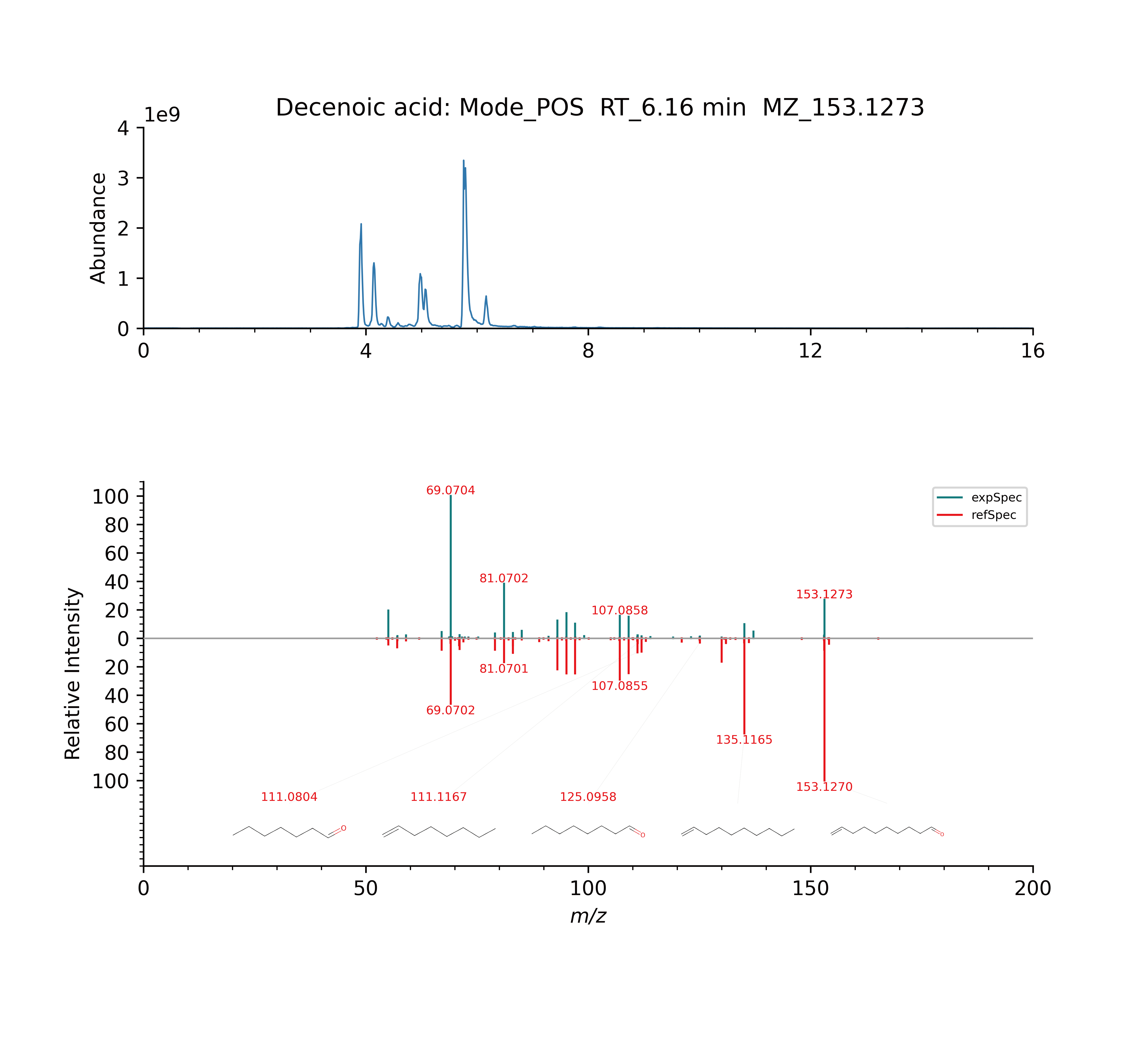

Supplement: Supplementary file 1 [file ijms-27-02203-s001.zip › ijms-4070482 Supplementary/Metabolite List Identified by LC-MS_MS from Rhodiola Species/141.png]

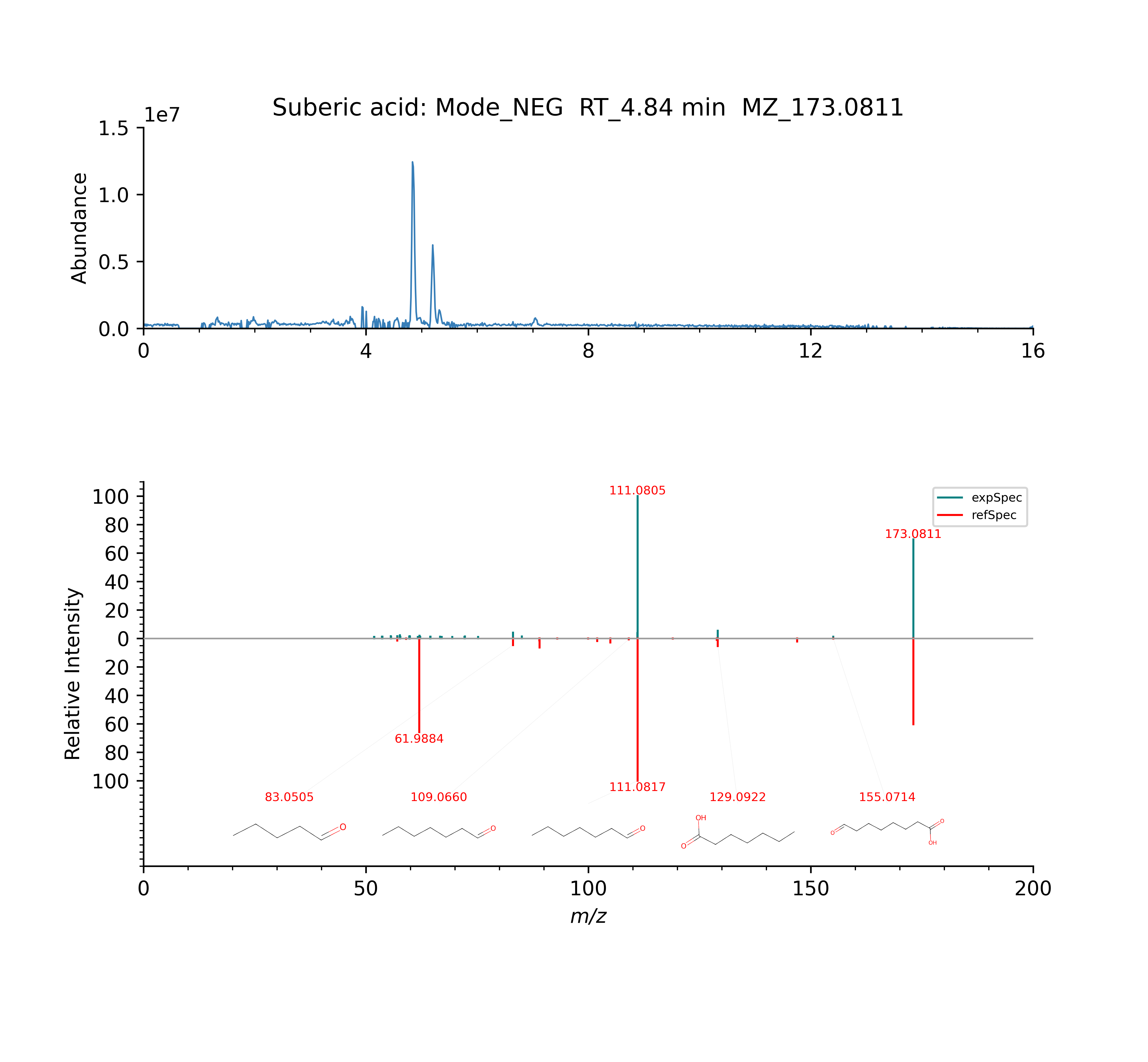

Supplement: Supplementary file 1 [file ijms-27-02203-s001.zip › ijms-4070482 Supplementary/Metabolite List Identified by LC-MS_MS from Rhodiola Species/142.png]

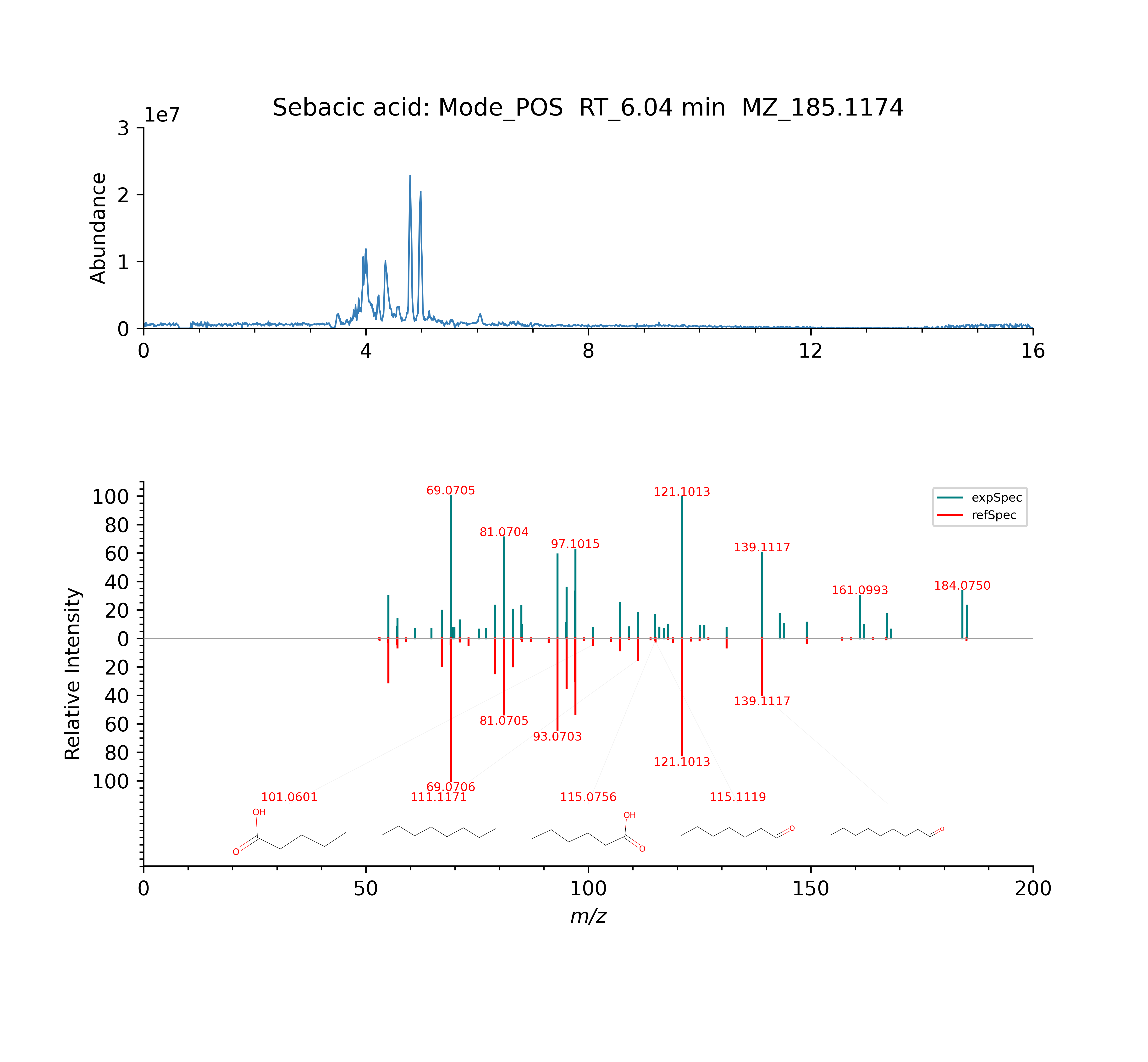

Supplement: Supplementary file 1 [file ijms-27-02203-s001.zip › ijms-4070482 Supplementary/Metabolite List Identified by LC-MS_MS from Rhodiola Species/143.png]

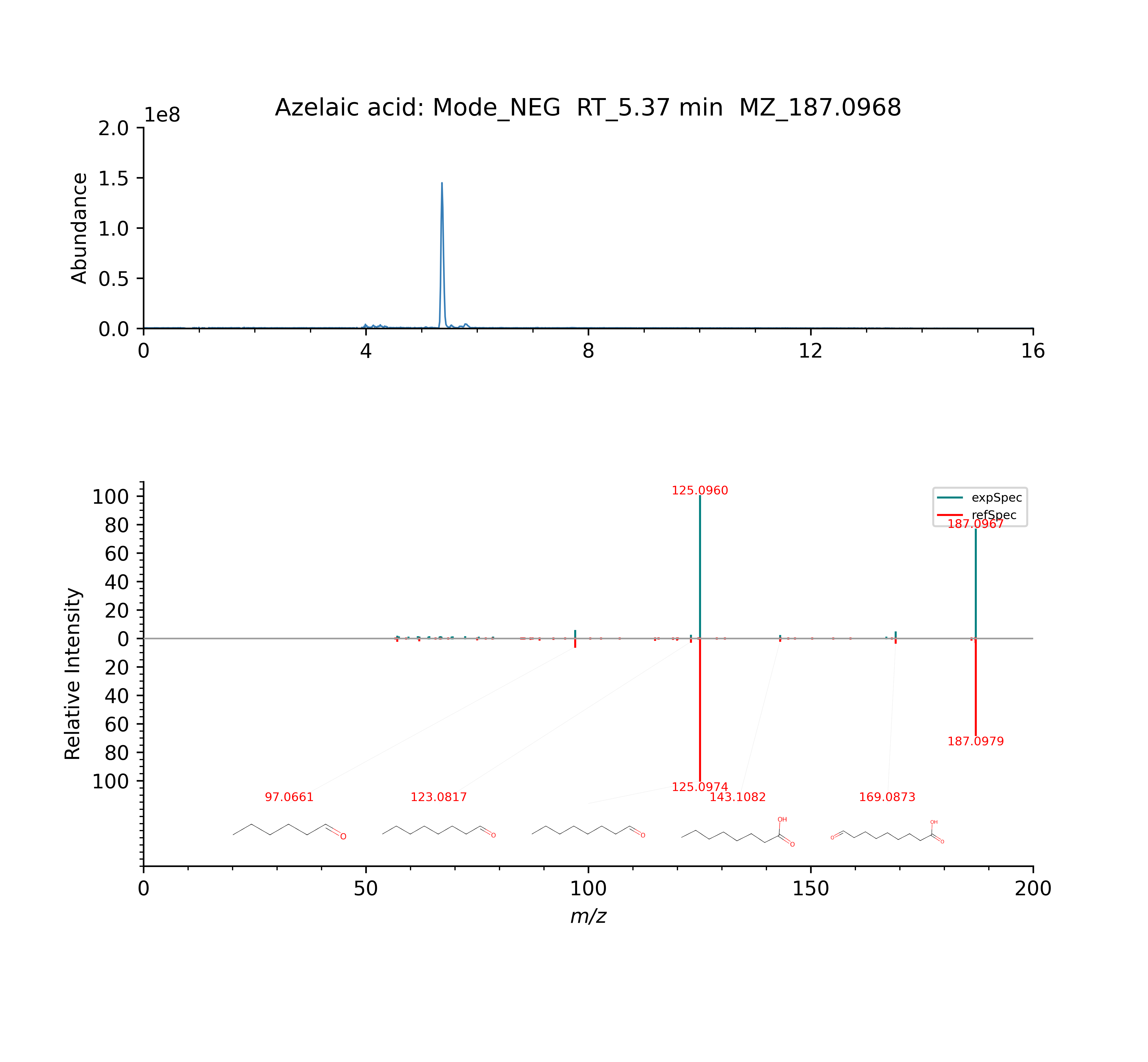

Supplement: Supplementary file 1 [file ijms-27-02203-s001.zip › ijms-4070482 Supplementary/Metabolite List Identified by LC-MS_MS from Rhodiola Species/144.png]

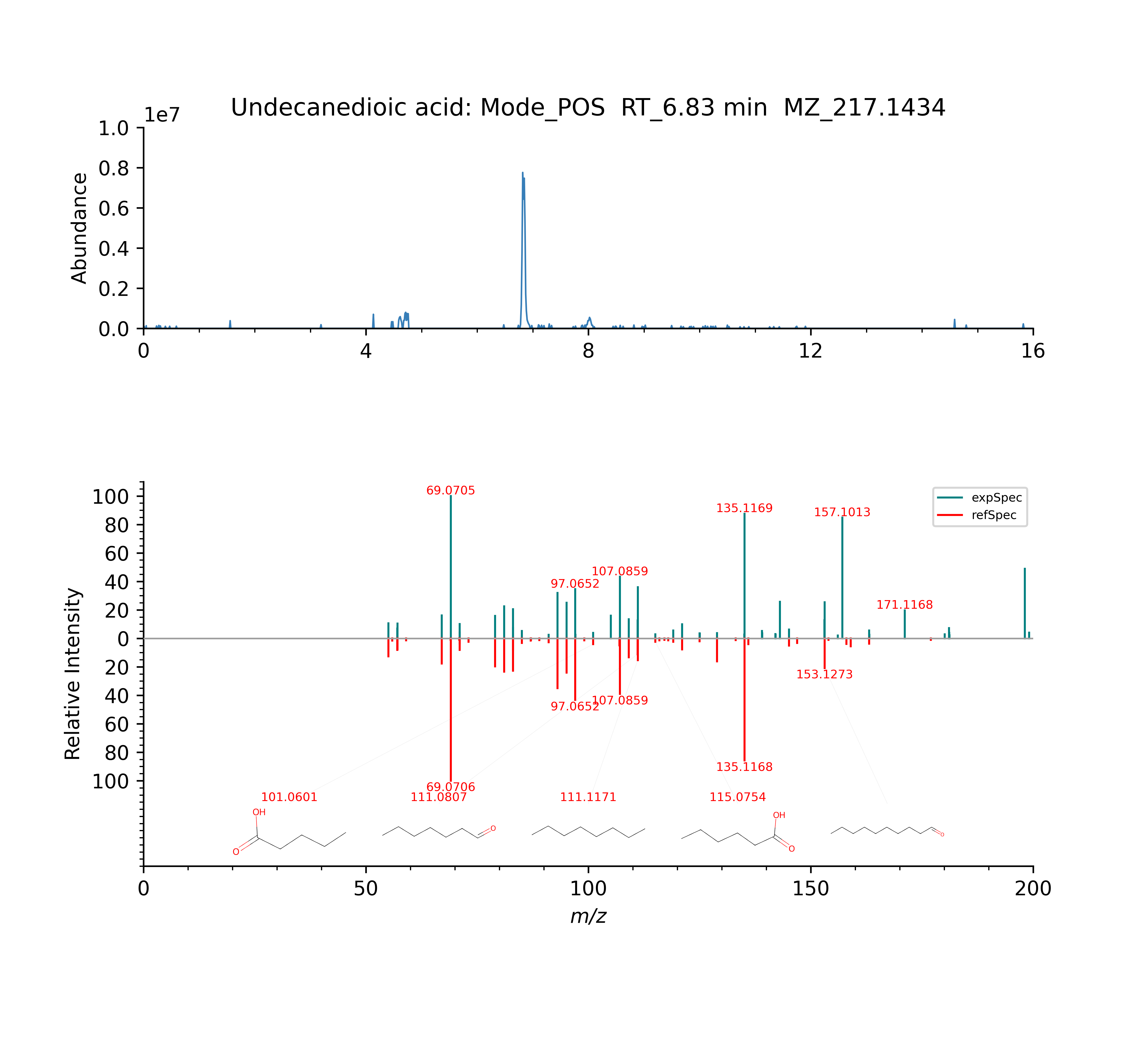

Supplement: Supplementary file 1 [file ijms-27-02203-s001.zip › ijms-4070482 Supplementary/Metabolite List Identified by LC-MS_MS from Rhodiola Species/145.png]

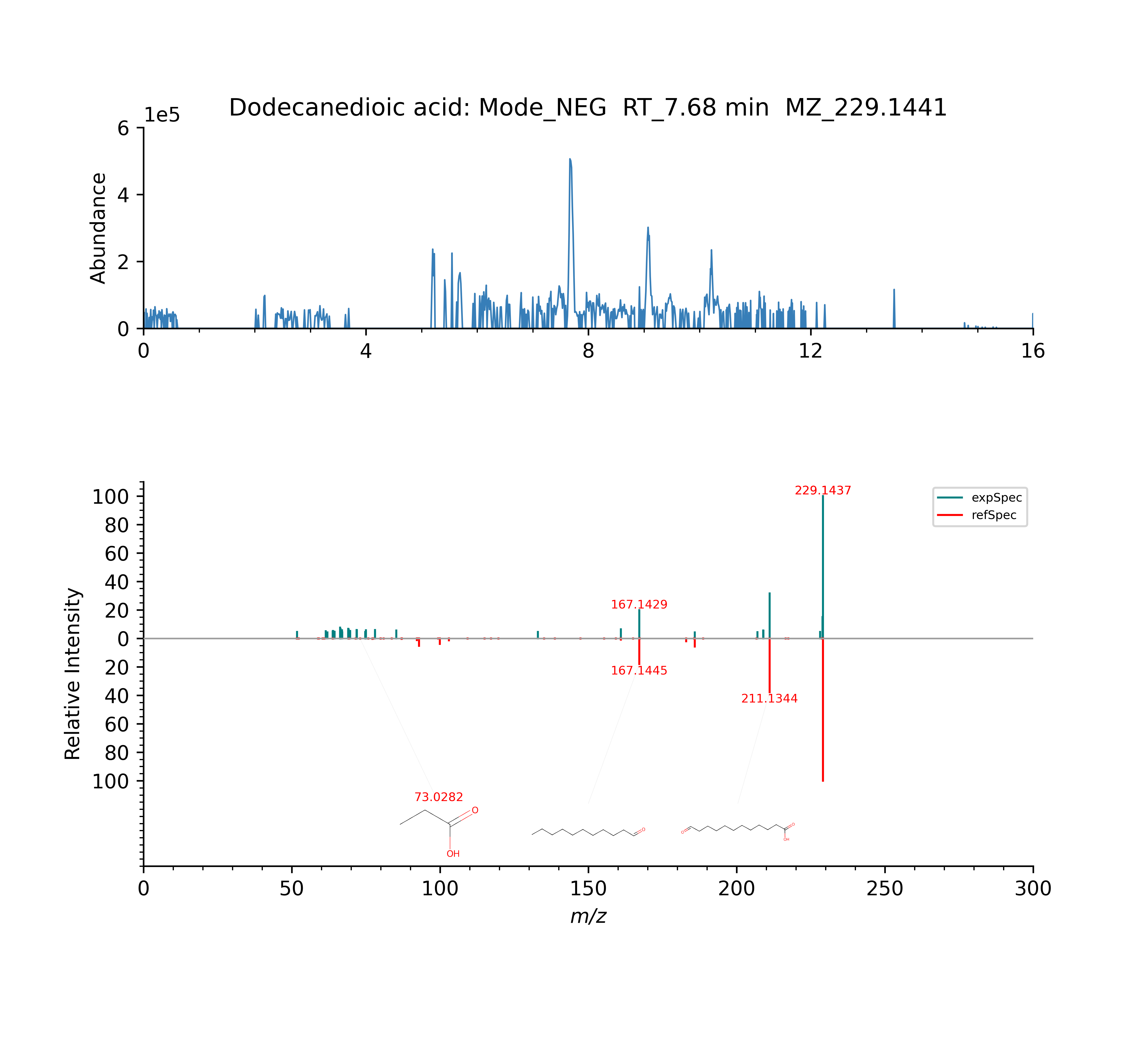

Supplement: Supplementary file 1 [file ijms-27-02203-s001.zip › ijms-4070482 Supplementary/Metabolite List Identified by LC-MS_MS from Rhodiola Species/146.png]

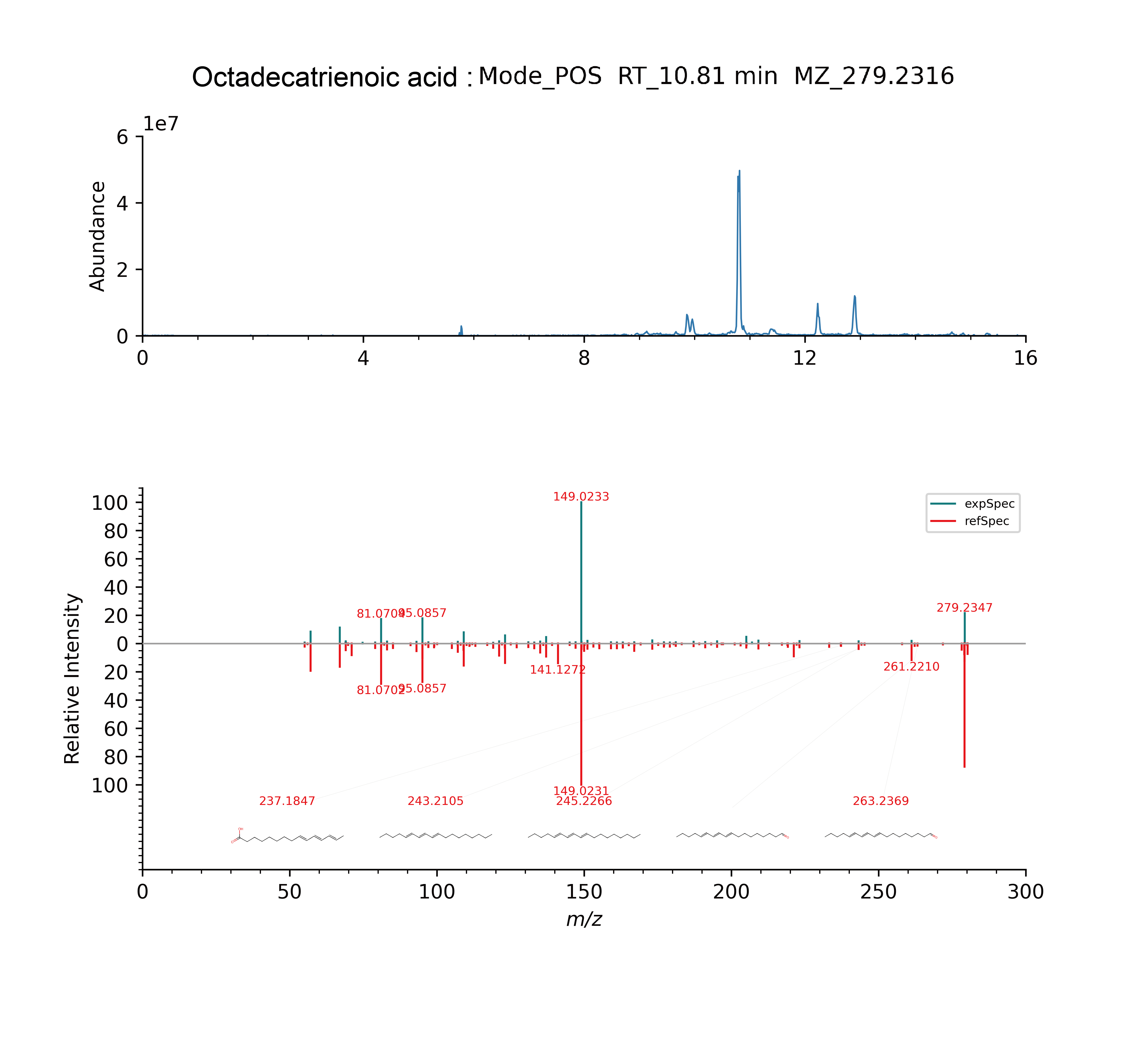

Supplement: Supplementary file 1 [file ijms-27-02203-s001.zip › ijms-4070482 Supplementary/Metabolite List Identified by LC-MS_MS from Rhodiola Species/147.png]

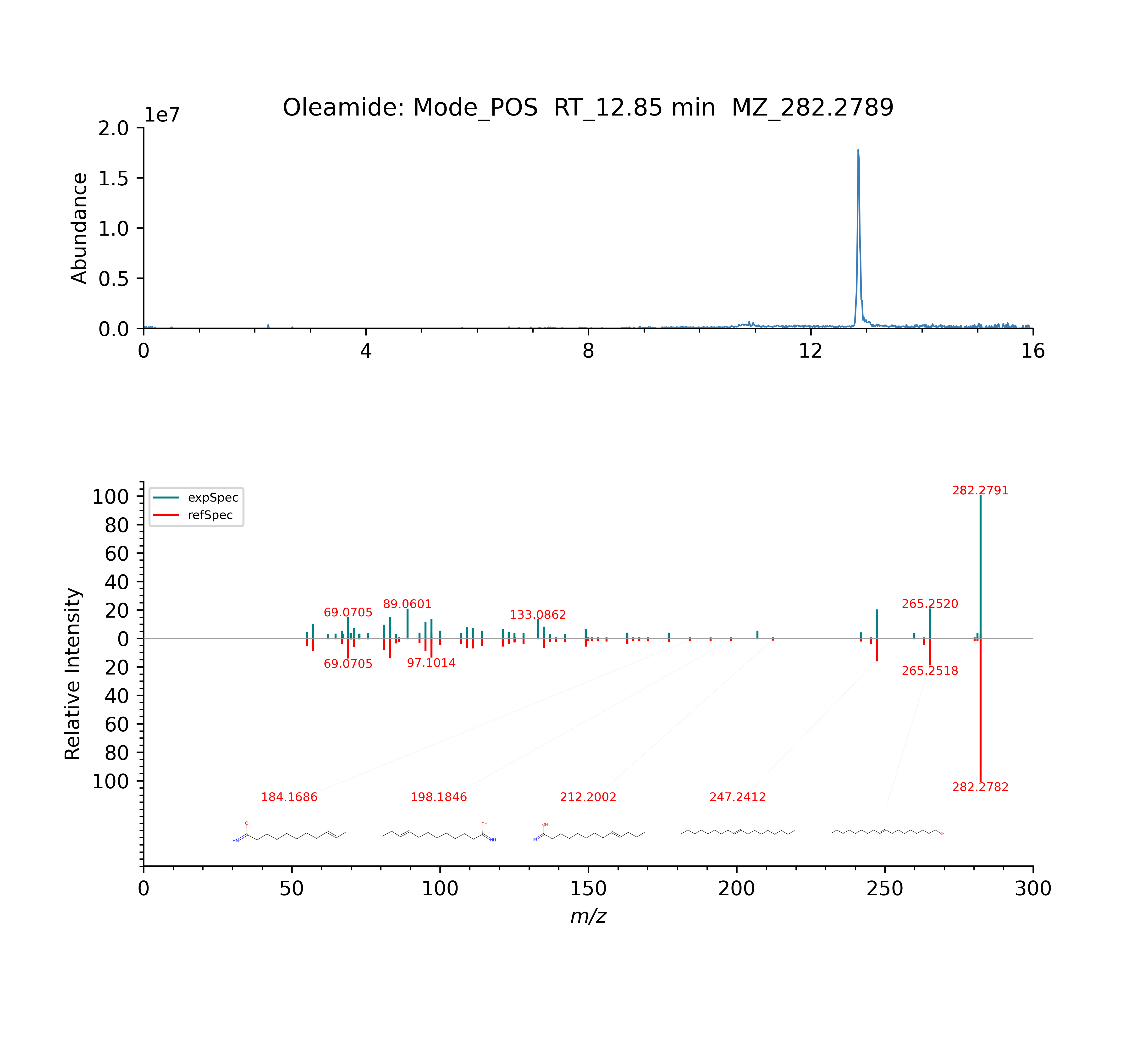

Supplement: Supplementary file 1 [file ijms-27-02203-s001.zip › ijms-4070482 Supplementary/Metabolite List Identified by LC-MS_MS from Rhodiola Species/148.png]

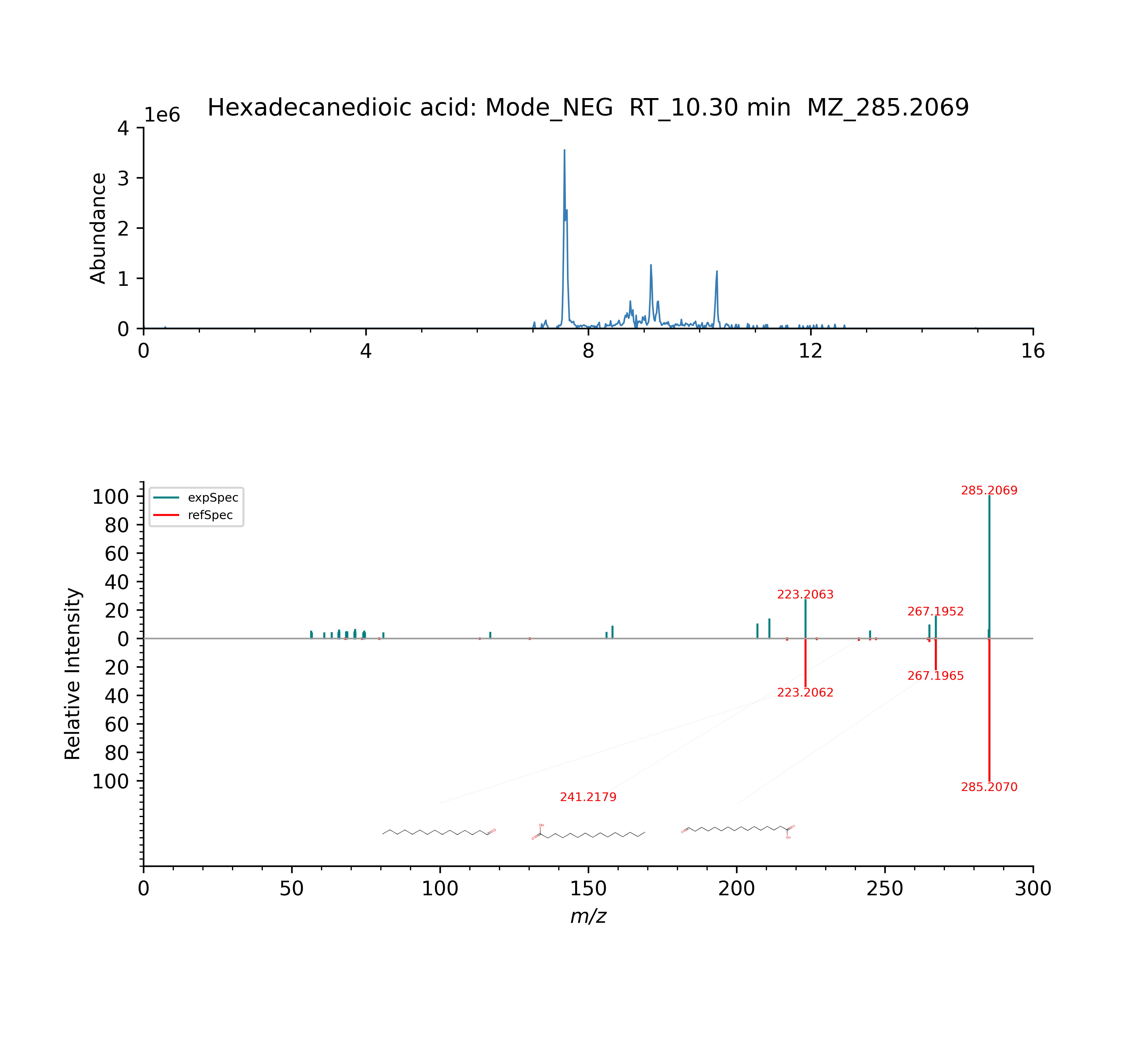

Supplement: Supplementary file 1 [file ijms-27-02203-s001.zip › ijms-4070482 Supplementary/Metabolite List Identified by LC-MS_MS from Rhodiola Species/149.png]

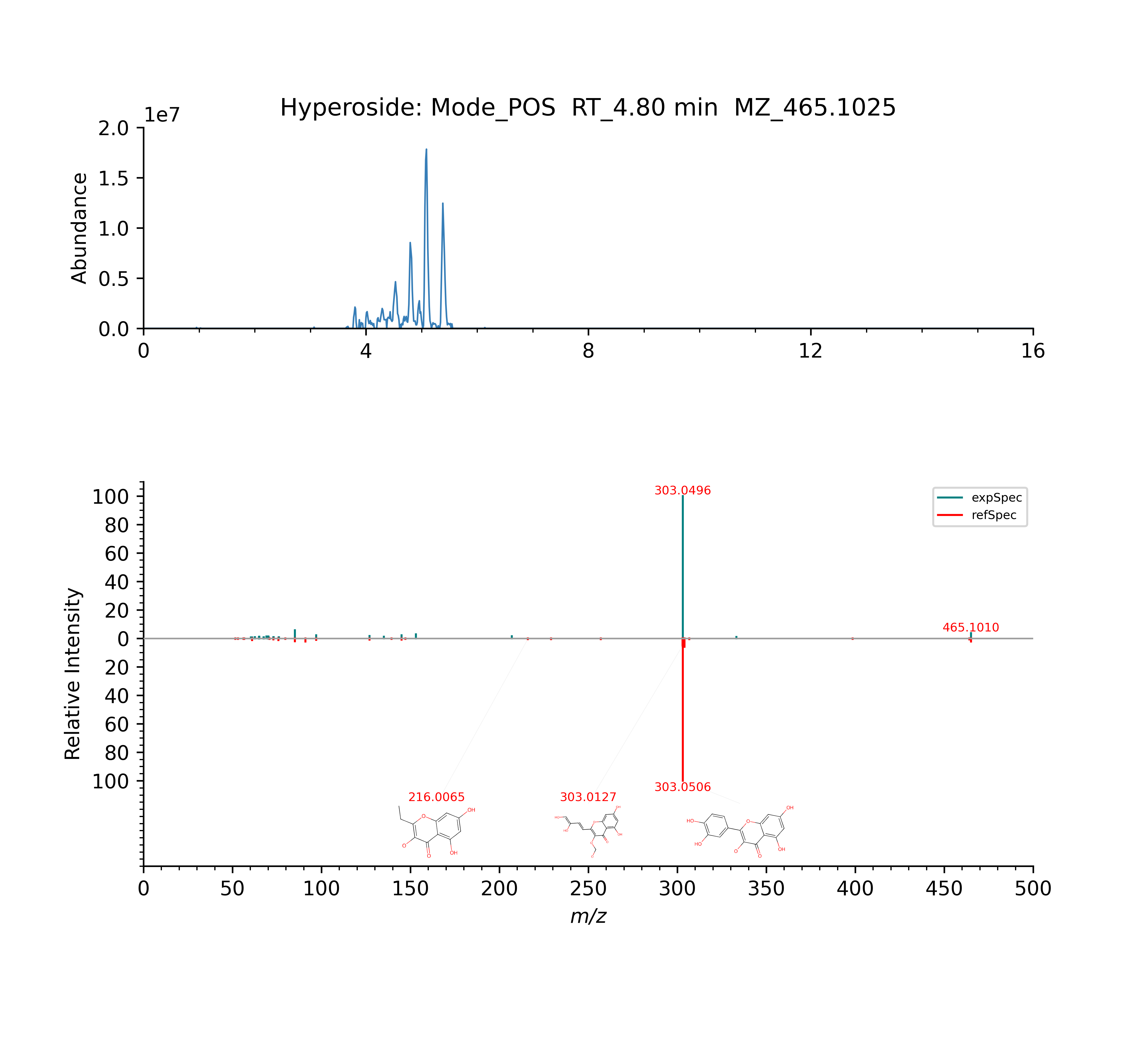

Supplement: Supplementary file 1 [file ijms-27-02203-s001.zip › ijms-4070482 Supplementary/Metabolite List Identified by LC-MS_MS from Rhodiola Species/15.png]

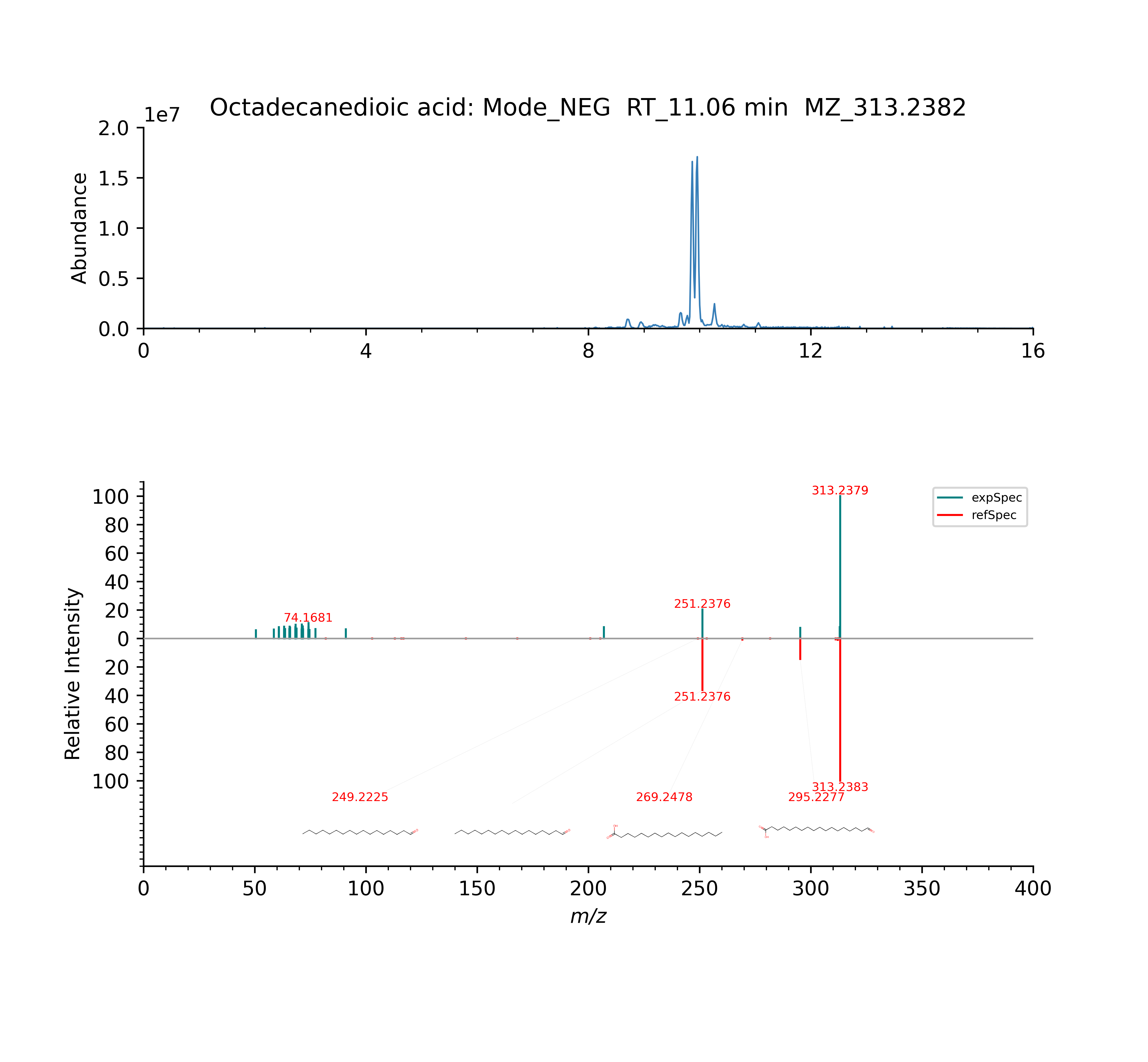

Supplement: Supplementary file 1 [file ijms-27-02203-s001.zip › ijms-4070482 Supplementary/Metabolite List Identified by LC-MS_MS from Rhodiola Species/150.png]

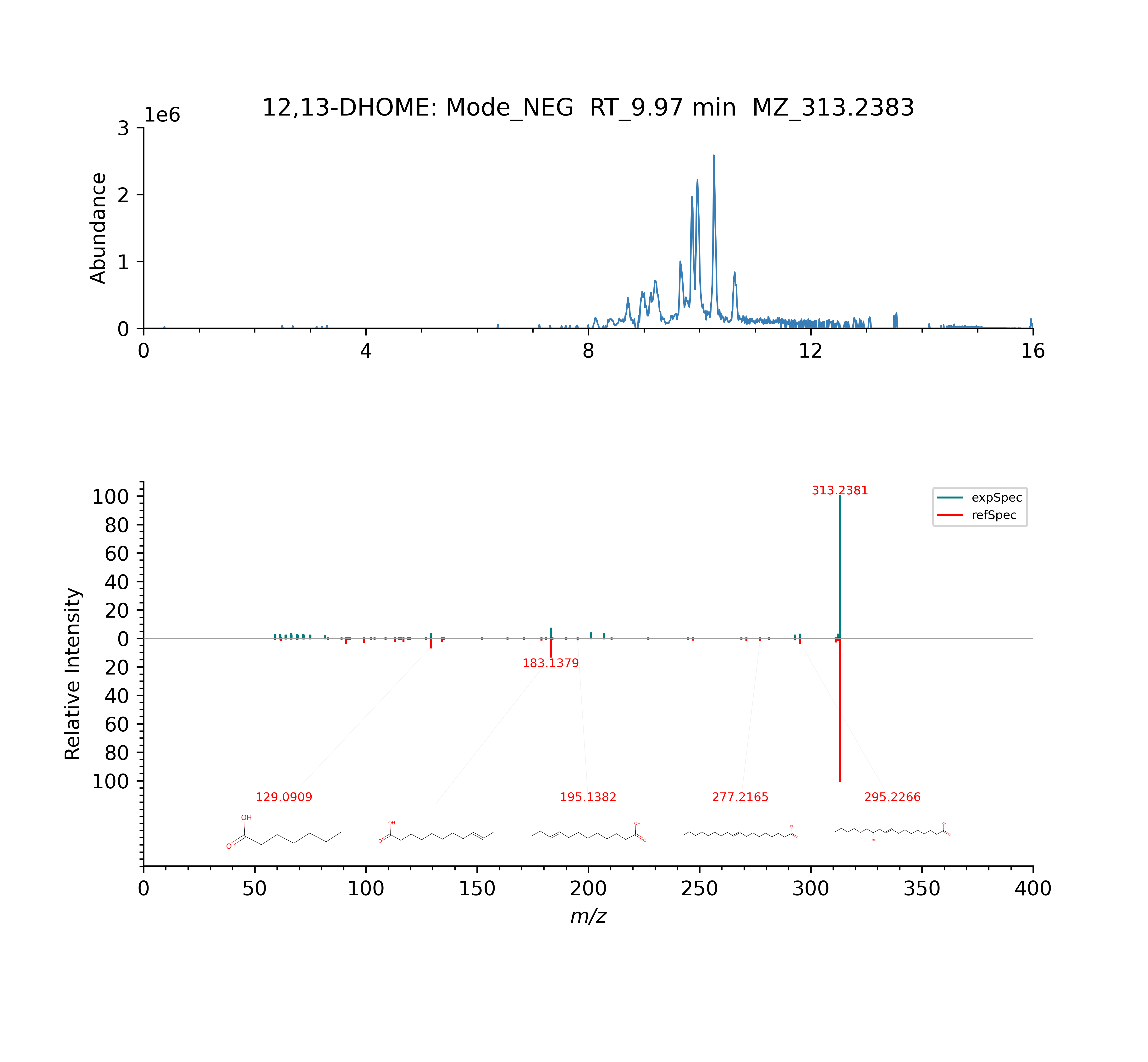

Supplement: Supplementary file 1 [file ijms-27-02203-s001.zip › ijms-4070482 Supplementary/Metabolite List Identified by LC-MS_MS from Rhodiola Species/151.png]

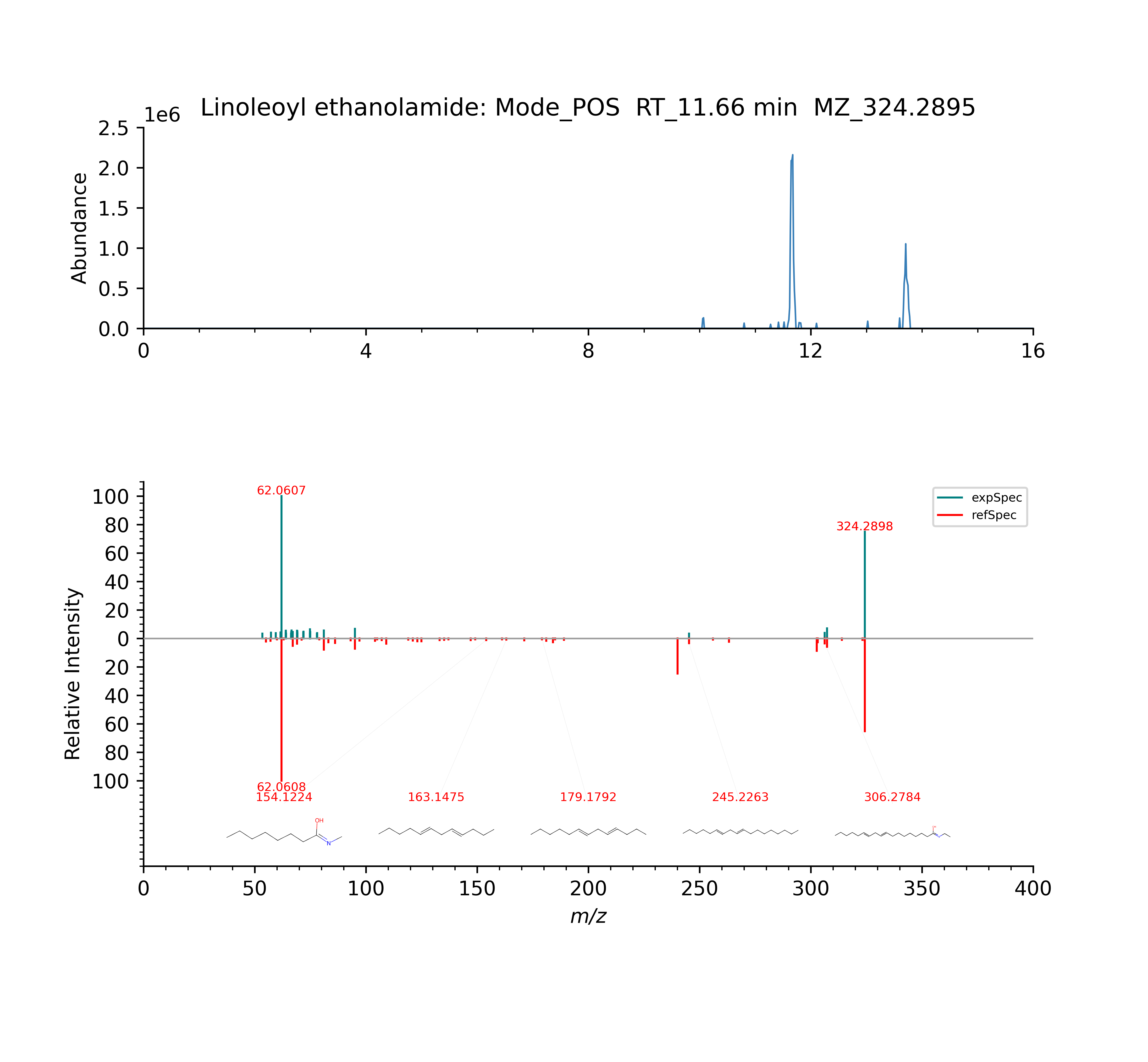

Supplement: Supplementary file 1 [file ijms-27-02203-s001.zip › ijms-4070482 Supplementary/Metabolite List Identified by LC-MS_MS from Rhodiola Species/152.png]

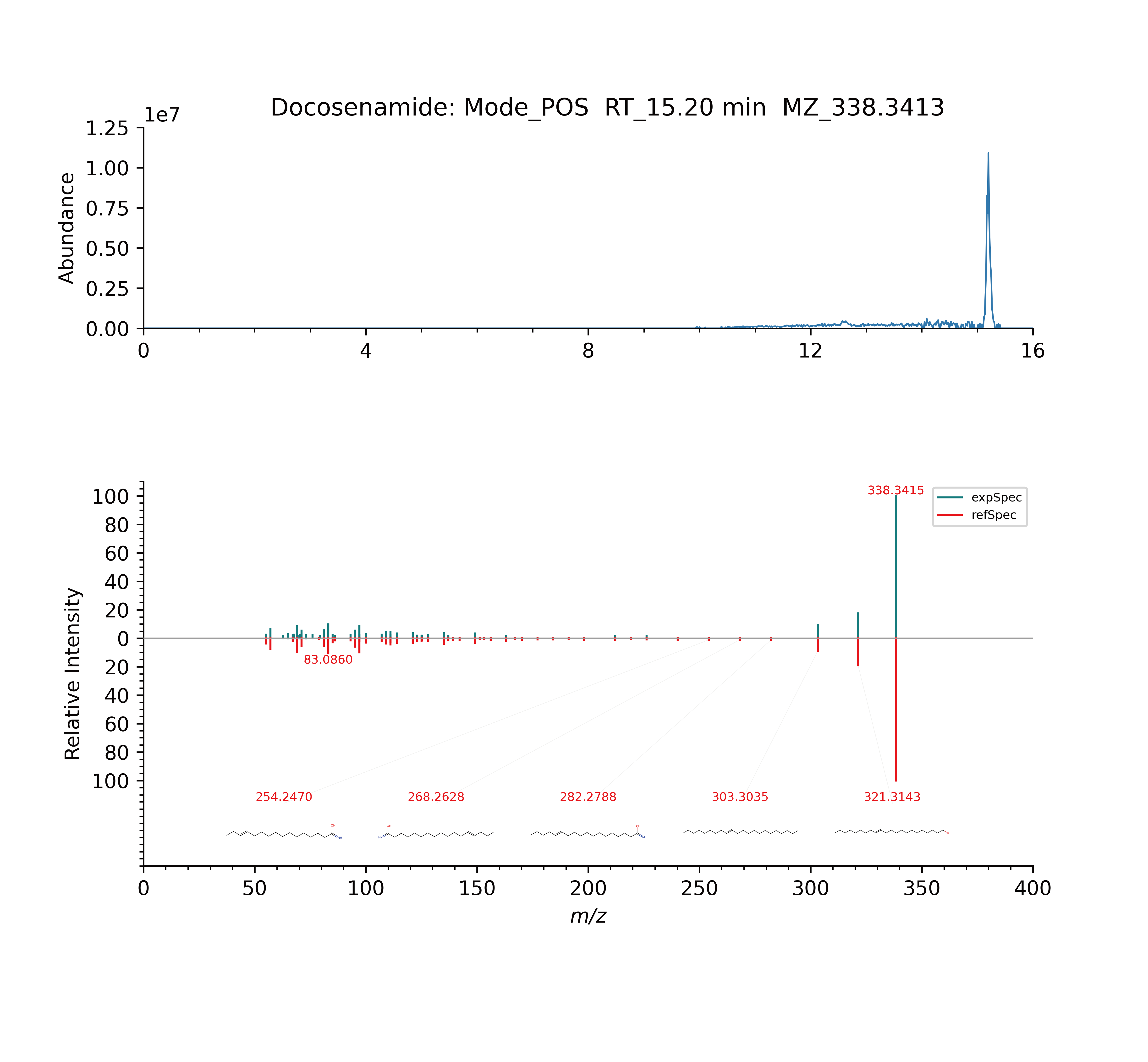

Supplement: Supplementary file 1 [file ijms-27-02203-s001.zip › ijms-4070482 Supplementary/Metabolite List Identified by LC-MS_MS from Rhodiola Species/153.png]

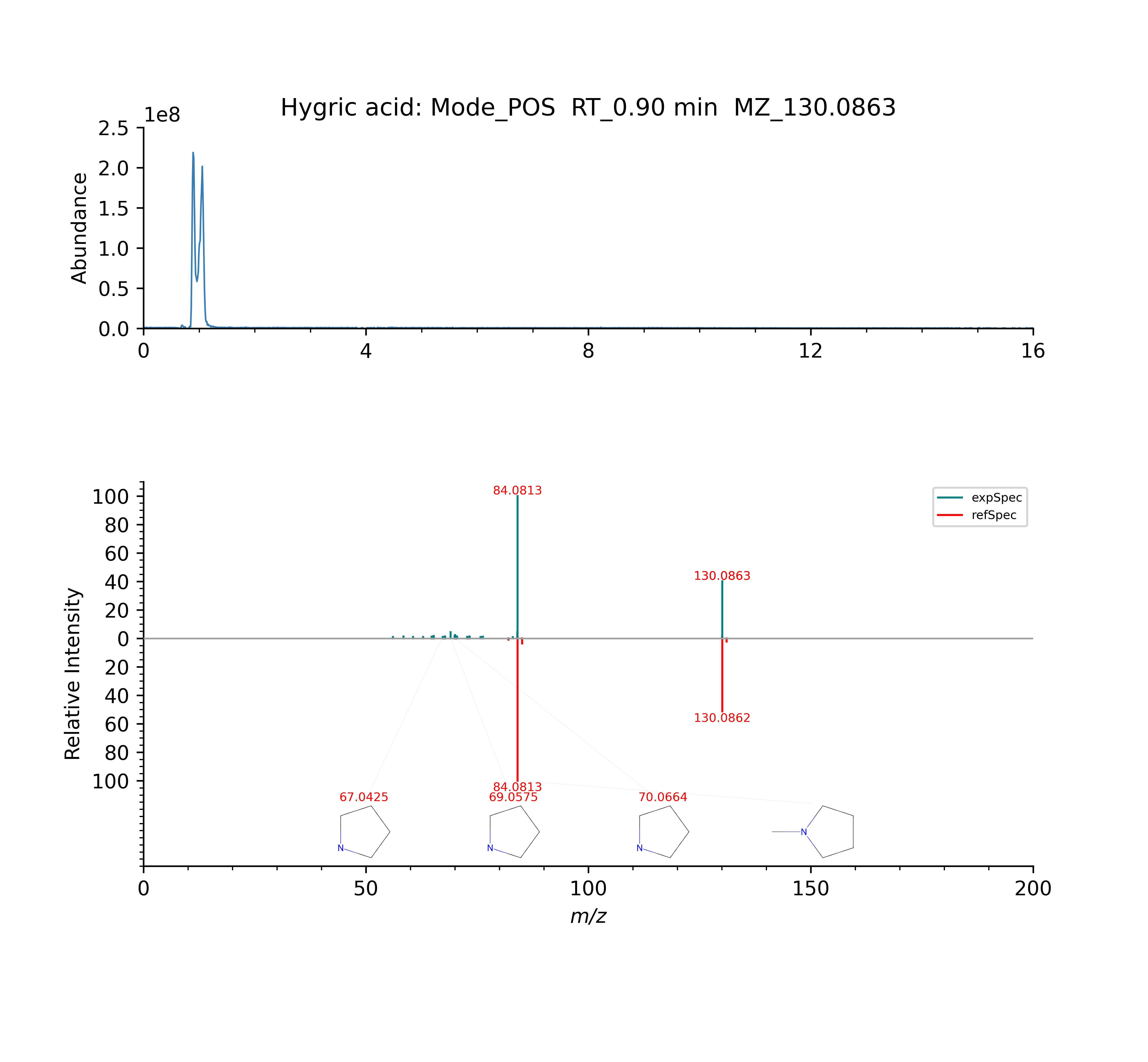

Supplement: Supplementary file 1 [file ijms-27-02203-s001.zip › ijms-4070482 Supplementary/Metabolite List Identified by LC-MS_MS from Rhodiola Species/154.png]

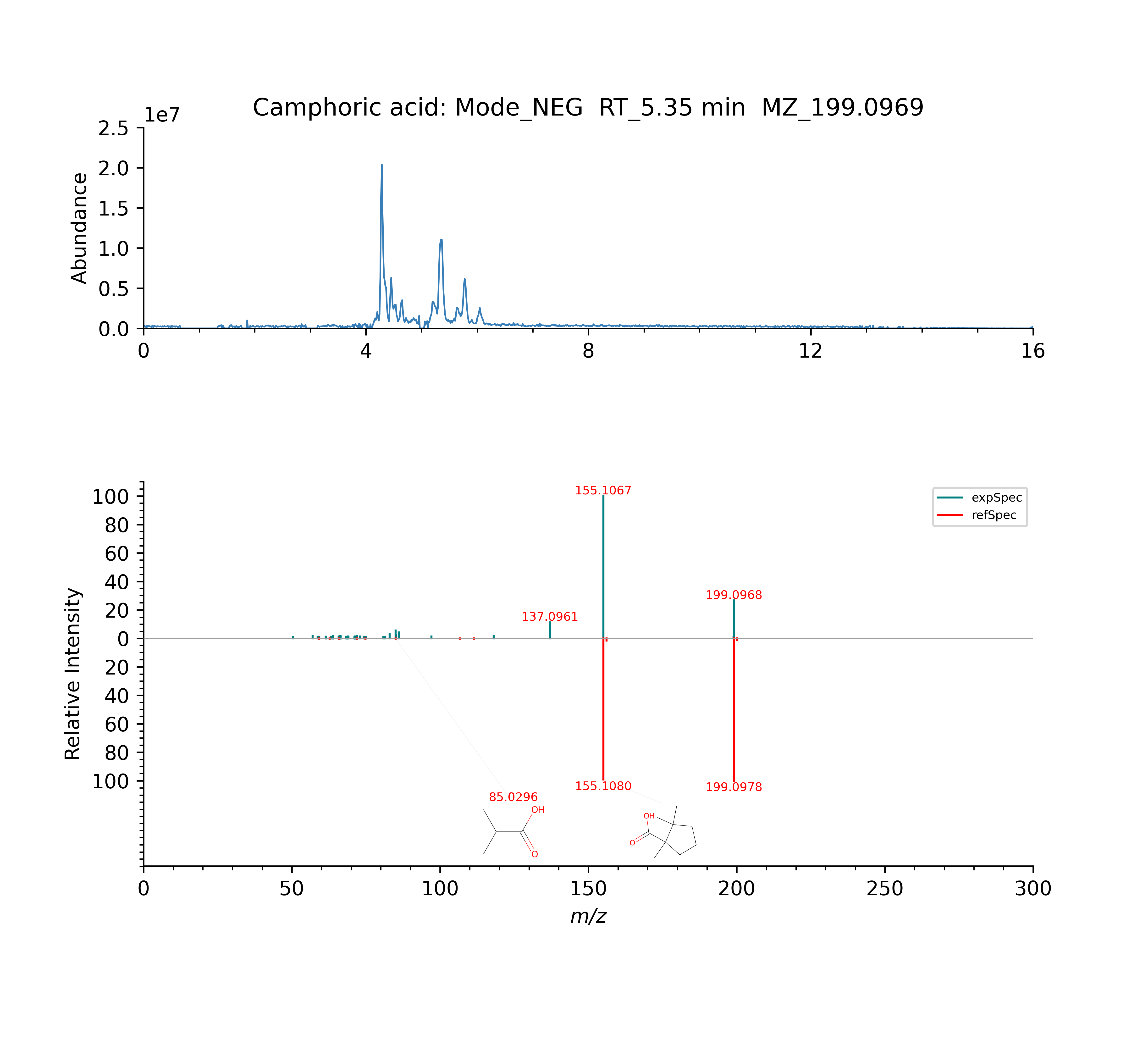

Supplement: Supplementary file 1 [file ijms-27-02203-s001.zip › ijms-4070482 Supplementary/Metabolite List Identified by LC-MS_MS from Rhodiola Species/155.png]

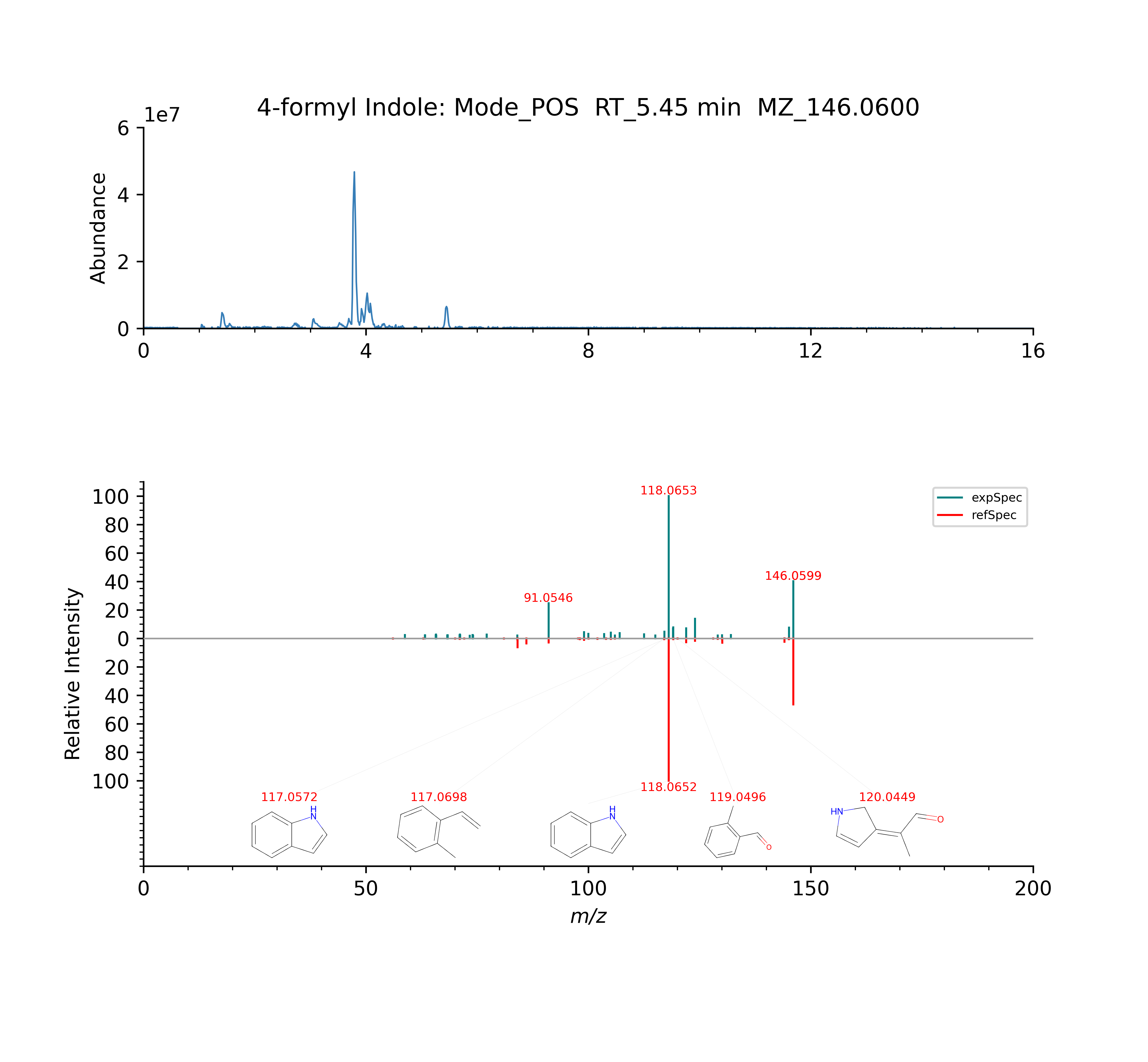

Supplement: Supplementary file 1 [file ijms-27-02203-s001.zip › ijms-4070482 Supplementary/Metabolite List Identified by LC-MS_MS from Rhodiola Species/156.png]

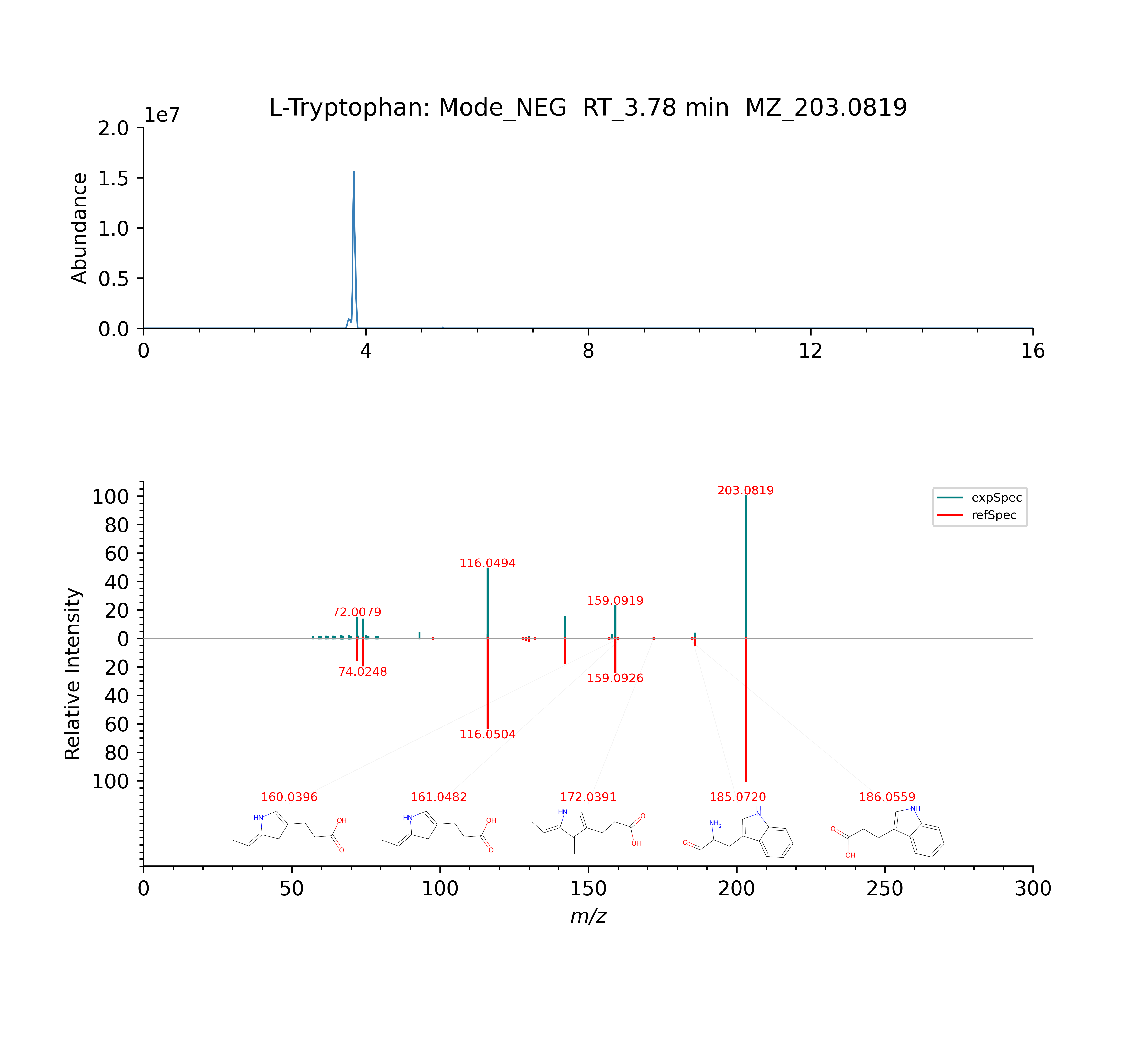

Supplement: Supplementary file 1 [file ijms-27-02203-s001.zip › ijms-4070482 Supplementary/Metabolite List Identified by LC-MS_MS from Rhodiola Species/157.png]

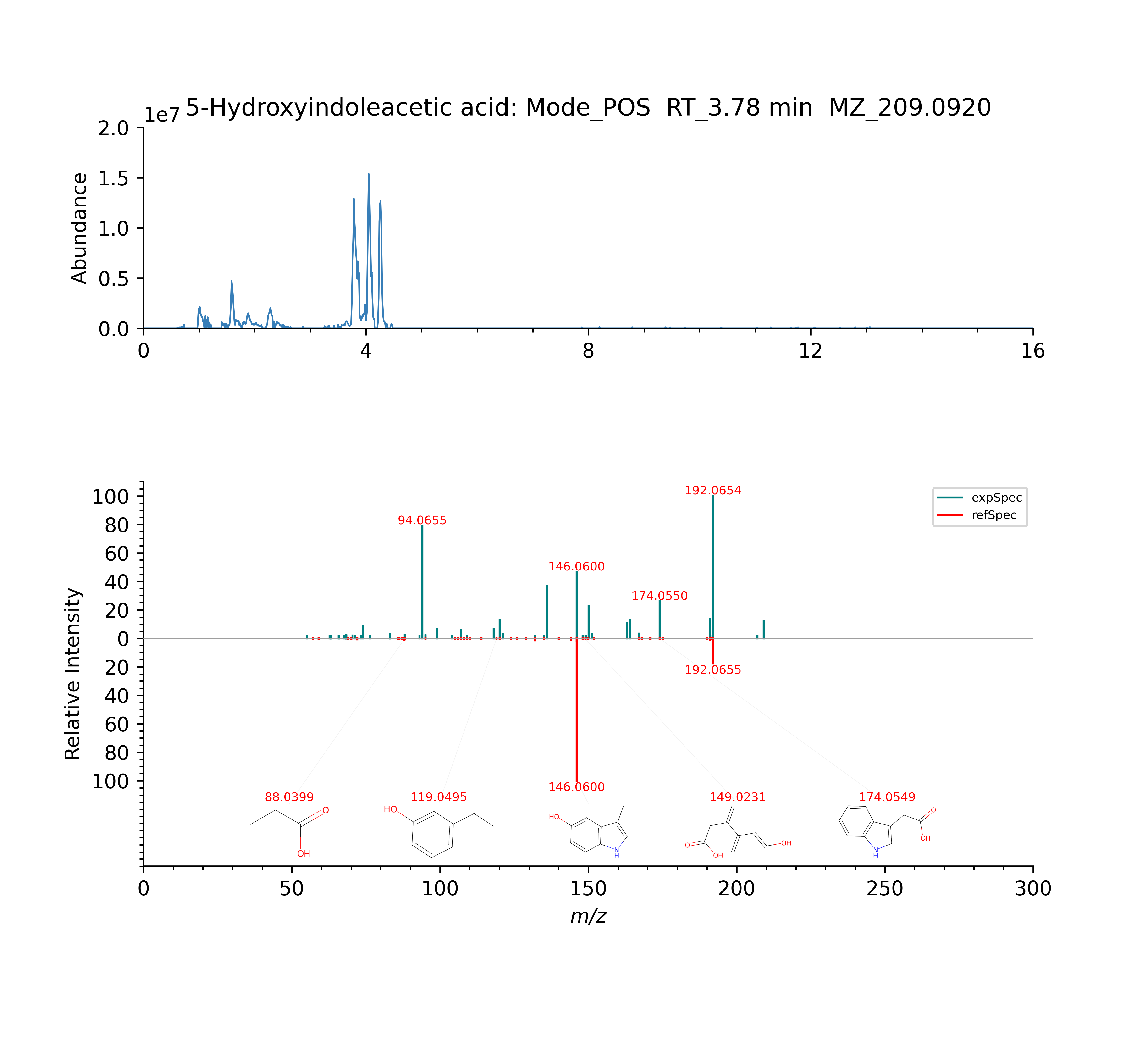

Supplement: Supplementary file 1 [file ijms-27-02203-s001.zip › ijms-4070482 Supplementary/Metabolite List Identified by LC-MS_MS from Rhodiola Species/158.png]

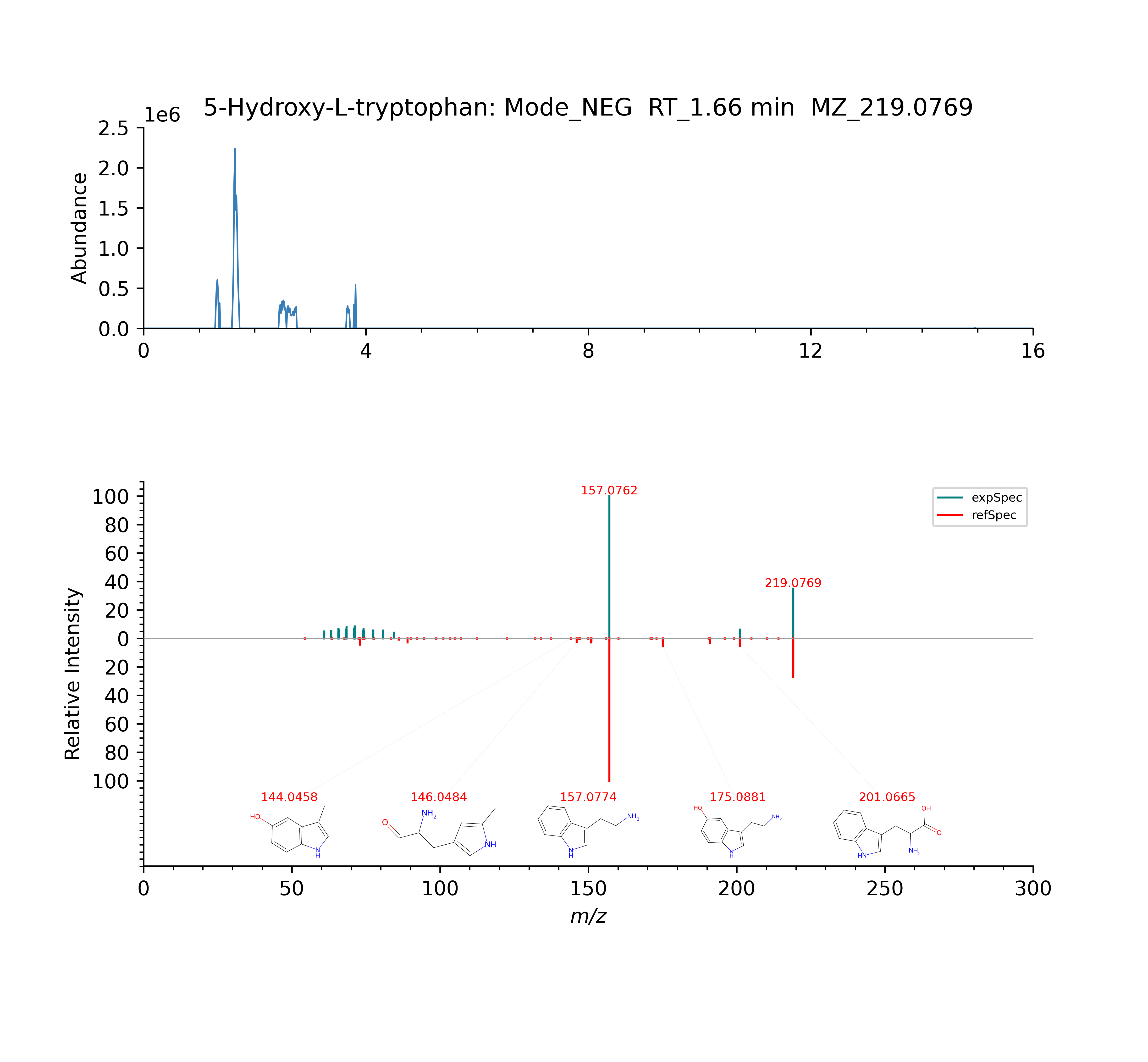

Supplement: Supplementary file 1 [file ijms-27-02203-s001.zip › ijms-4070482 Supplementary/Metabolite List Identified by LC-MS_MS from Rhodiola Species/159.png]

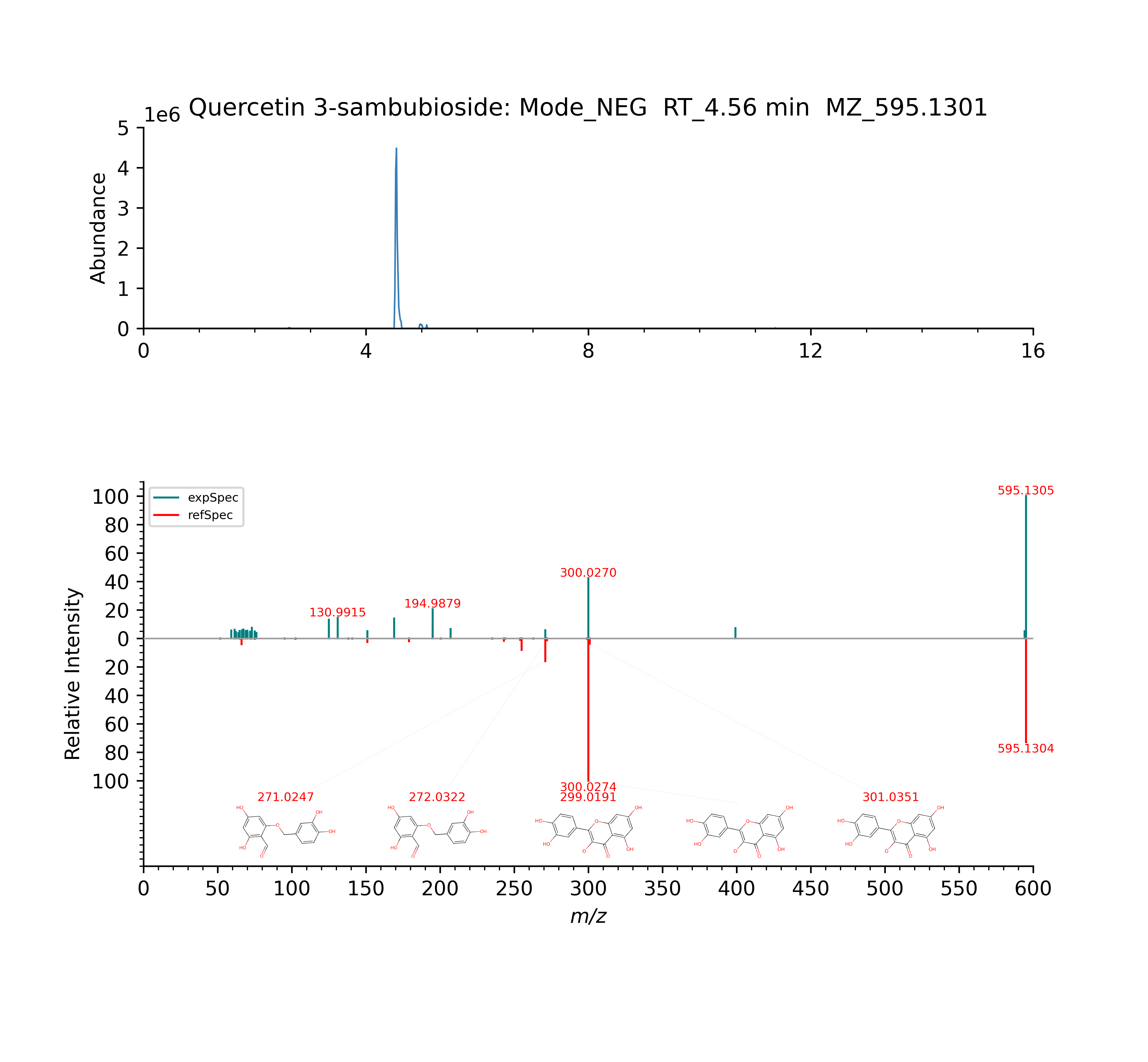

Supplement: Supplementary file 1 [file ijms-27-02203-s001.zip › ijms-4070482 Supplementary/Metabolite List Identified by LC-MS_MS from Rhodiola Species/16.png]

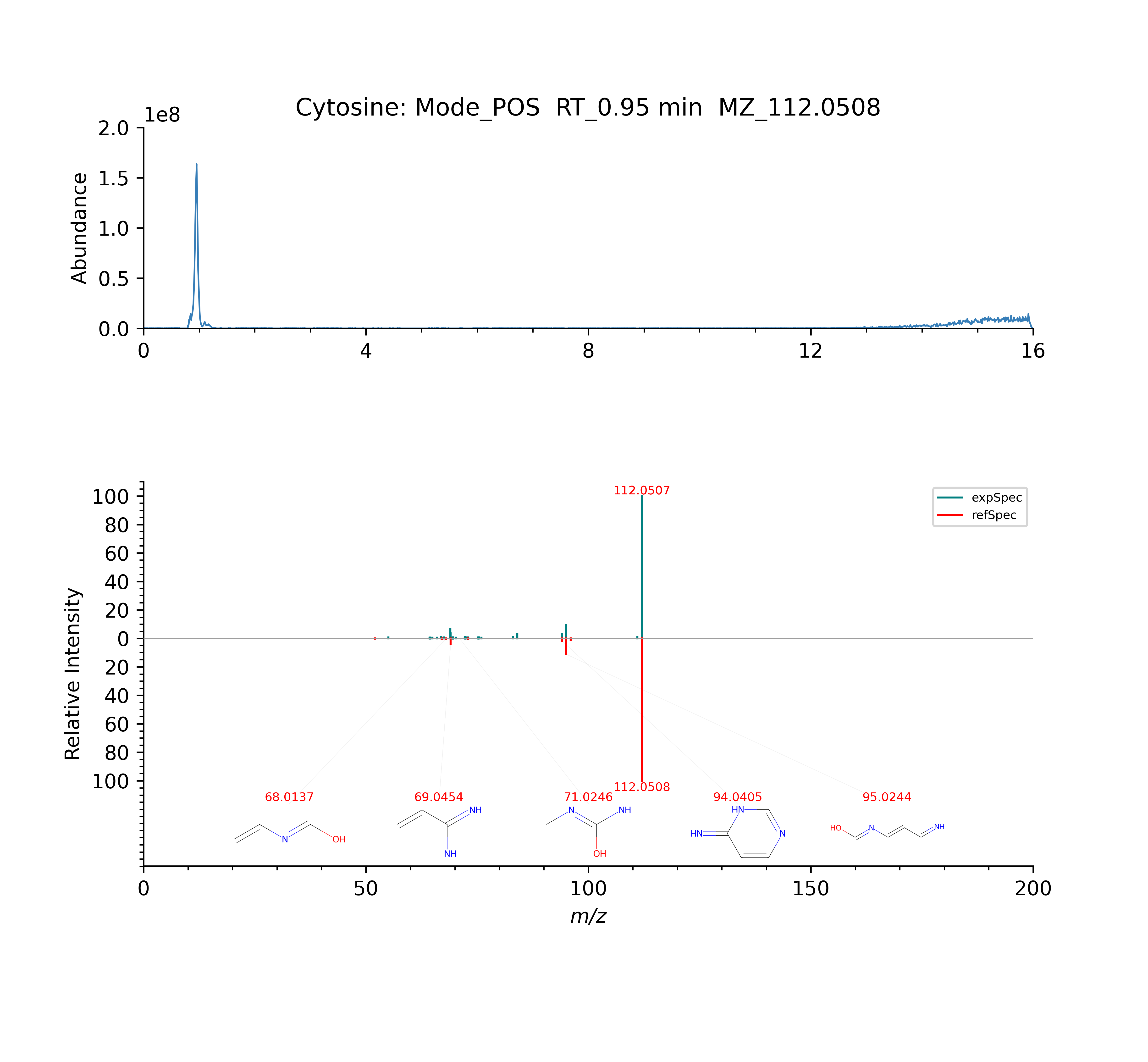

Supplement: Supplementary file 1 [file ijms-27-02203-s001.zip › ijms-4070482 Supplementary/Metabolite List Identified by LC-MS_MS from Rhodiola Species/160.png]

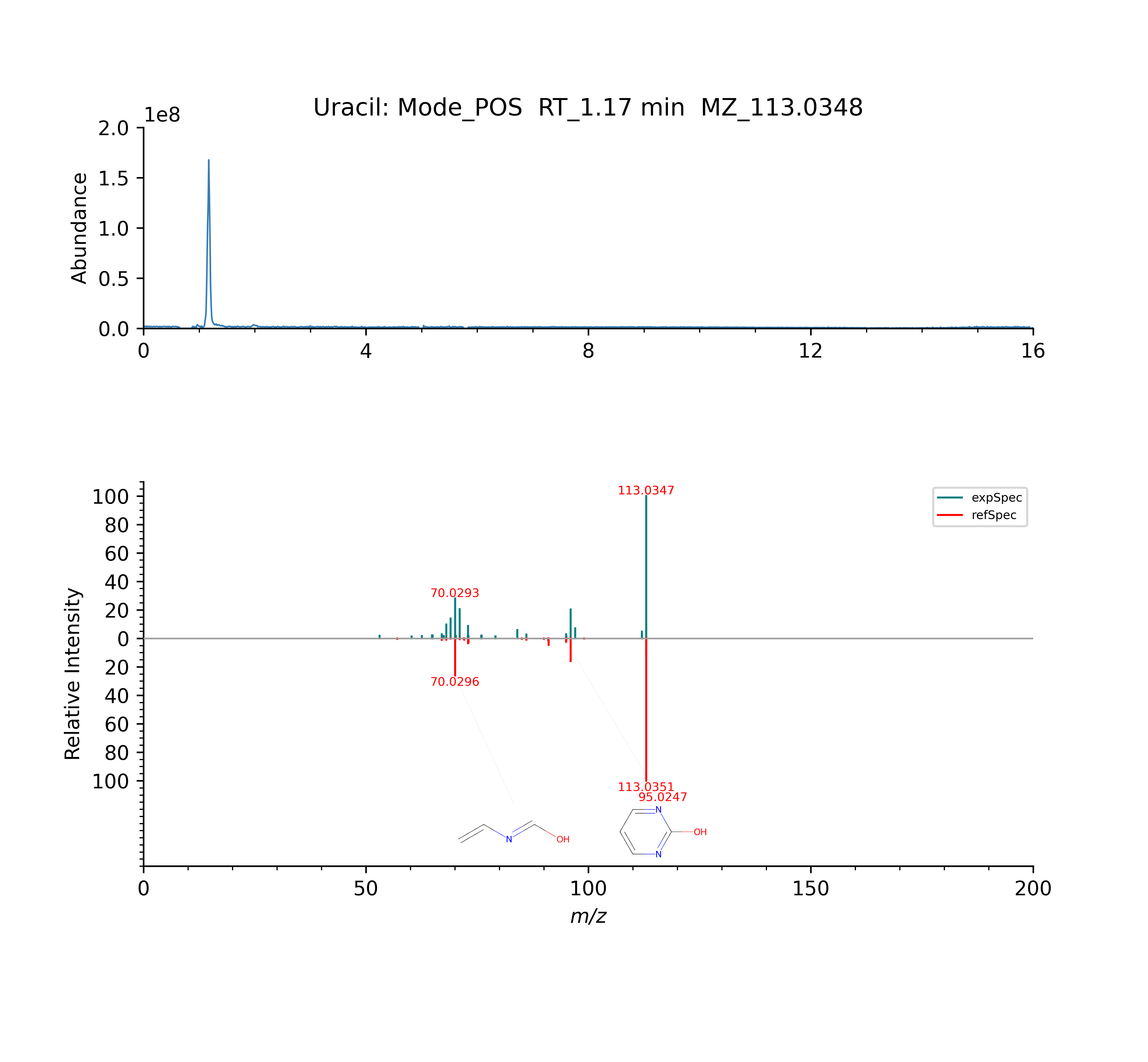

Supplement: Supplementary file 1 [file ijms-27-02203-s001.zip › ijms-4070482 Supplementary/Metabolite List Identified by LC-MS_MS from Rhodiola Species/161.png]

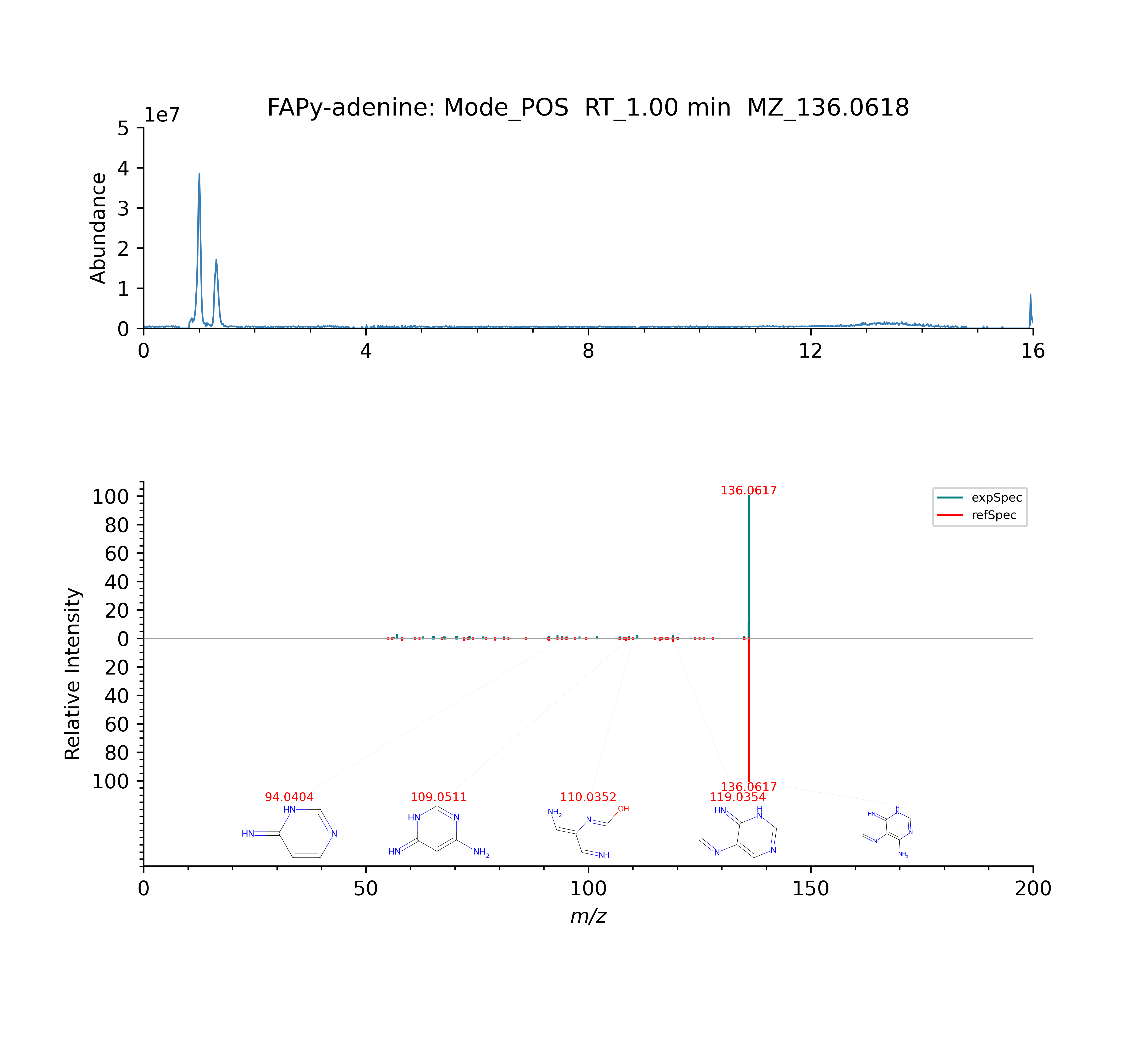

Supplement: Supplementary file 1 [file ijms-27-02203-s001.zip › ijms-4070482 Supplementary/Metabolite List Identified by LC-MS_MS from Rhodiola Species/162.png]

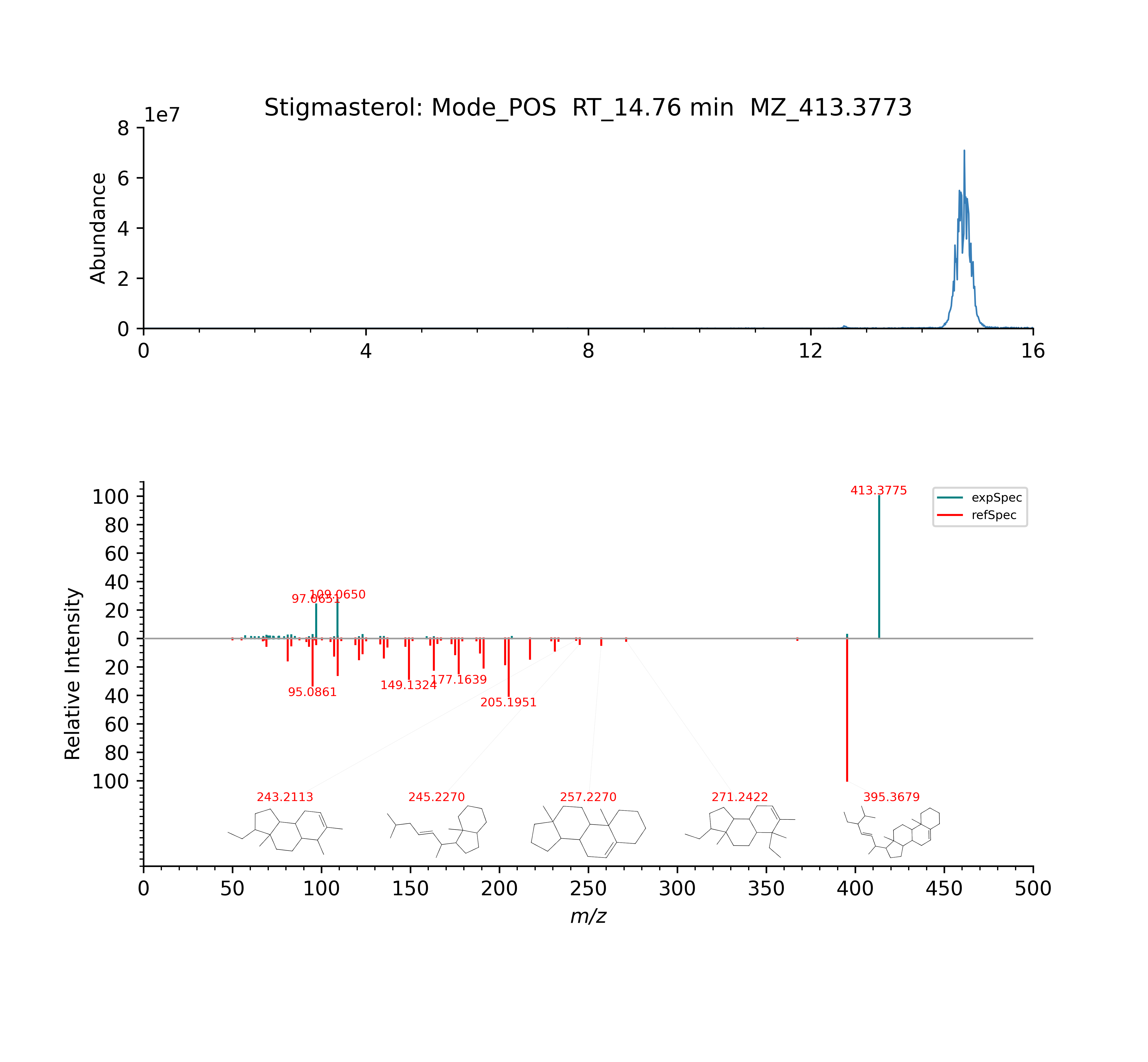

Supplement: Supplementary file 1 [file ijms-27-02203-s001.zip › ijms-4070482 Supplementary/Metabolite List Identified by LC-MS_MS from Rhodiola Species/163.png]

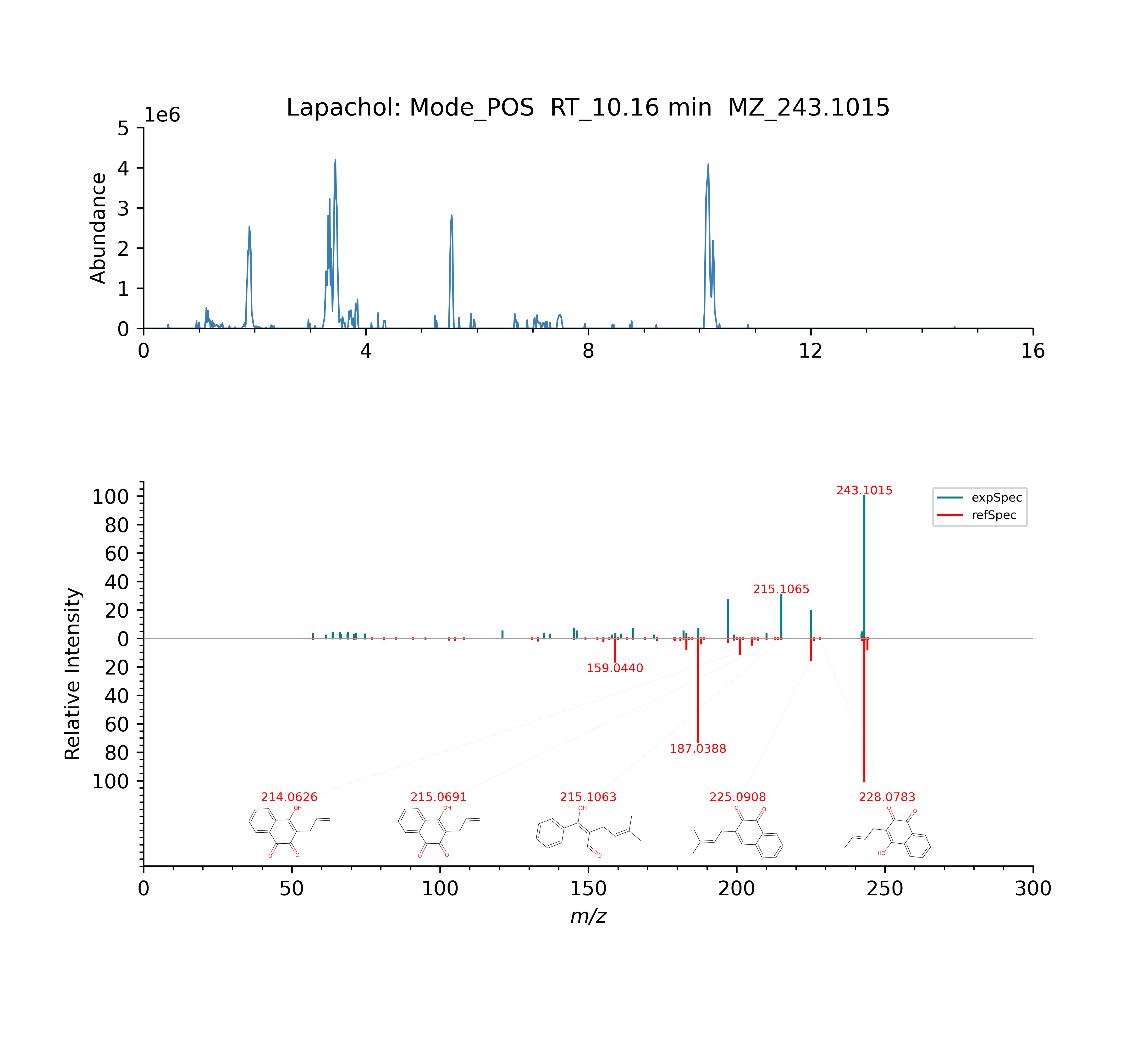

Supplement: Supplementary file 1 [file ijms-27-02203-s001.zip › ijms-4070482 Supplementary/Metabolite List Identified by LC-MS_MS from Rhodiola Species/164.png]

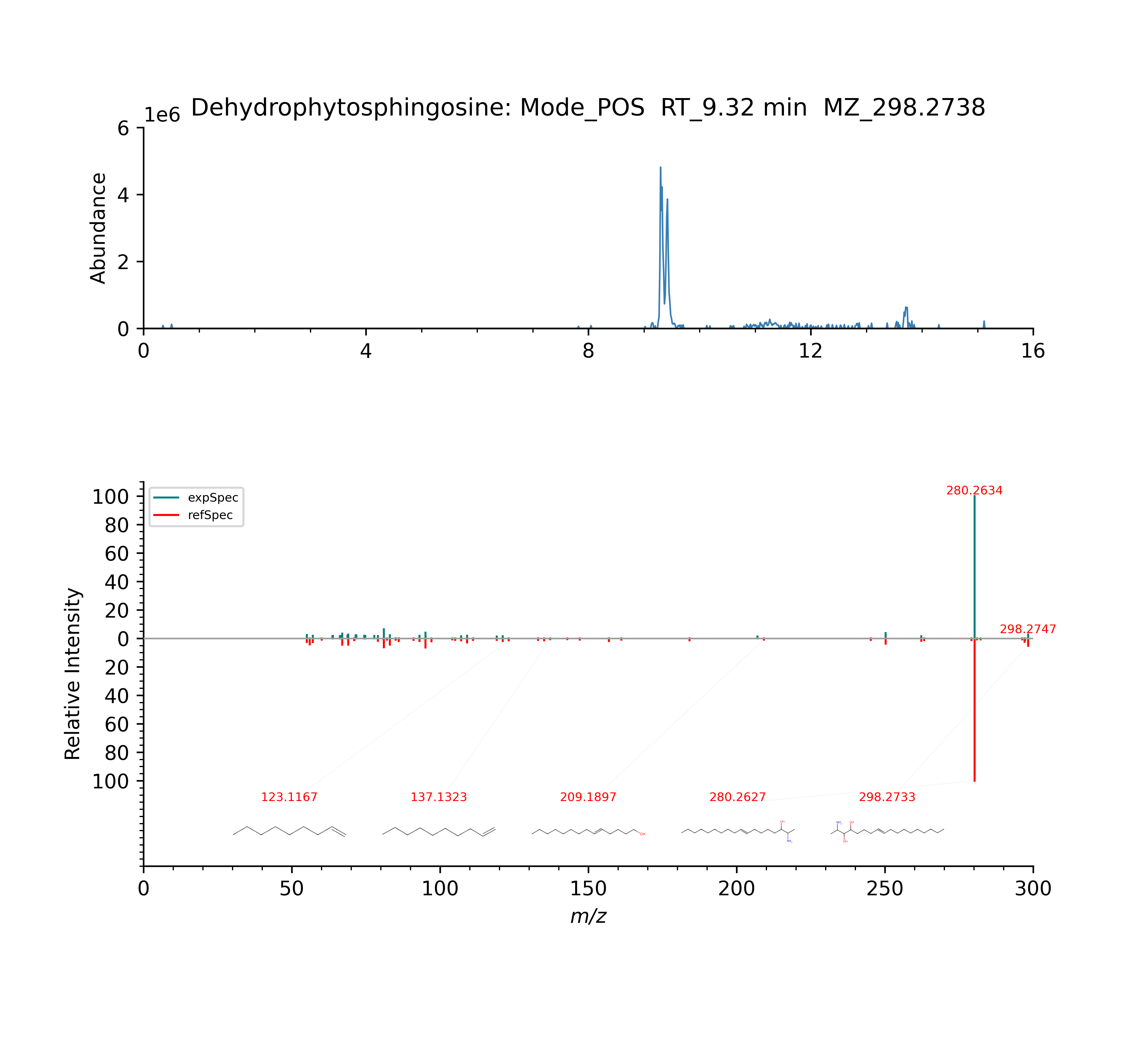

Supplement: Supplementary file 1 [file ijms-27-02203-s001.zip › ijms-4070482 Supplementary/Metabolite List Identified by LC-MS_MS from Rhodiola Species/165.png]

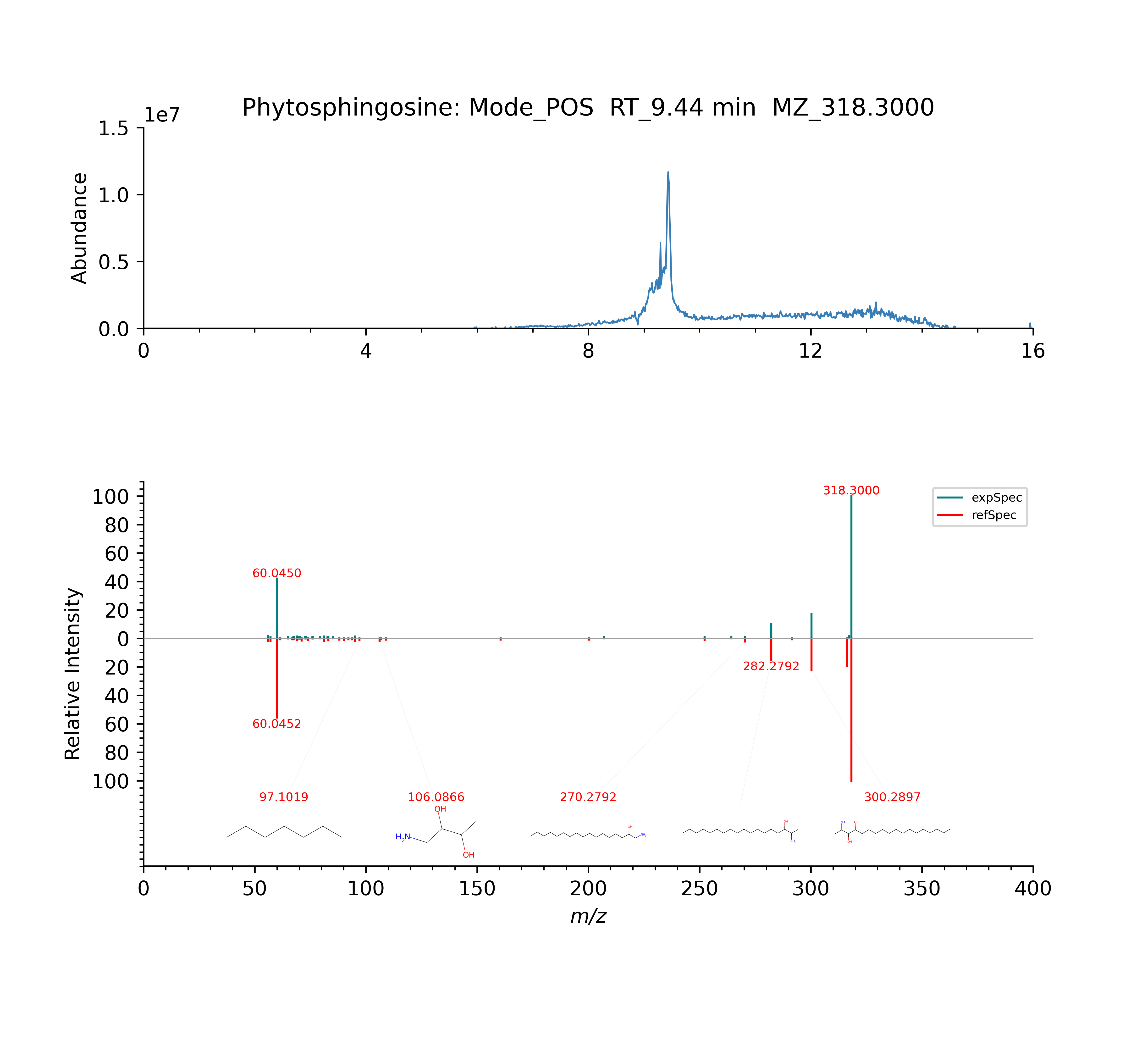

Supplement: Supplementary file 1 [file ijms-27-02203-s001.zip › ijms-4070482 Supplementary/Metabolite List Identified by LC-MS_MS from Rhodiola Species/166.png]

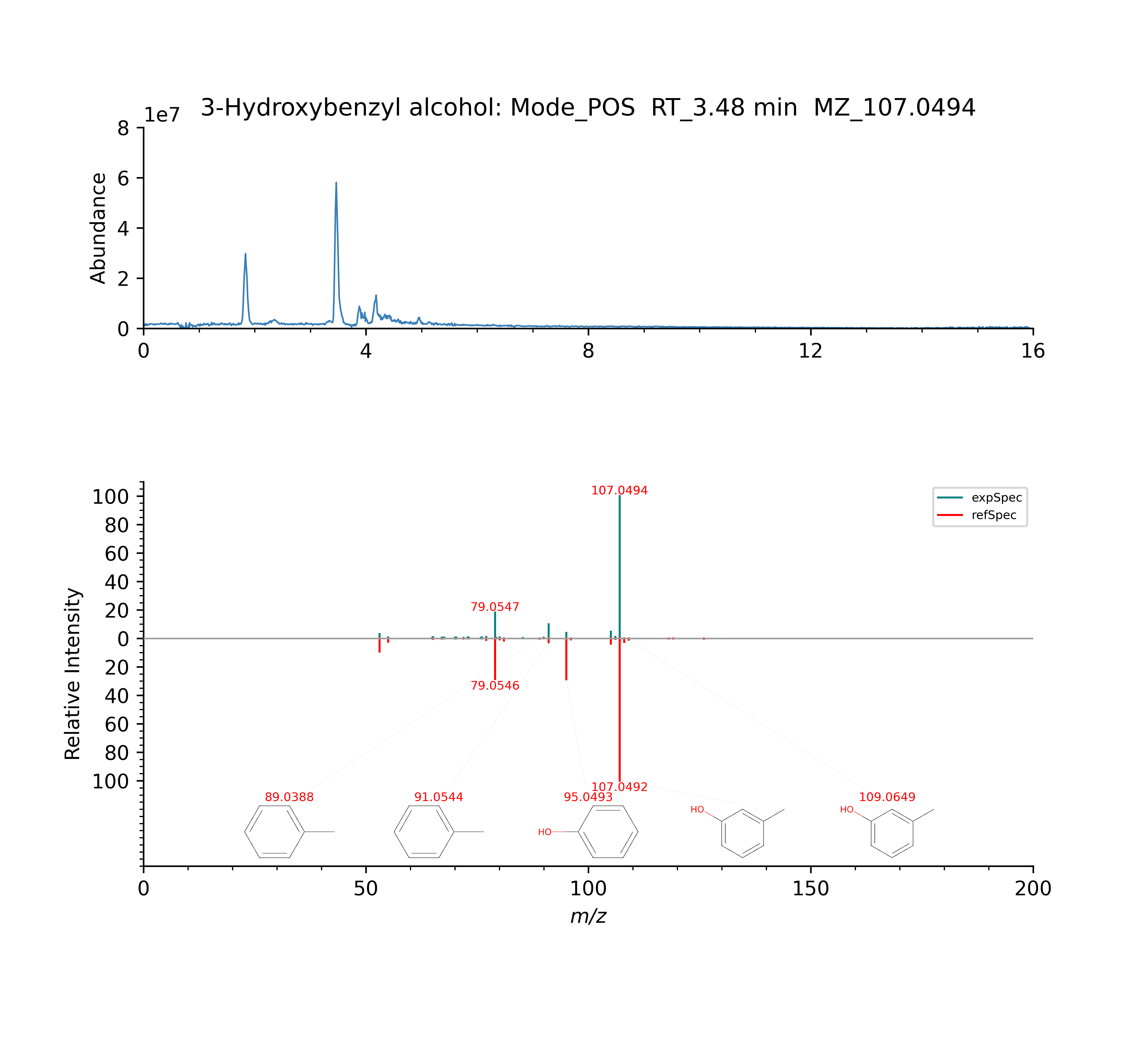

Supplement: Supplementary file 1 [file ijms-27-02203-s001.zip › ijms-4070482 Supplementary/Metabolite List Identified by LC-MS_MS from Rhodiola Species/167.png]

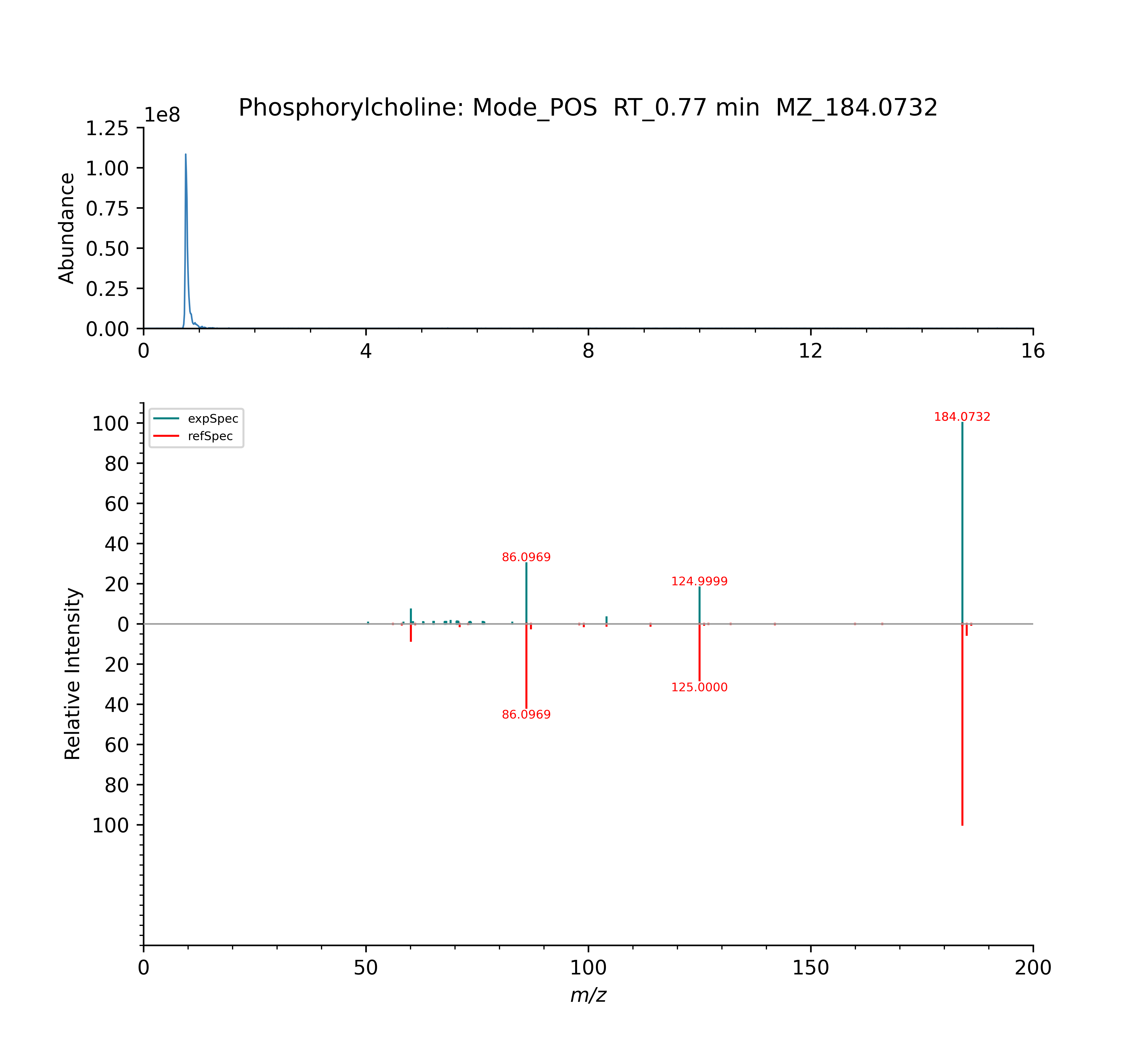

Supplement: Supplementary file 1 [file ijms-27-02203-s001.zip › ijms-4070482 Supplementary/Metabolite List Identified by LC-MS_MS from Rhodiola Species/168.png]

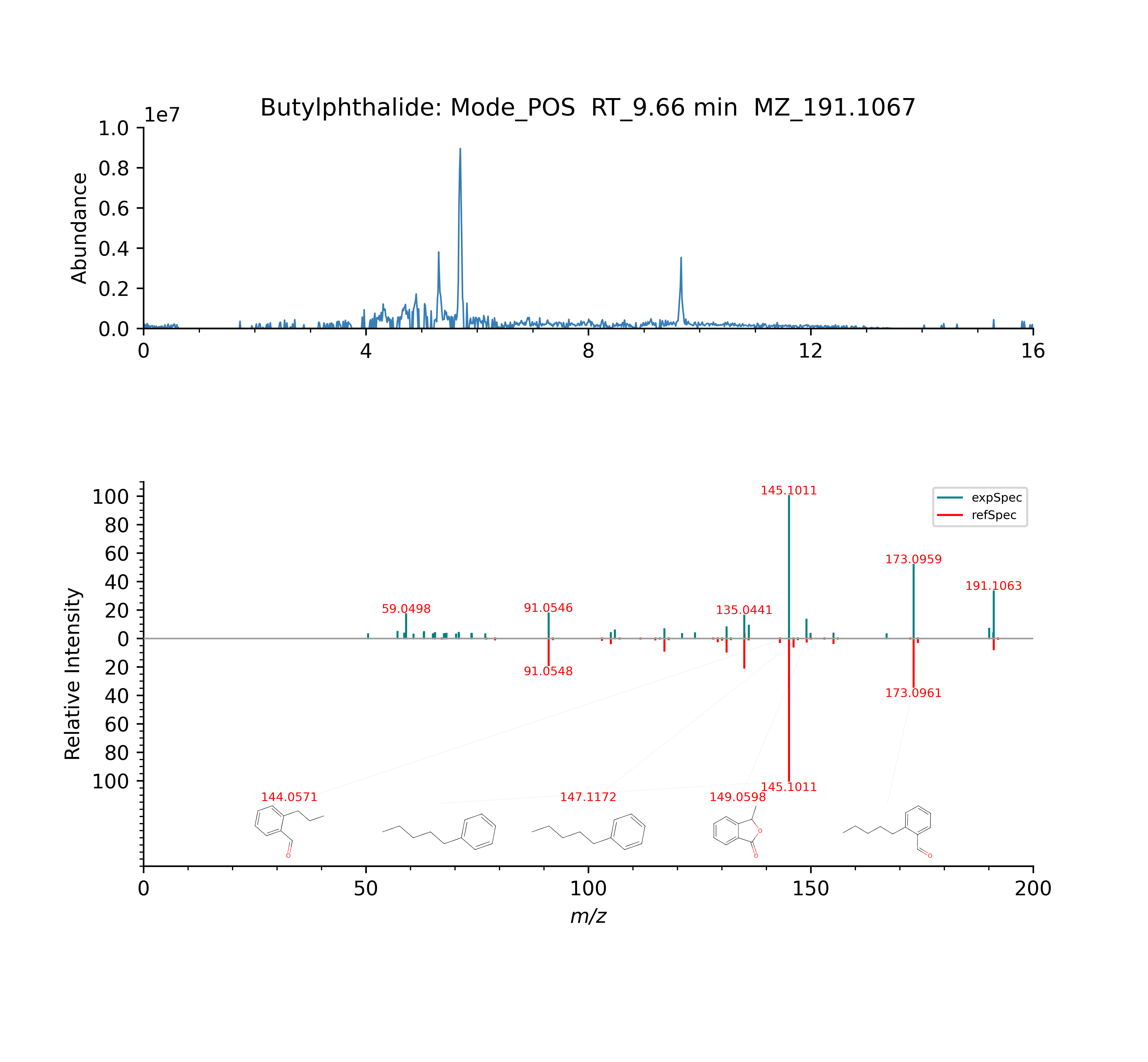

Supplement: Supplementary file 1 [file ijms-27-02203-s001.zip › ijms-4070482 Supplementary/Metabolite List Identified by LC-MS_MS from Rhodiola Species/169.png]

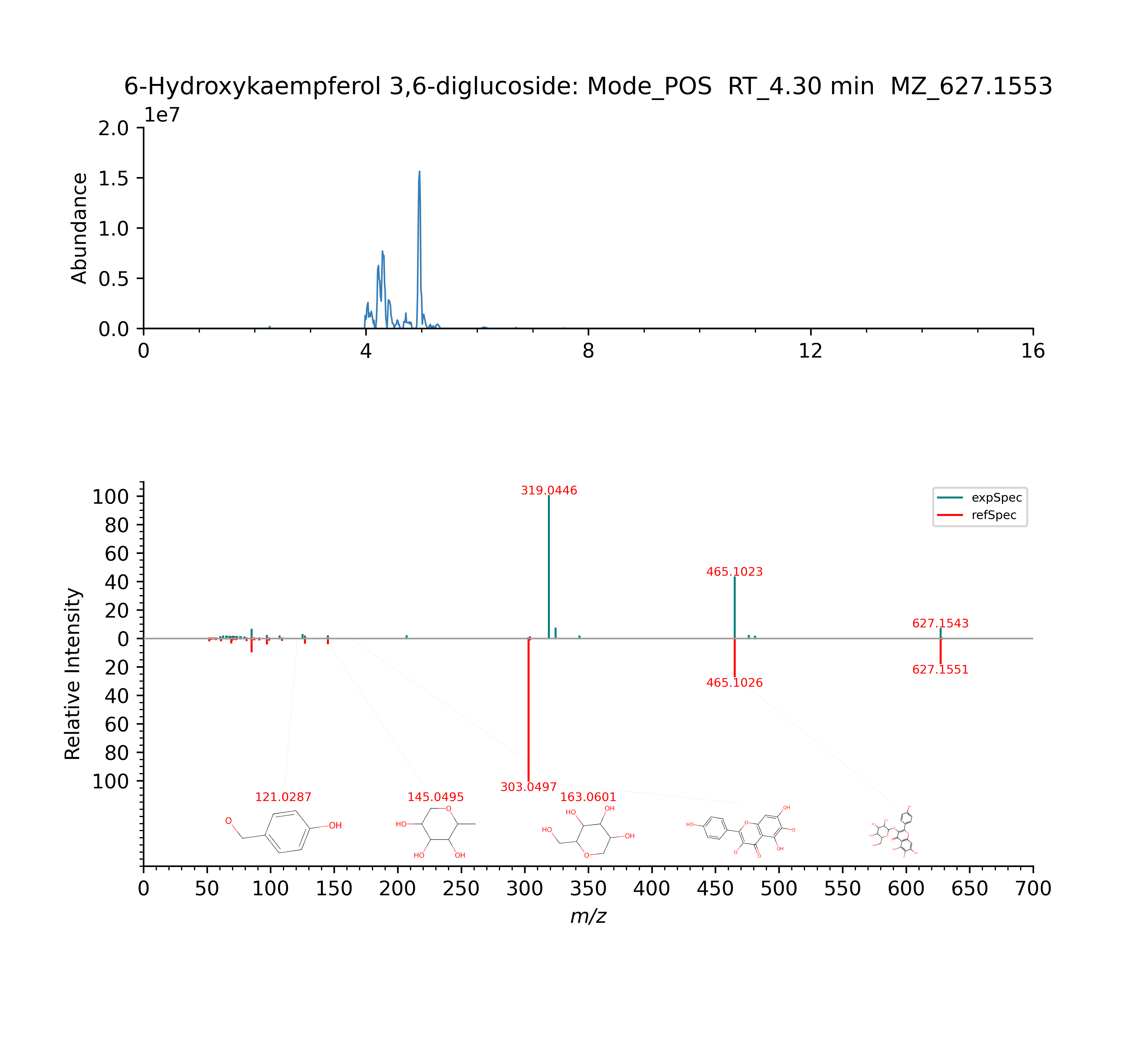

Supplement: Supplementary file 1 [file ijms-27-02203-s001.zip › ijms-4070482 Supplementary/Metabolite List Identified by LC-MS_MS from Rhodiola Species/17.png]

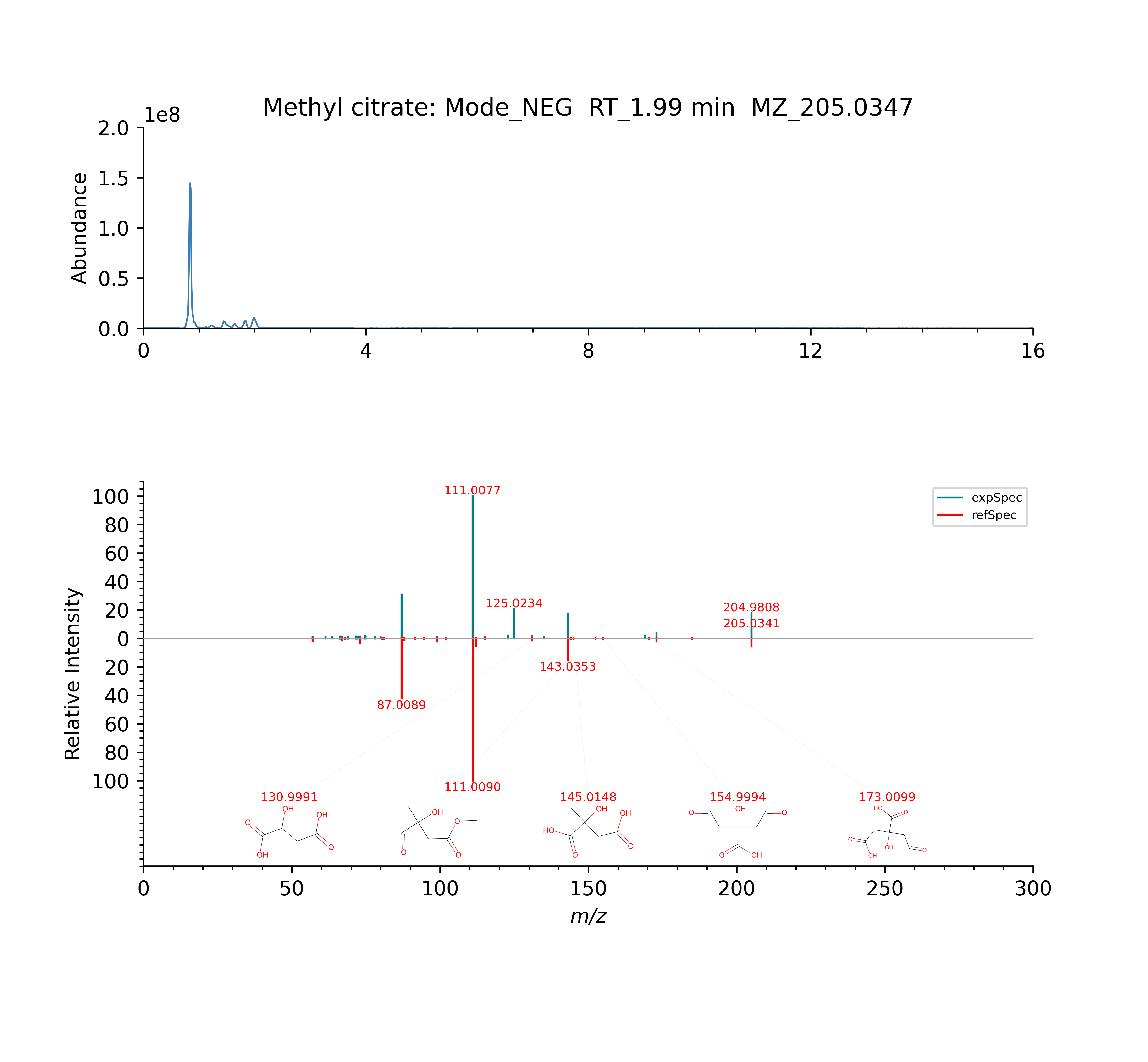

Supplement: Supplementary file 1 [file ijms-27-02203-s001.zip › ijms-4070482 Supplementary/Metabolite List Identified by LC-MS_MS from Rhodiola Species/170.png]

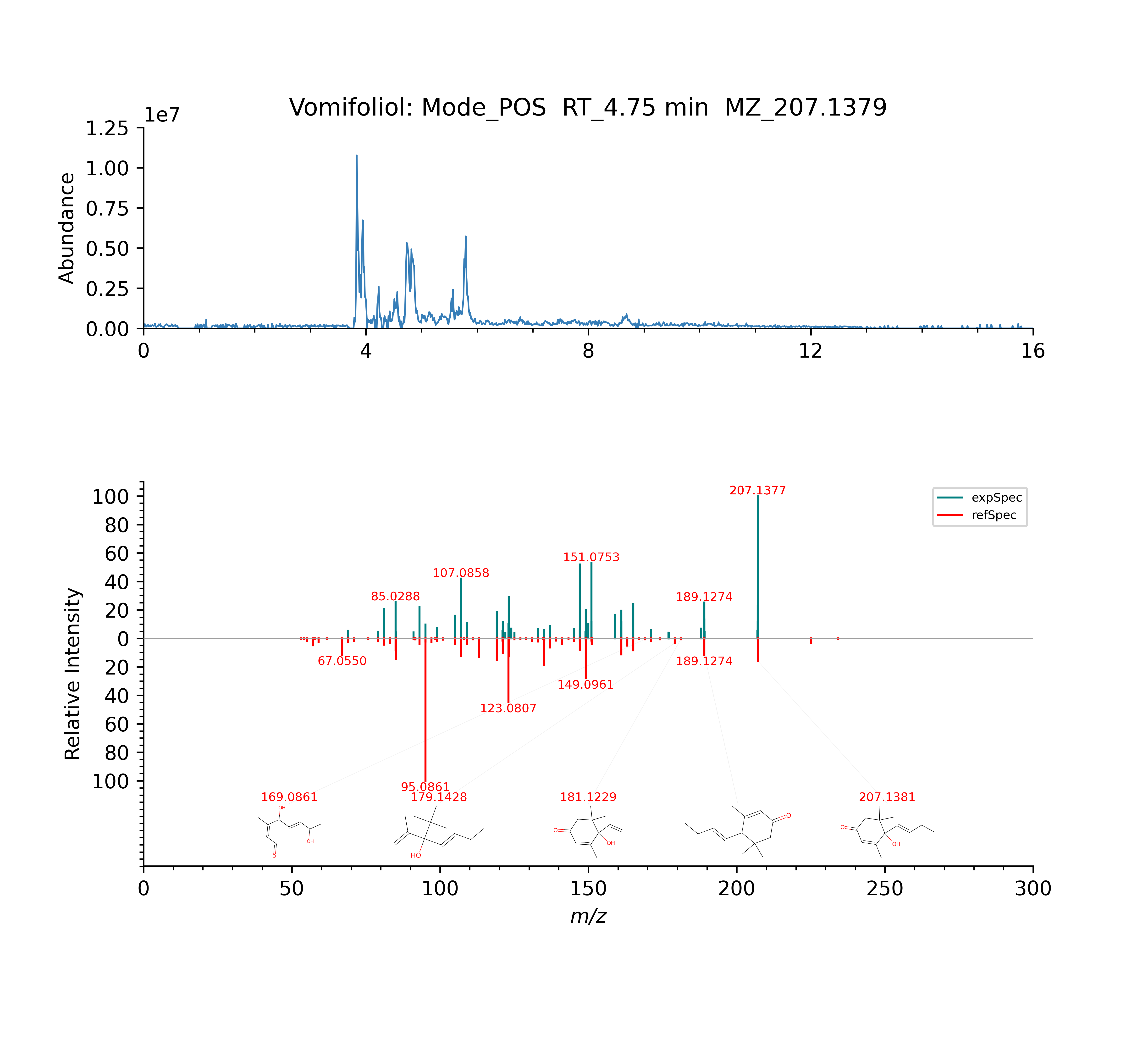

Supplement: Supplementary file 1 [file ijms-27-02203-s001.zip › ijms-4070482 Supplementary/Metabolite List Identified by LC-MS_MS from Rhodiola Species/171.png]

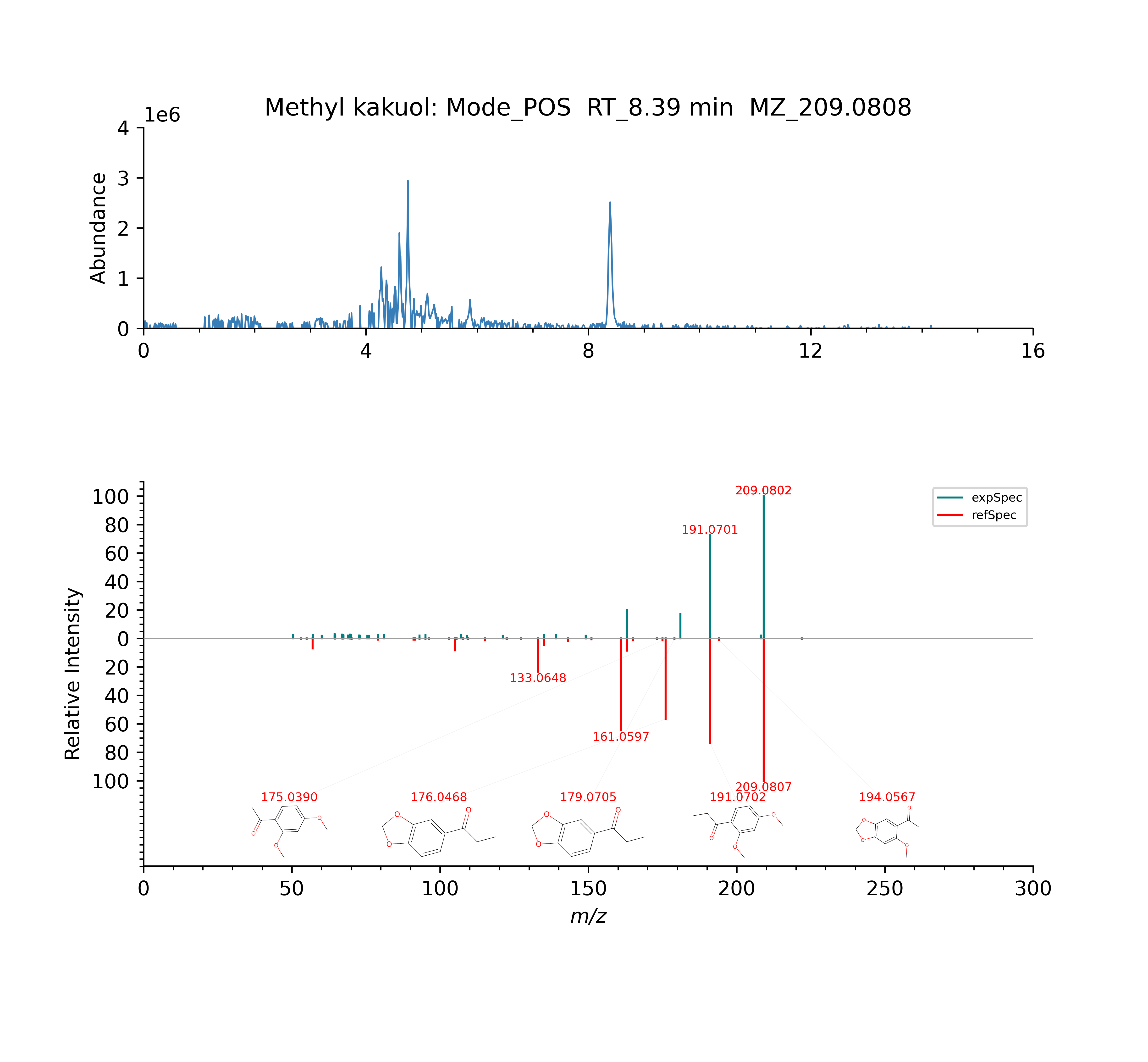

Supplement: Supplementary file 1 [file ijms-27-02203-s001.zip › ijms-4070482 Supplementary/Metabolite List Identified by LC-MS_MS from Rhodiola Species/172.png]

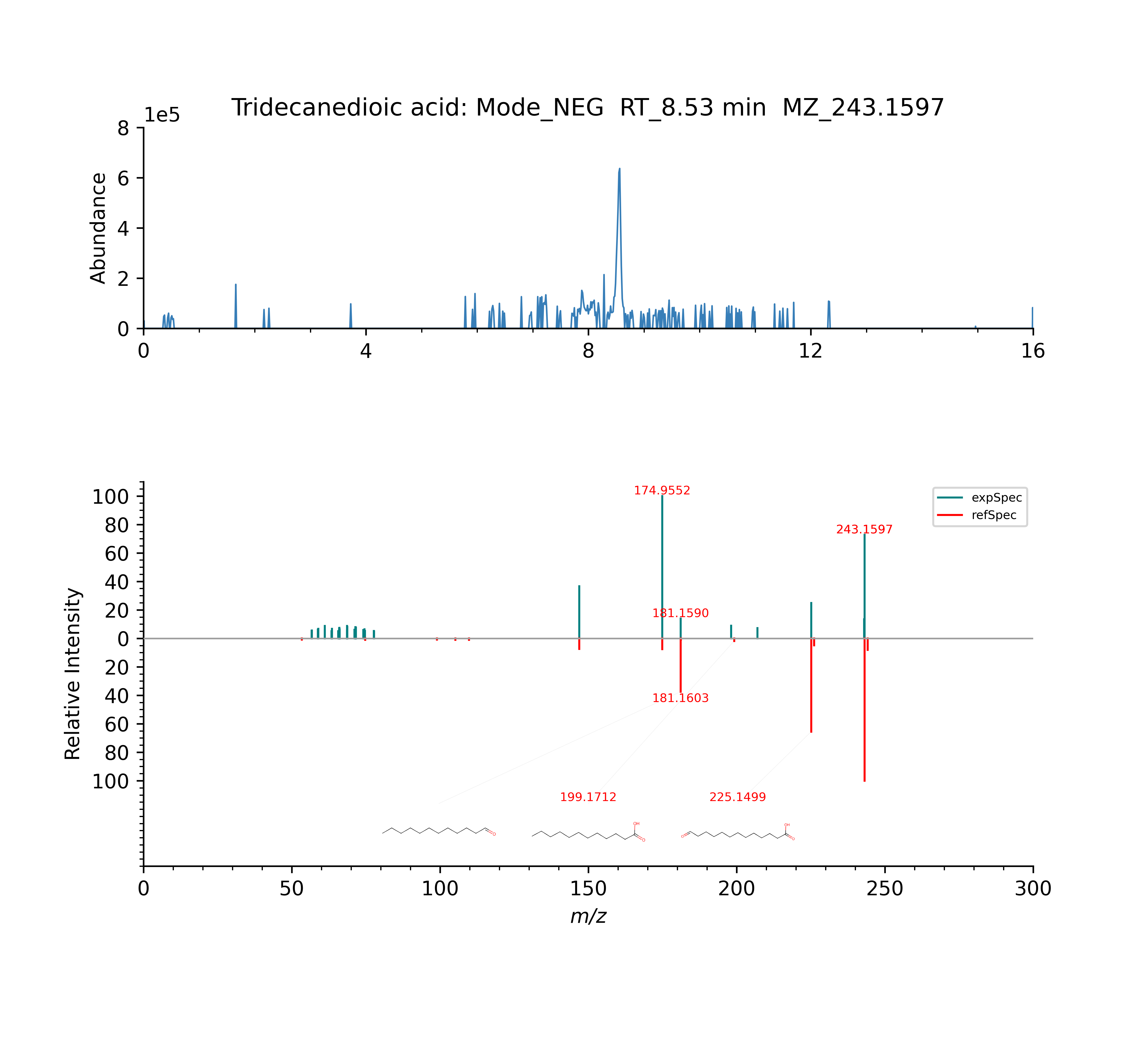

Supplement: Supplementary file 1 [file ijms-27-02203-s001.zip › ijms-4070482 Supplementary/Metabolite List Identified by LC-MS_MS from Rhodiola Species/173.png]

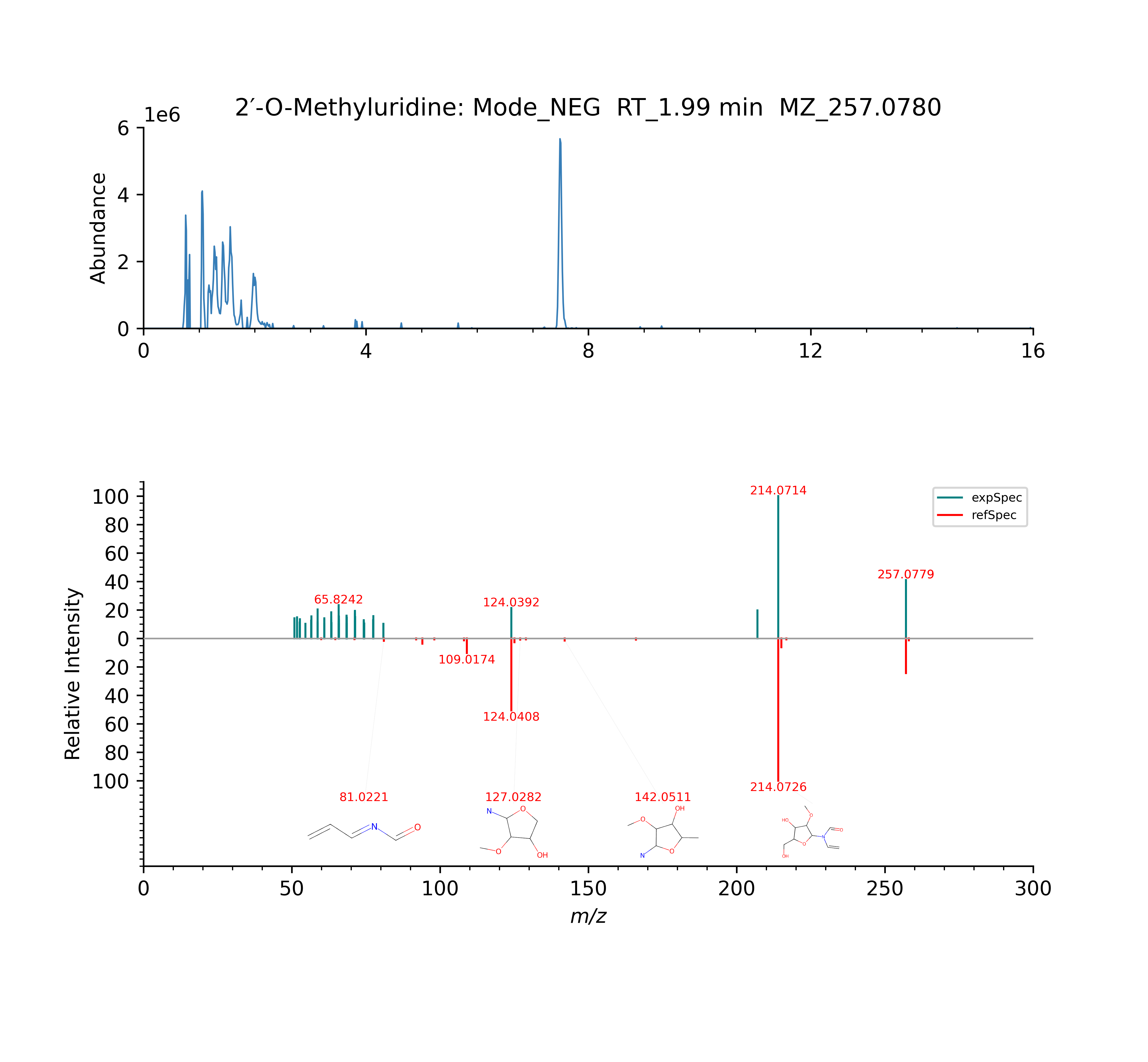

Supplement: Supplementary file 1 [file ijms-27-02203-s001.zip › ijms-4070482 Supplementary/Metabolite List Identified by LC-MS_MS from Rhodiola Species/174.png]

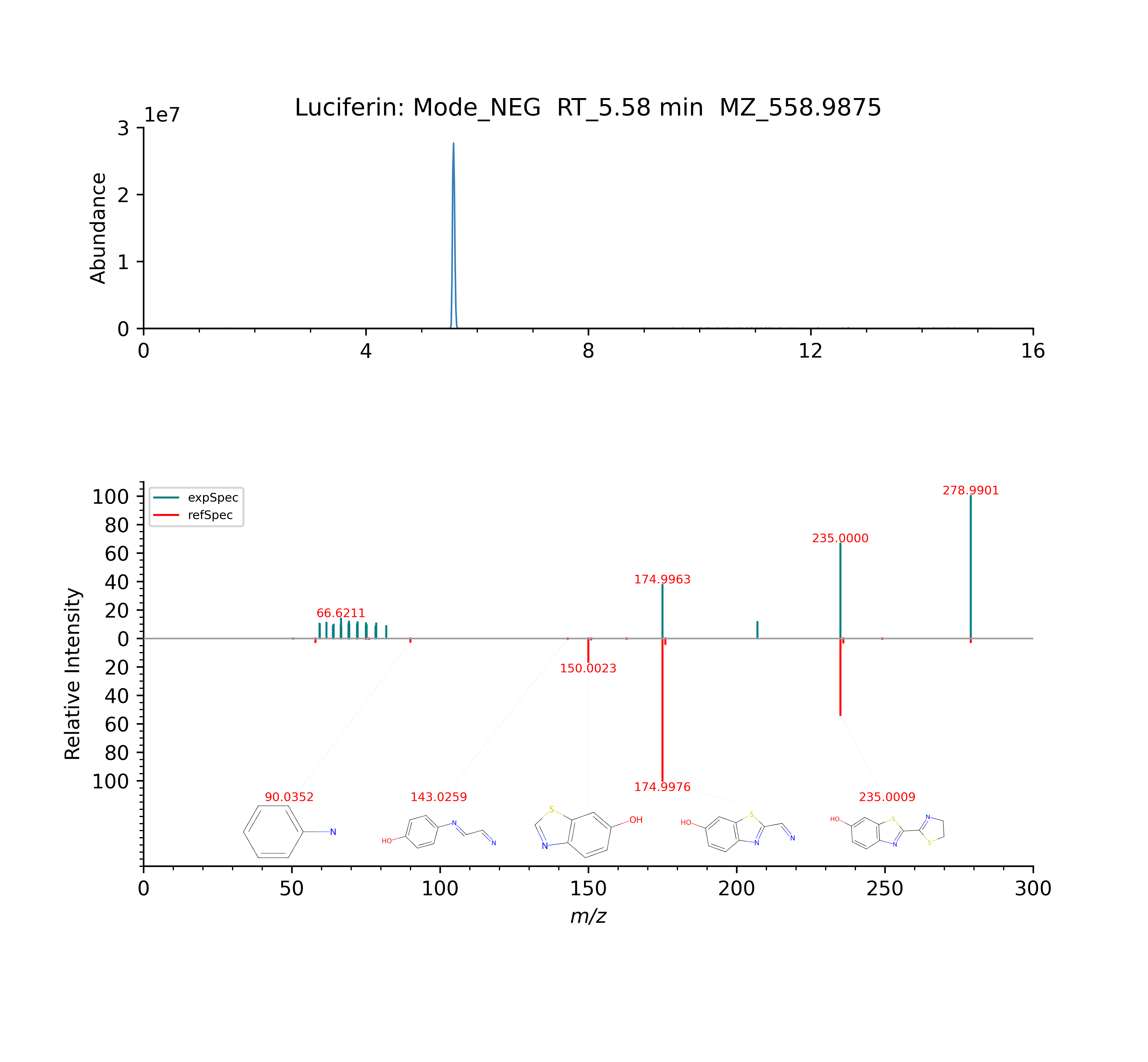

Supplement: Supplementary file 1 [file ijms-27-02203-s001.zip › ijms-4070482 Supplementary/Metabolite List Identified by LC-MS_MS from Rhodiola Species/175.png]

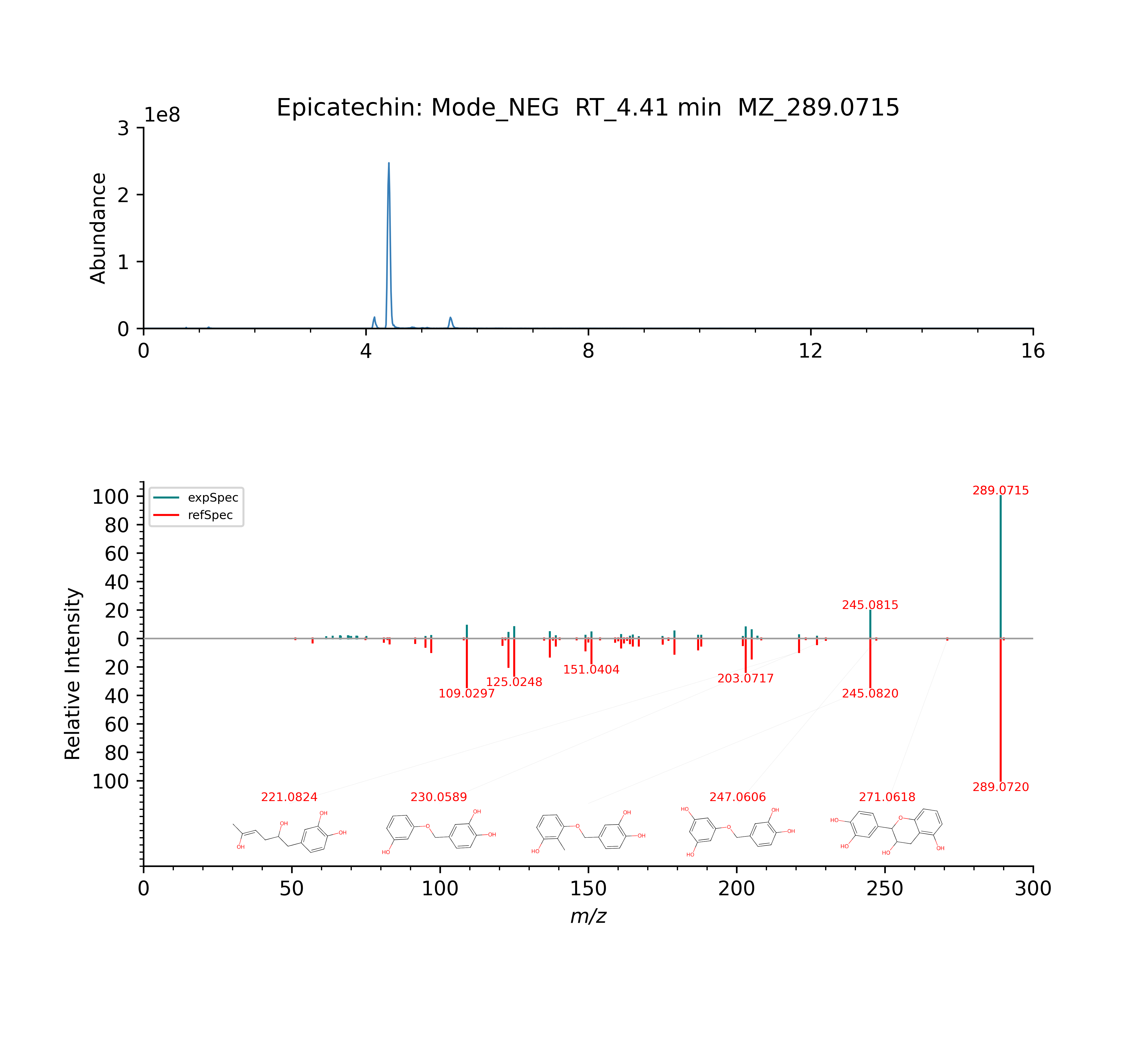

Supplement: Supplementary file 1 [file ijms-27-02203-s001.zip › ijms-4070482 Supplementary/Metabolite List Identified by LC-MS_MS from Rhodiola Species/18.png]

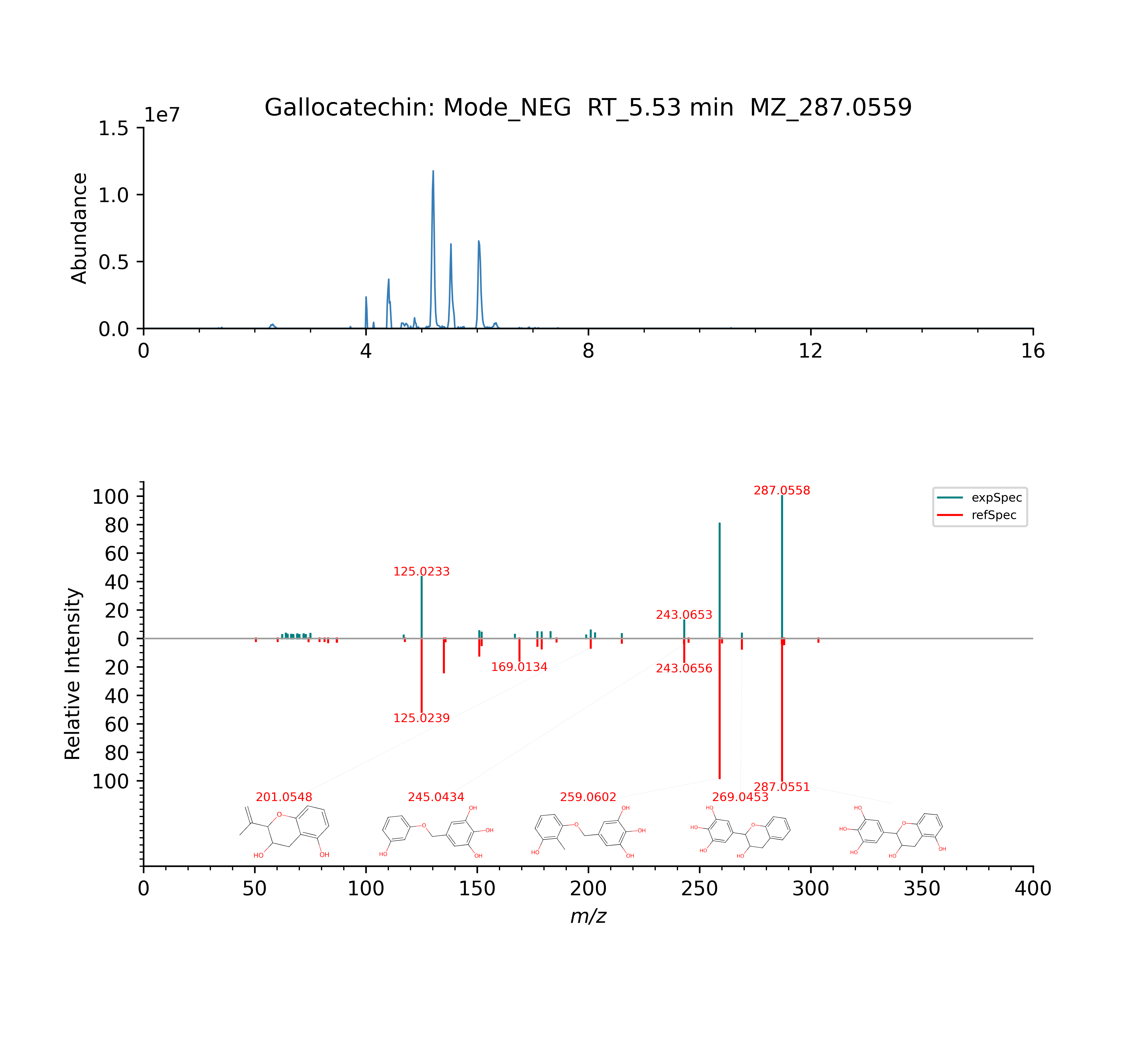

Supplement: Supplementary file 1 [file ijms-27-02203-s001.zip › ijms-4070482 Supplementary/Metabolite List Identified by LC-MS_MS from Rhodiola Species/19.png]

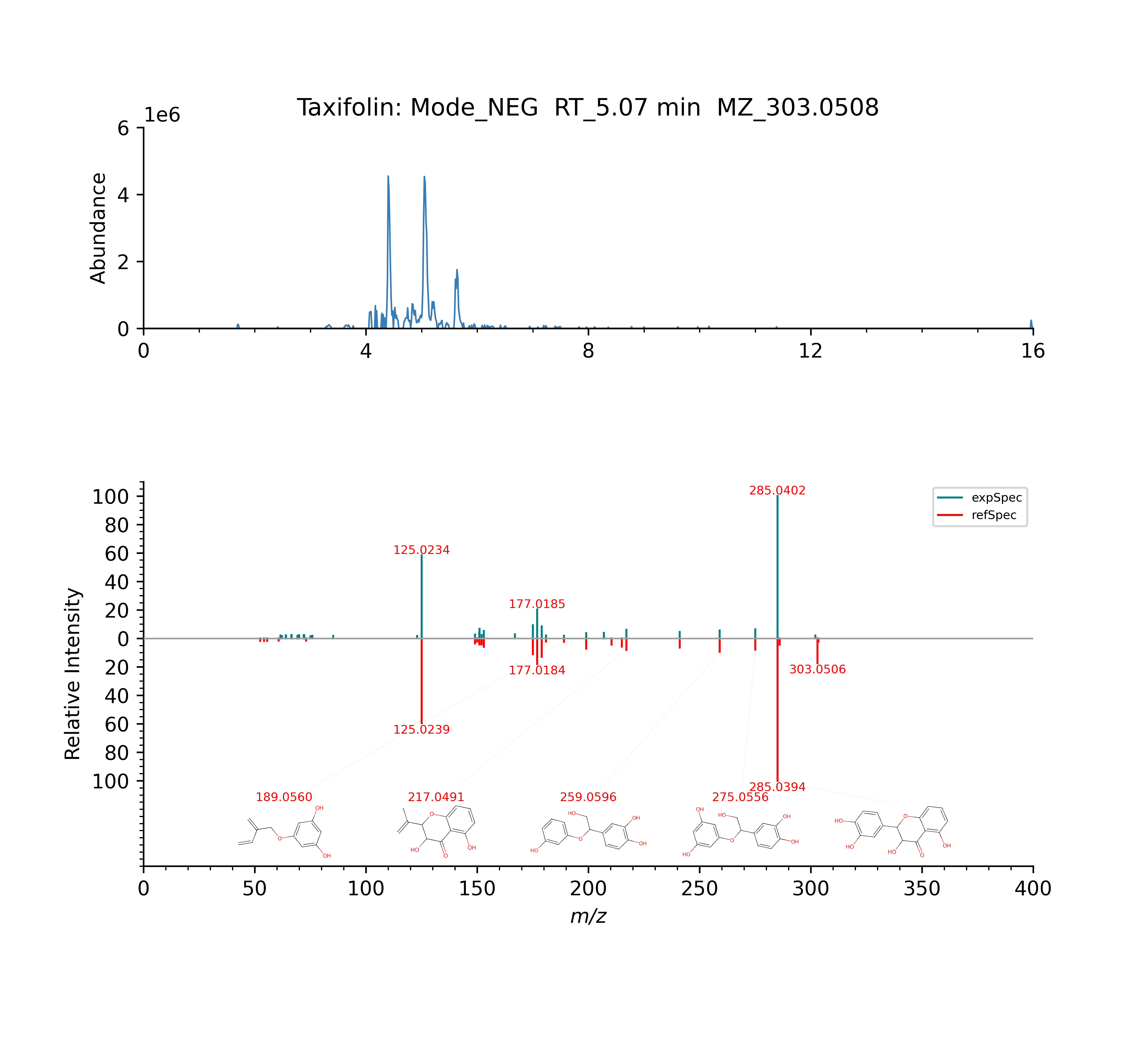

Supplement: Supplementary file 1 [file ijms-27-02203-s001.zip › ijms-4070482 Supplementary/Metabolite List Identified by LC-MS_MS from Rhodiola Species/2.png]

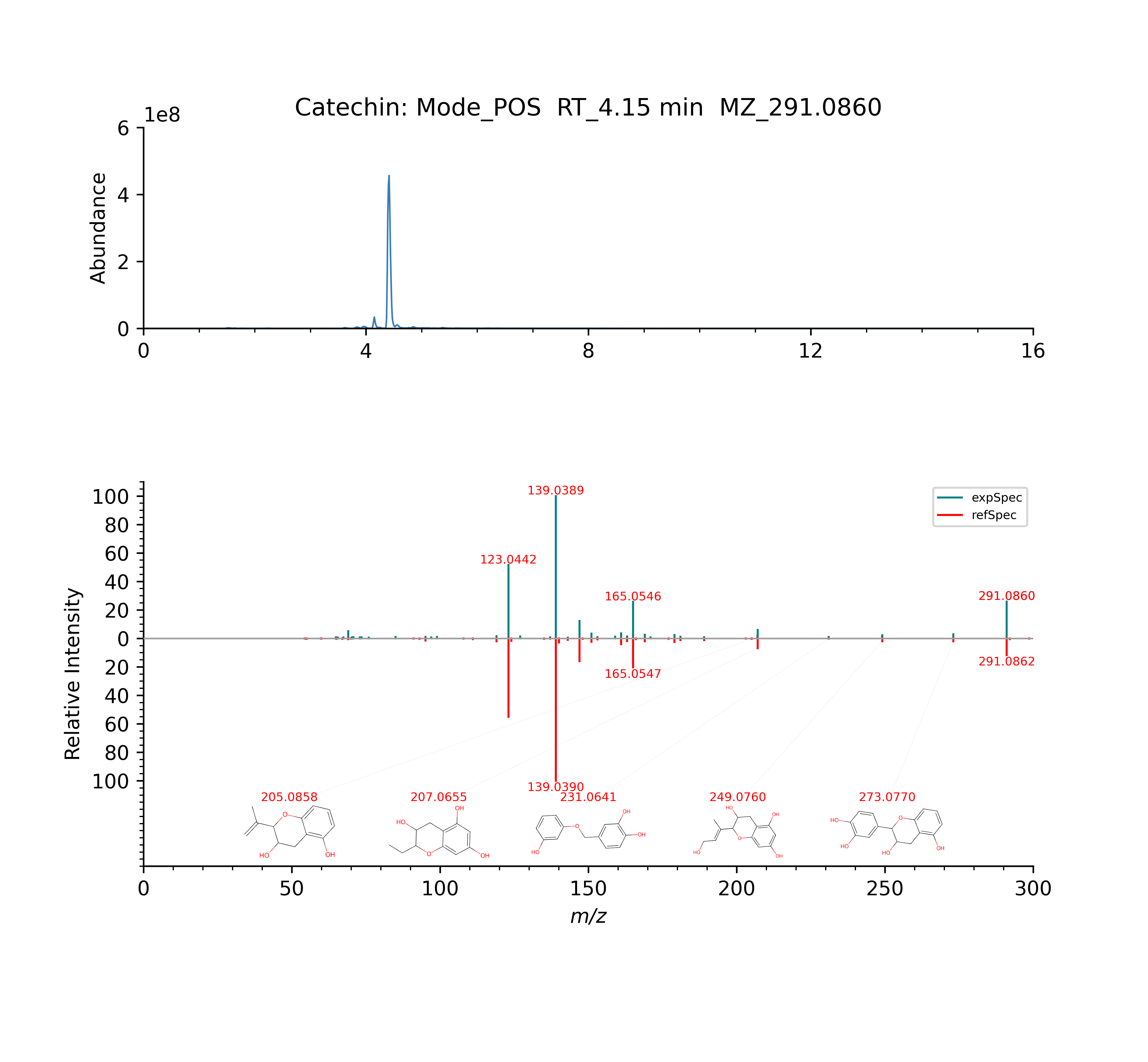

Supplement: Supplementary file 1 [file ijms-27-02203-s001.zip › ijms-4070482 Supplementary/Metabolite List Identified by LC-MS_MS from Rhodiola Species/20.png]

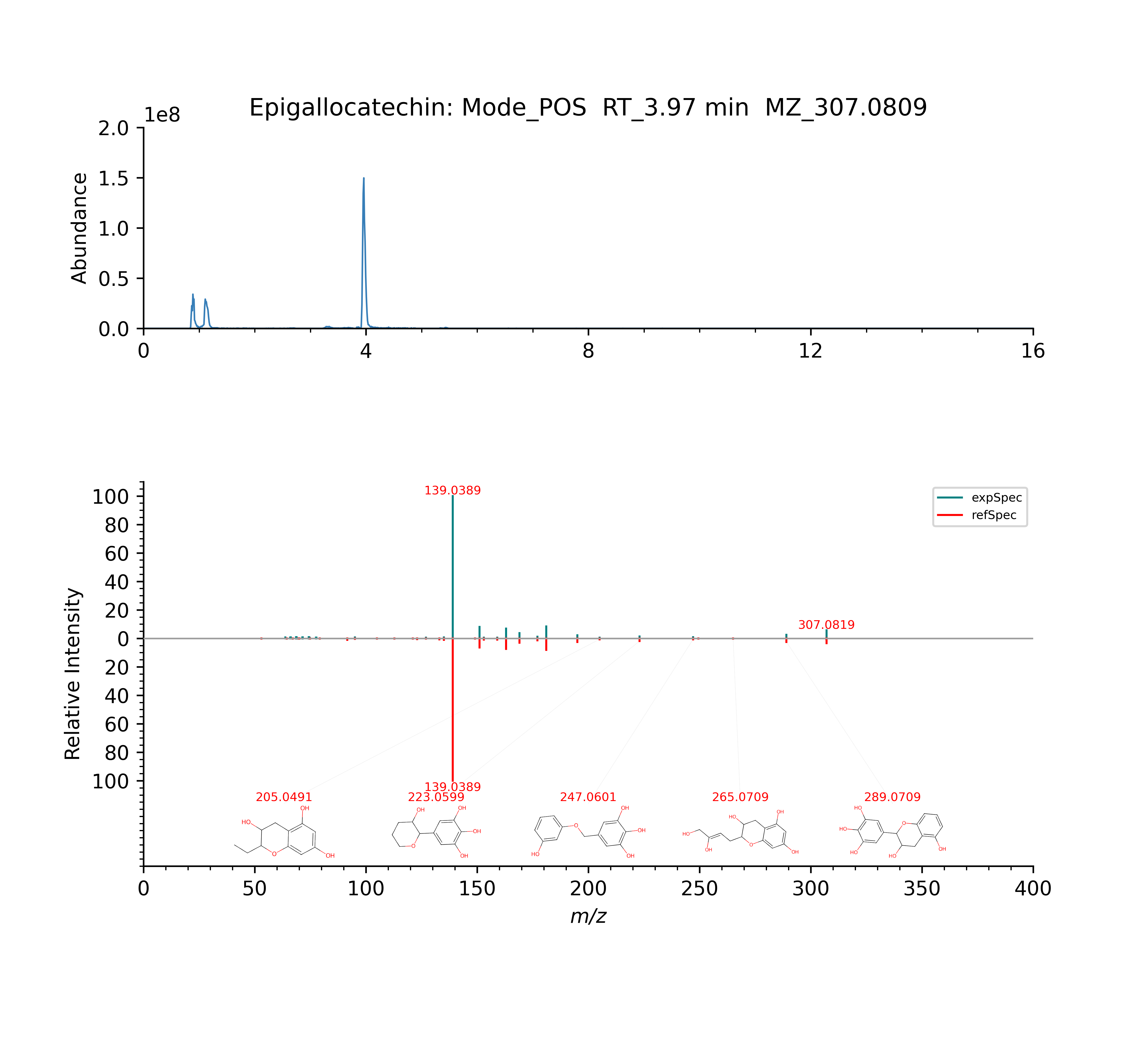

Supplement: Supplementary file 1 [file ijms-27-02203-s001.zip › ijms-4070482 Supplementary/Metabolite List Identified by LC-MS_MS from Rhodiola Species/21.png]

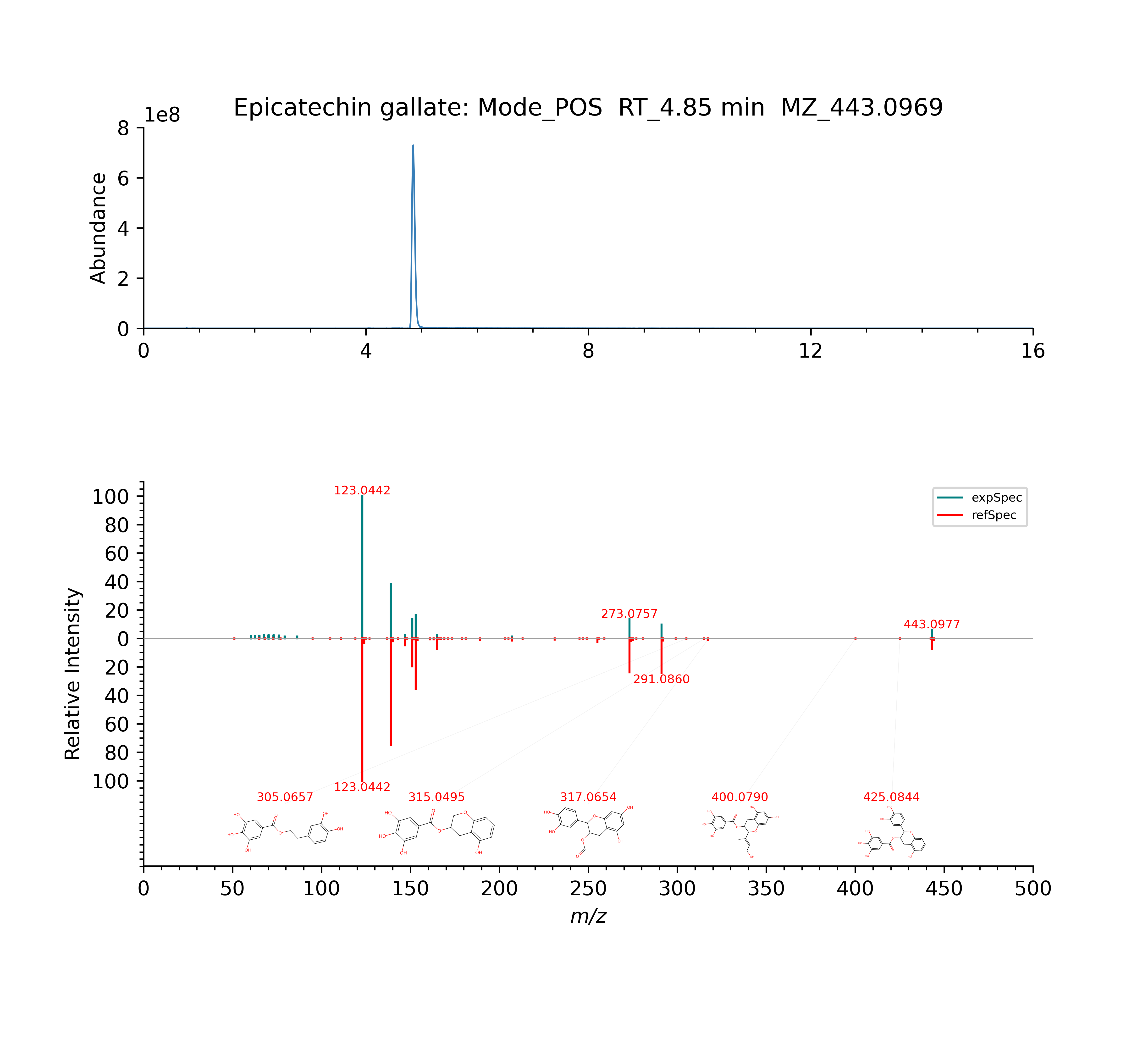

Supplement: Supplementary file 1 [file ijms-27-02203-s001.zip › ijms-4070482 Supplementary/Metabolite List Identified by LC-MS_MS from Rhodiola Species/22.png]

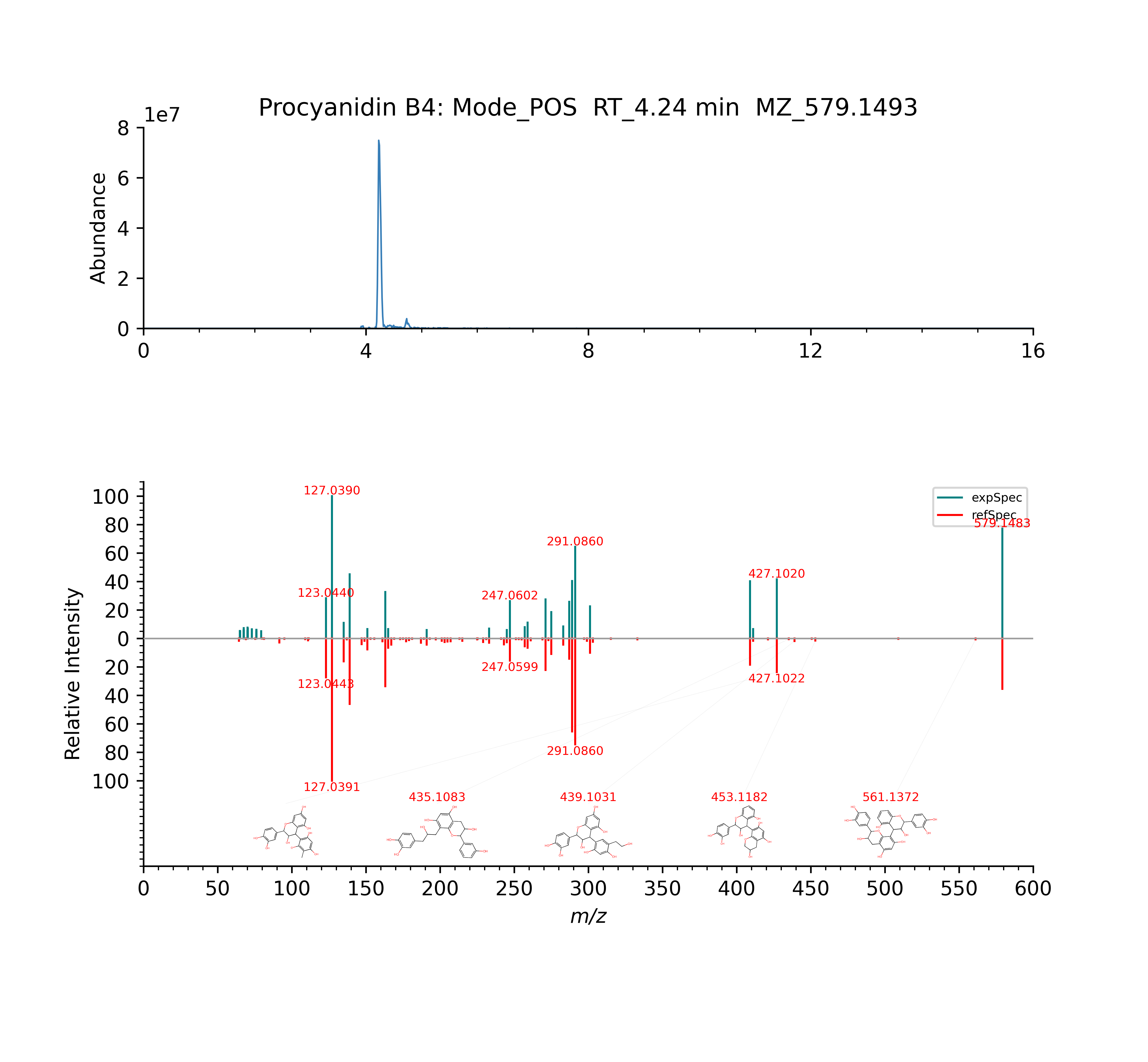

Supplement: Supplementary file 1 [file ijms-27-02203-s001.zip › ijms-4070482 Supplementary/Metabolite List Identified by LC-MS_MS from Rhodiola Species/23.png]

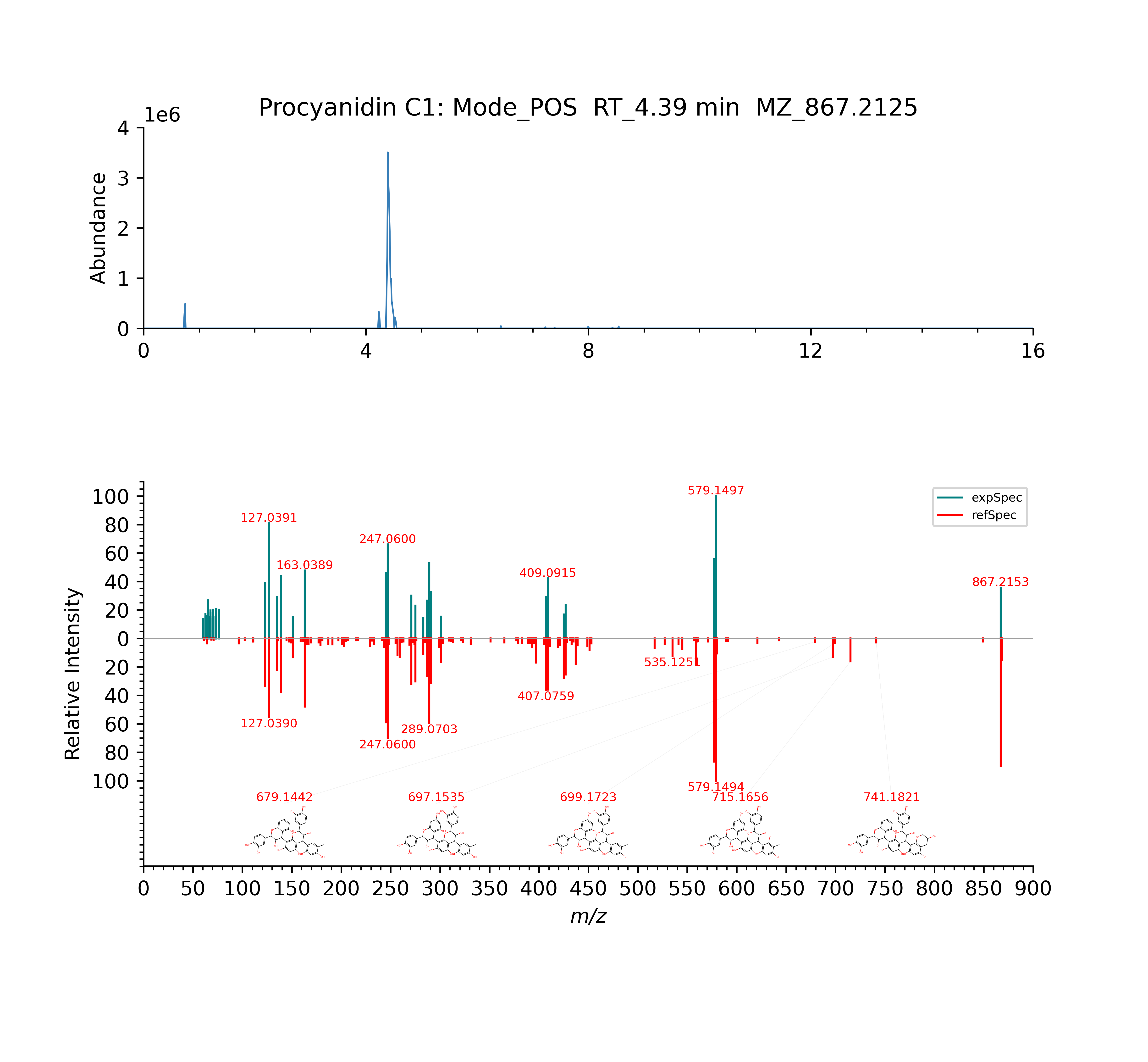

Supplement: Supplementary file 1 [file ijms-27-02203-s001.zip › ijms-4070482 Supplementary/Metabolite List Identified by LC-MS_MS from Rhodiola Species/24.png]

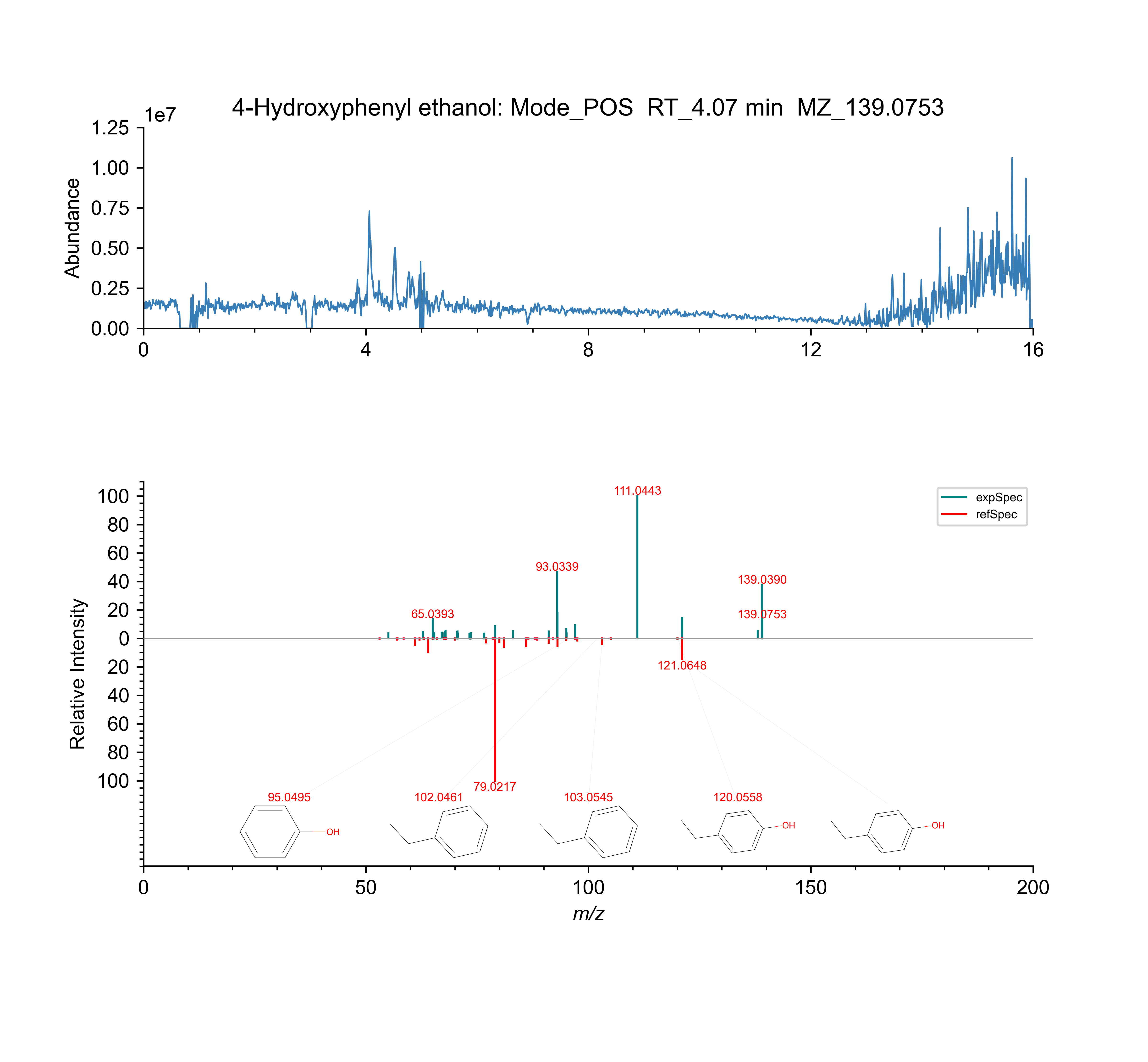

Supplement: Supplementary file 1 [file ijms-27-02203-s001.zip › ijms-4070482 Supplementary/Metabolite List Identified by LC-MS_MS from Rhodiola Species/25.png]

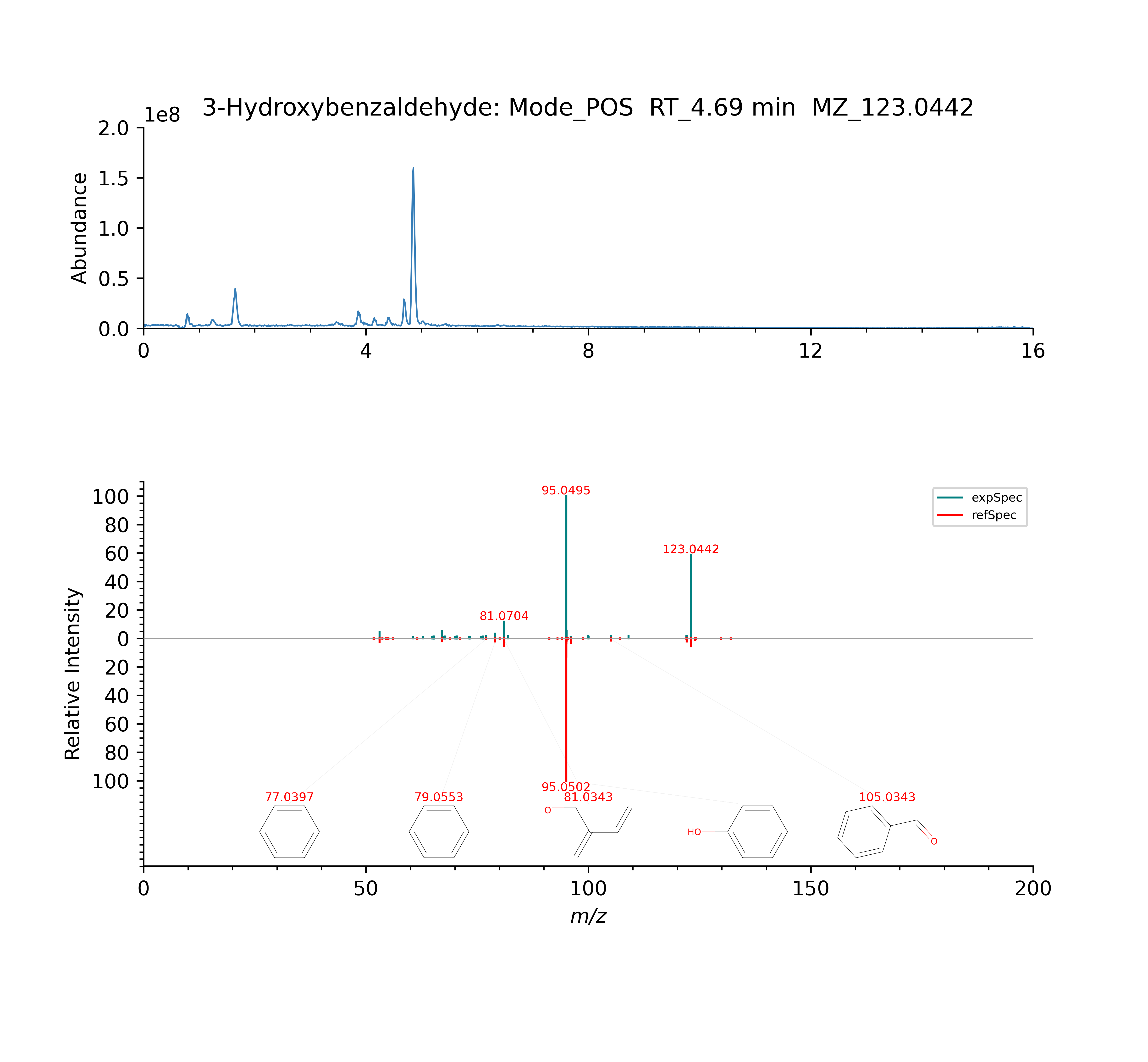

Supplement: Supplementary file 1 [file ijms-27-02203-s001.zip › ijms-4070482 Supplementary/Metabolite List Identified by LC-MS_MS from Rhodiola Species/26.png]

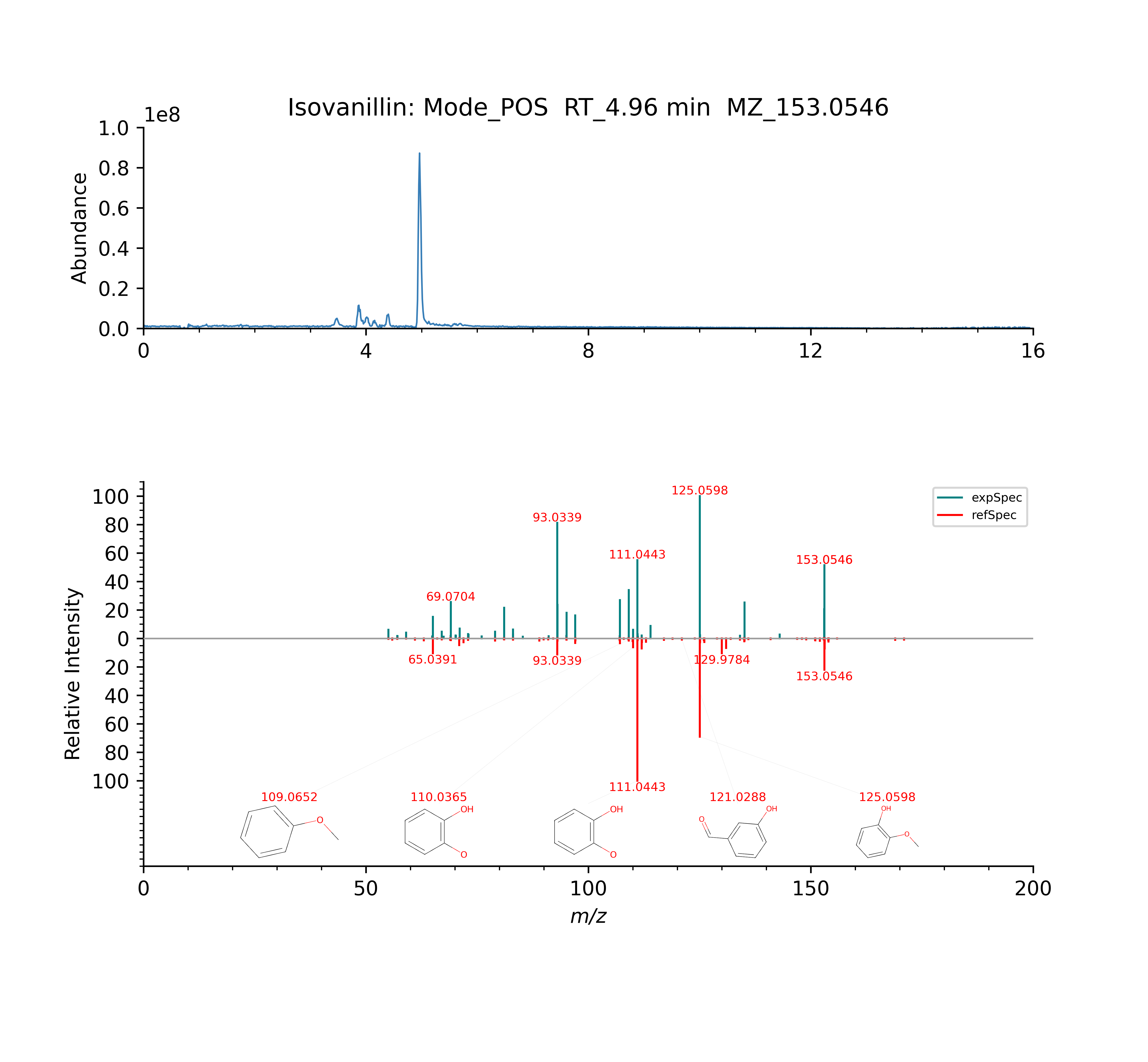

Supplement: Supplementary file 1 [file ijms-27-02203-s001.zip › ijms-4070482 Supplementary/Metabolite List Identified by LC-MS_MS from Rhodiola Species/27.png]

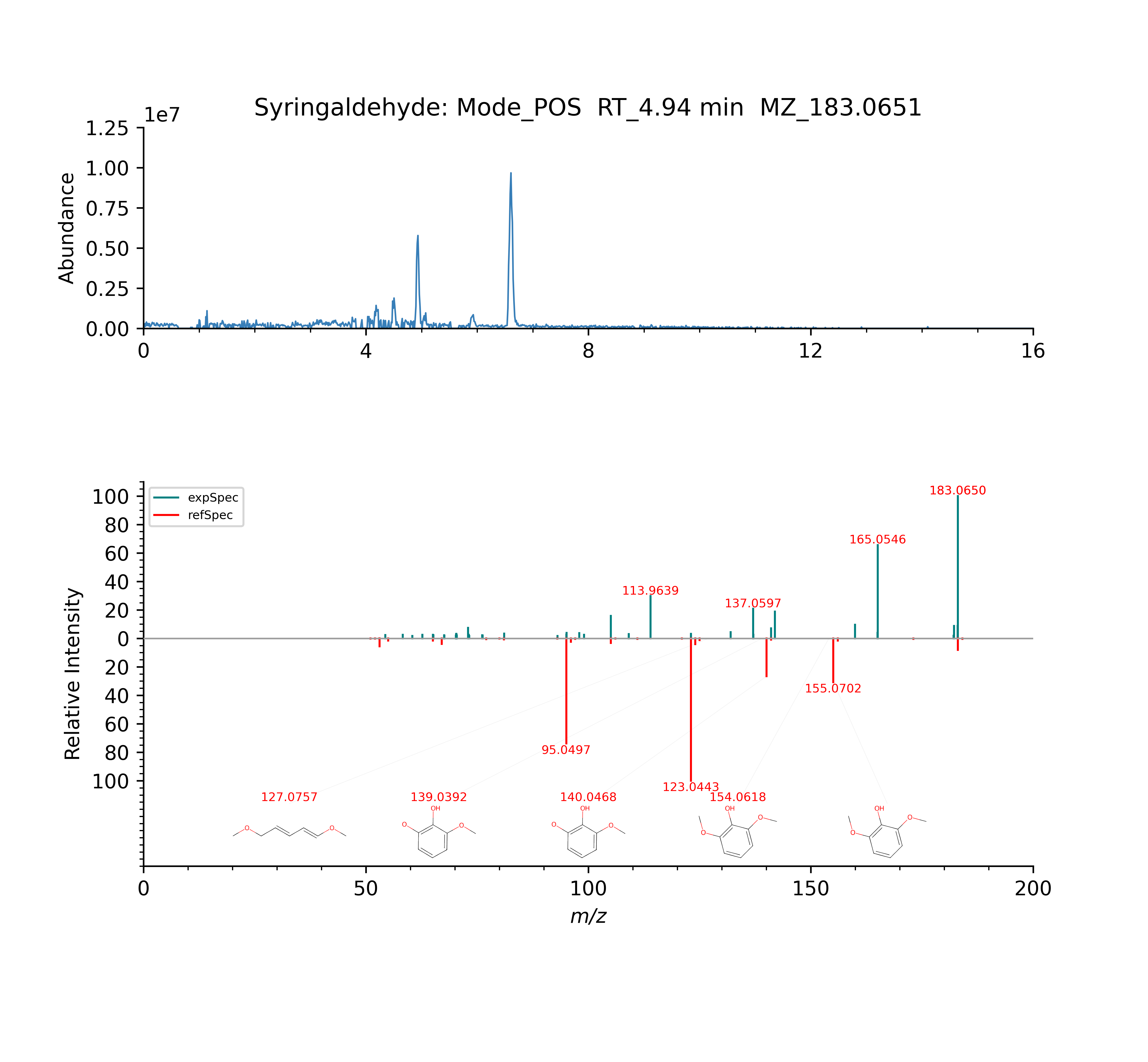

Supplement: Supplementary file 1 [file ijms-27-02203-s001.zip › ijms-4070482 Supplementary/Metabolite List Identified by LC-MS_MS from Rhodiola Species/28.png]

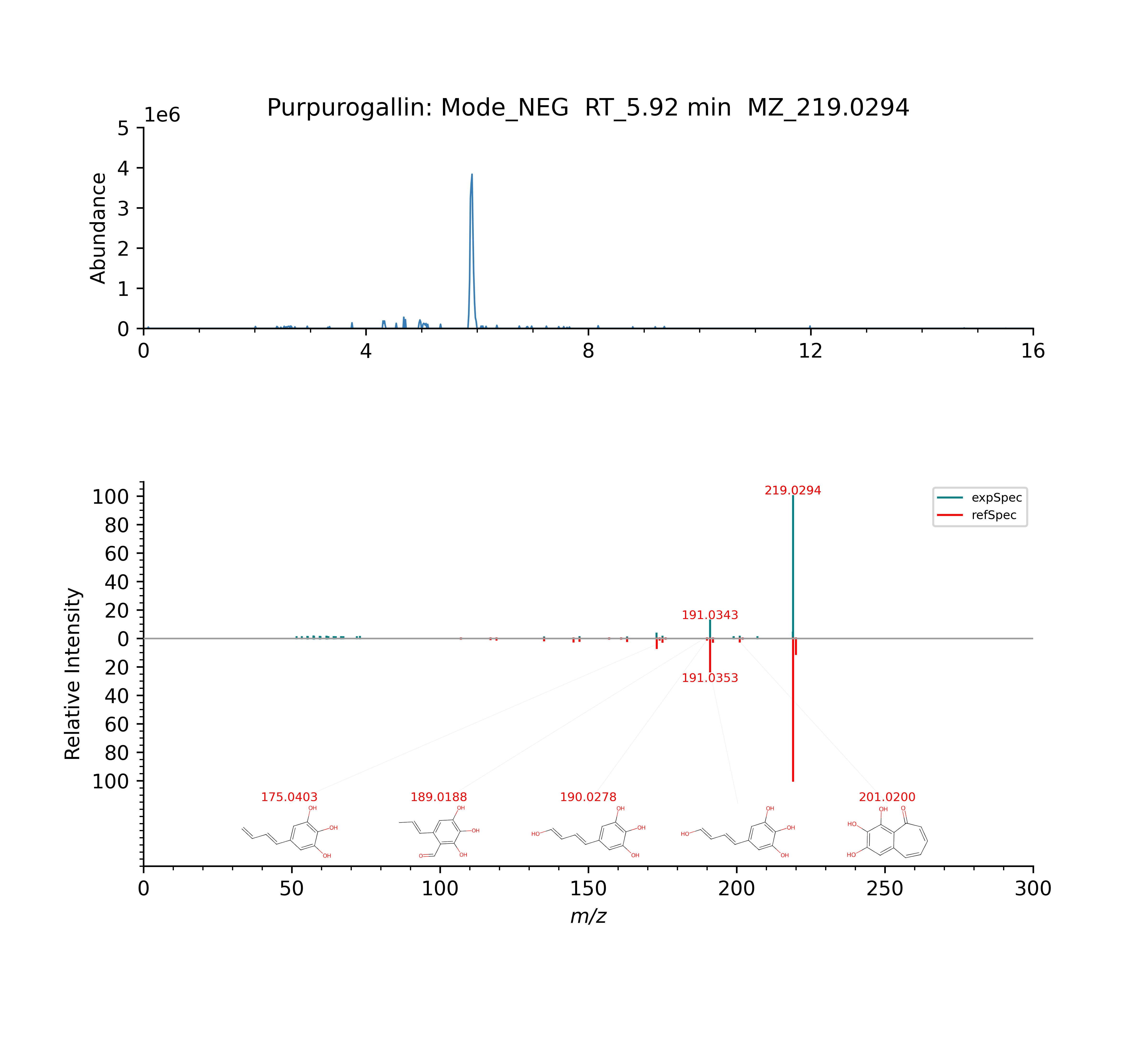

Supplement: Supplementary file 1 [file ijms-27-02203-s001.zip › ijms-4070482 Supplementary/Metabolite List Identified by LC-MS_MS from Rhodiola Species/29.png]

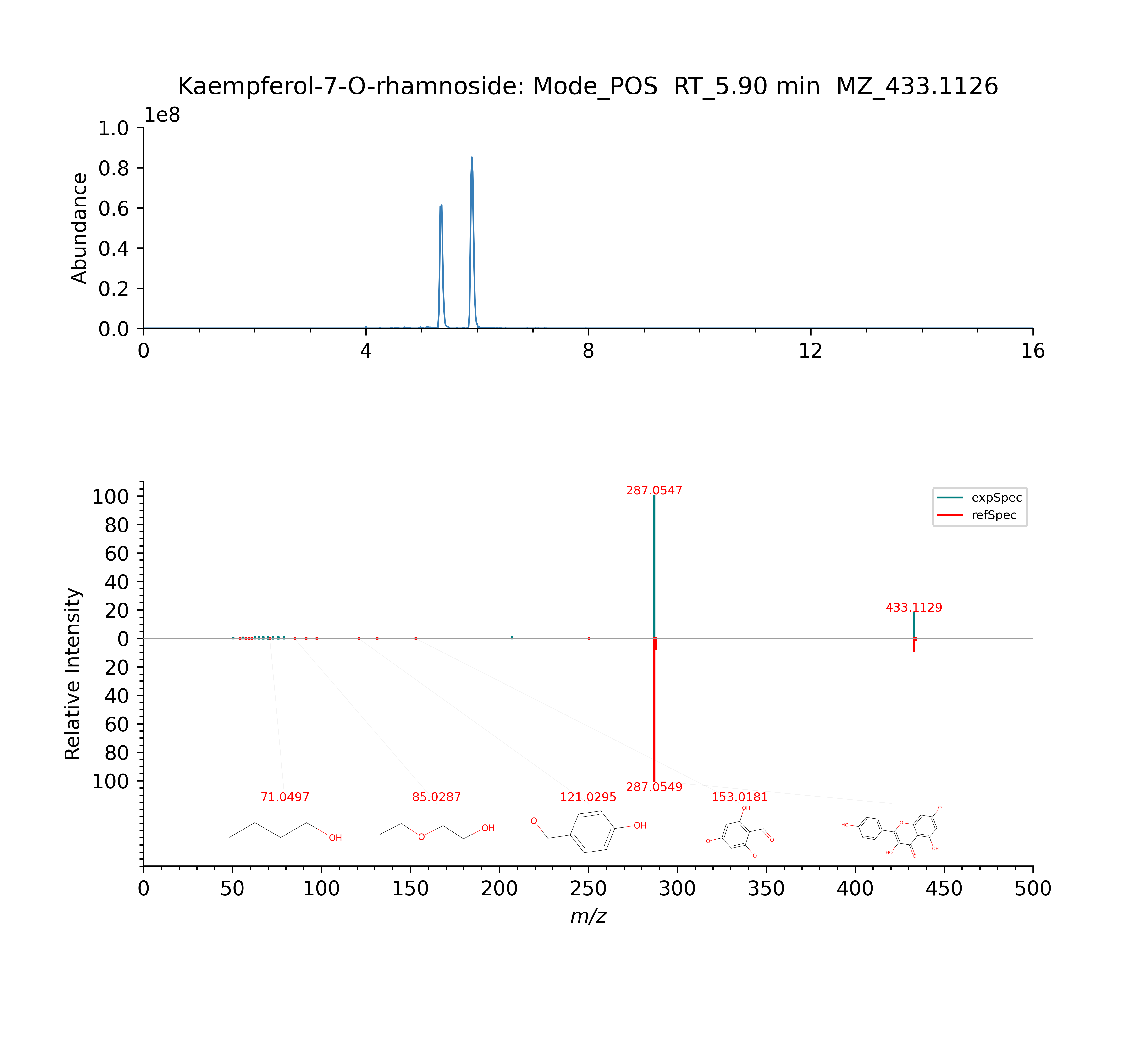

Supplement: Supplementary file 1 [file ijms-27-02203-s001.zip › ijms-4070482 Supplementary/Metabolite List Identified by LC-MS_MS from Rhodiola Species/3.png]

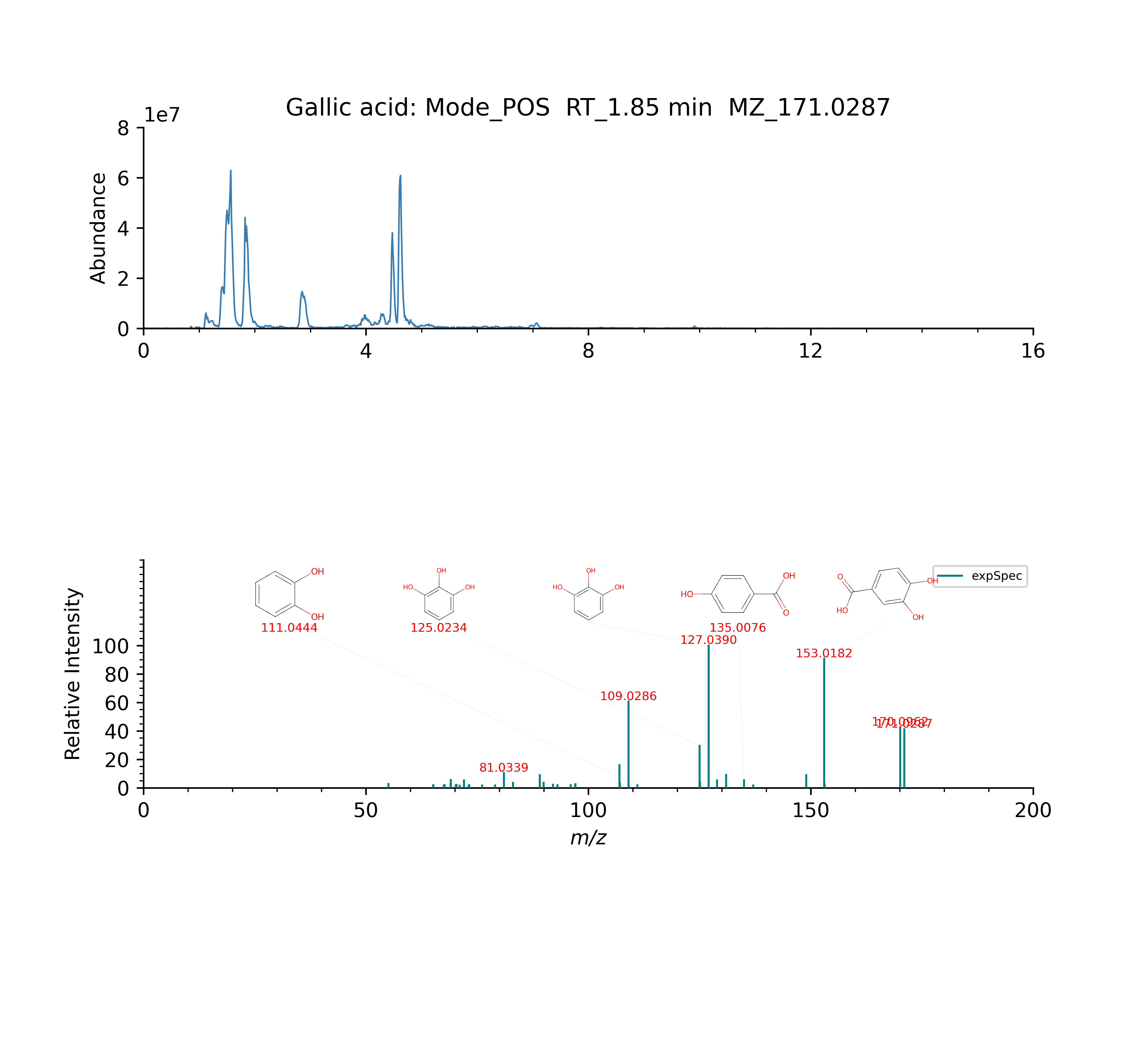

Supplement: Supplementary file 1 [file ijms-27-02203-s001.zip › ijms-4070482 Supplementary/Metabolite List Identified by LC-MS_MS from Rhodiola Species/30.png]
